# GRable Version 1.0: A Software Tool for Site-Specific Glycoform Analysis With Improved MS1-Based Glycopeptide Detection With Parallel Clustering and Confidence Evaluation With MS2 Information

**DOI:** 10.1016/j.mcpro.2024.100833

**Published:** 2024-08-23

**Authors:** Chiaki Nagai-Okatani, Daisuke Tominaga, Azusa Tomioka, Hiroaki Sakaue, Norio Goda, Shigeru Ko, Atsushi Kuno, Hiroyuki Kaji

**Affiliations:** 1 Molecular and Cellular Glycoproteomics Research Group, Cellular and Molecular Biotechnology Research Institute, National Institute of Advanced Industrial Science and Technology (AIST), Tsukuba, Ibaraki, Japan; 2 Department of Systems Medicine, Keio University School of Medicine, Shinjuku, Tokyo, Japan; 3 Institute for Glyco-core Research (iGCORE), Nagoya University, Nagoya, Aichi, Japan

**Keywords:** glycoproteomics, site-specific glycoform, glycan heterogeneity, retention time, software

## Abstract

High-throughput intact glycopeptide analysis is crucial for elucidating the physiological and pathological status of the glycans attached to each glycoprotein. Mass spectrometry–based glycoproteomic methods are challenging because of the diversity and heterogeneity of glycan structures. Therefore, we developed an MS1-based site-specific glycoform analysis method named "Glycan heterogeneity-based Relational IDentification of Glycopeptide signals on Elution profile (Glyco-RIDGE)" for a more comprehensive analysis. This method detects glycopeptide signals as a cluster based on the mass and chromatographic properties of glycopeptides and then searches for each combination of core peptides and glycan compositions by matching their mass and retention time differences. Here, we developed a novel browser-based software named GRable for semi-automated Glyco-RIDGE analysis with significant improvements in glycopeptide detection algorithms, including "parallel clustering." This unique function improved the comprehensiveness of glycopeptide detection and allowed the analysis to focus on specific glycan structures, such as pauci-mannose. The other notable improvement is evaluating the "confidence level" of the GRable results, especially using MS2 information. This function facilitated reduced misassignment of the core peptide and glycan composition and improved the interpretation of the results. Additional improved points of the algorithms are "correction function" for accurate monoisotopic peak picking; one-to-one correspondence of clusters and core peptides even for multiply sialylated glycopeptides; and "inter-cluster analysis" function for understanding the reason for detected but unmatched clusters. The significance of these improvements was demonstrated using purified and crude glycoprotein samples, showing that GRable allowed site-specific glycoform analysis of intact sialylated glycoproteins on a large-scale and in-depth. Therefore, this software will help us analyze the status and changes in glycans to obtain biological and clinical insights into protein glycosylation by complementing the comprehensiveness of MS2-based glycoproteomics. GRable can be freely run online using a web browser via the GlyCosmos Portal (https://glycosmos.org/grable).

## Highlights

• GRable employs MS1-based glycopeptide detection method for improving sensitivity.
• GRable visualizes site-specific glycoforms of a glycopeptide as a cluster.
• Glycopeptides are matched with peptides and glycans using mass and retention time.
• The most plausible matches are selected by multi-evidence including MS2 evaluation.
• This web tool for glycoproteomics is freely available at the GlyCosmos Portal.

## Graphical Abstract

**Figure:**
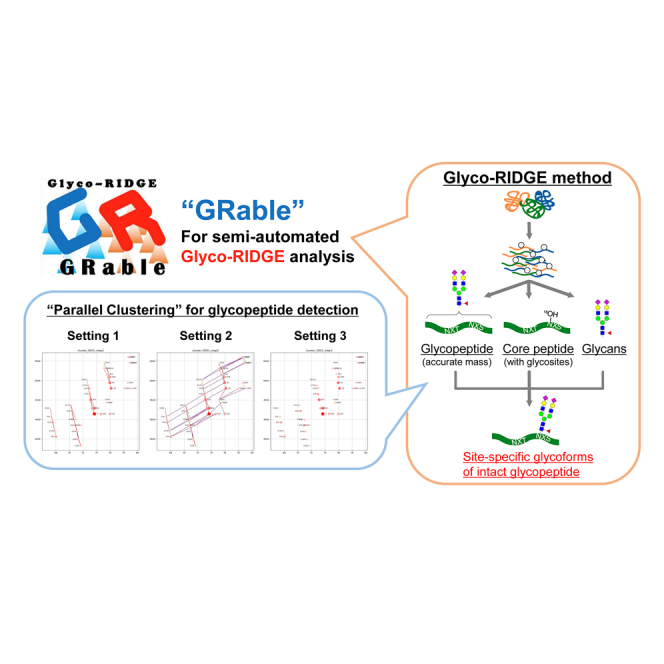

## In Brief

GRable is a novel software for semi-automated site-specific glycoforms analysis, balancing between coverage and accuracy of glycopeptides. GRable employs MS1-based glycopeptide detection method for efficient and comprehensive finding of glycopeptides using a "parallel clustering" function, ensuring accuracy of MS1-based estimations by selecting and evaluating using MS2 information as a "confidence level." GRable serves as complementary roles to MS2-based glycoproteomics for expanding glycopeptide detection and providing supportive evidence of MS2-based identifications. This web tool is freely available at the GlyCosmos Portal.

Protein glycosylation is a common and complex posttranslational modification in eukaryotes (1) that plays pivotal roles in various biological processes (2). This posttranslational modification regulates the function and localization of glycoproteins by modulating their tertiary structures and interactions with other molecules (2). However, revealing the glycan structure–function relationship is challenging because glycan structures are highly diverse and heterogeneous and differ in organisms, cells, proteins, and the attached sites (3). Furthermore, the posttranslational modification are altered depending on the cell state through differentiation, carcinogenesis, infection, nutrition, medicine, and stimuli (3). Glycans are naturally heterogeneous and elucidating their function by uncovering the state and changes in glycans with heterogeneities at each level is important. The heterogeneity levels of glycans include the micro (glycan variation of one glycosite), macro (with or without glycosylation of one glycosite), and meta (glycan variation of the entire glycoprotein molecule) levels, which are important features responsible for the presence of different proteoforms (4).

Although current improvements in mass spectrometry (MS) have made it possible to perform an in-depth structural analysis of the peptide backbones and glycomes of glycopeptides, elucidating the glycan compositions of specific sites for proteins with multiple glycosylated sites (i.e., micro-heterogeneity) is challenging (5). First, the glycan compositions of each site must be determined to estimate the site-specific glycoforms. For this purpose, the analysis of only glycomes and deglycosylated peptides is insufficient; thus, intact glycopeptides must be analyzed directly. Current standard glycoproteomic approaches rely on MS2 spectra to identify site-specific glycoforms (6). For MS2-based glycoproteomics, the commercial software Byonic (7) is often used (6) and many software programs have been developed (8, 9, 10, 11, 12, 13, 14, 15, 16, 17, 18, 19, 20). However, these MS2-based methods have a critical problem in that they require glycopeptide fragmentation, leading to lower detection sensitivity than the corresponding naked peptides. The decreased detection sensitivity results from the lower ionization efficiency of glycopeptides, which is notable, especially for sialylated glycopeptides because of their negative properties. In addition, glycan heterogeneity decreases the abundance of each glycopeptide. Therefore, in some MS1 spectra, several signals are not selected for MS2 analysis, and frequently, MS2 spectra do not contain sufficient information for confident glycopeptide identification.

**Figure 1:**
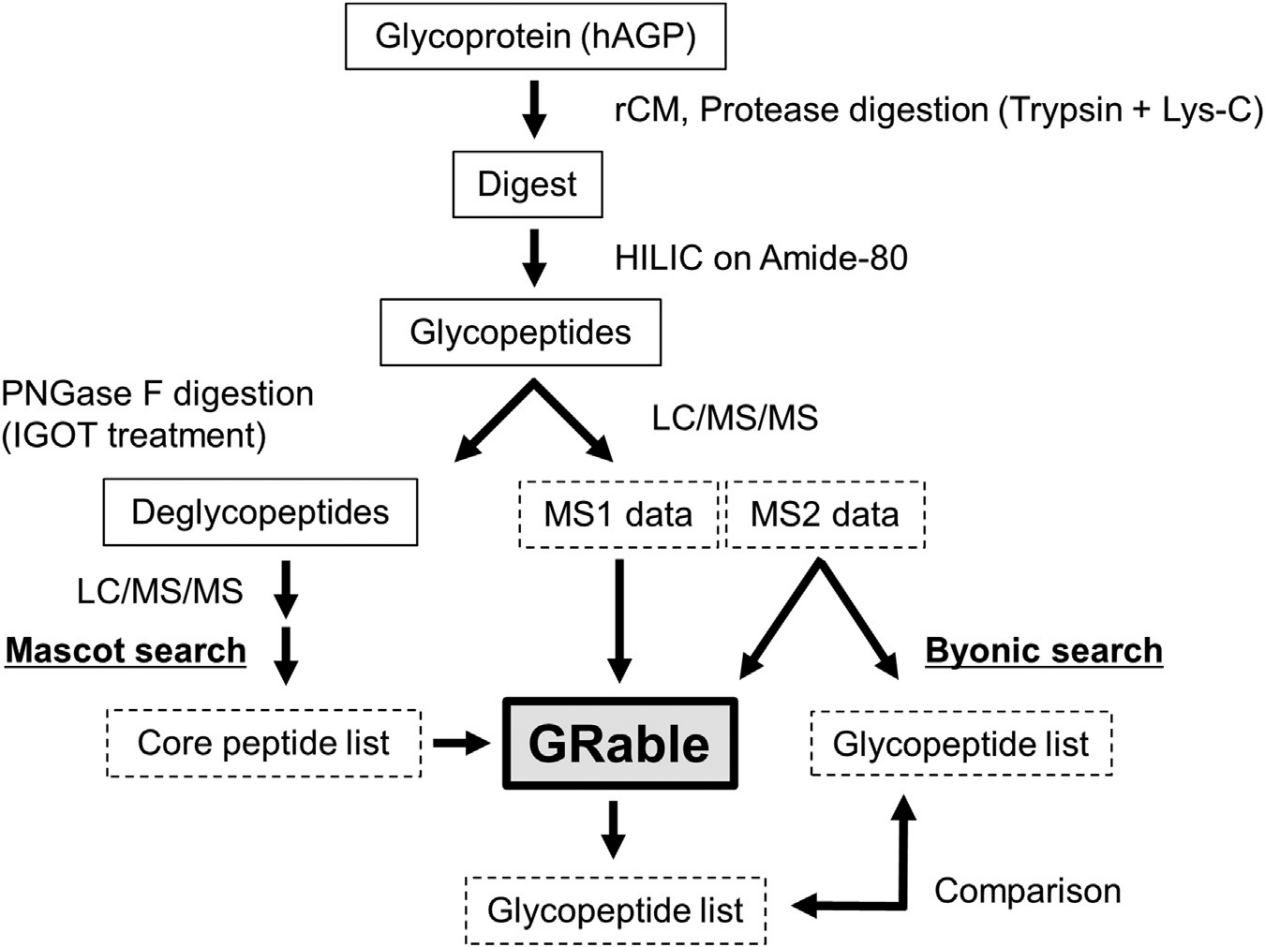
**Workflow of glycoproteomic analysis for hAGP using GRable.** hAGP was subjected to reduction and carbamidomethylation (rCM) using DTT and iodoacetamide, respectively, and digested with trypsin and Lys-C endopeptidase. The digest was used in hydrophilic interaction chromatography (HILIC) on an Amide-80 column to capture glycopeptides. A small aliquot of the glycopeptide fraction was treated with peptide-*N*-glycosidase F (PNGase F) in ^18^O-labeled water to remove *N*-glycans and to label deglycosylated Asn as ^18^O-labeled Asp (isotope-coded glycosylation site-specific tagging; IGOT) (25, 26). The IGOT-treated deglycopeptides were analyzed using liquid chromatography-tandem mass spectrometry (LC/MS/MS), and the MS2 data were used for database search using Mascot to prepare a core peptide list. In parallel, another aliquot of the glycopeptide fraction was analyzed through LC/MS/MS. MS1 data of the glycopeptide analysis underwent GRable analysis. GRable used the core peptide (Supplemental Table S1) and glycan composition (Supplemental Table S2) lists to assign each glycopeptide signal, whereas MS2 data were used for adding the confidence level to the GRable results. Glycopeptide analysis MS2 data were also used for glycopeptide estimation using Byonic. The resulting glycopeptide lists of GRable and Byonic analyses were compared.

To overcome this limitation, we developed an MS1-based glycoproteomic approach named "Glycan heterogeneity-based Relational IDentification of Glycopeptide signals on Elution profile" (Glyco-RIDGE) (21), which was first introduced in 2015 (22). One limitation of MS1-based approaches is that combinations of core peptides and glycans must be "estimated," leading to the possibility of misassignment because of isobaric combination issues caused by adducts and core peptide modifications observed even in MS2-based approaches (23, 24). To minimize such errors, a general workflow of the Glyco-RIDGE analysis using liquid chromatography/mass spectrometry (LC/MS) data of glycopeptide samples requires information on the core peptides present in the analyte, as well as presumed glycans (Fig. 1); these sequences and *N-*glycosites can be identified by isotope-coded glycosylation site-specific tagging (IGOT)-LC/MS/MS (25, 26), in which *N*-glycosites are specifically labeled by peptide-*N*-glycosidase F (PNGase F)-mediated incorporation of a stable isotope tag (^18^O). In the Glyco-RIDGE workflow, MS1 signals derived from glycopeptides with the same core peptide are assigned as a cluster in the LC/MS data based on their mass differences and closeness of retention times (RTs). Then, the combination of the core peptide and glycan composition for these glycopeptides is annotated by matching the observed accurate glycopeptide mass with the sum of the masses of the pre-identified core peptide candidates and presumed glycans. A notable feature of Glyco-RIDGE is the detection of glycopeptide signals based only on MS1 data, in contrast to other MS1-based approaches that employ MS2 data to identify at least one glycopeptide for each core peptide (27, 28, 29, 30, 31). With this feature, the Glyco-RIDGE method is expected to achieve a more high-sensitive and comprehensive analysis of protein glycosylation than current fragmentation-dependent identification methods. Notably, a previous study (32) demonstrated that among the MS2 spectra assigned using the Glyco-RIDGE method and Byonic search, the ratio of the same assignment was quite high (349/353 = 98.9%), demonstrating that the reliability of this method is similar to that of Byonic.

The feasibility and usefulness of the Glyco-RIDGE method have been demonstrated using in-house software. It was applied to analyze complex glycoprotein mixtures after the removal of sialic acids (22, 32). Subsequently, this method has been utilized for site-specific glycoform analysis of intact (i.e., sialylated) glycopeptides (32, 33, 34, 35, 36), revealing site-specific "glycostem" and "glycoleaf" features (36). However, the prototype software has limitations. One technical issue was the low accuracy of the monoisotopic signal detection from MS1 data, resulting in a reduced number of detected glycopeptide signals. Additionally, clusters of glycopeptides with the same core peptide but different numbers of sialic acid residues were detected separately, hindering the identification and interpretation of micro-heterogeneity. Another issue was the presence of detected glycopeptide clusters that were not assigned, potentially due to adducts or peptide modifications; however, the reason remains unclear and needs improvement. Furthermore, the process of selecting the most plausible match for each glycopeptide cluster as the final assignment and evaluating the assignments manually through searching the MS2 spectra is time-consuming. It is also challenging to optimize search parameter settings and acceptance criteria while maintaining a balance between coverage and accuracy in the analysis, which is crucial for MS2-based approaches (37).

The present study introduces a novel software named "GRable" designed to semi-automatically conduct the Glyco-RIDGE analysis with various technical enhancements. Key features include "parallel clustering" for efficient and thorough detection of glycopeptide clusters, as well as the addition of a selection step to choose and assess assignments using MS2 data as a "confidence level." Some additional improvements include a "correction function" for precise monoisotopic peak picking, ensuring a one-to-one correspondence between clusters and core peptides even for multiply sialylated glycopeptides and an "inter-cluster analysis" function to determine the cause of detected but unmatched clusters. To demonstrate the feasibility and utility of this software, we analyzed MS data obtained with intact glycopeptides prepared from human, α1-acid glycoprotein (hAGP), a glyco-biomarker candidate for liver fibrosis (38), as a model of targeted analysis. In addition, we used data on glycopeptides obtained from human promyelocytic leukemia-60 (HL-60) cell lysates (32), as a crude sample model.

## Experimental Procedures

**Figure 2:**
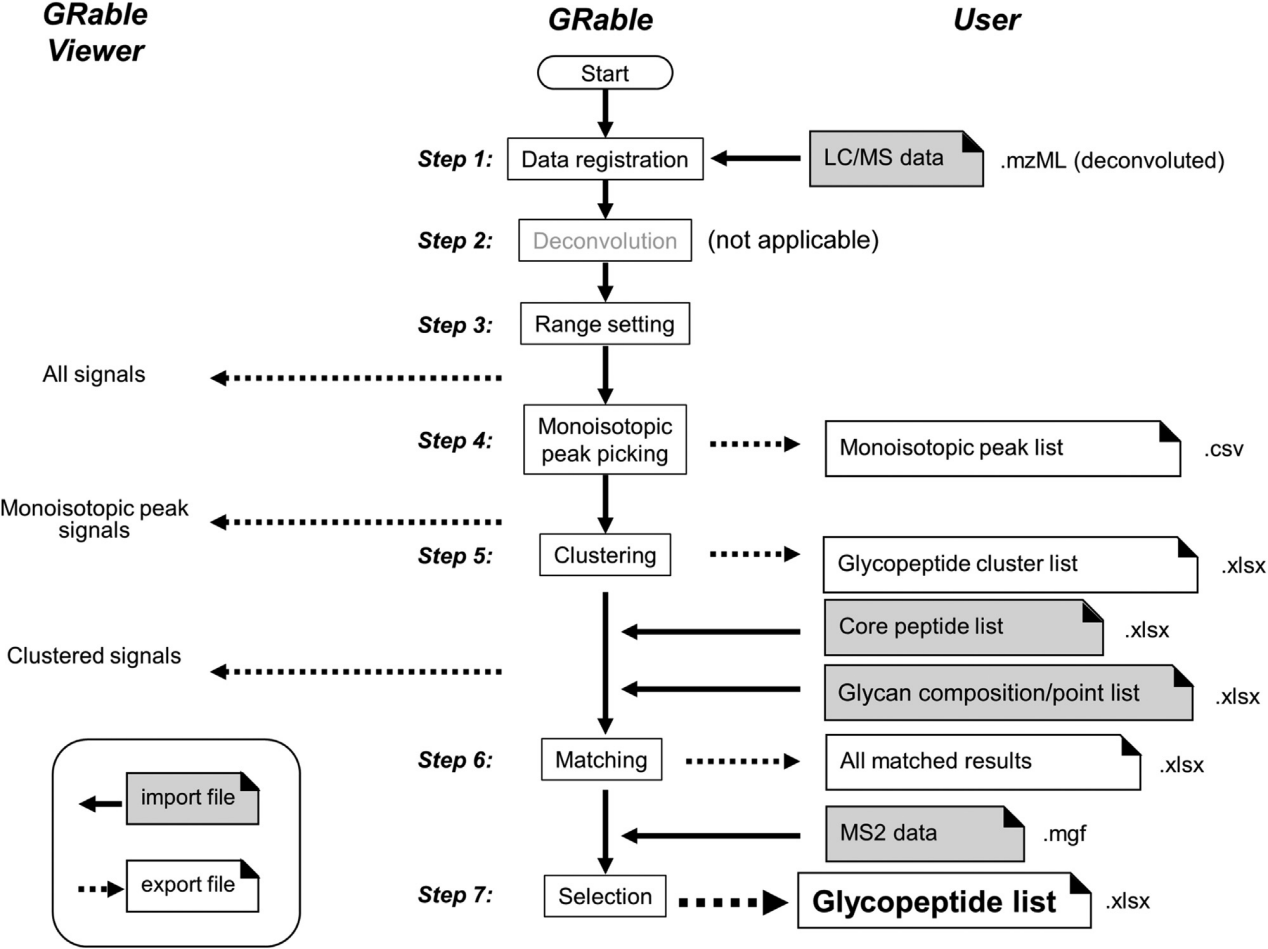
**Overview of data processing by GRable.** GRable proceeds in seven steps using the four files indicated in *gray* font. In step 1, GRable allows the registration of the LC/MS data of glycopeptides in the mzML format, which ensures great versatility and ease of data processing. In the current version, the deconvolution function (Step 2) is not applicable although it is visible in the user interface for future implementation. Step 3 was designed to set the RT range, mass range, and minimum signal intensity (threshold) over which the analysis was performed. Step 4 was intended to find and group signals of an identical ion based on three parameters: time (scan), mass (MH+), and intensity and to obtain the monoisotopic mass of each signal group at the peak time, using our unique algorithms. In step 5, a series of signals from the glycopeptide group were found as a cluster based on the elution behavior and mass difference of its members. In step 6, GRable searches for a combination of core peptides and glycan compositions that match the mass of the putative glycopeptide within the allowed mass error (user setting) according to the following equation: Observed M(glycopeptide) = calculated M(core peptide identified) + M(Hex)∗i + M(HexNAc)∗j + M(dHex)∗k + M(NeuAc)∗l (M is a mass value, and i, j, k, and l are integers). In step 7, the most plausible combination among multiple candidate combinations suggested for one glycopeptide cluster was selected. Subsequently, their reliability at the cluster level as well as that at the single glycopeptide level was evaluated using information from the results and additional MS2 information. The results of steps 3 to 5 were visually confirmed using a viewer in the main window of the software. The detailed results of steps 4 to 7 can be exported as an Excel file with each setting, as shown for hAGP (Data S1 and S2) and HL-60 cell lysates (Data S3 and S4).

GRable version 1.0 requires the following four files: LC/MS data of glycopeptides after deconvolution (.mzML); a core peptide list (.xlsx); a glycan point list (.xlsx); and LC/MS/MS data for glycopeptides (.mgf) (Fig. 2). Details of the input files and their preparation are described below and in the user manual (Document S1). Note that this version of GRable is guaranteed only for Thermo Fisher Scientific data.

### Preparation of hAGP Glycopeptide Samples

To prepare large-scale analytes for repeated verification, 5 mg of freeze-dried hAGP (Sigma-Aldrich) was dissolved in Milli-Q water (500 μl) and the aliquot was denatured with 0.1% Rapigest (Waters) in 50 mM Tris–HCl, reduced with DTT (Fujifilm Wako), alkylated with iodoacetamide (Fujifilm Wako), and digested with Lys-C (1/200 weight of protein; Fujifilm Wako) and sequencing grade modified trypsin (1/100 weight of protein; Promega, Madison) at 37 °C overnight in 0.1% Rapigest. After digestion, Rapigest was cleaved by acidification with 0.1% trifluoroacetic acid (TFA; Fujifilm Wako). Glycopeptides from hAGP tryptic digests were then captured using an Amide-80 column (TSK-gel Amide-80, 2 × 50 mm; TOSOH) equilibrated with 75% acetonitrile (Merck)/0.1% TFA and eluted isocratically with 50% acetonitrile/0.1% TFA after washing. To prepare the sialidase-treated hAGP, an aliquot (5 μg) of the glycopeptide sample was treated with α2,3,6,8-sialidase (50 units; New England BioLabs) at 37 °C overnight in an attached buffer (GlycoBuffer1).

### LC/MS/MS Analysis of hAGP Glycopeptide Samples

Glycopeptides were analyzed using an LC-electrospray ionization-MS system equipped with a nanoflow LC system (UltiMate-3000; Thermo Fisher Scientific) and a tandem mass spectrometer (Orbitrap Fusion Tribrid mass spectrometer; Thermo Fisher Scientific). Glycopeptides were trapped on a C18 cartridge column (Acclaim PepMap100 C18, 0.3 mm I.D. × 5 mm; Thermo Fisher Scientific) and separated on a C18 tip column (75 μm I.D. × 15 cm, 3 μm particle; Nikkyo Technos) using 2 to 36% acetonitrile/0.1% formic acid (Fujifilm Wako) gradient (90 min) at a flow rate of 300 nl/min. The ionization voltage was 2.0 kV (positive), and the temperature of the ion transfer tube was 275 °C. Data were acquired using the data-dependent mode (cycle time: 2.5 s) with internal mass calibration (lock mass) at 445.12003. The mass range was 377–2000 m/z (MS1) and 135–2000 m/z (MS2). The mass resolutions were 120,000 (MS1, Orbitrap) and 15,000 (MS2, Orbitrap). The fragmentation mode was higher-energy collision-induced dissociation with stepped collision energies of 25, 30, and 35%. The acquired raw data file was deconvoluted using the Xtract of Proteome Discoverer (ver. 2.4, Thermo Fisher Scientific) and exported as an mzML file for GRable analysis.

For the in-silico spike-in test, we analyzed 0.25 μg of HeLa lysate (HeLa protein digest standard; Thermo Fisher Scientific) in LC/MS/MS under a similar condition as hAGP. Subsequent data processing and GRable analysis were also performed similarly as the case for hAGP.

### IGOT-LC/MS/MS Analysis of hAGP Glycopeptide Samples

IGOT-LC/MS analysis was performed for core peptide identification as reported previously (25, 26). Glycopeptides were treated with PNGase F (Takara Bio) in 50 mM TriswHCl, pH 8.5, prepared with H_2_^18^O (isotope purity: 95%; Taiyo Nippon Sanso) at 37 °C overnight. Deglycosylated peptides (IGOT peptides) were analyzed before glycopeptide analysis using LC/MS/MS under the same conditions as for the glycopeptide sample.

### Preparation of a Core Peptide List

The format of the core peptide list required by the software was fixed and created in a manner similar to that used in this study (Supplemental Table S1). The details of the preparation are documented in the manual (Document S1, Section 4). Items minimally required for preparing a core peptide list are the calculated mass, retention time of each peptide, and at least one peptide identifier such as the core peptide number. In the matching step, such information is used to identify potential glycoforms of the core sequences listed by calculating the mass differences within the limited RT range. For the identification of core peptides, IGOT-LC/MS analysis, as described above, is recommended to confirm their *N*-glycosylations, but it is possible to list the peptides identified by proteomic analysis of deglycopeptides (i.e., the digested mixture after glycan removal). Confirmation of the presence of core peptides for glycoproteins is crucial for crude samples. For the analysis of a highly purified glycoprotein, it is also possible to list the peptides predicted based on protease digestion patterns. It is also possible to create a core peptide list based on glycopeptides identified in advance by MS2-based glycoproteomics, to obtain additional potential glycoforms.

For hAGP, the raw data acquired by IGOT-LC/MS/MS were converted to mgf using Mascot Distiller (ver. 2.7.0; Matrix Science), and searched using Mascot (ver. 2.5.1; Matrix Science) using a human protein sequence file (SwissProt_UniProtKB_isoform; downloaded in April 2019; entry: 42,431). The search parameter settings were as follows: enzyme: trypsin (full or semi); missed cleavage: 2; mass tolerance: 7 ppm (MS1) and 0.02 Da (MS2); fixed modification: carbamidomethyl (C); variable modifications: Gln to pyro-Glu (peptide N-term, Q), ammonia-loss (peptide N-term, carbamidomethyl C), oxidation (M), and Delta: H(-1)N(-1)18O(1) (N); and target false discovery rate: 1%. Mascot search results with a peptide rank of 1 and peptide expectation value of <0.05 were selected. Matched sequences containing IGOT modifications at Asn on the consensus sequence for *N*-glycosylation (Asn-Xaa-[Ser/Thr]; Xaa is not Pro) obtained using trypsin (full) and trypsin (semi) were combined. The resulting glycopeptide list was used to create a core peptide list for GRable analysis. To ensure comprehensive analysis, hAGP peptide sequences that were not identified in the data used here but found in other analyses were manually added to the list with observed or predicted RTs (Supplemental Table S1). For example, ENGTISR deglycopeptide is hydrophilic and thus cannot always be detected. That is, five core peptides with predicted hAGP sequences were added to the list, and three core peptides were matched with glycopeptides as clusters.

### Preparation of a Glycan Point List

The format of the glycan point list required by the software was fixed and created in a manner similar to that used in this study (Supplemental Table S2). The details of the preparation are documented in the manual (Document S1). In the Matching setting, users can define any permissible glycan compositions (i.e., the type and number of each glycan unit). Four glycan units (Hex, HexNAc, dHex, and NeuAc) were considered as the glycan components attached to hAGP. *N*-glycan compositions were expressed as the generic monosaccharide composition as "Hex(∗)HexNAc(∗)dHex(∗)NeuAc(∗)", where ∗ represents the number of individual monosaccharide residues. The default settings were as follows: Hex: 0 to 12, HexNAc: 1 to 12, dHex: 0 to 4, and NeuAc: 0 to 4. The maximum numbers of Hex and HexNAc residues were determined using the approximate masses of the ionizable glycopeptides. The maximum number of NeuAc residues was limited to four because of our sample preparation conditions, which did not allow the retention of any remaining oligo- or polysialic acids. The number of dHex (fucose) was also limited to four or fewer because the mass of dHex(5) is similar to that of Hex(2)HexNAc(2) (difference = 0.025), corresponding to approximately 5 ppm for a 5000 Da molecule. If the measurement is performed with an accuracy greater than 2 ppm, it may be possible to distinguish between five or more fucoses. Within the range of glycan compositions, users can provide arbitrary points for likely compositions in the glycan list. In contrast, among the possible compositions, many are considered unlikely based on biosynthetic pathways, and matches with such unusual compositions can be considered incorrect. Accordingly, users can also define unusual compositions in the glycan list and such incorrect matches are marked in the match results by giving negative points and thus, will be considered in the selection.

The list presented here (Supplemental Table S3) is recommended for human samples and thus does not feature NeuGc. The list for hAGP analysis was almost identical to that for the analysis of the human *N*-glycome supplied by Byonic, except for one composition (Hex(7)HexNAc(6)), which was manually added because this composition is considered common in the human *N*-glycome. In this list, 139 glycan compositions were assigned one point if the composition matched. The list contained glycan compositions in *N*-glycan major biosynthetic pathways, from the glycan produced by oligosaccharide transferase to the glycan processed by many glycosyltransferases and glycosidases. The largest is Hex(7)HexNAc(6)dHex(4)NeuAc(4), which corresponds to a tetra-antennary glycan with four Fuc and four NeuAc. The smallest is HexNAc(1), the core GlcNAc retained on Asn. Unusual compositions were defined based on *N*-glycan biosynthetic pathways; however, negative points were not assigned for such unusual compositions in this study.

### Byonic Search for hAGP Data

Glycopeptide MS2 data were searched using the Byonic search engine ver. 2.15.7 (Protein Metrics) using the human protein sequence file from SwissProt (downloaded on May 19, 2020; entry: 42,296) and the glycan point list (Supplemental Table S3). Search parameter settings were as follows: enzyme: Trypsin_KR (Full or Semi), max missed cleavages: 2, static modifications: carbamidomethyl (C), dynamic modifications: ammonia-loss (N-term C), Gln to pyro-Glu (N-term Q), oxidation (M), peptide mass tolerance: ±3 ppm, fragment mass tolerance: 0.02 Da. From the search results, the estimated glycopeptides with Confidence = High and Byonic Score ≥200 were selected to compare GRable results. The two results obtained with Trypsin_KR (full) and Trypsin_KR (semi) were combined, and the resultant glycopeptide list was compared with the GRable results.

### GRable Analysis of HL-60 Cell Data

LC/MS/MS data for HL-60 cell lysates were obtained from a previous study (Supplemental Fig. S1) (32). A protease digest of the HL-60 cell lysate was prepared in a manner similar to that for hAGP. An aliquot of the digest was acidified and heated to remove sialic acid, decreasing glycan heterogeneity and increasing the relative abundance of each glycopeptide. An aliquot of the desialylated glycopeptide fraction was subjected to hydrophilic interaction chromatography (HILIC) to collect four glycopeptide fractions, which were then analyzed using LC/MS/MS and IGOT-LC/MS/MS to obtain glycopeptide and deglycopeptide MS data, respectively. The glycans obtained from the IGOT procedure were analyzed using matrix-assisted laser desorption/ionization time-of-flight MS to prepare a glycan point list, in which positive points were given to the glycans present in the sample, for increasing the reliability of the GRable results. Because HL-60 cell data were for desialylated glycopeptides, only three glycan units (Hex, HexNAc, and dHex) were considered glycan components. The definition of the unusual composition was the same as that used for the hAGP. The resulting core peptide list (Supplemental Table S4) and glycan point list (Supplemental Table S5) for fraction 2 were used for the GRable analysis.

### Software Implementation

GRable was implemented as a web application by Hitachi Solutions Technology Ltd. The user interface was mostly written in JavaScript, data management in Java, and scientific calculations in Python. PostgreSQL was used as the background for user and data file management. The software was developed and tested on Ubuntu Linux 22.04 LTS.

## Results and Discussion

GRable is a web application that users can access through web browsers. Notably, the deconvoluted LC/MS data must be uploaded in the current version. For data processing (Fig. 2), all steps were executed step-by-step after uploading the required data and setting the appropriate parameters. A detailed instruction manual for GRable is provided in Document S1. The data processing, search parameter settings, and results of each step are described below, primarily focusing on the improved aspects over the prototype.

### Monoisotopic Peak Picking with Improved Accuracy Using the Correction Function

After the ranges of RT and mass were set in the Range setting step, monoisotopic peaks were selected using a new algorithm in this step. In a previous in-house prototype, the search for monoisotopic peaks started from the highest signal, which is time-consuming. Therefore, GRable uses a new algorithm to improve the speed of data processing. First, a filter of five scans × 5 Da was used to search for a local peak in each group comprising a single ion cluster. When the highest signal was centered in the filter, it was recorded as a local peak, and the other signals within the filter were excluded from the local peak candidates. The local peaks were used as the starting points for identifying the corresponding groups comprising the same ion cluster. The local peak signal spectrum was integrated with the same ion spectra before and after the scans, and the resulting spectrum was used to determine the monoisotopic signal. This algorithm change considerably accelerates the monoisotopic peak-picking process.

In addition, the algorithms for finding monoisotopic signals were also modified to improve the accuracy of the monoisotopic assignment. GRable evaluates the selected monoisotopic peaks using a multinomial distribution based on the averagine for a peptide as the abundance ratio of five isotopes, including C, H, N, O, and S. By fitting the observed spectrum with the calculated spectrum, the need to correct the monoisotopic signal obtained in the preceding step is suggested. This correction function facilitated an increased number of matched glycopeptide group members compared to the GRable results obtained without this function. Users can confirm corrected monoisotopic peaks by the update flag = 1 in a "Monoiso Peak List" sheet and in other result sheets of subsequent steps, where the peak no. was identical to that indicated in the monoisotopic peak list (Data S2). For hAGP, 5369 monoisotopic signals were detected, of which 2140 were automatically corrected by the correction function, demonstrating its utility for the accurate selection of monoisotopic signals.

### Clustering for Efficient and Comprehensive Detection of Glycopeptide Signals Using Parallel Clustering Function

**Figure 3:**
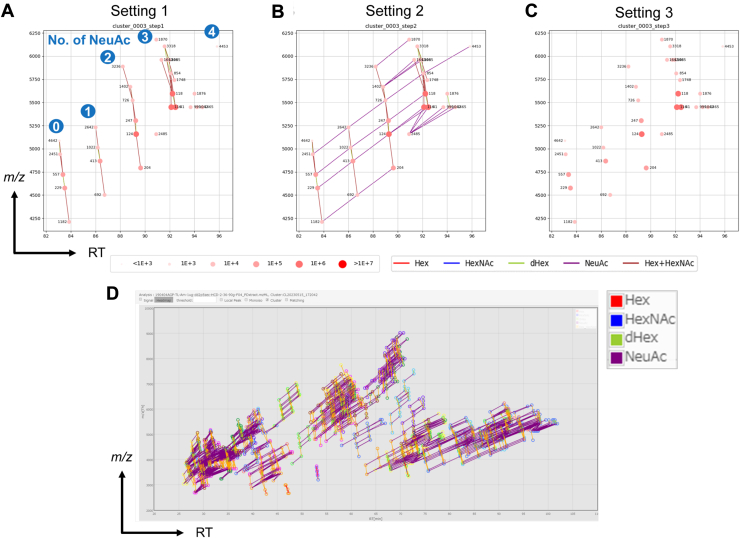
**hAGP clustering results.** Representative results (Cluster 3) of parallel clustering with three search parameter settings: *A*, setting 1 (a setting in our previous prototype), (*B*) setting 2 (an extra setting for detecting sialylated glycopeptides), and (*C*) setting 3 (an extra setting for detecting oligo-mannosylated glycopeptides). The detailed settings are as follows: for setting 1, neutral saccharides (Hex, HexNAc, dHex, Hex + HexNAc): -1–0 (min), and the minimum number of members: 4 (setting for maximizing No. of detected clusters with minimized misdetection); for setting 2, neutral saccharide: -1–0 (min), acidic saccharide (NeuAc for human): 1 to 4 (min), and minimum number of members: 10 (setting for guaranteeing the accuracy of assignment); and for setting 3, neutral saccharide (Hex only): -1–0 (min), and minimum number of members: 3. Dots indicate the monoisotopic masses of the glycopeptide signals at each RT peak, and the size and color depth of the dot indicate the signal intensity. *Deep red* and larger dots indicate high intensity, and small *pale red dots* indicate low intensity. All the clusters for hAGP shown in Data S2 can be visualized in the viewer of the GRable main window (*D*). The colors of lines that connect the signals indicate the type of glycan unit between the signals: *red*: Hex, *blue*: HexNAc, *green*: dHex, Hex + HexNAc: *brown*, and NeuAc: *purple*.

In this step, tentative glycopeptide signals were identified as a cluster from the monoisotopic peaks selected in the previous step, based on the relationship between the masses and retention times. The prototype could set a single RT range to find the glycopeptide group as a cluster because the time difference for extending the neutral monosaccharides, namely Hex, HexNAc, and dHex, was considered. Glycopeptide groups with different numbers of acidic saccharides, such as NeuAc, were obtained as different clusters (33) because adding neutral saccharides slightly shortens the RT, whereas adding acidic sugar units increases it (39). In the previous RT setting, as the acidic shift increased in proportion to the number of sialic acid units, four glycopeptide clusters with asialo-, monosialo-, disialo-, and trisialo-glycans were found separately in a representative hAGP cluster (Fig. 3A). Therefore, the glycopeptide groups with the same core but different numbers of acidic units cannot be combined. To improve this, GRable was constructed to allow setting the RT difference separately for each acidic and neutral saccharide and any motif such as Hex + HexNAc (LacNAc). In the current version, four clusters with the same core peptide were successfully connected into a single cluster because the range of RT shifts for each unit could be set separately (Fig. 3B). This improvement facilitated a better understanding of the micro-heterogeneity of a glycosite by visualizing the signal intensity of each glycopeptide plot. Notably, a new tetrasialo-glycan was also detected. Such highly sialylated members could not be detected as a cluster because of the limited variety of the glycan stem portion with the four sialylations. Accordingly, this improvement facilitated the increased detection of glycan micro-heterogeneity for one glycosite. In addition, considering the RT difference separately for each glycan unit is also useful for reducing any misdetections caused by isobaric combinations, especially neutral and acidic combinations, such as NeuAc + NH4^+^ and Hex + dHex (24). Similarly, RT also could differentiate between dHex (2) versus NeuAc-containing glycans, even when the monoisotopic mass was not correctly assigned.

As is the case with MS2-based approaches, the optimization of search parameter settings is crucial for obtaining reliable results in glycopeptide identification (40); thus, optimization is required depending on the purpose of analysis. For this need, GRable can integrate multiple clustering results obtained in parallel under different (up to five) search parameter settings for more comprehensive detection of glycopeptide clusters. As the clustering results of each processing step were combined into a single cluster, each search parameter setting was independent of the processing order. This "parallel clustering" function facilitates optimization for the minimum No. of members for clustering, which is the most important parameter that must be optimized according to the purpose of the analysis; a smaller No. of members is expected to result in an increase in the detected clusters. However, the ratio of clusters without supportive information for evaluating certainty will also increase. For example, using this function, the clusters obtained using the previous setting with the No. of members = 4 (setting 1; Fig. 3A), and the current setting with No. of members = 10 (setting 2; Fig. 3B) can be easily compared. When the results with No. of members = 2, 3, and 4 were compared for hAGP, the No. of members = 2 resulted in an increased number of detected clusters. However, most of the increased clusters were excluded in the subsequent selection step (Supplemental Table S6). In addition, manual inspection after the selection step was required with No. of members = 2. In contrast, with No. of members = 3, more assignments were obtained without manual inspection after selection compared to No. of members = 4, demonstrating that the No. of members = 3 is the most suitable for hAGP analysis.

We often use a setting that considers only the number of Hex units to find clusters to detect glycopeptide clusters with only high- or oligo-mannose glycans. There are many possible compositions for complex- and hybrid-type glycans, while most oligo-mannose glycans are M9-M5, limiting the variety of compositions on one *N*-glycosite. Thus, the minimum cluster number was reduced to three to estimate oligo-mannose glycan carriers. In hAGP samples, the abundance of oligo-mannose glycans was low, resulting in no detection in this setting (Setting 3; Fig. 3C). This finding aligns with previous reports showing that glycan structures attached to hAGP are branched, sialylated, fucosylated at the branches, and slightly extended by polylactosamine (41, 42, 43, 44).

The individual time settings for each type of glycan unit and parallel clustering with different settings effectively increased the detection sensitivity, and multiple clusters with the same core but different sialic acid numbers could now be combined into one cluster. With parallel clustering of these three search parameter settings for hAGP, 65 tentative glycopeptide clusters consisting of 1422 potential site-specific glycoforms were identified (Fig. 3D). Notably, among these clustered monoisotopic peaks of glycopeptides, 532 (37%) were corrected by correction functions, which demonstrates their utility in improving the efficiency of glycopeptide detection. The average number of members in each cluster was 19.1. Among the detected monoisotopic signals, 43% (2309 signals) were assigned to glycopeptide clusters, which was reasonable considering the analysis of glycopeptides enriched by HILIC and the pretreatment of input MS1 data for noise cutting in the external deconvolution procedure.

**Figure 4:**
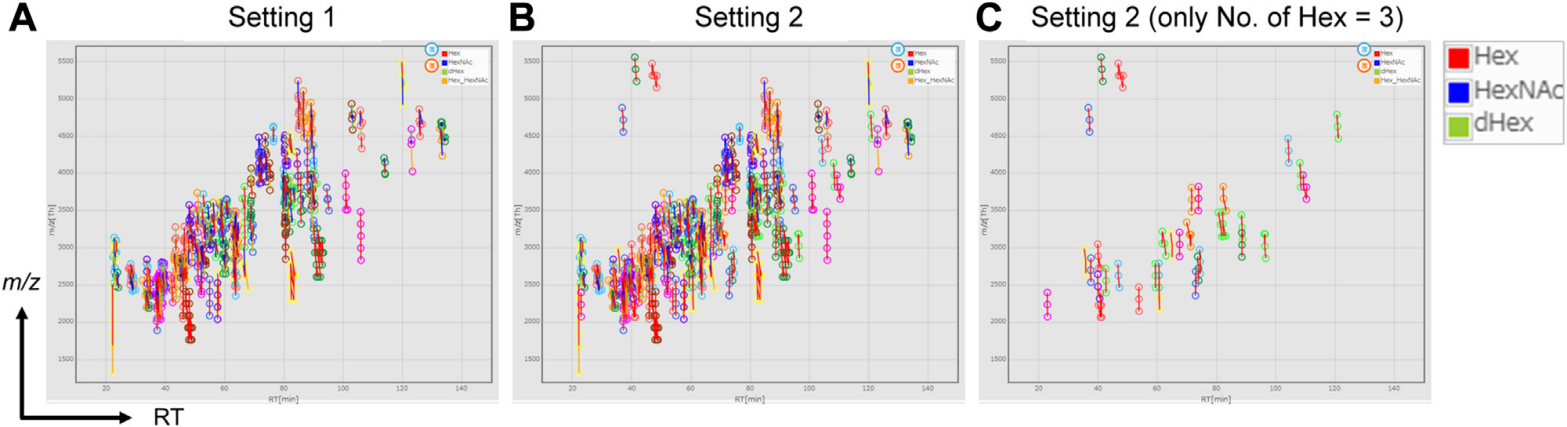
**Clustering view of HL-60 cell lysate samples.** All clusters for HL-60 cell lysates (HILIC Fr.2) shown in Data S4 can be visualized in the viewer of the GRable main window. Parallel clustering was performed under two search parameter settings: (*A*) setting 1 (a setting of the prototype) and (*B*) setting 2 (a setting for detecting oligo-mannosylated glycopeptides). The detailed settings are as follows: for setting 1, neutral saccharides (Hex, HexNAc, dHex, Hex + HexNAc): -1–0 (min), and minimum number of members: 4; for setting 2, neutral saccharide (Hex only): -1–0 (min), and minimum number of members: 3. Among the detected clusters in setting 2, only clusters with three members having oligo-mannosylated glycans are presented (*C*).

To evaluate the effectiveness of the parallel clustering function for detecting glycopeptides with a specific glycan structure, such as oligo-mannosylated glycopeptides, we used a dataset of HL-60 cell lysates (HILIC fraction 2) as a complex mixture of unknown proteins containing glycopeptides decorated with oligo-mannose (32). Because the sample glycopeptides used were desialylated, parallel clustering was performed under two search parameter settings: setting 1 (previous setting) and setting 3 (oligo-mannose glycans) for the hAGP sample. Figure 4A shows the clusters detected in setting 1, in which the minimum number of clusters was set to four. When setting 2 (only Hex was considered a glycan unit and the minimum number of clusters was three) was added to the search for setting 1, the number of detected clusters increased (Fig. 4B). For clarity, only the newly detected clusters with No. of members: 3 were visualized (Fig. 4C), demonstrating the presence of oligo-mannosylated glycopeptides. Thus, GRable can be applied to large-scale and in-depth analyses of glycoproteins, in which the comprehensiveness of glycan heterogeneity is improved, and glycan structure-specific search is allowed.

### Matching with Improved Evaluation of the Analysis Using Inter-Clustering Analysis Function

In this step, GRable comprehensively searches all combinations of core peptide and glycan compositions for each glycopeptide cluster by consistency in their masses, based on the lists of possible peptide and glycan compositions provided by users. Accordingly, the results sheet provides information regarding the three matched components (glycopeptides, peptides, and glycans) and the differences from the calculated mass values for each cluster. Note that this step considers matching by mass alone; thus, all matched results that meet the criteria can be provided for a single cluster. In the subsequent step, the most plausible match was selected from among the matching candidates for each cluster, and the accuracy of the selected match was evaluated. The likelihood of the assigned glycan composition was crucial for this selection. Accordingly, positive points for likely compositions (and negative points for unusual compositions as an option), which can be defined in the glycan composition list, were assigned to the glycan compositions that matched with the matching result list, and the total score was calculated for each cluster as the sum of points of all members.

Regarding this step, the previous Glyco-RIDGE analysis was limited by a low match rate. Mass calculations assume that peptides are ionized with protons such as MH^+^. Some clusters with no appropriate match were non-proton adduct clusters. Accordingly, GRable was equipped with an inter-cluster analysis function in the Matching step to detect clusters comprising glycopeptides ionized with nonproton cations such as iron (Fe^3+^) and ammonium (NH^4+^). In this inter-cluster analysis, the presence of such adducts was judged for each unit of the glycopeptide cluster and was unlikely to include unintended misdetection caused by isobaric combinations (e.g., NeuAc + NH_4_^+^ and Hex + dHex (24)). In the matching results for hAGP, using lists of 60 core peptides (Supplemental Table S1) and 134 glycan compositions (Supplemental Table S2), 65 clusters were identified. Among them, 46 clusters were matched to peptides and glycans, while 19 were not (Data S2). Over half of the clusters (i.e., 44 clusters) were related to the corresponding iron or ammonium adducts with the inter-cluster analysis function, as shown in the "relation between clusters" sheet (Data S2). This information facilitates technical evaluations because nonproton adducts cause loss of sensitivity; thus, reducing these adducts during sample preparation and analysis is essential. The remaining unmatched clusters may be attributed to the lack of core peptides in the list; therefore, the inter-cluster analysis function provides clues as to how the list should be improved.

### Selection of Plausible Assignments with Improved Criteria

This last step is crucial because selecting the most plausible match is essential for guaranteeing the certainty of the Glyco-RIDGE analysis results, considering that the false discovery rate cannot be defined. Because this selection step was conducted with manual inspections in our previous procedure, using this step with clear criteria in GRable represents a large technical improvement. As previously described, a crucial criterion is the total score of each matched cluster. Accordingly, this step sets the total score threshold, and only matches that are within the threshold are shown in the selection result sheet (Data S2). When the Clustering and Matching steps are correctly performed, all members for one cluster are matched with any glycan composition with the same carrier in the core peptide list (Supplemental Table S1), and many points are obtained based on the glycan point list (Supplemental Table S2). In addition to the total score, additional criteria were the delta mass and delta RT between the glycopeptide and the corresponding core peptide, albeit unused for automated selection by GRable. The usefulness of relative RT information has also been demonstrated in MS2-based *N*-glycoproteomics (24). Thus, all members within these criteria are highlighted in the results sheet, visually confirming the certainty of the results. Notably, identical results were obtained for hAGP analysis, even without RTs of the core peptides (i.e., RT = 0 for all peptides in the core peptide list), suggesting that it is possible to use predicted peptide lists when an analyte is a purified glycoprotein.

Using the exported file after the selection step (Data S2), the final assignment list for hAGP (Supplemental Table S7) was generated as follows. First, matches with delta mass and delta RT being colored (i.e., within the threshold) were selected. For cluster 1, there were two candidate matches with core peptides (hAGP isoform 1 [A1AG1] and haptoglobin (HPT)), where the total scores were 28 and 11, respectively. The delta RTs of all members for HPT are over 15 min, whereas the delta RT of A1AG1 members is within −6.5 to 5.1 min. Accordingly, A1AG1 was selected as the core peptide for cluster 1 due to meeting the defined criteria. In cases where multiple candidate matches still exist for one cluster, the match with the higher total score is selected; for cluster 3, the match with total score of 21 was selected over the one with a total score of 4. If there are multiple candidates with the same total score for one cluster (e.g., cluster 65), these matches are excluded. Next, each remaining match was checked to ensure that the ratio of members with glycan points for each cluster is >50%; matches with lower percentages of cluster members were excluded from the final assignments. By applying these criteria, users can easily choose the most plausible matches for all matched clusters to obtain the final assignment of GRable analysis.

### Improved Evaluation of the Assignments Using MS2 Information

As the other important improved point, this selection step allows the evaluation of the "confidence level" of the selected results using MS2 information. This scrutiny and curation step to reduce misassignment and ambiguity is critical in current *N*-glycoproteomics (37). Therefore, as with many MS2-based glycopeptide identification methods (12), GRable first searches glycan fragment ions, which are called "diagnostic ions," in MS2 spectra for cluster members to evaluate whether the spectrum is attributed to a glycopeptide. Diagnostic ions, such as fragments of HexNAc and HexNAc + Hex, were searched as defaults. The variation in the diagnostic ions can be set without restricting the number of ions. These diagnostic ions are not used for selection and are only added to the list of selection results, allowing users to utilize this information for different purposes. In addition, glycopeptide fragments such as Y0, Y1, and Y2 were searched to estimate the mass of Y0, namely, the peptide moiety. Because the Y0 signals are often too weak to be detected, GRable is equipped with a function for predicting the Y0 mass based on the masses of other related signals if multiple signals are present. A matched peptide was considered correct if the mass value coincided with that of the matched peptide. This MS2 information is summarized for the selected clusters in the "MS2 info for Clusters" sheet, and all search results are available in the "All MS2 info" sheet of an exported file (Data S2). The presence of such MS2 information is visible in the "Selection results" sheet and accessible directly via an intra-file link for all glycopeptides in the sheet.

For hAGP, all assigned glycopeptides showed MS2 spectra with HexNAc(204) as diagnostic ions characteristic of glycopeptides, along with glycopeptide-derived ions, supporting the certainty of assignment (Supplemental Table S7). In addition, 45 of 78 monoisotopic peaks in cluster 1 were accompanied by MS2 information, and the predicted peptide sequence was an A1AG1-derived peptide (WFYIASAFRNEEYNK). Therefore, the selection result for this cluster was confirmed using MS2 information.

In this context, the Glyco-RIDGE results were presented with the "confidence level" using GRable. The probability/confidence of the assignment for core peptides is ranked as follows: 1) "High"; clusters having any member(s) showing Y0-related ions corresponding to the presumed core, 2) "High"; clusters having any member(s) identified as the same core using the MS2-based search engine, 3) "Medium"; clusters having any member(s) showing glycan diagnostic ions, 4) "Low"; clusters without any MS2 support. We believe that the confidence of the assignment is high even in the "Low" rank when their mass and RT differences fit under the threshold and the glycan point is the highest in the cluster. This was supported when the assigned core peptide for a low-rank cluster was found in the core peptide list based on confident deglycopeptide identification for the identical samples with 1% FDR. Originally, GRable was designed to estimate the glycopeptide signals that could not be identified by the MS2 spectra; thus, cluster members without MS2 support and clusters with no MS2 data for all members, which can be confirmed in a following targeted experiment, are notable features of GRable. In addition, the glycopeptide detection methodology of GRable allows the evaluation of the certainty of MS2 information-lacking assignments based on their connected members in the same cluster. In this evaluation, visualization of the two-dimensional (i.e., RT and mass) relationship of each cluster member in the cluster sheet allows manual inspection of whether an unexpected glycan composition is likely; with this manual evaluation, unconvincing assignments can be excluded from the final assignments.

Notably, oxidized Met in the deglycopeptide search for improving coverage can result in accidental mismatching for one glycopeptide; for example, oxidation of the peptide and HexNAc, and Hex and carbamidomethylation of the peptide (23). However, in the Glyco-RIDGE method, such misassignments are eliminated in the selection process, where the core peptide of each glycopeptide is evaluated as a glycopeptide cluster. Because peptides with the same sequence but different modifications are handled as "different" core peptides, only one core peptide can be assigned per cluster. Consequently, incidentally matched glycopeptides can be detected and excluded. Similarly, carbamidomethylation of the Met within a core peptide, not accounted for in the peptide list preparation and posing a risk of mismatches (23), will be excluded from the selection results based on its association with other members of the same cluster.

### Improved In-Depth Glycoproteomics Results Using GRable

**Figure 5:**
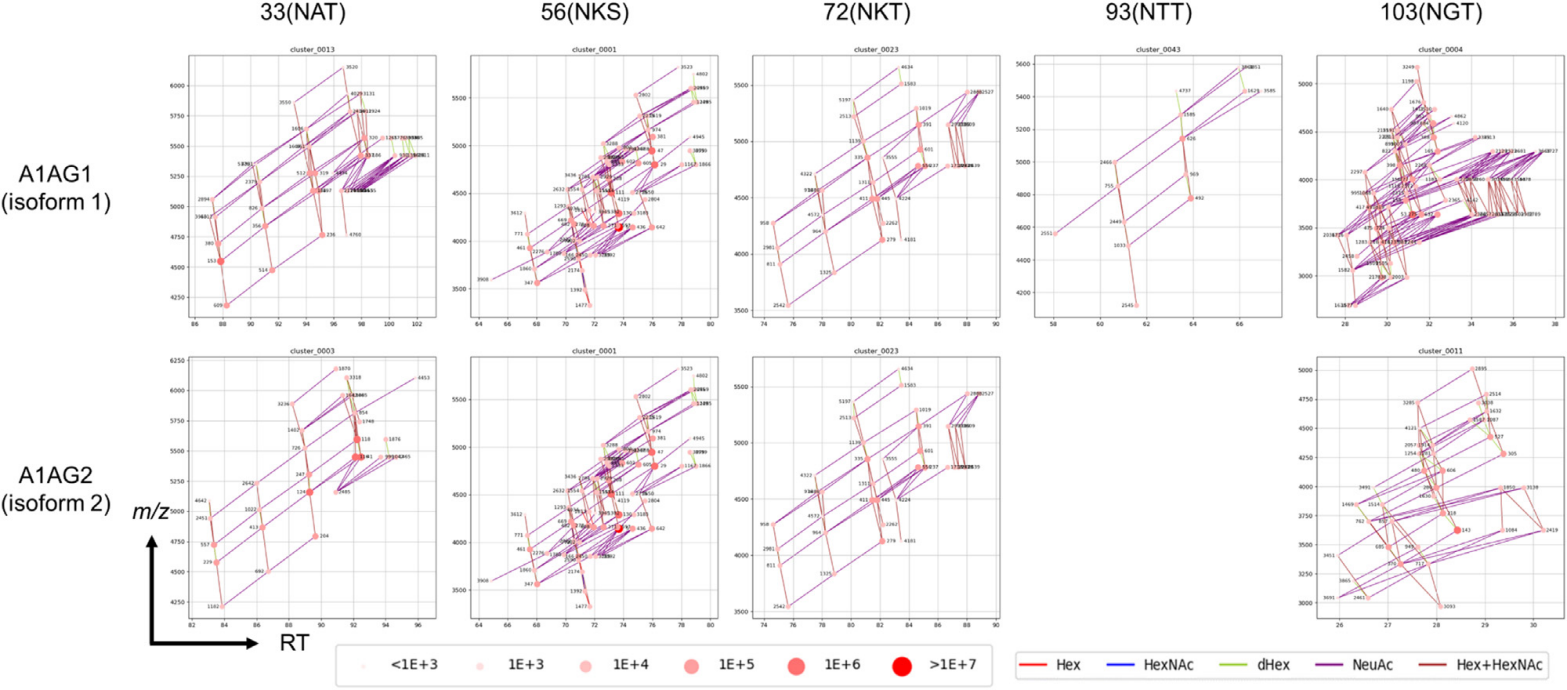
**Glycopeptide clusters assigned for five *N*-glycosylation sites of hAGP isoforms.** Corresponding core peptide sequences for each cluster are indicated in Table 1. Since the core peptide of two isoforms is identical for sites 56 and 72, the same clusters are indicated for both isoforms.

Two isoforms of hAGP exist, isoform 1 (A1AG1) and isoform 2 (A1AG2), of which A1AG1 is the major component. Based on the improved GRable procedure, five glycoproteins, including HPT, plasma protease C1 inhibitor, and lymphatic vessel endothelial hyaluronic acid receptor 1 as serum-derived contaminants, were assigned to the hAGP samples (Supplemental Table S7). Among the 14 assigned clusters, seven (1, 4, 13, 23, 26, 43, and 48) and six (1, 3, 6, 11, 19, and 23) were assigned as A1AG1 and A1AG2, respectively. These clusters covered all five potential *N*-glycosylation sites of A1AG1 and A1AG2 (the two sites are common in A1AG1), except for site 93 of A1AG2; *N*-glycosylation impairment at this site is consistent with a previous report (44). The estimated core peptides for each cluster were the same as those estimated using the MS2 information utilization function of GRable and Byonic, increasing the confidence of the results. Each cluster strictly corresponded to one core peptide; thus, the clustering view facilitated effective glycan macro-heterogeneity visualization for each glycosite (Fig. 5). Notably, two *N*-glycosites (sites 33 and 103) were detected in almost the same core peptide, except for one residue between the two isoforms; however, their site-specific glycoforms could be separately visualized. This result highlights the usefulness of GRable for elucidating the differences in the glycosylation status of target glycoproteins, even with other contaminated glycoproteins.

Using GRable, 123 and 108 site-specific glycoforms of A1AG1 and A1AG2 were estimated, respectively (Tables 1 and S8). Certain *N*-glycosites showed the same glycan compositions across multiple core peptides, reinforcing result accuracy. For instance, at site 103 of A1AG1, 24 of 29 compositions assigned for ENGTISR were also detected for the other two peptide sequences. hAGP had *N*-glycosylations with highly sialylated and fucosylated branches and extended with polylactosamine (43, 44). Similar results were obtained with GRable (Supplemental Table S4); tetrasialo-glycans were estimated at all five A1AG1 sites, indicating high sialylation. Glycans with dHex ≥2 were estimated at four A1AG1 sites, indicating fucosylation at both its core and branches. In addition, glycan components for polylactosamine (i.e., Hex + HexNAc ≥10) were estimated at four sites, indicating the presence of such extended glycans.

**Table 1:** Site-specific hAGP glycoforms estimated using GRable and Byonic

Site	Sequence^b^	No. of site-specific glycoforms^a^			
		A1AG1		A1AG2	
		GRable	Byonic	GRable	Byonic
33 (NAT)	A1AG1: QIPLCANLVPVPIT**N**ATLDQITGK	21 (14)	7 (0)	23 (13)	10 (0)
	A1AG2∗: QIPLCANLVPVPIT**N**ATLDRITGK				
56 (NKS)	WFYIASAFRNEEY**N**K	35 (31)	6 (2)	35 (31)	6 (2)
72 (NKT)	SVQEIQATFFYFTP**N**K	24 (18)	6 (0)	24 (18)	6 (0)
93 (NTT)	A1AG1: QDQCIY**N**TTYLNVQR	13 (11)	2 (0)	0 (0)	0 (0)
	A1AG2: QNQCFY**N**SSYLNVQR				
103 (NGT)	A1AG1∗: E**N**GTISR	29 (23)	6 (0)	25 (21)	4 (0)
	A1AG2∗: E**N**GTVSR				
		122 (97)	27 (2)	107 (83)	26 (2)

a Numbers within parentheses indicate the number of glycoforms estimated using only GRable or Byonic.

b *N*-glycosylation sites are indicated in *bold*. Different residues in the two isoforms are *underlined*. The asterisks indicate the presence of other peptide sequences of different lengths.

### Evaluation of the Certainty of Glycoproteomic Results Using GRable

To evaluate the suitability of the search parameter settings used here for accurate glycopeptide detection, we performed a GRable analysis of the IGOT-LC/MS data (i.e., *N*-deglycoproteomic data) used for preparing the core peptide list using the same search parameter settings as those for the *N*-glycoproteomic data. As expected, the detected clusters for *N*-deglycoproteomic data significantly decreased for both hAGP (from 65 to 4 clusters) and HL-60 (from 207 to 32 clusters). Most selected glycopeptides were also observed in the corresponding *N*-glycoproteomic data, and the few remaining ones (only one each for hAGP and HL-60) was considered possible based on their glycan compositions (Supplemental Table S9, and S10), confirming the absence of unintended misassignment. These results indicate that the settings used for GRable analysis were appropriate. Notably, GRable is intended to assign glycopeptides with single glycosite. When there are still unremovable glycopeptides after PNGase F treatment and the glycan components are unlikely for *N*-glycopeptides, *O*-glycosylation search of IGOT-treated *N*-deglycopeptides may be useful; this additional search is expected to evaluate the presence of *O*-glycosylation and determine the sequence of *O*-glycosylated peptide, assessing the possibility of misinterpretation of the GRable results of site-specific *N*-glycoform analysis.

To further evaluate the adequacy of GRable settings and the reliability of the results, sialidase-treated hAGP was also analyzed similarly to intact hAGP, and their GRable results were compared. We performed additional LC-MS/MS analysis of hAGP after sialidase treatment. When specific clusters of the same core peptides were compared (Supplemental Fig. S3), the signal intensities of glycopeptides with glycans without NeuAc were higher in Sialidase(+) compared to those in Sialidase(−), as expected. Additionally, further extended glycans were newly detected. A total of 177 site-specific glycoforms were assigned for sialidase(+) hAGP, and 94 of them were not found for sialidase(−) hAGP. These sialidase(+)-specific glycopeptides had relatively higher *m/z*, making them potentially undetectable without sialidase treatment (Fig. 3D). Among the 94 glycopeptides found exclusively in sialidase(+) hAGP, 26 glycopeptides had glycans containing dHex ≥2. With the correction function, we could confirm that monoisotopic signals were correctly assigned, reducing the likelihood of misdetection of dHex(2) and NeuAc(1). Consequently, such glycopeptides had glycans containing dHex ≥2, which could be detected due to reduced glycan heterogeneity and increased signal intensity. These results support the reliability of the GRable analysis methodology presented here.

Next, to evaluate whether the presence of other (glyco)peptides would affect the detection of the glycopeptide of interest, we also performed an in-silico spike-in test. In this test, HeLa cell lysate, which was used as a model of a complex mixture containing glycopeptides, was analyzed by LC/MS/MS in a similar condition as hAGP and the monoisotopic peak list was prepared using GRable (Supplemental Table S11) and then merged with the list for hAGP alone (Data S2). In GRable analysis using the merged list, the detected glycopeptide clusters (Supplemental Fig. S2) were completely identical to the clusters for hAGP alone (Fig. 3D). In addition, the selection results were identical to the results for hAGP alone (Data S2), confirming no false positives. These results support the reliability of the glycopeptide detection methodology using GRable, especially for the analysis of complex mixtures.

In the hAGP analysis, the confidence level of all assigned clusters was high or medium, indicating that at least one member had MS2 information for every glycopeptide cluster. To validate the certainty of the obtained results for hAGP analysis, 110 MS2 spectra of all assigned glycopeptides with MS2 information were manually checked (Supplemental Fig. S4), confirming that the core peptides were correctly assigned. Notably, in MS2 spectra for glycopeptides containing site 33, some y ions derived from glycopeptides with HexNAc(1) and HexNAc(2) were observed, confirming the presence of *N*-glycosylation. It was also notable that NeuAc-derived diagnostic ions were observed for several glycopeptides without NeuAc owing to insufficient isolation, highlighting the risk of evaluating the accuracy of glycoforms based only on diagnostic ions.

In addition, to evaluate the certainty and significance of the Glyco-RIDGE results obtained using GRable, the results were compared with those obtained using the representative MS2-based software Byonic. Notably, GRable could estimate more than four-fold more site-specific glycoforms than Byonic and covered most site-specific glycoforms estimated by Byonic. Only two of the site-specific glycoforms (Hex(2)HexNAc(1) and Hex(3)HexNAc(2)) were estimated by Byonic but not by GRable at site 56 because one component was absent (Hex(3)HexNAc(3) or Hex(3)HexNAc(2)NeuAc(1)). As expected, the MS2-based estimation by Byonic succeeded on higher-intensity glycopeptides (Data S5). Notably, such highly sialylated and polylactosamine-attached glycopeptides were not detected by Byonic (Supplemental Table S4), and the median number of NeuAc for 38 glycopeptides assigned by GRable but not by Byonic was 3, whereas the median number of 27 glycopeptides assigned by Byonic was 2. Accordingly, the MS2 spectra could have been too poor to assign a glycopeptide, partly due to their low ionization efficiency. Thus, these results confirm the reliability of the Glyco-RIDGE results obtained by GRable, highlighting the usefulness of this method as a complementary method to the current gold standard, MS2-based glycoproteomics.

## Conclusion

We developed a novel software program, GRable, to enable semi-automated Glyco-RIDGE analysis. GRable version 1.0 can run online freely with a demo or user data using a web browser via the GlyCosmos Portal (https://glycosmos.org/grable). Note that the current version is guaranteed only for Thermo Fisher Scientific data. Some algorithms of the existing Glyco-RIDGE method were improved during the implementation of GRable version 1.0, which can be applied for in-depth site-specific glycoform analysis of intact sialylated glycopeptides derived from purified and crude glycoproteins. Thus, this software will help analyze the status and changes in glycans to obtain biological and clinical insights into protein glycosylation. The novel parallel clustering function enabled a targeted search focusing on multiple glycan structural layers, including the stem, branching, and terminal moieties such as Lewis epitopes. This software also allows the evaluation of a "confidence level" especially using MS2 information, for each glycopeptide obtained by MS1-based detection methodology. Using the MS2 utilization function opened doors to all glycoproteomics researchers who considered MS2-based glycoproteomics as the gold standard, allowing for the community evaluation of GRable by comparing it with other glycoproteomics software. The combined use of MS1-based glycoproteomics will be useful for expanding the glycopeptide detection and providing supporting evidence for MS2-based glycoproteomics. Thus, the Glyco-RIDGE method with its unique MS1-based glycopeptide detection principle has complementary roles to MS2-based glycoproteomic methods, and GRable will be a powerful glycoproteomics tool when combined with the currently developed MS2-based software. Accordingly, GRable will be updated by continuously improving the Glyco-RIDGE methodology and software and by comparing it with the upcoming MS2-based methods and software.

## Data Availability

The MS data set for hAGP presented in this study has been deposited in the ProteomeXchange Consortium via the jPOST partner repository with the dataset identifiers PXD046226 (ProteomeXchange) and JPST001876 (jPOST).

## Supplemental data

This article contains supplemental data.

## Conflicts of interest

The authors declare that they have no conflicts of interest with the contents of this article.
