# Supplementary material for: GRable Version 1.0: A Software Tool for Site-Specific Glycoform Analysis With Improved MS1-Based Glycopeptide Detection With Parallel Clustering and Confidence Evaluation With MS2 Information
Source: Mol Cell Proteomics. 2024 Aug 23;23(9):100833. doi: 10.1016/j.mcpro.2024.100833 (PMC11421343; doi:10.1016/j.mcpro.2024.100833)
Supplement: Supplementary Fig S1-S4 [file mmc1.pdf]

Supplementary Materials

GRable version 1.0: A software tool for site-specific glycoform analysis using the improved Glyco-RIDGE method with parallel clustering and MS2 information

Chiaki Nagai-Okatani,[*,†,‡] *Daisuke Tominaga,*[†,‡] *Azusa Tomioka,*[†,‡] *Hiroaki Sakaue,*[‡] *Norio Goda,*[§] *Shigeru Ko,*[§] *Atsushi Kuno,*[‡] *Hiroyuki Kaji*[*,‡,¶]

[‡] Molecular and Cellular Glycoproteomics Research Group, Cellular and Molecular Biotechnology Research Institute, National Institute of Advanced Industrial Science and Technology (AIST), Tsukuba, Ibaraki 305-8565, Japan

[§] Department of Systems Medicine, Keio University School of Medicine, Shinjuku, Tokyo 160-8582, Japan

[¶] Institute for Glyco-core Research (iGCORE), Nagoya University, Furo-cho, Chikusa, Nagoya, Aichi 464-8601, Japan

[*] Correspondence

E-mail: chiaki-okatani@aist.go.jp (C.N.-O.); kaji-hiroyuki@igcore.nagoya-u.ac.jp (H.K.)

Table of Contents

Supplementary Figures (present in this file)

Figure S1. Glycan composition list for GRable and Byonic analyses of hAGP

Figure S2. Clustering views of HeLa cell lysate and HeLa + hAGP in in-silico spike-in analysis

Figure S3. Effects of sialidase treatment on hAGP analysis

Figure S4. MS2 spectra of glycopeptides assigned for hAGP

Supplementary Tables

Table S1. Core peptide list for GRable analysis of hAGP

Table S2. Glycan point list for GRable analysis of hAGP

Table S3. List of glycan compositions used for GRable and Byonic analyses of hAGP.

Table S4. Core peptide list for GRable analysis of HL-60 cell lysates

Table S5. Glycan point list for GRable analysis of HL-60 cell lysates

Table S6. Effects of changes in the minimum cluster member on hAGP analysis using GRable

Table S7. Assigned site-specific glycoforms for the hAGP sample using GRable

Table S8. Glycan compositions of each N-glycosite on hAGP estimated using GRable and Byonic

Table S9. Selection results for IGOT(+) hAGP sample using GRable

Table S10. Selection results for IGOT(+) HL-60 sample using GRable

Table S11. Monoisotopic peak list of HeLa cell lysate used for in-silico spike-in analysis

Supplementary Data

Data S1. Peak list of GRable hAGP analyses

Data S2. Output file of the GRable analysis for hAGP after selection

Data S3. Peak list for GRable analysis of HL-60 cell lysates

Data S4. Output file of the GRable analysis of HL-60 lysates after selection

Data S5. Output file of the Byonic analysis for hAGP

Supplementary Document

Document S1. Instruction manual for GRable version 1.0

Supplementary Figures

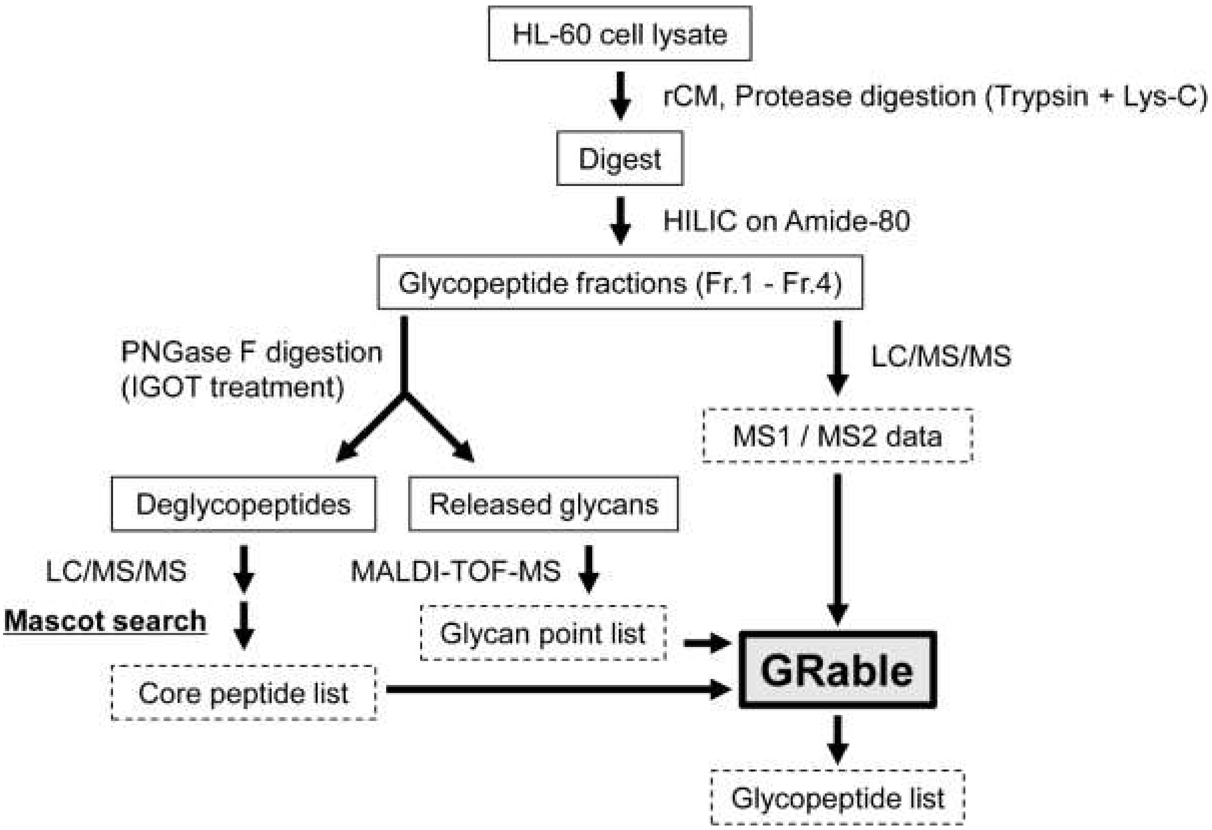

Figure S1. Workflow of glycopeptide analysis for HL-60 cell lysates using GRable

The details of the workflow are documented in the Experimental Procedure. rCM, reduction and carbamidomethylation; HILIC, hydrophilic interaction liquid chromatography; IGOT, isotope-coded glycosylation site-specific tagging; LC-MS/MS, liquid chromatography-tandem mass spectrometry; MALDI-TOF-MS, matrix-assisted laser desorption/ionization time-of-flight mass spectrometry.

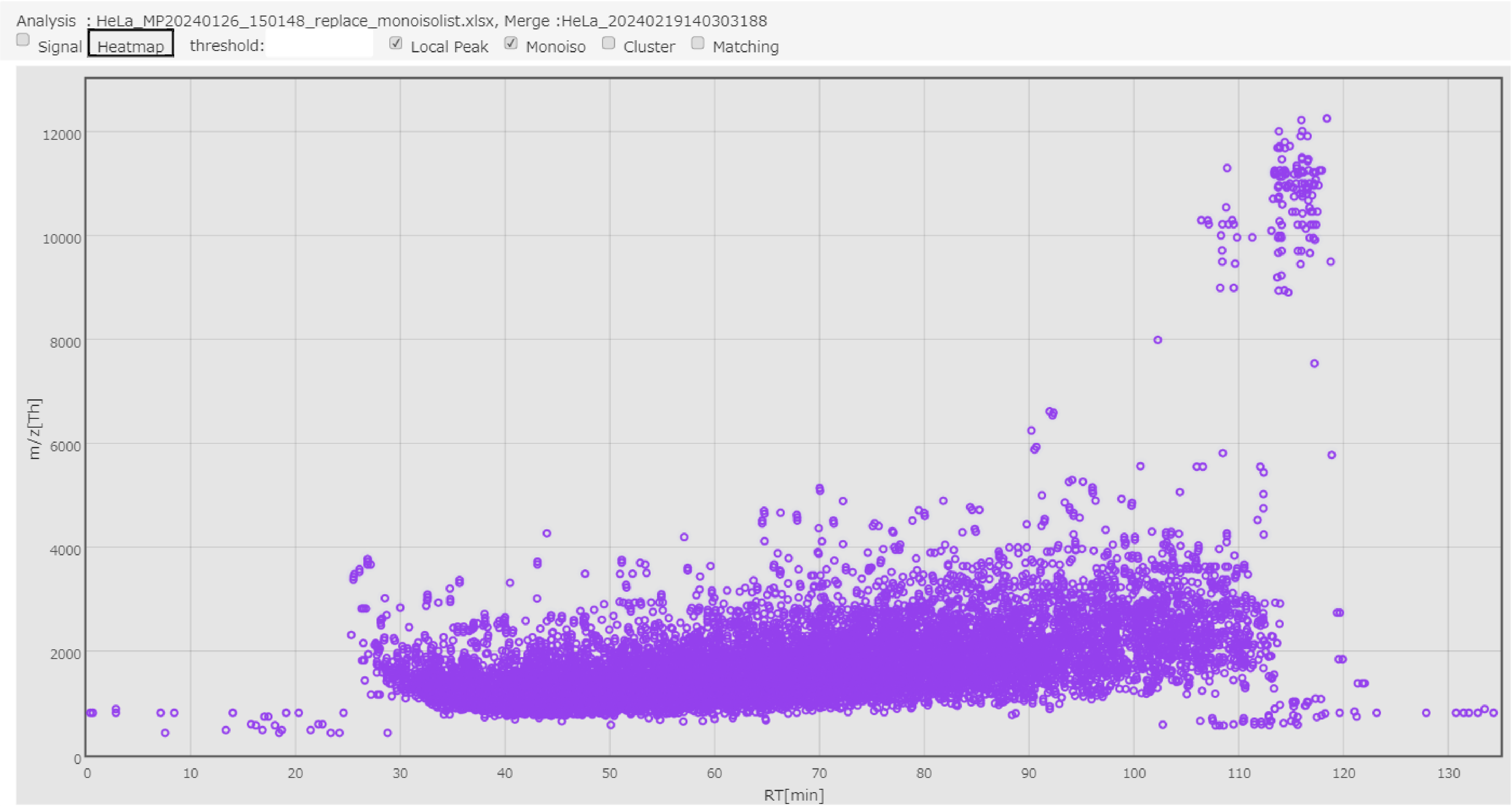

Figure S2-1. Clustering view of HeLa cell lysate in in-silico spike-in analysis

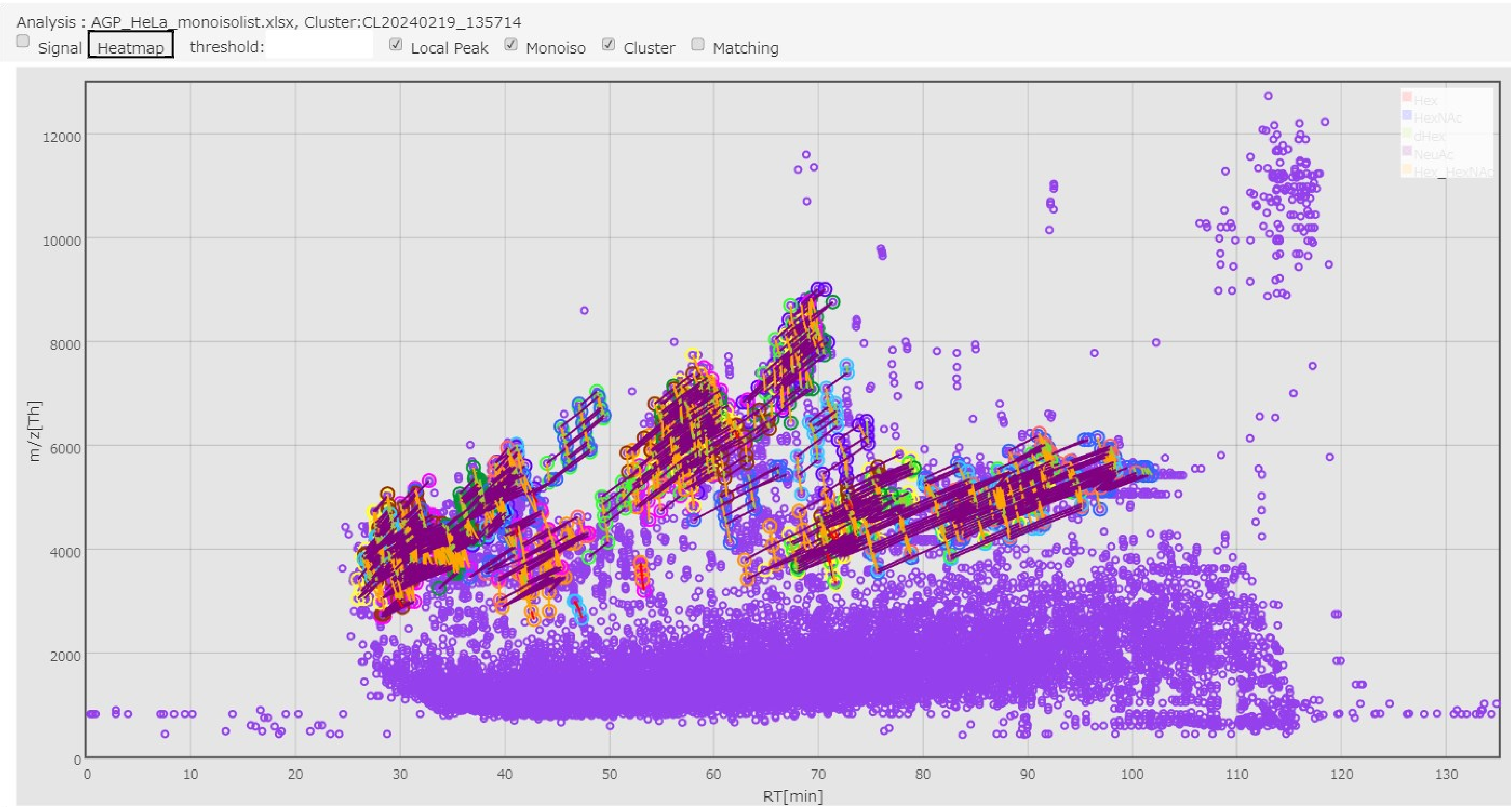

Figure S2-2. Clustering view of HeLa + hAGP in in-silico spike-in analysis

A1AG1/A1AG2_72(NKT)_SVQEIQATFFYFTPNK

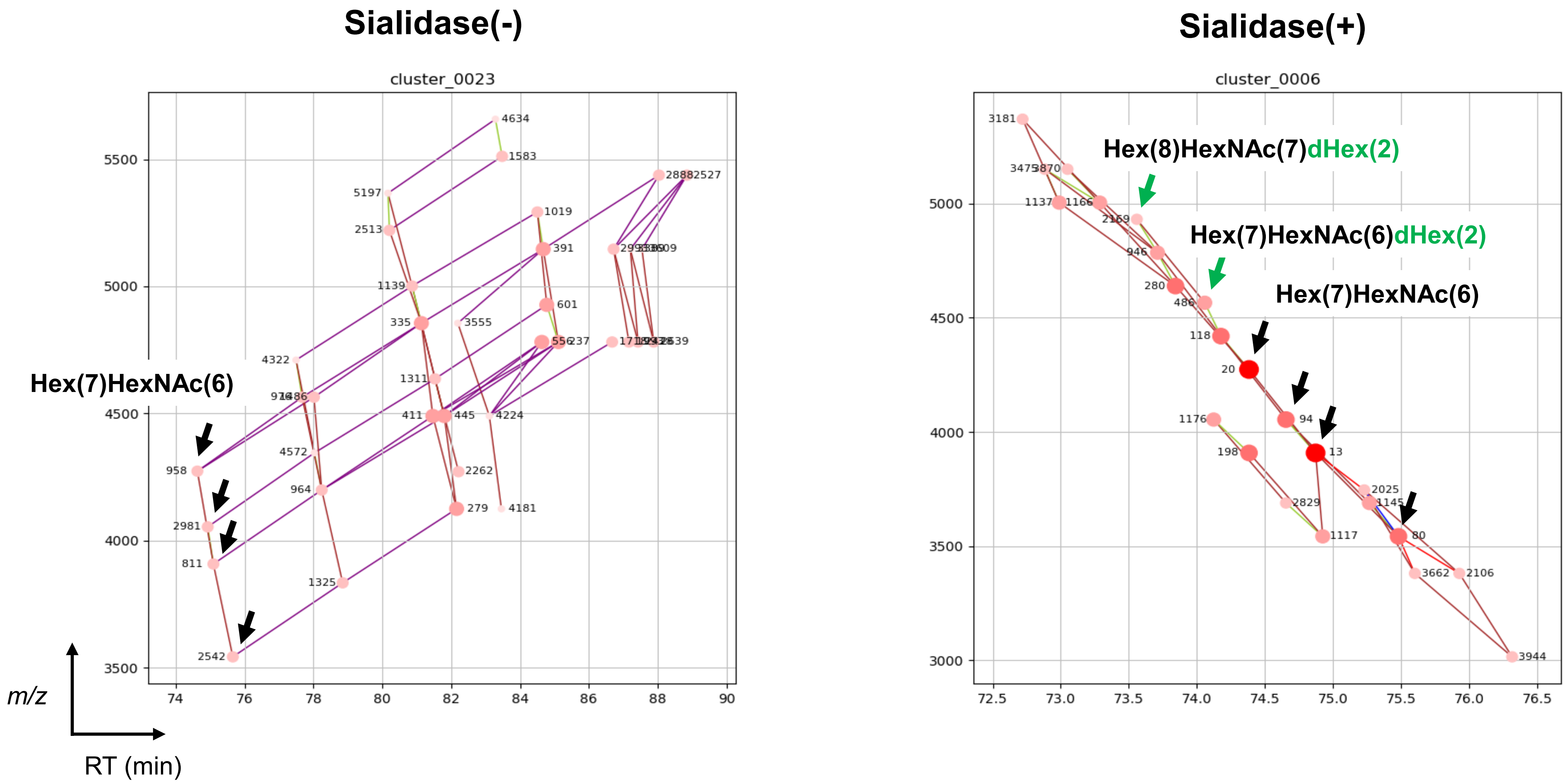

Figure S3-1. Effects of sialidase treatment on hAGP analysis. A representative cluster with the same core peptide for sialidase(-) and sialidase(+) are shown. When specific clusters of the same core peptides were compared, the signal intensities of glycopeptides with glycans without NeuAc (black arrows) were higher in sialidase(+) compared to sialidase(-). In addition, further extended glycans including Hex(8)HexNAc(7)dHex(2) and Hex(7)HexNAc(6)dHex(2) (green arrows) were newly detected.

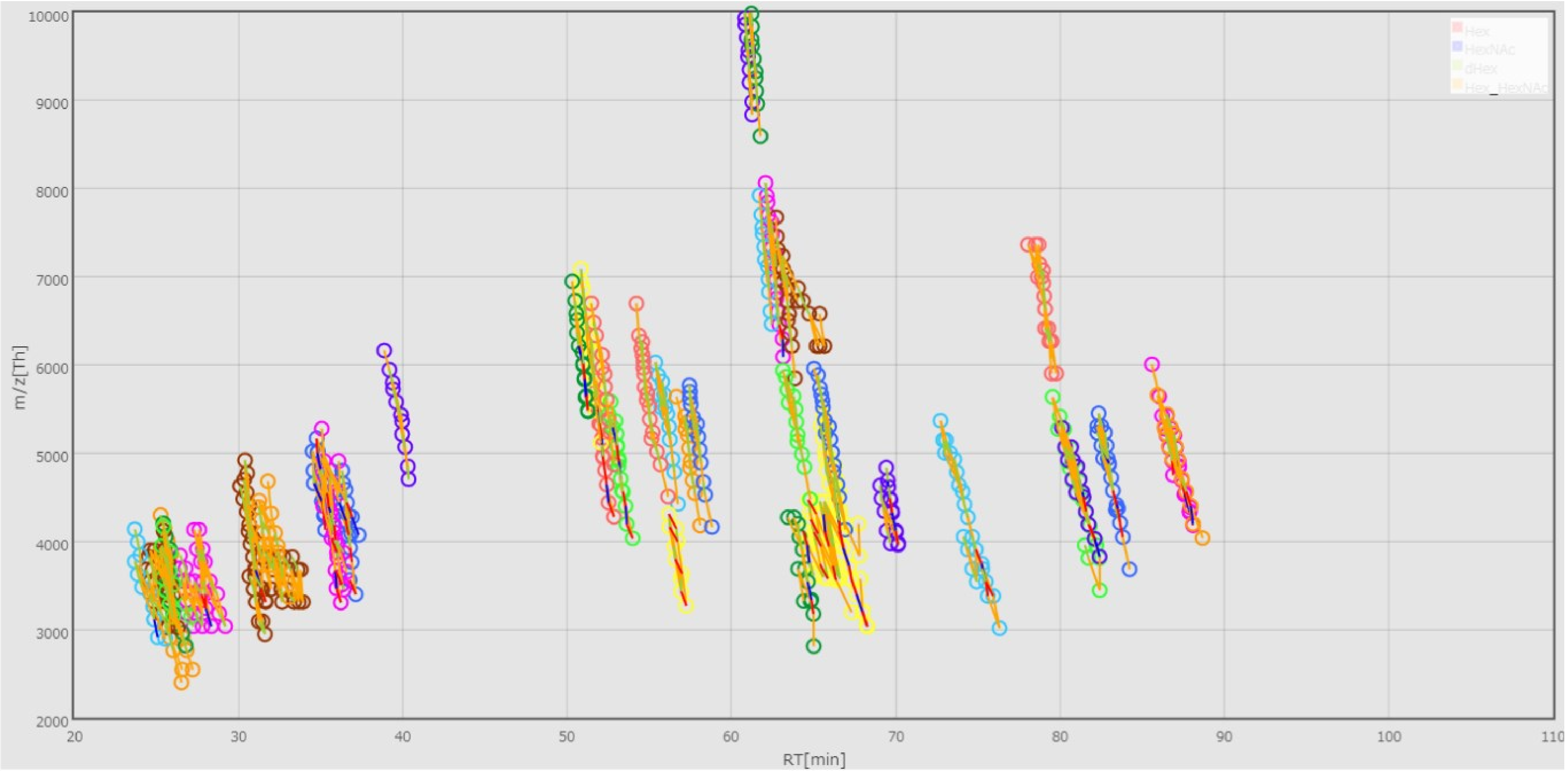

Figure S3-2. Effects of sialidase treatment on hAGP analysis. Clustering view of sialidase(+) for comparison with the view of sialidase(-) (Figure 3D) is shown.

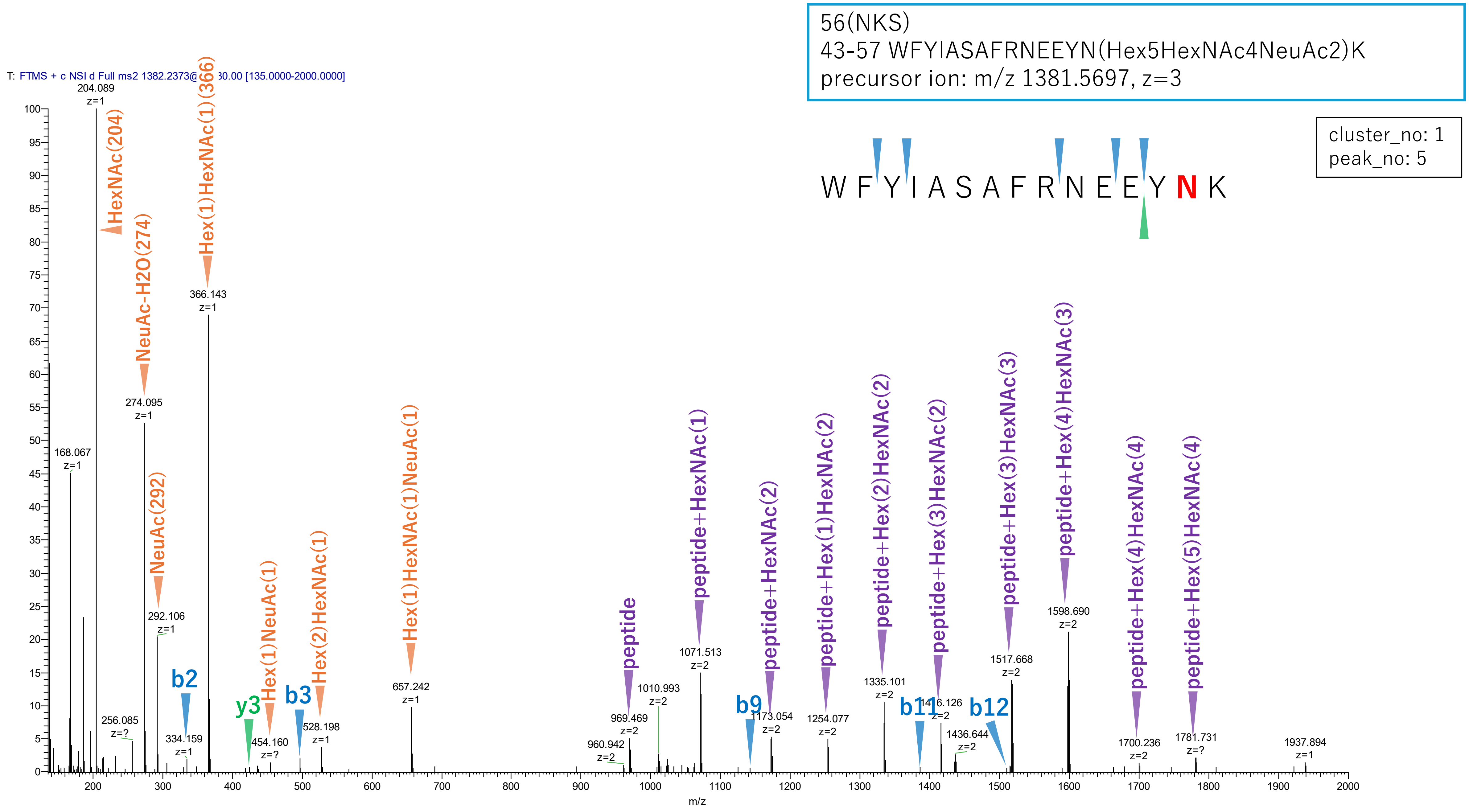

Figure S4-1. MS2 spectra of glycopeptides assigned for hAGP

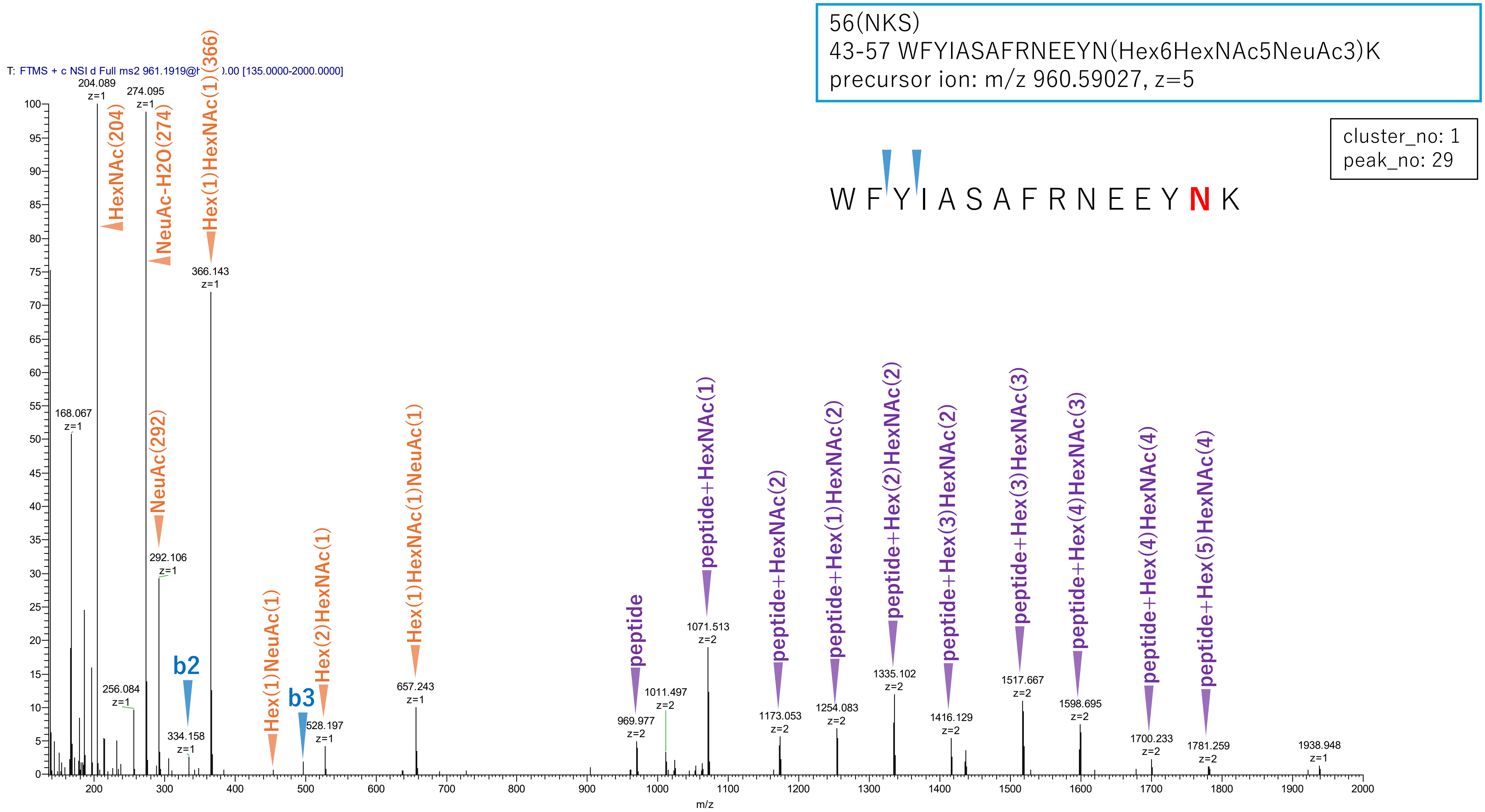

Figure S4-2. MS2 spectra of glycopeptides assigned for hAGP

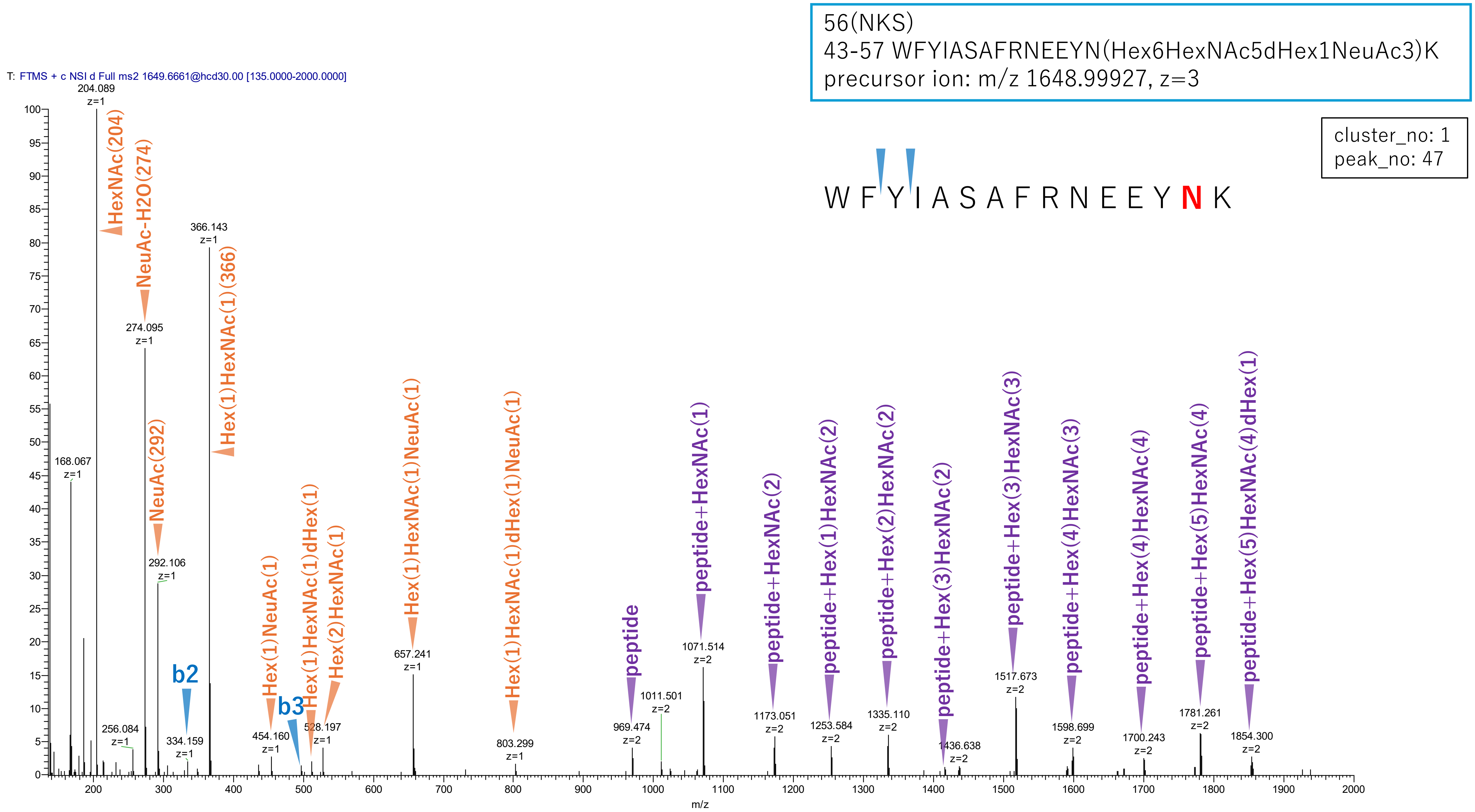

Figure S4-3. MS2 spectra of glycopeptides assigned for hAGP

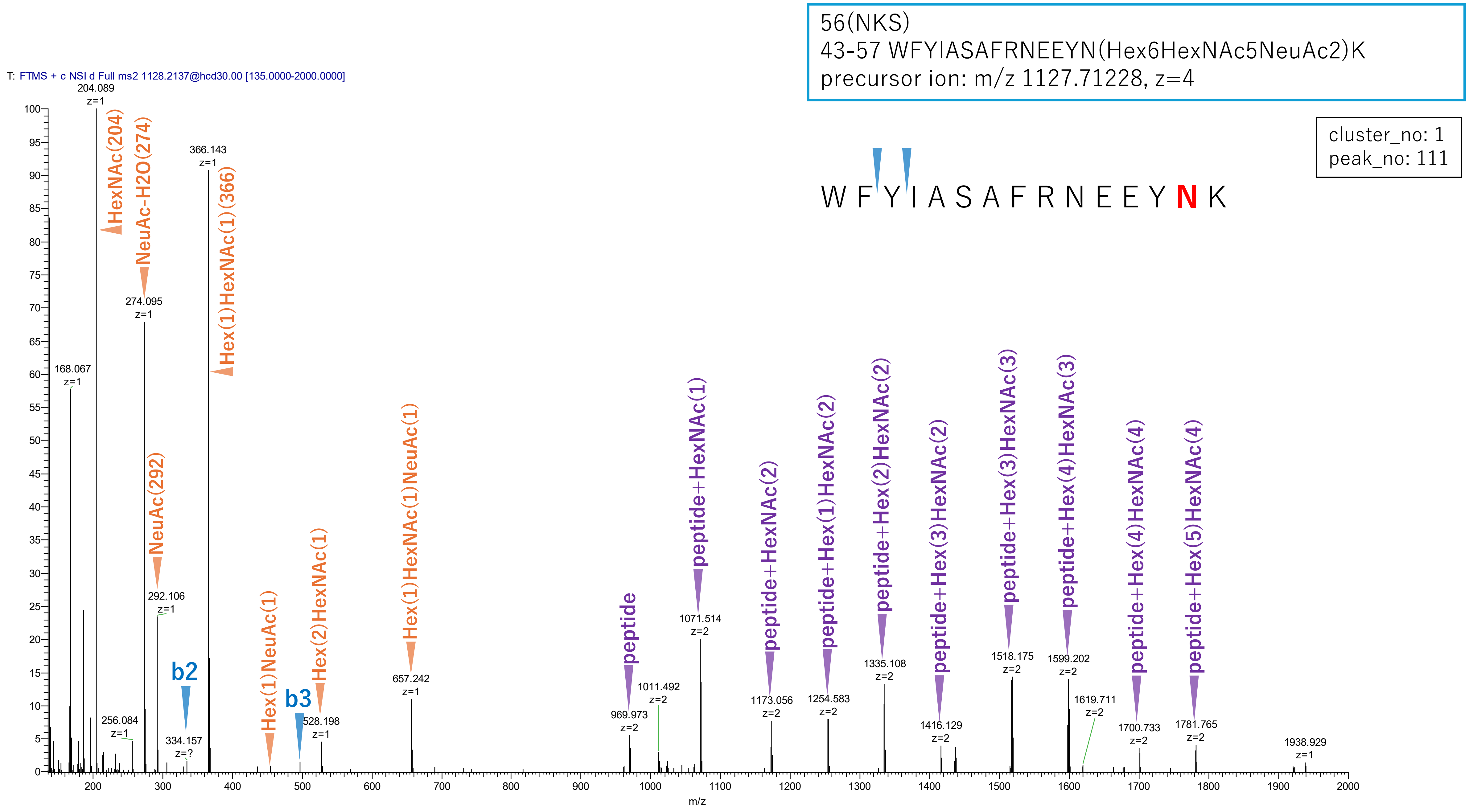

Figure S4-4. MS2 spectra of glycopeptides assigned for hAGP

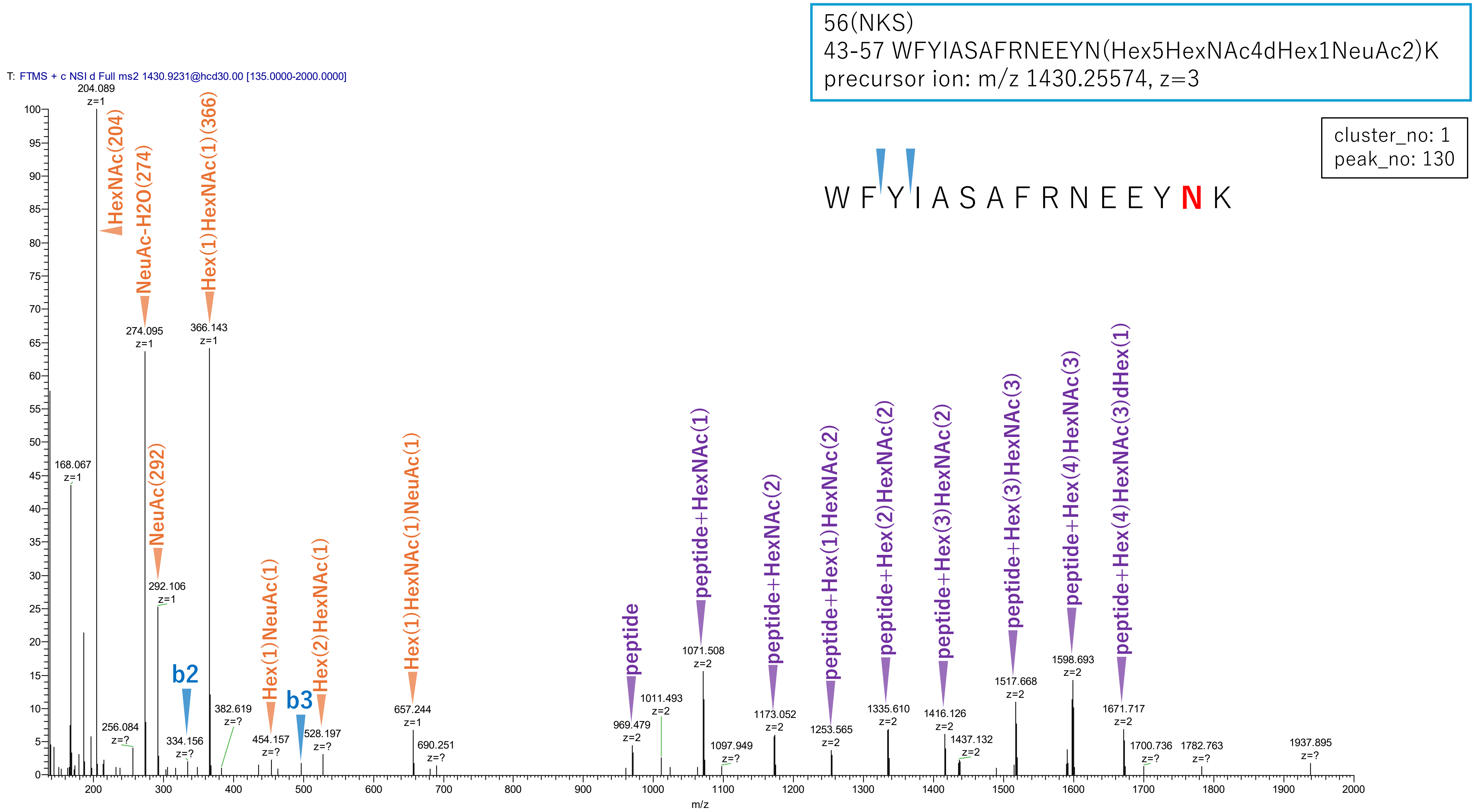

Figure S4-5. MS2 spectra of glycopeptides assigned for hAGP

56(NKS)
43-57 WFYIASAFRNEEYN(Hex5HexNAc4NeuAc1)K
precursor ion: m/z 1284.53784, z=3

cluster_no: 1
peak_no: 166

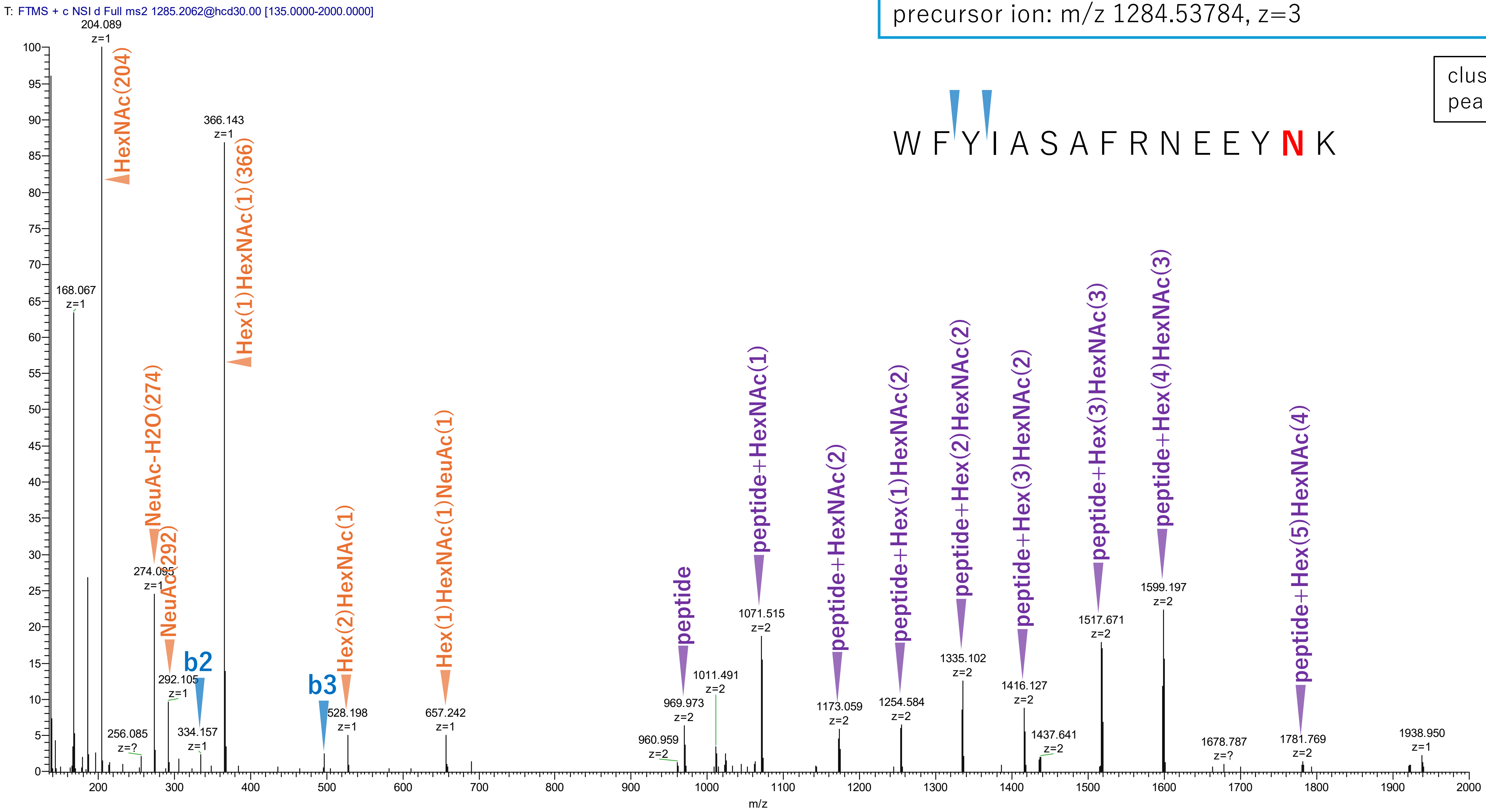

Figure S4-6. MS2 spectra of glycopeptides assigned for hAGP

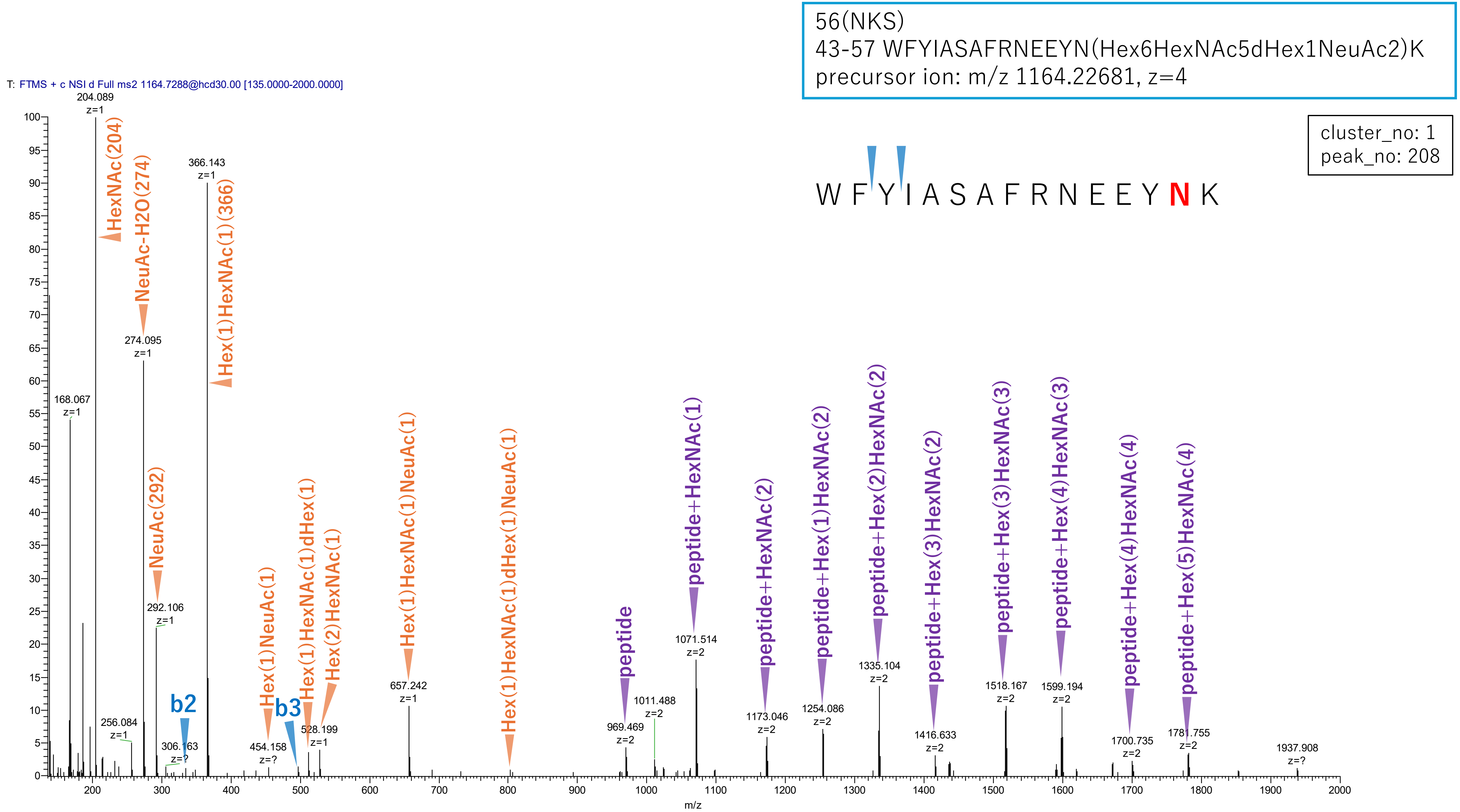

Figure S4-7. MS2 spectra of glycopeptides assigned for hAGP

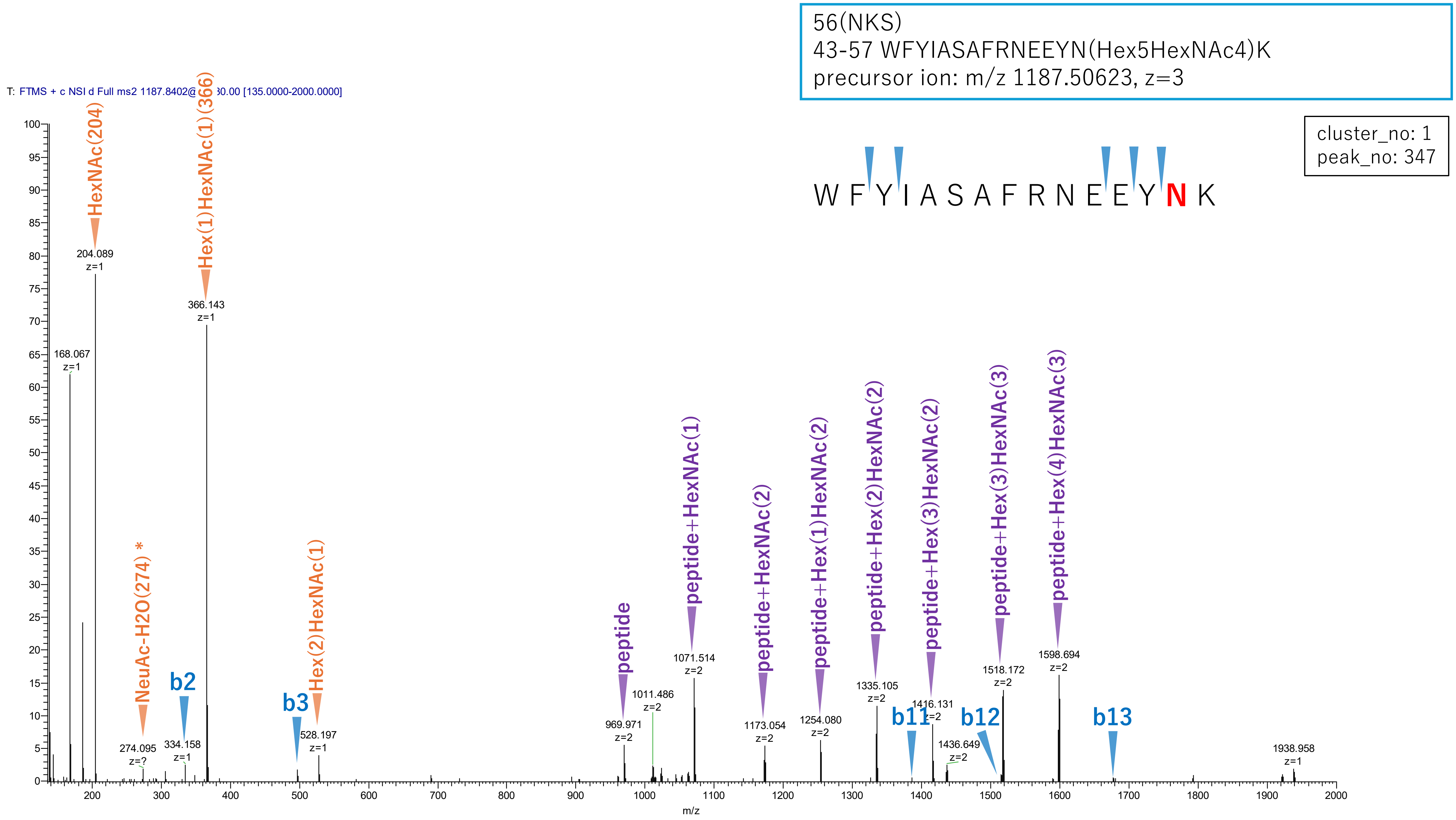

Figure S4-8. MS2 spectra of glycopeptides assigned for hAGP.

*Asterisks indicate diagnostic ions of NeuAc derived from a contaminated glycopeptide.

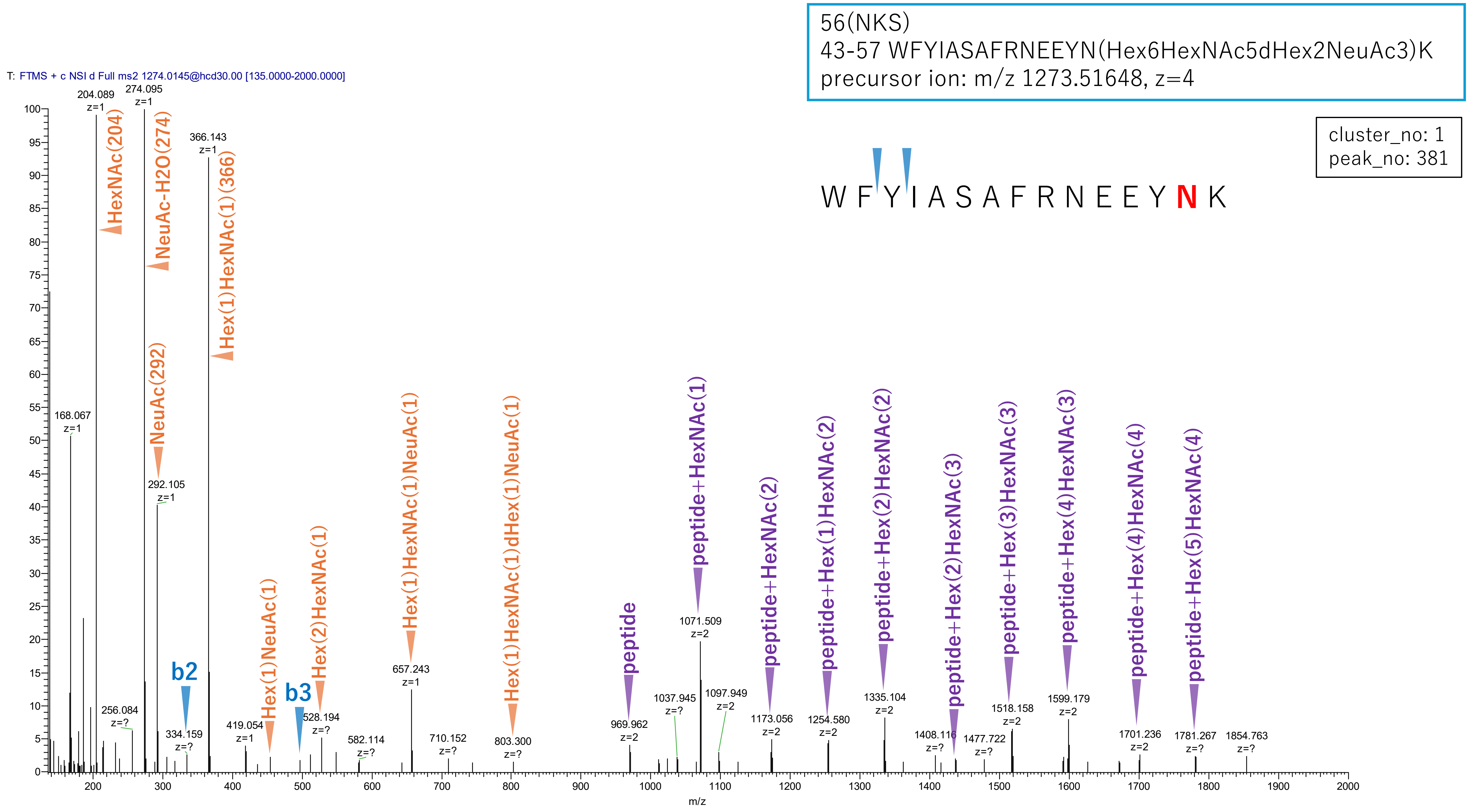

Figure S4-9. MS2 spectra of glycopeptides assigned for hAGP

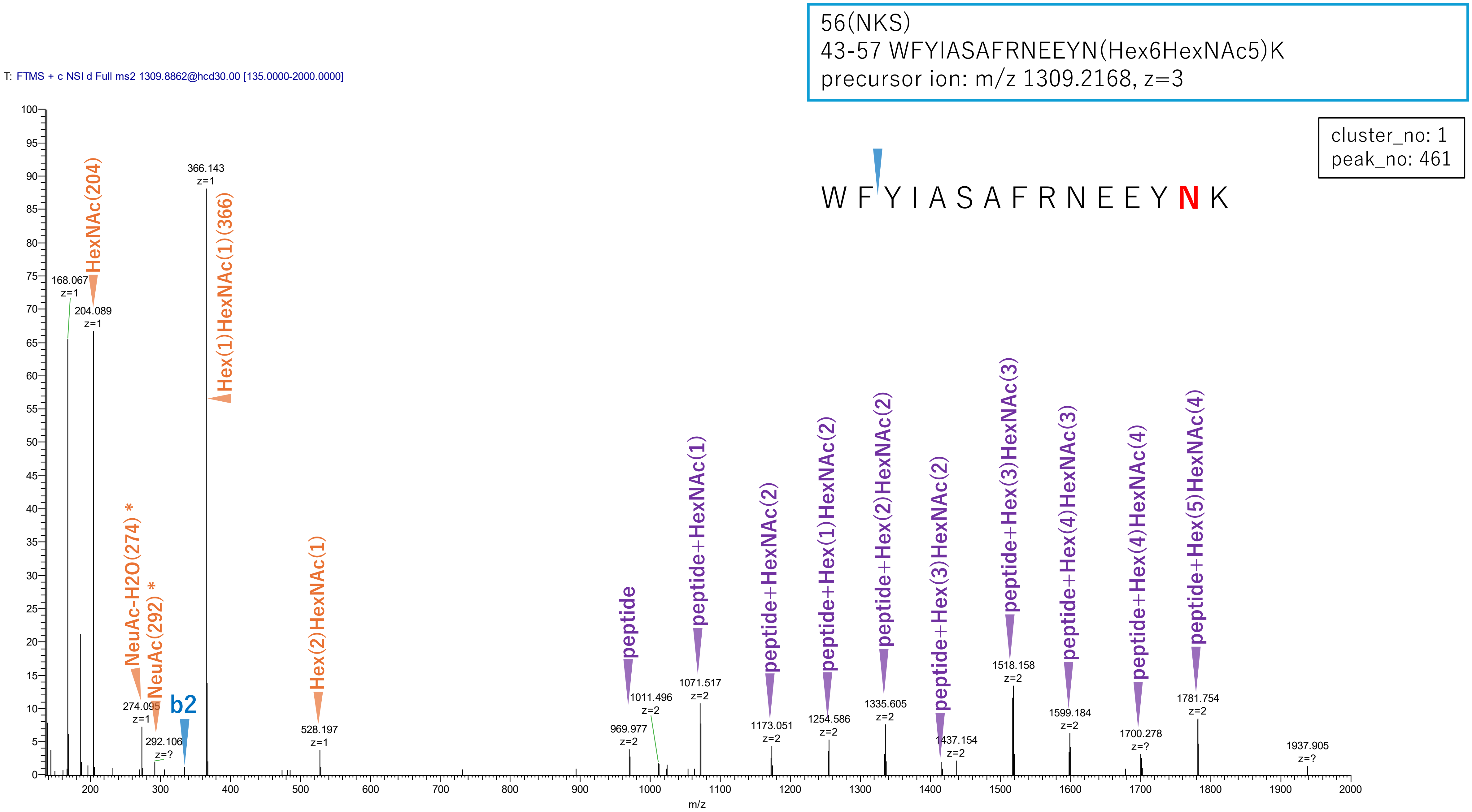

Figure S4-10. MS2 spectra of glycopeptides assigned for hAGP

*Asterisks indicate diagnostic ions of NeuAc derived from a contaminated glycopeptide.

56(NKS)
43-57 WFYIASAFRNEEYN(Hex6HexNAc5NeuAc1)K
precursor ion: m/z 1054.93787, z=4

cluster_no: 1
peak_no: 669

W F Y I A S A F R N E E Y **N** K

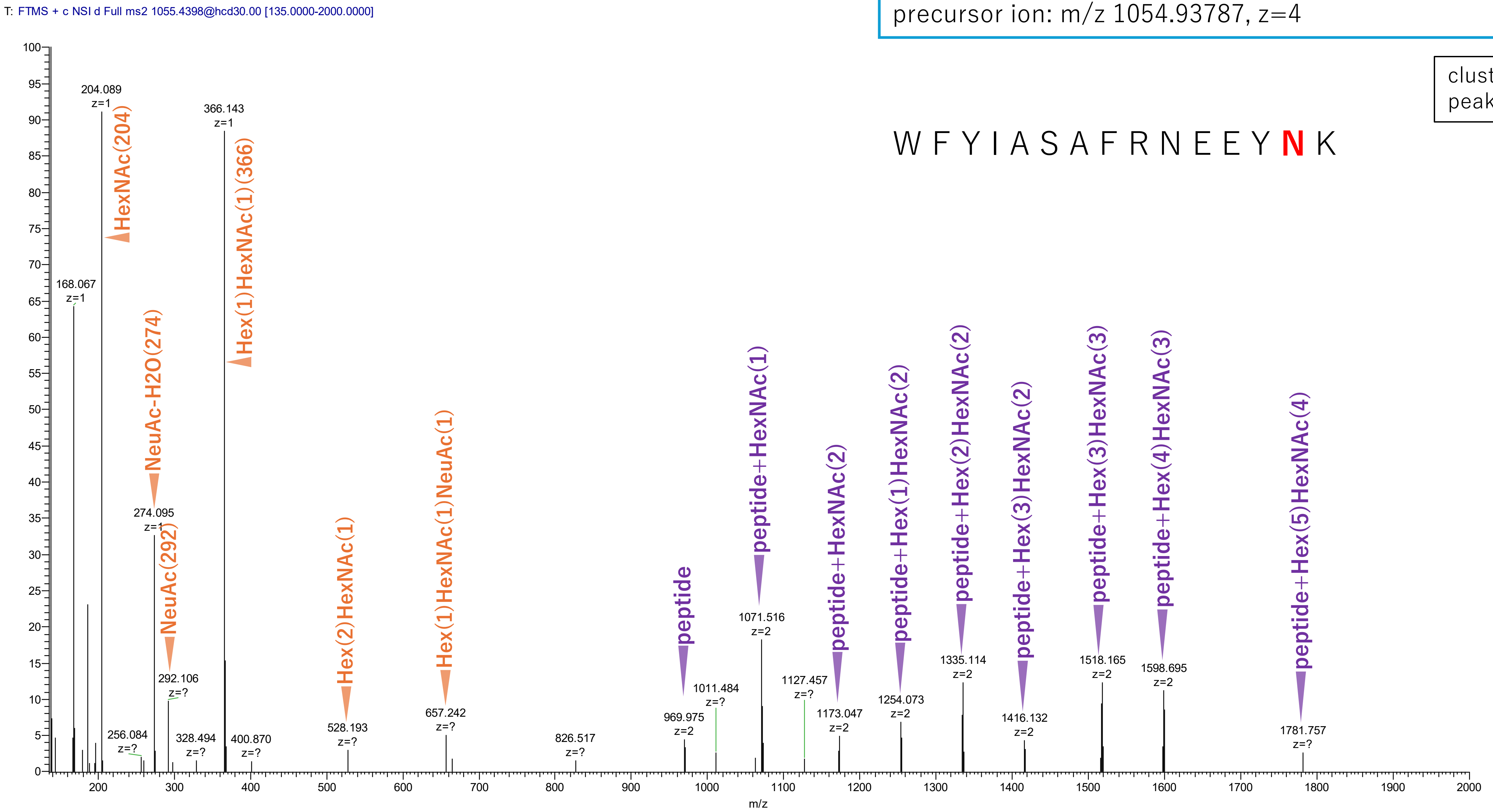

Figure S4-11. MS2 spectra of glycopeptides assigned for hAGP

56(NKS)
43-57 WFYIASAFRNEEYN(Hex6HexNAc5dHex1)K
precursor ion: m/z 1018.67743, z=4

cluster_no: 1
peak_no: 771

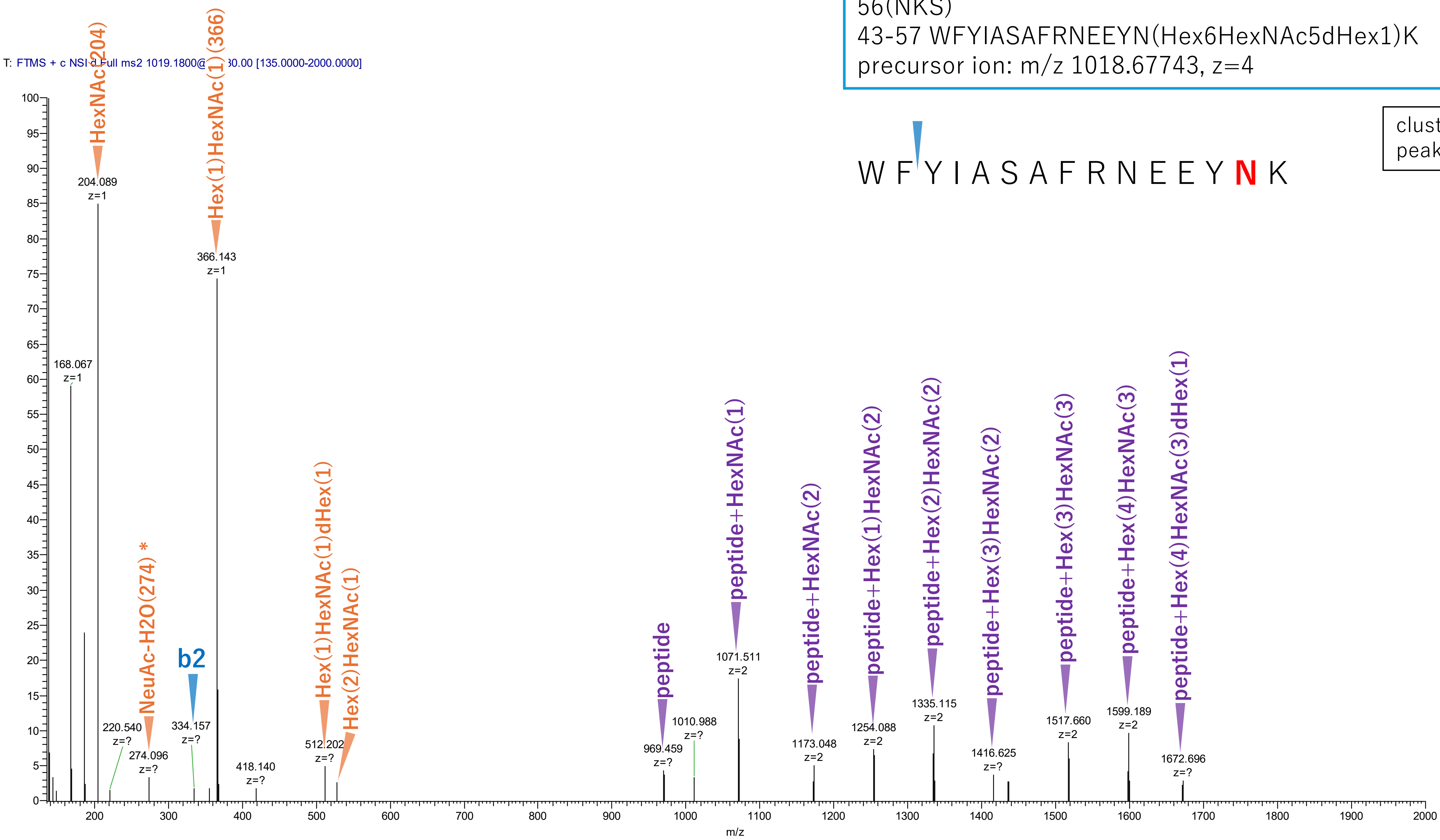

Figure S4-12. MS2 spectra of glycopeptides assigned for hAGP

*Asterisks indicate diagnostic ions of NeuAc derived from a contaminated glycopeptide.

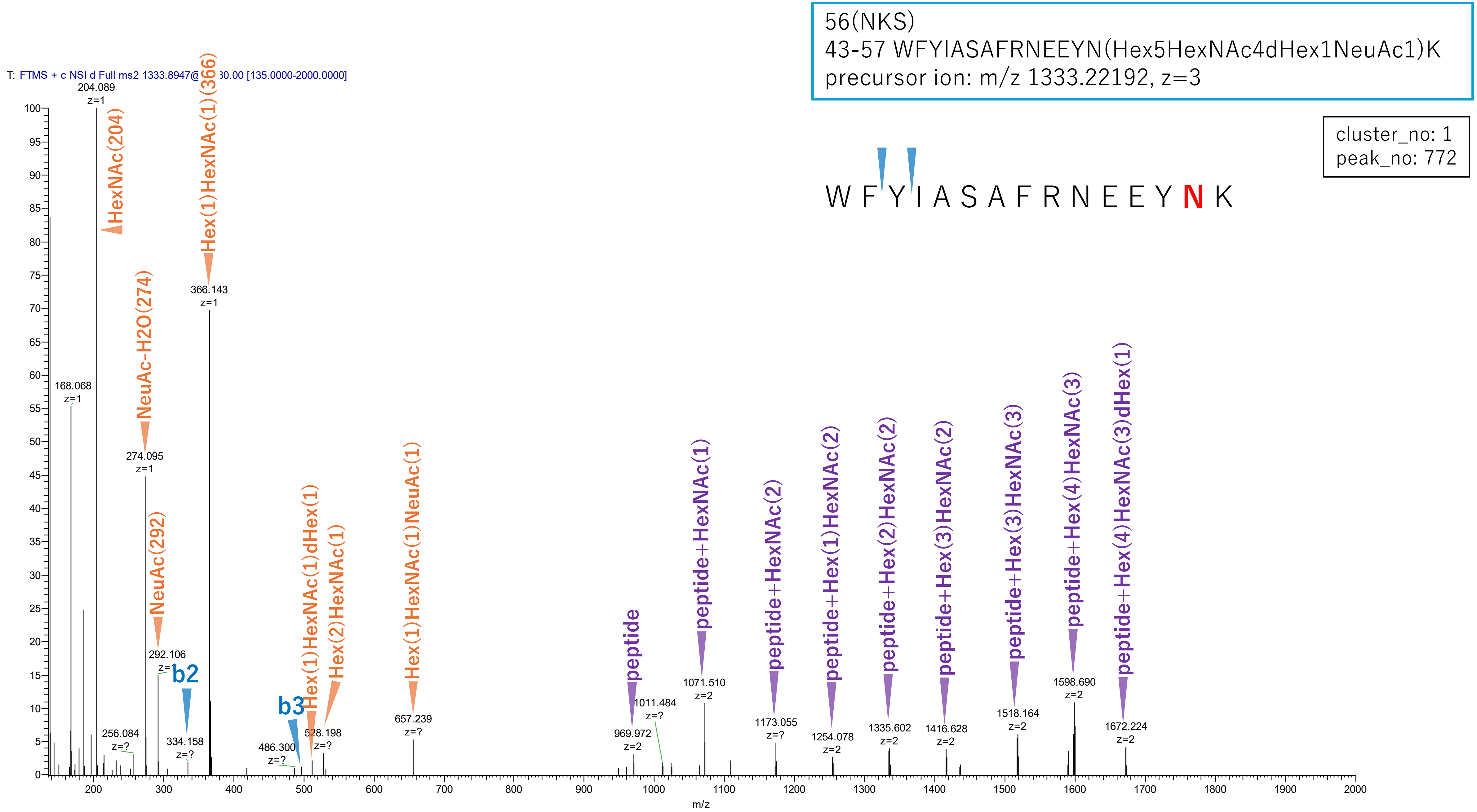

Figure S4-13. MS2 spectra of glycopeptides assigned for hAGP

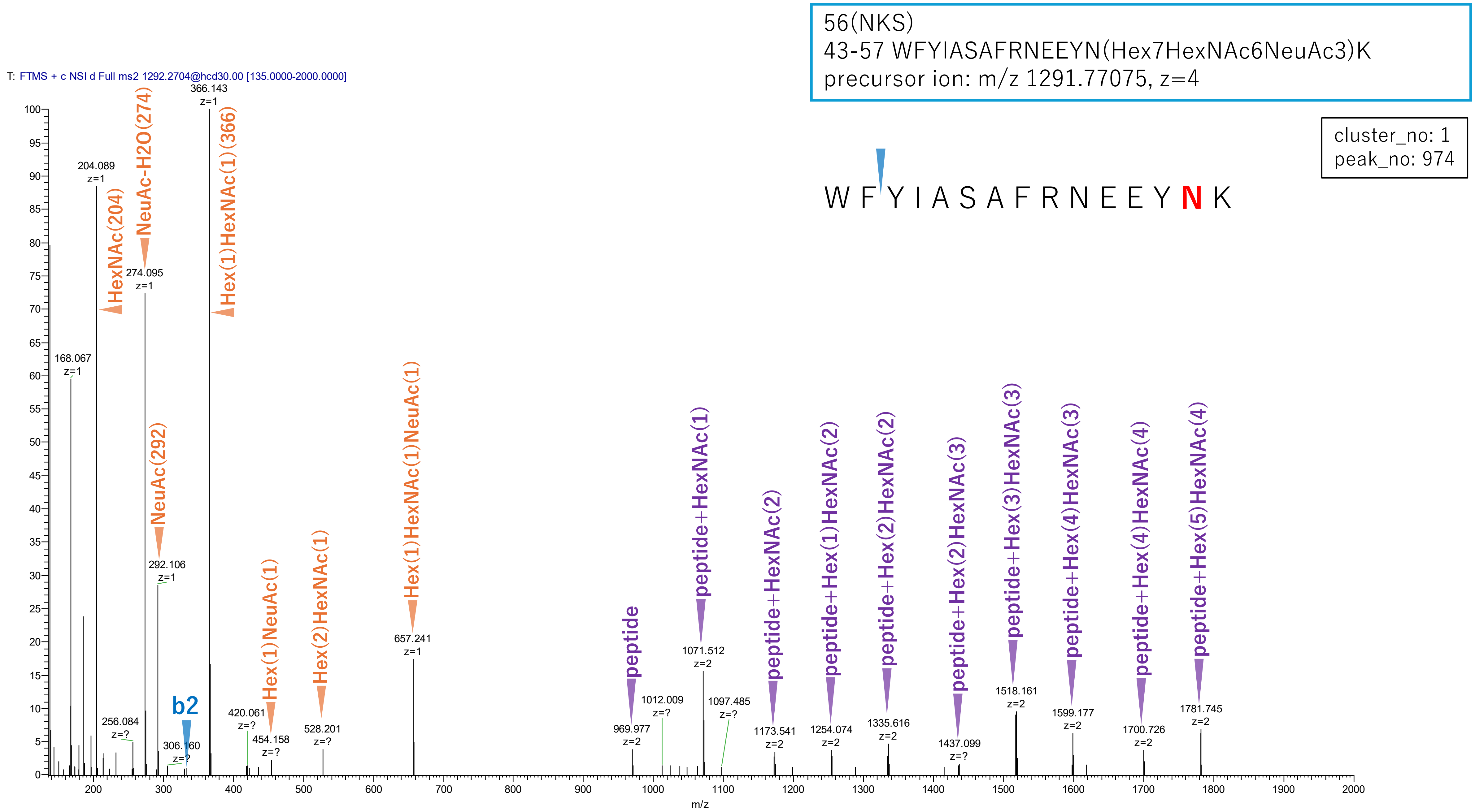

Figure S4-14. MS2 spectra of glycopeptides assigned for hAGP

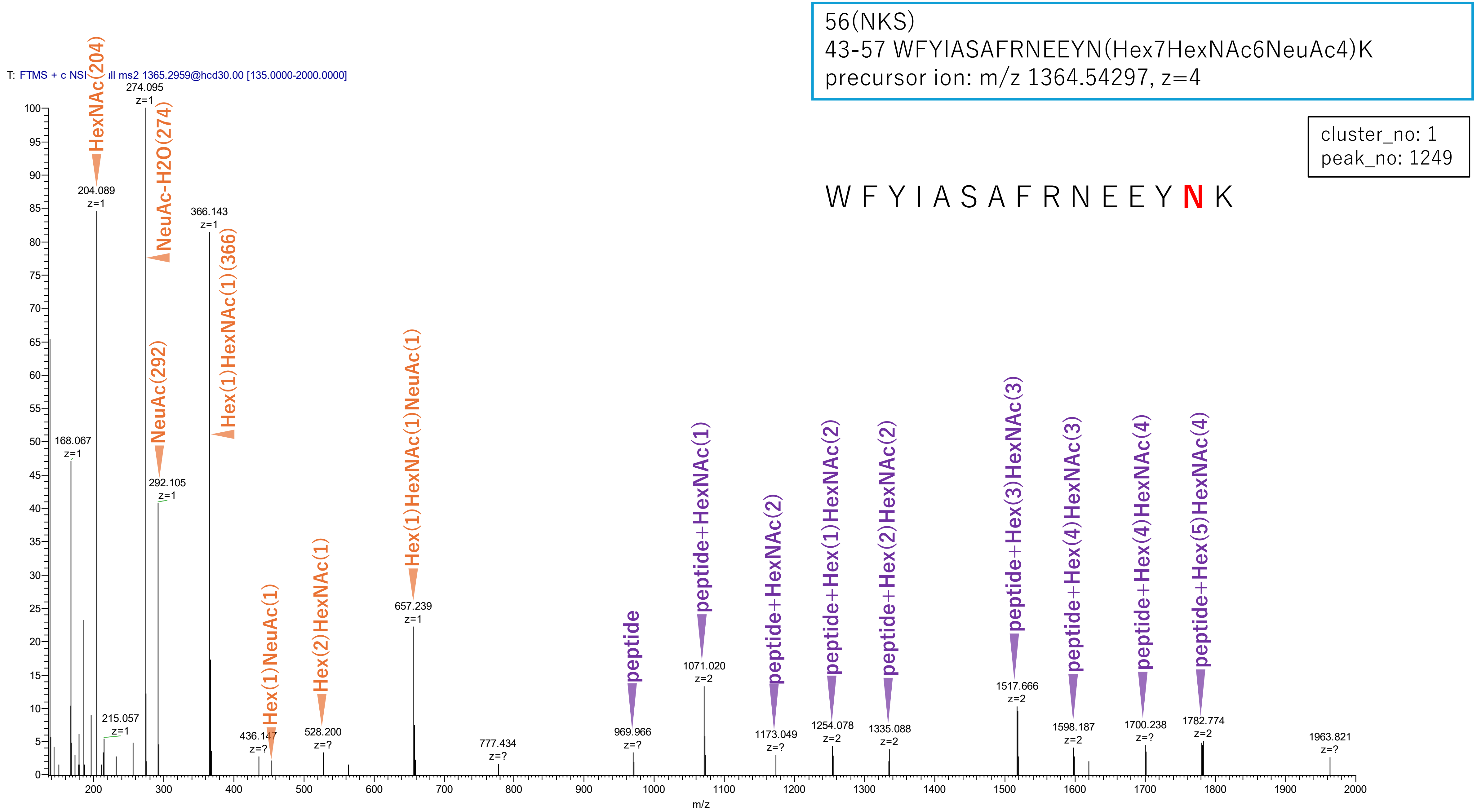

Figure S4-15. MS2 spectra of glycopeptides assigned for hAGP

56(NKS)
43-57 WFYIASAFRNEEYN(Hex6HexNAc5dHex1NeuAc1)K
precursor ion: m/z 1091.45264, z=4

cluster_no: 1
peak_no: 1293

W F Y I A S A F R N E E Y **N** K

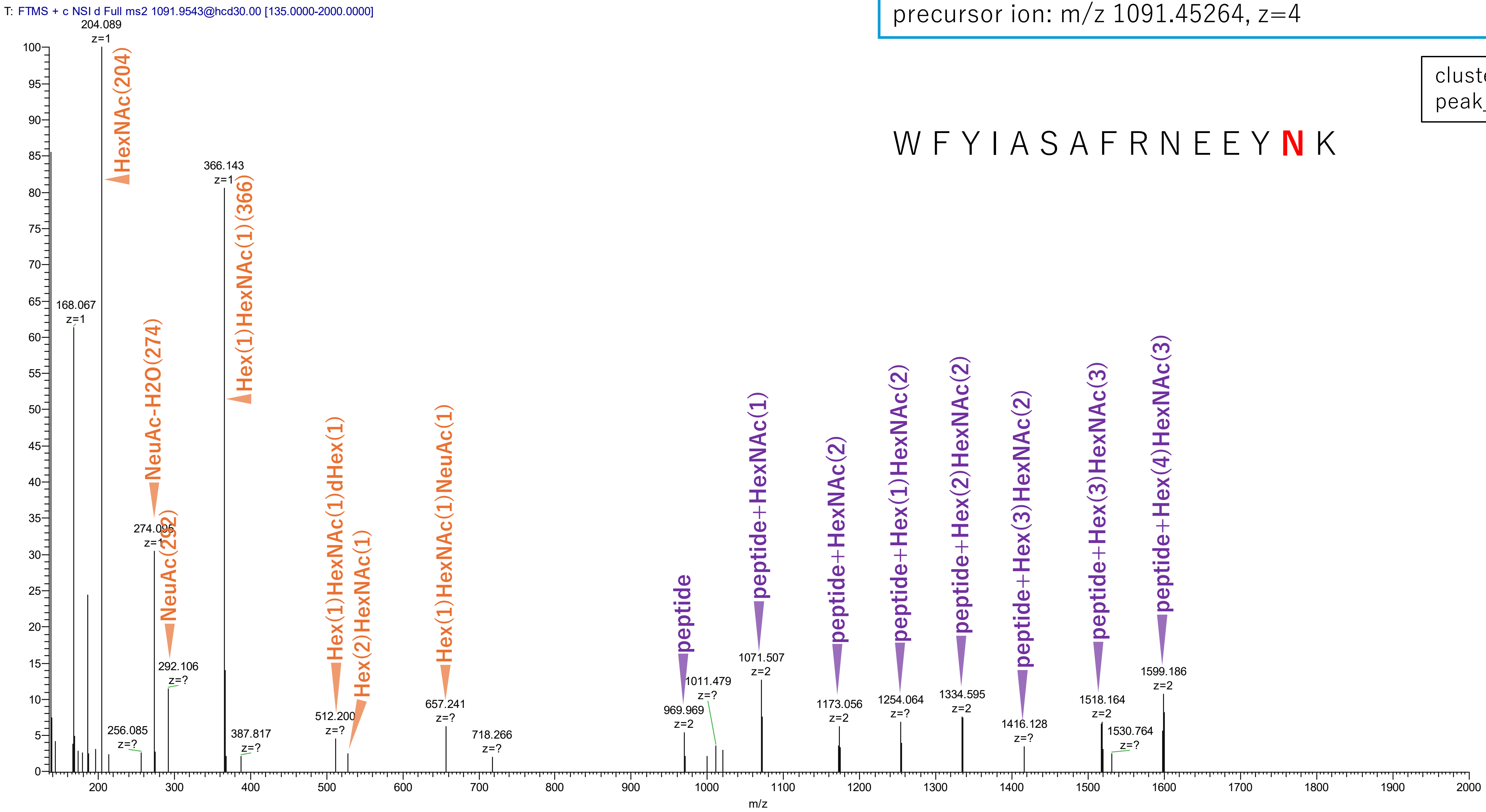

Figure S4-16. MS2 spectra of glycopeptides assigned for hAGP

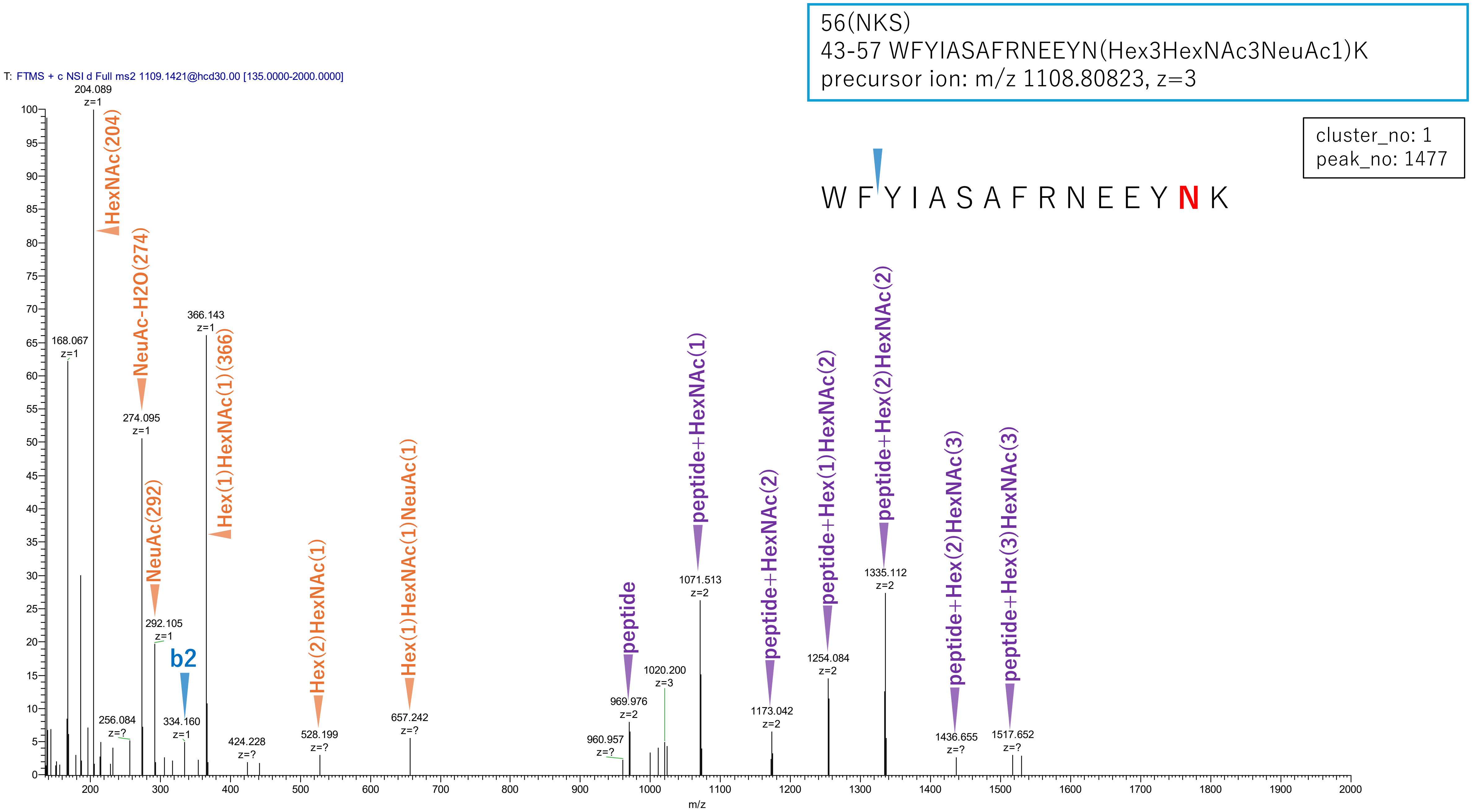

Figure S4-17. MS2 spectra of glycopeptides assigned for hAGP

56(NKS)
43-57 WFYIASAFRNEEYN(Hex7HexNAc6dHex1NeuAc3)K
precursor ion: m/z 1328.28418, z=4

cluster_no: 1
peak_no: 1619

W F Y I A S A F R N E E Y **N** K

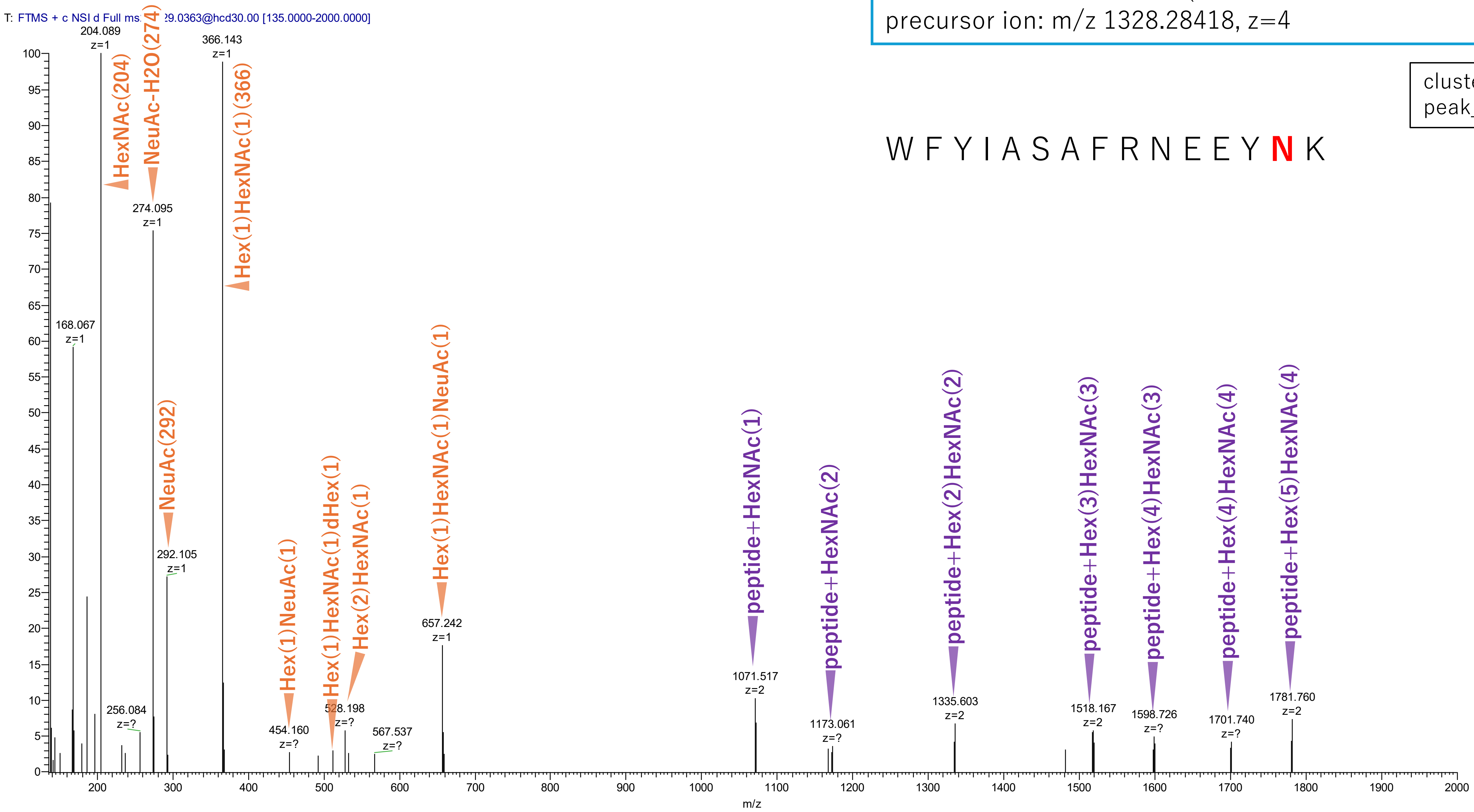

Figure S4-18. MS2 spectra of glycopeptides assigned for hAGP

56(NKS)
43-57 WFYIASAFRNEEYN(Hex5HexNAc4dHex1)K
precursor ion: m/z 1236.18945, z=3

cluster_no: 1
peak_no: 1860

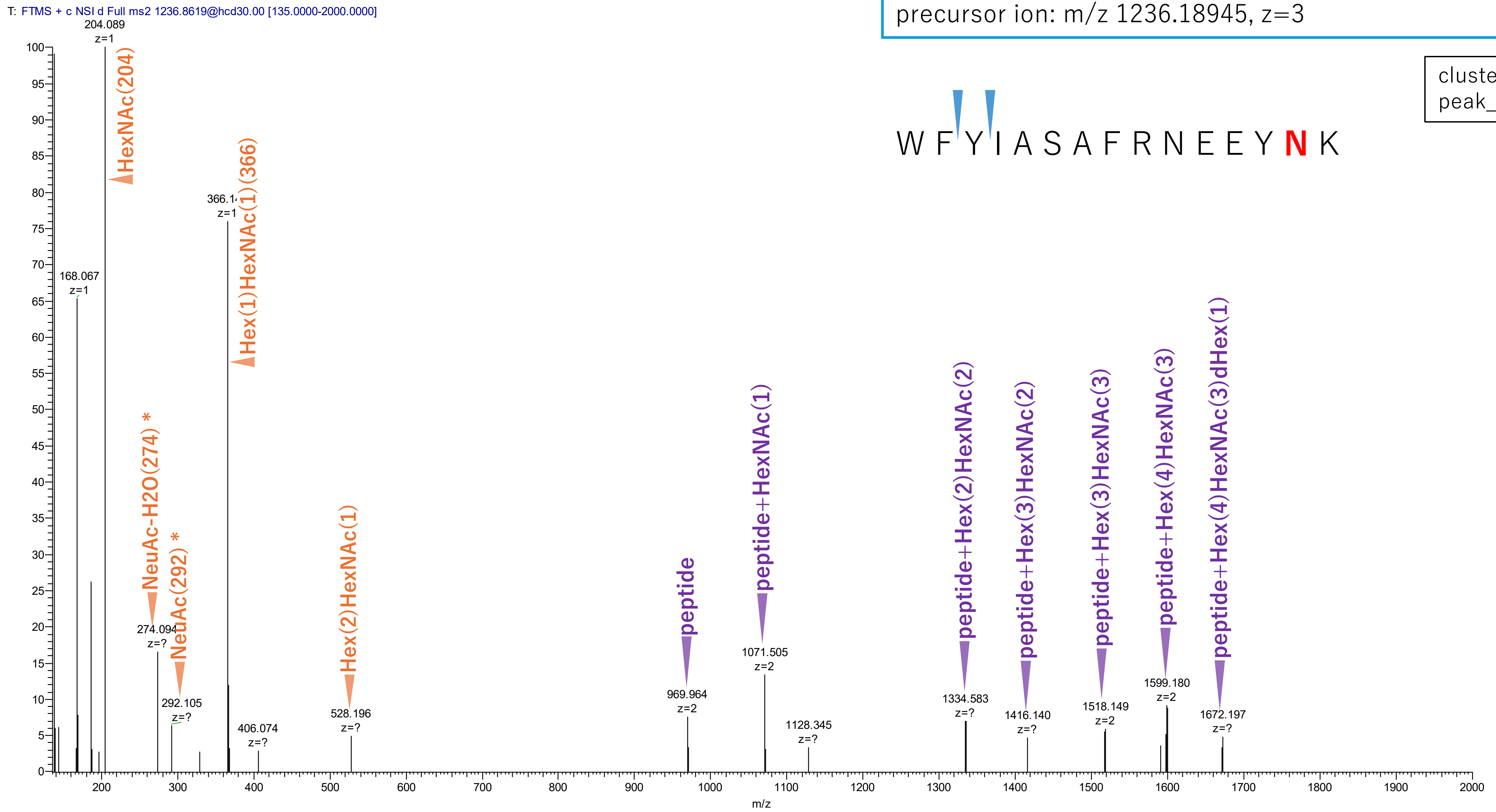

Figure S4-19. MS2 spectra of glycopeptides assigned for hAGP

*Asterisks indicate diagnostic ions of NeuAc derived from a contaminated glycopeptide.

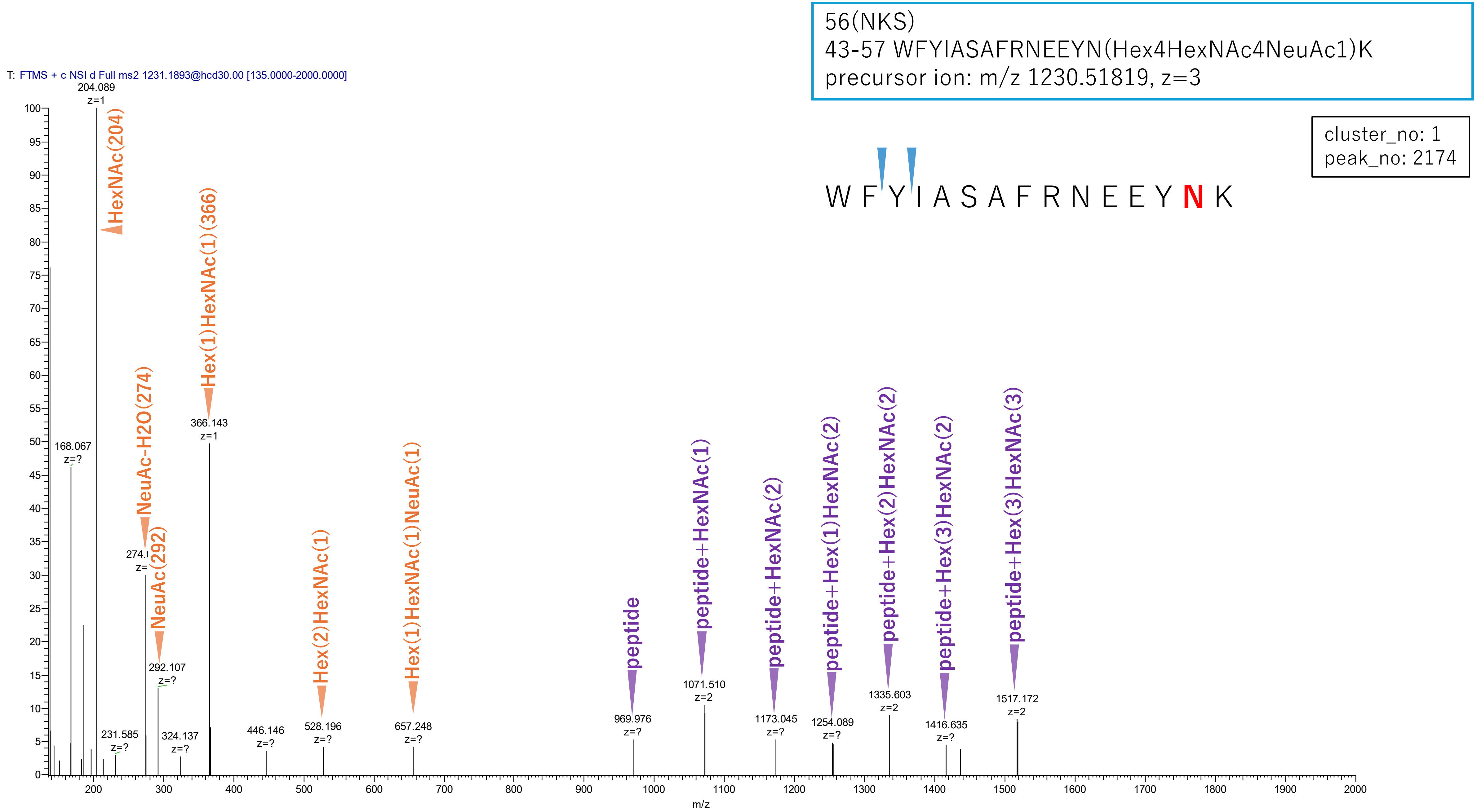

Figure S4-20. MS2 spectra of glycopeptides assigned for hAGP

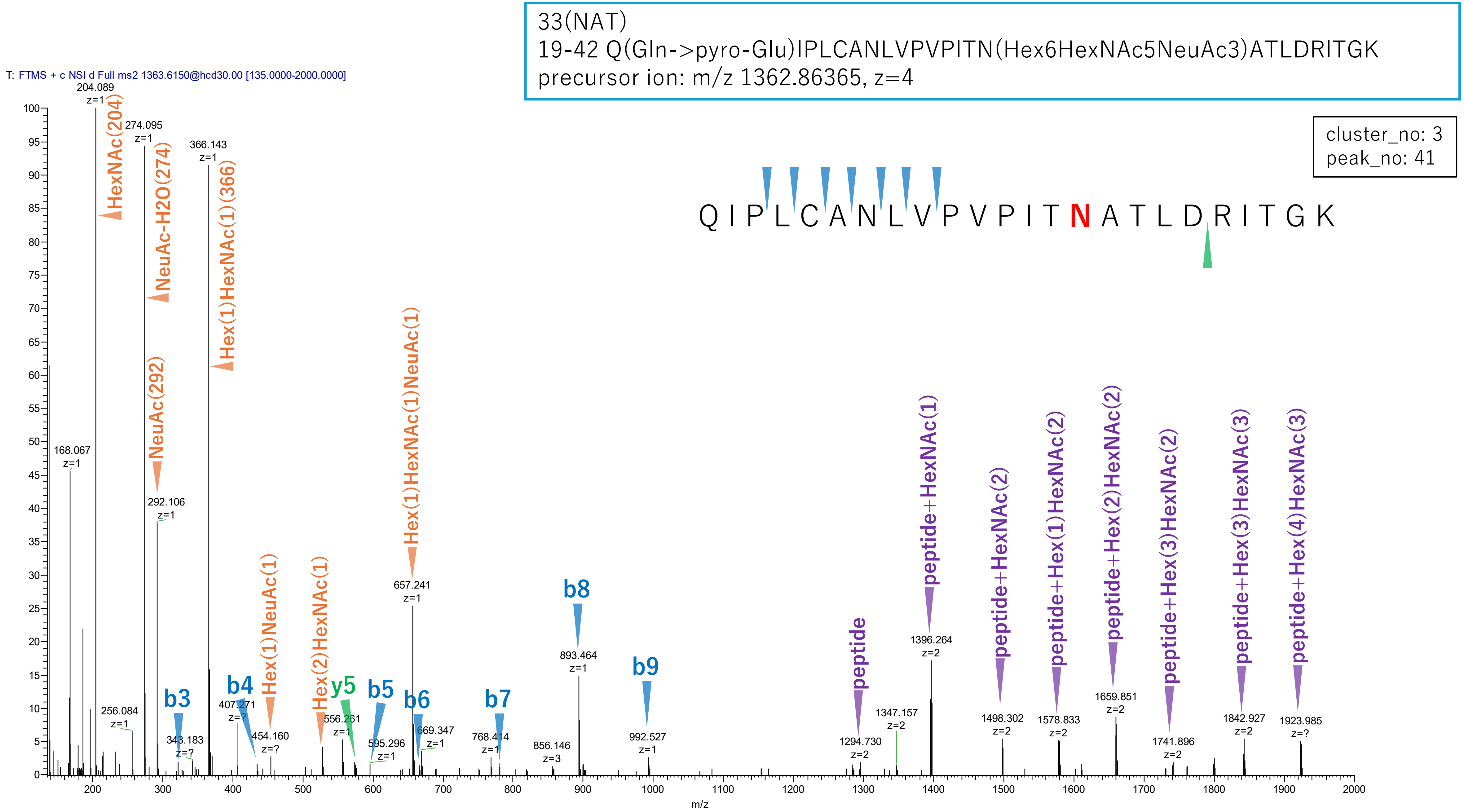

Figure S4-21. MS2 spectra of glycopeptides assigned for hAGP

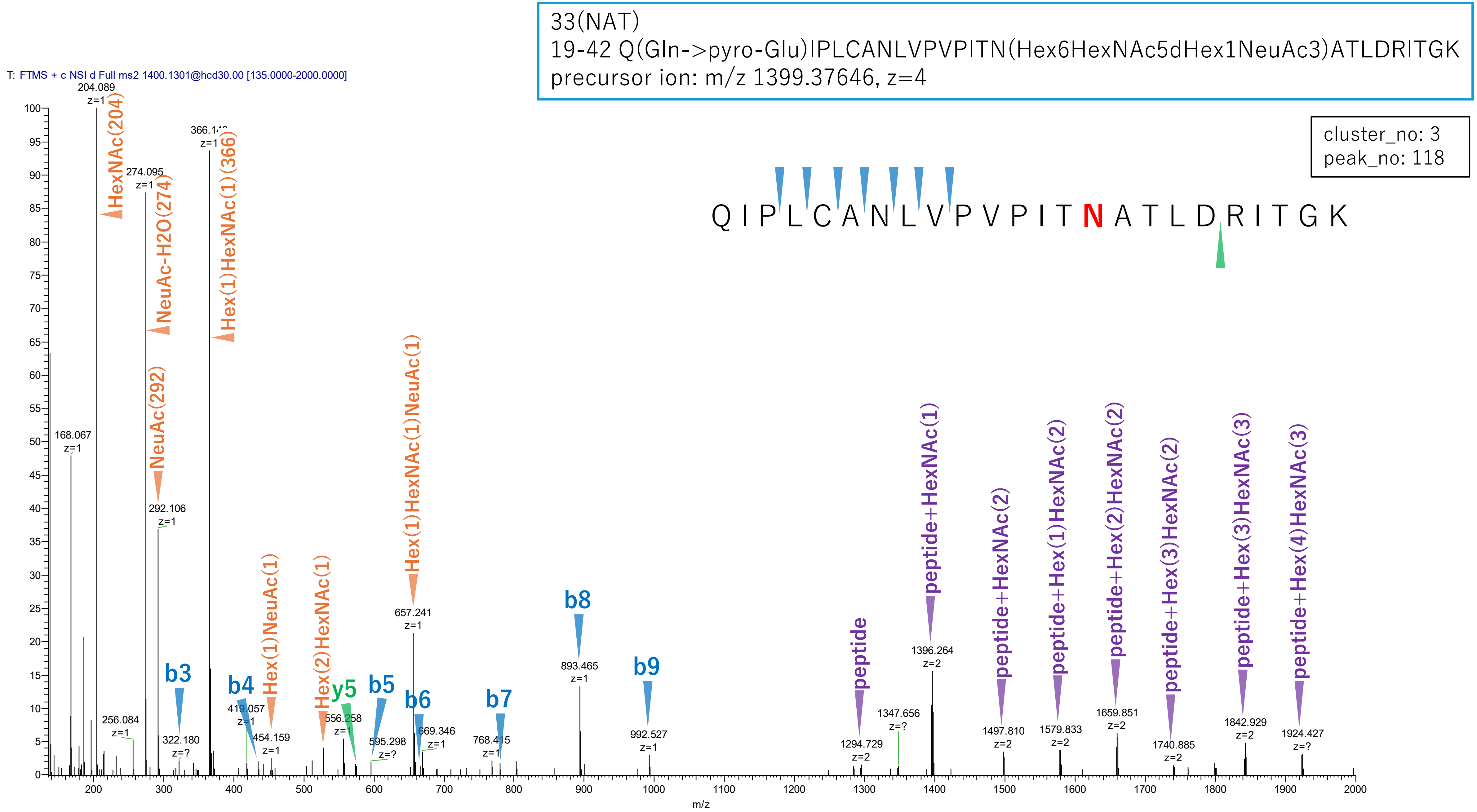

Figure S4-22. MS2 spectra of glycopeptides assigned for hAGP

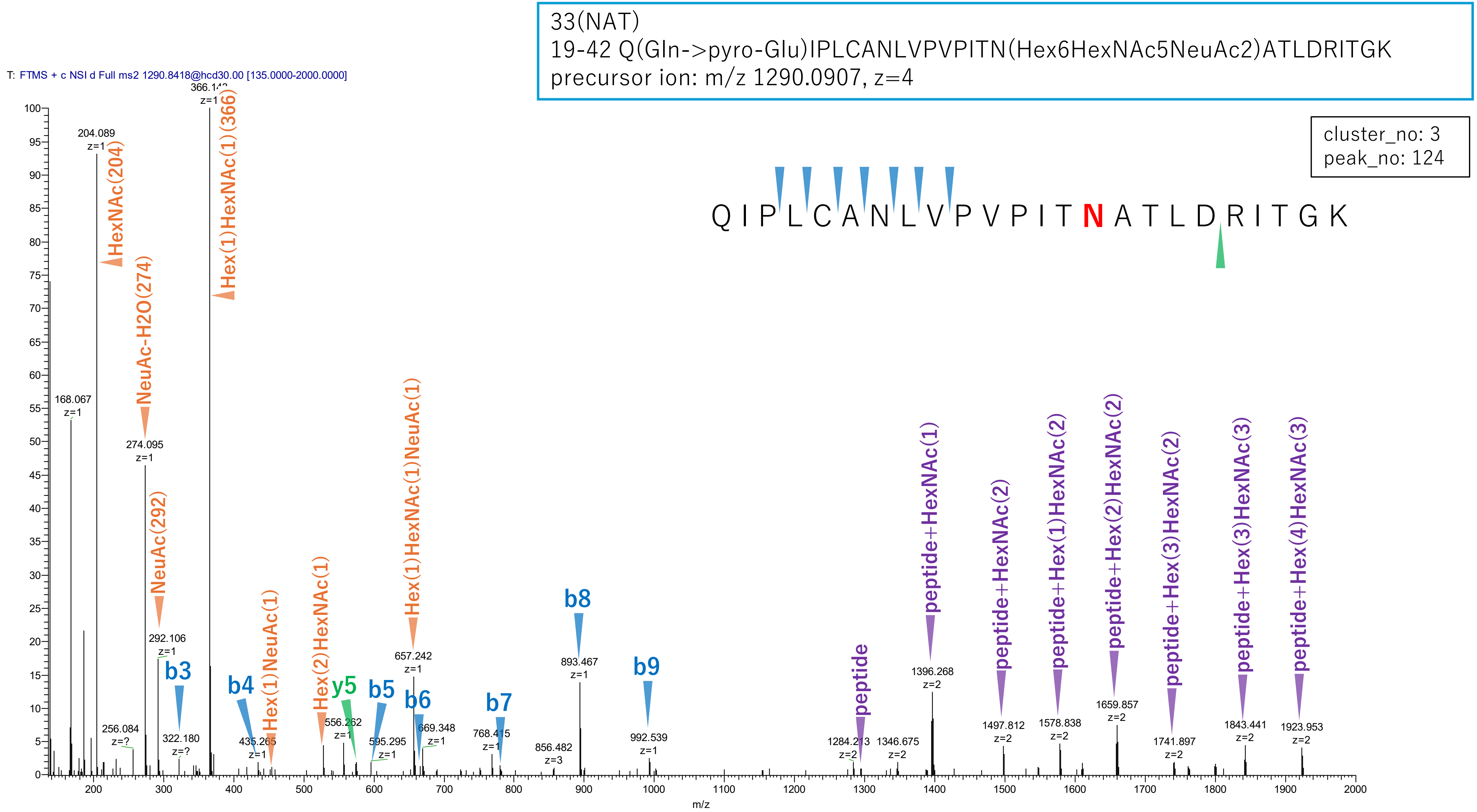

Figure S4-23. MS2 spectra of glycopeptides assigned for hAGP

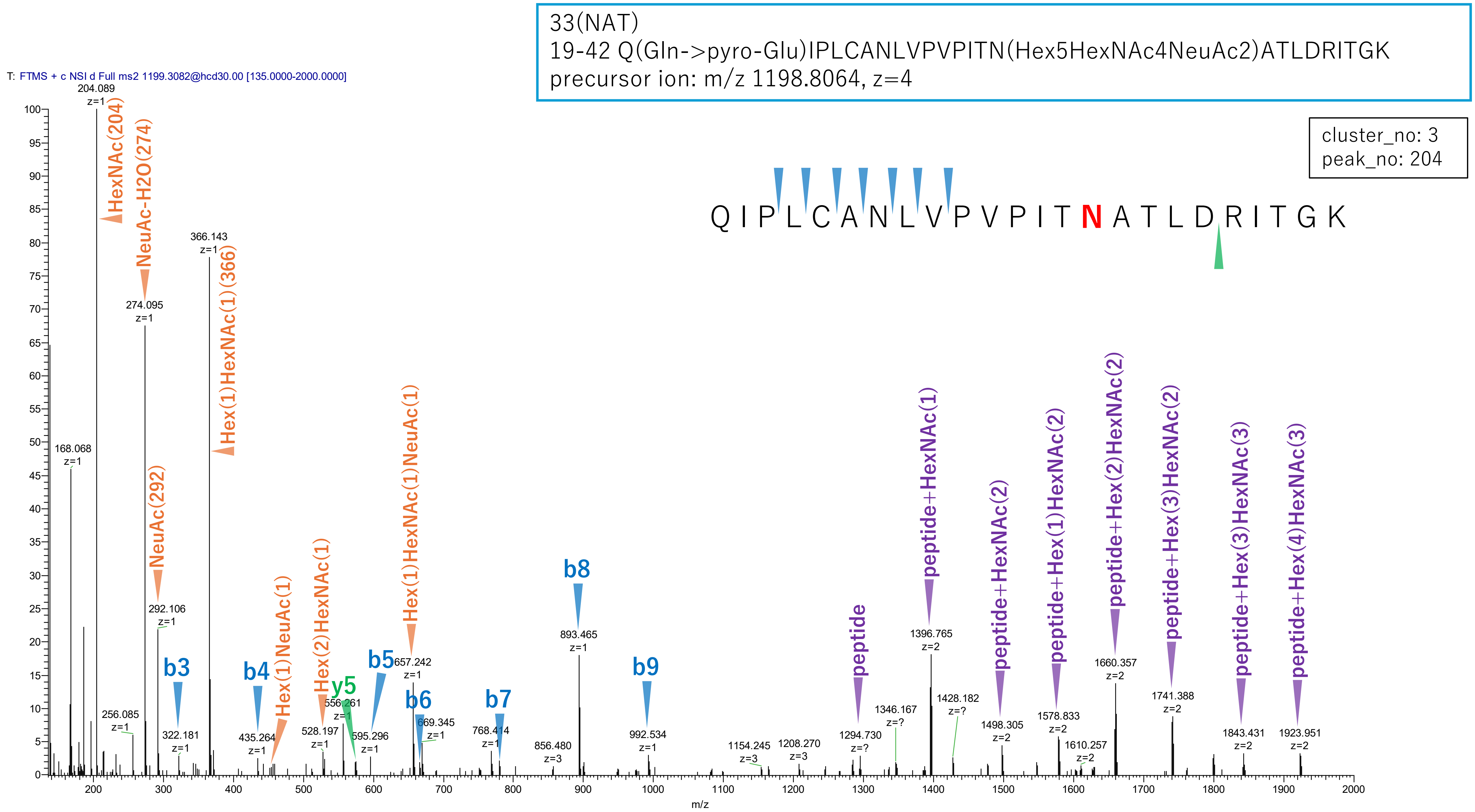

Figure S4-24. MS2 spectra of glycopeptides assigned for hAGP

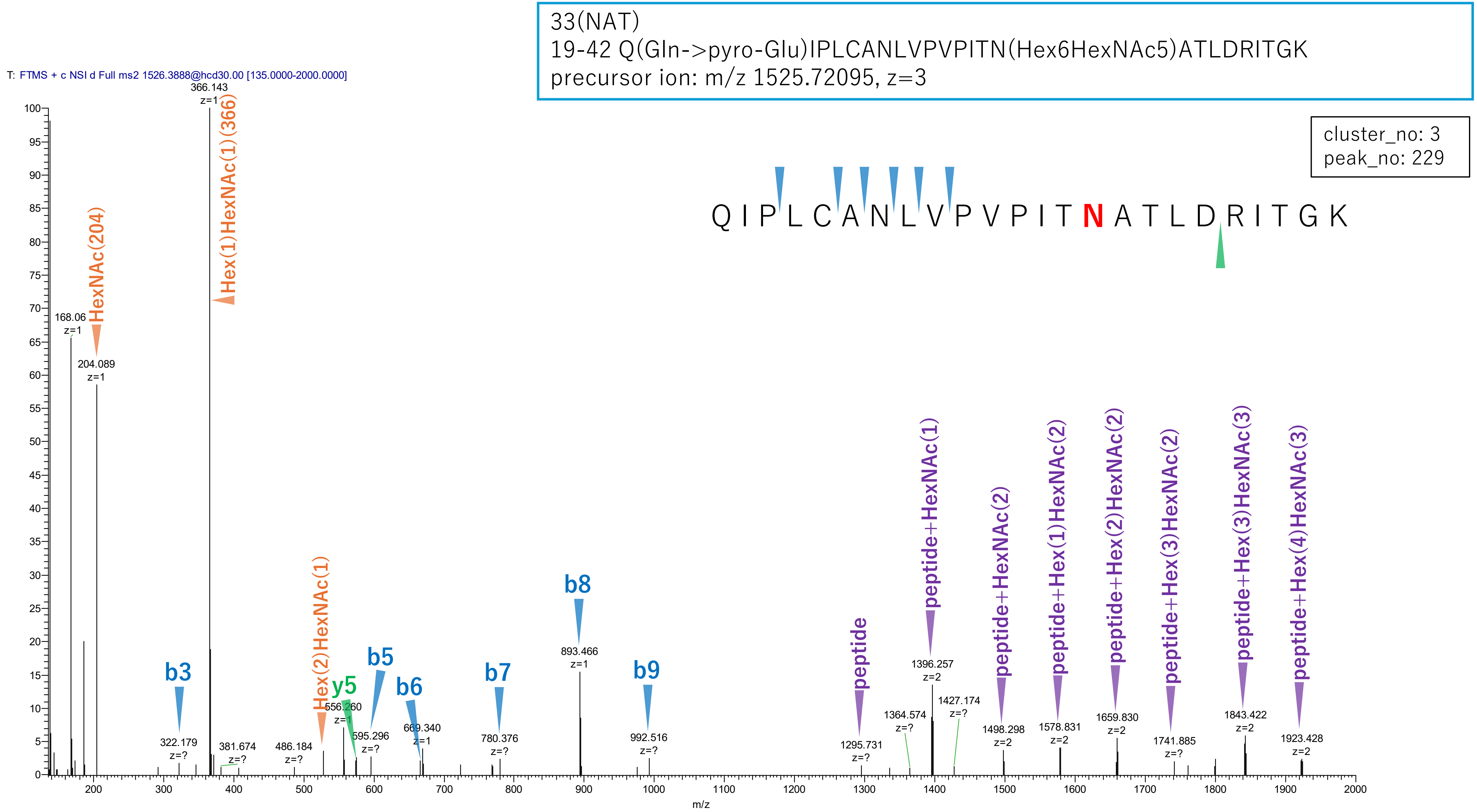

Figure S4-25. MS2 spectra of glycopeptides assigned for hAGP

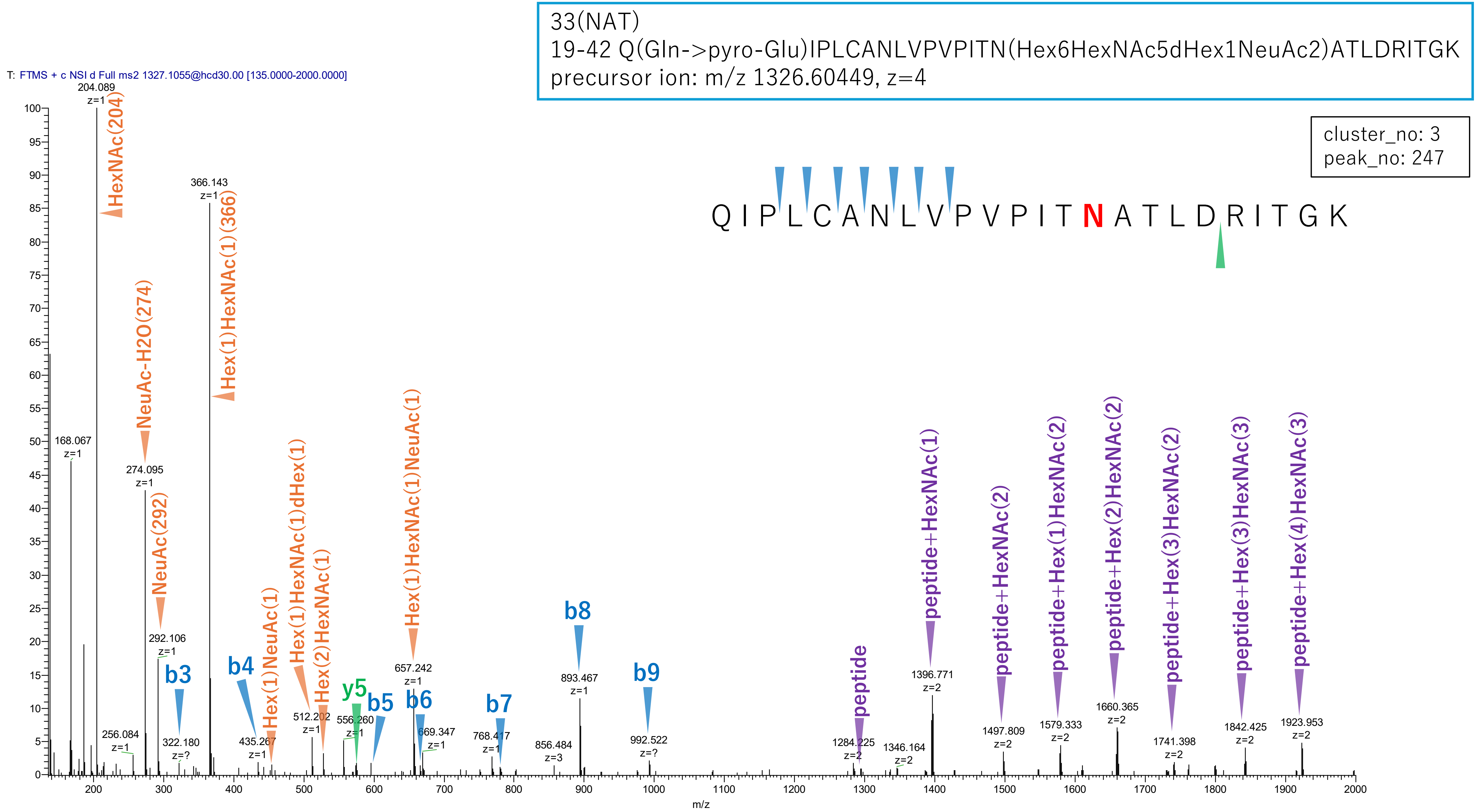

Figure S4-26. MS2 spectra of glycopeptides assigned for hAGP

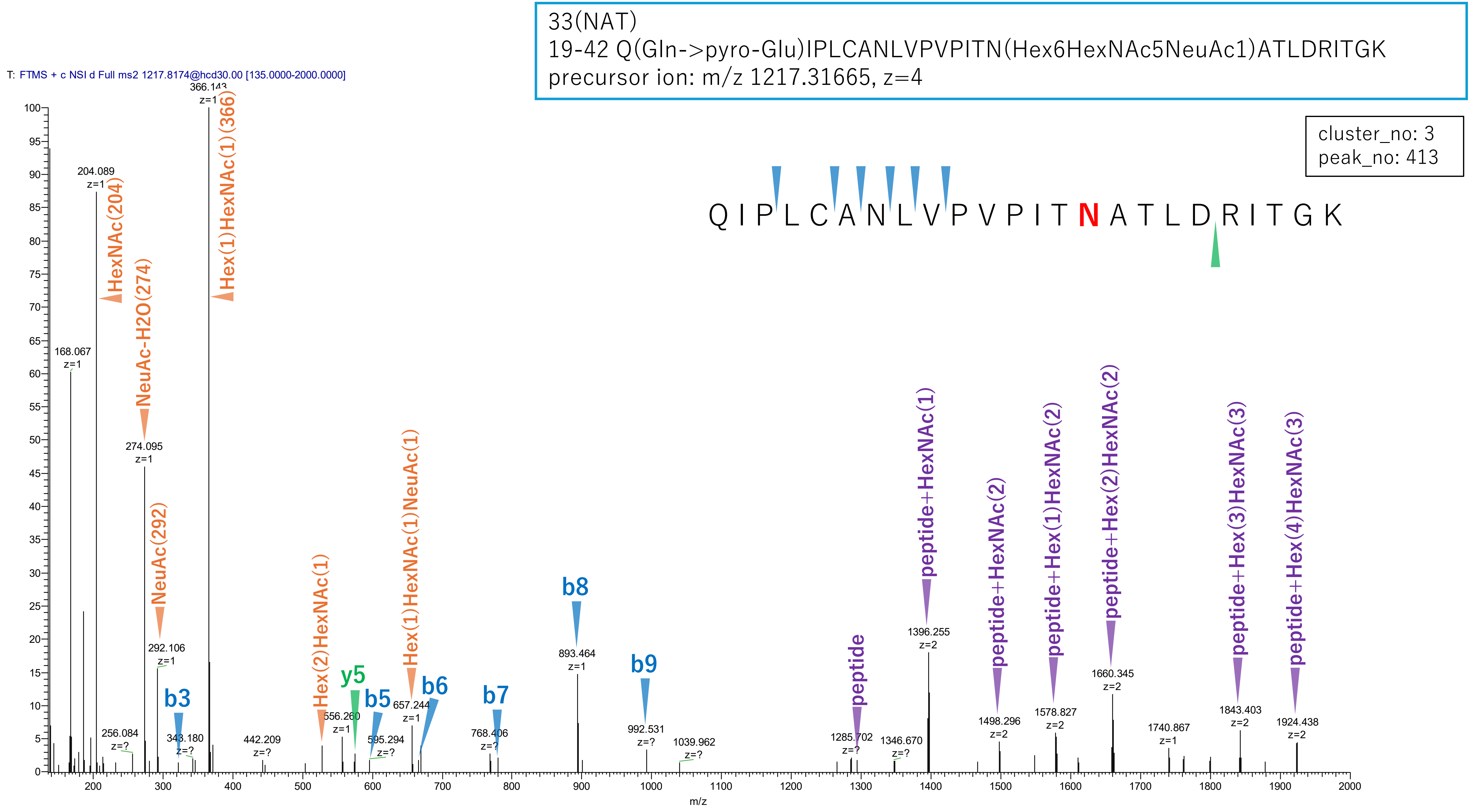

Figure S4-27. MS2 spectra of glycopeptides assigned for hAGP

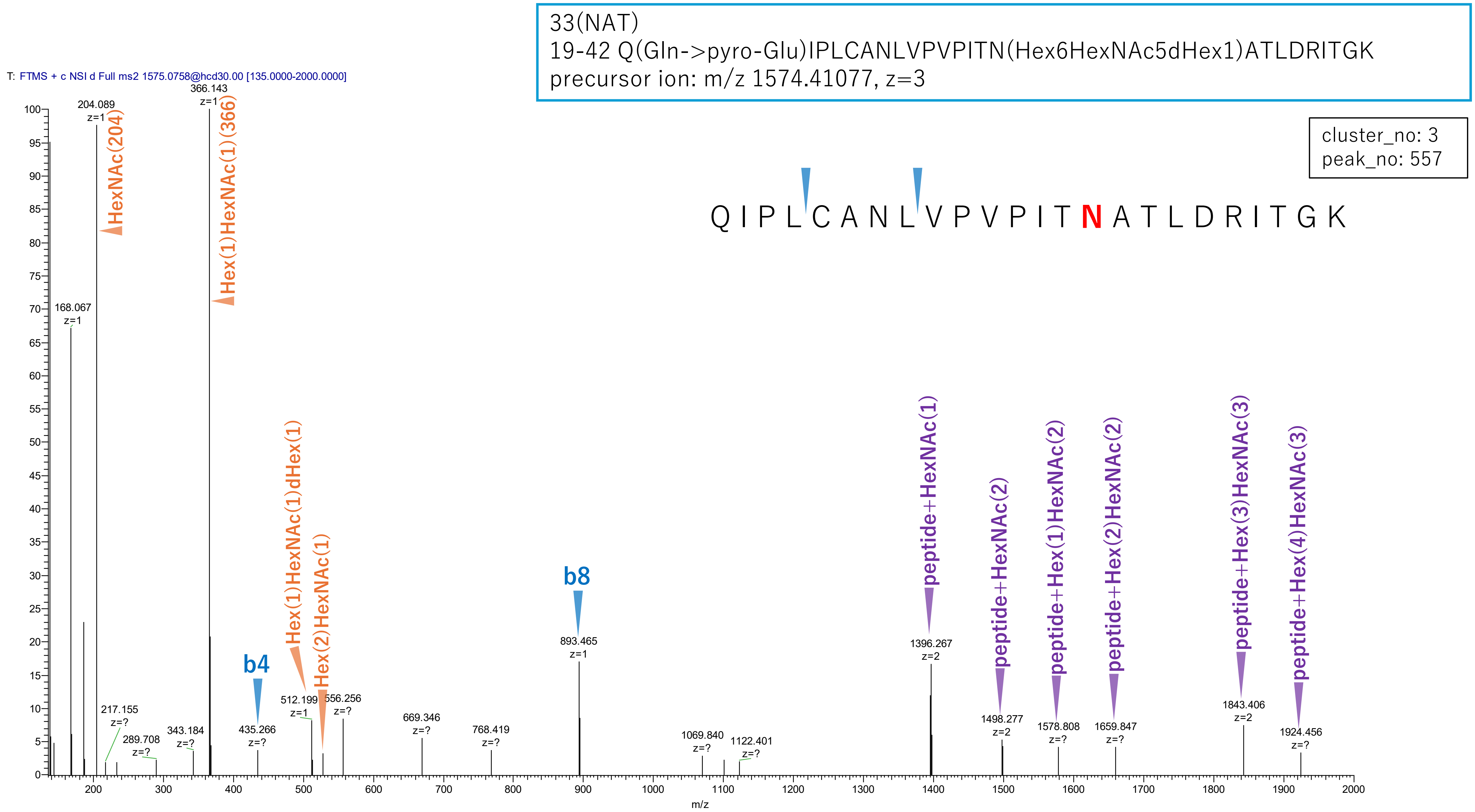

Figure S4-28. MS2 spectra of glycopeptides assigned for hAGP

33(NAT)
19-42 Q(Gln->pyro-Glu)IPLCANLVPVPITN(Hex7HexNAc6NeuAc3)ATLDRITGK
precursor ion: m/z 1454.14612, z=4

cluster_no: 3
peak_no: 854

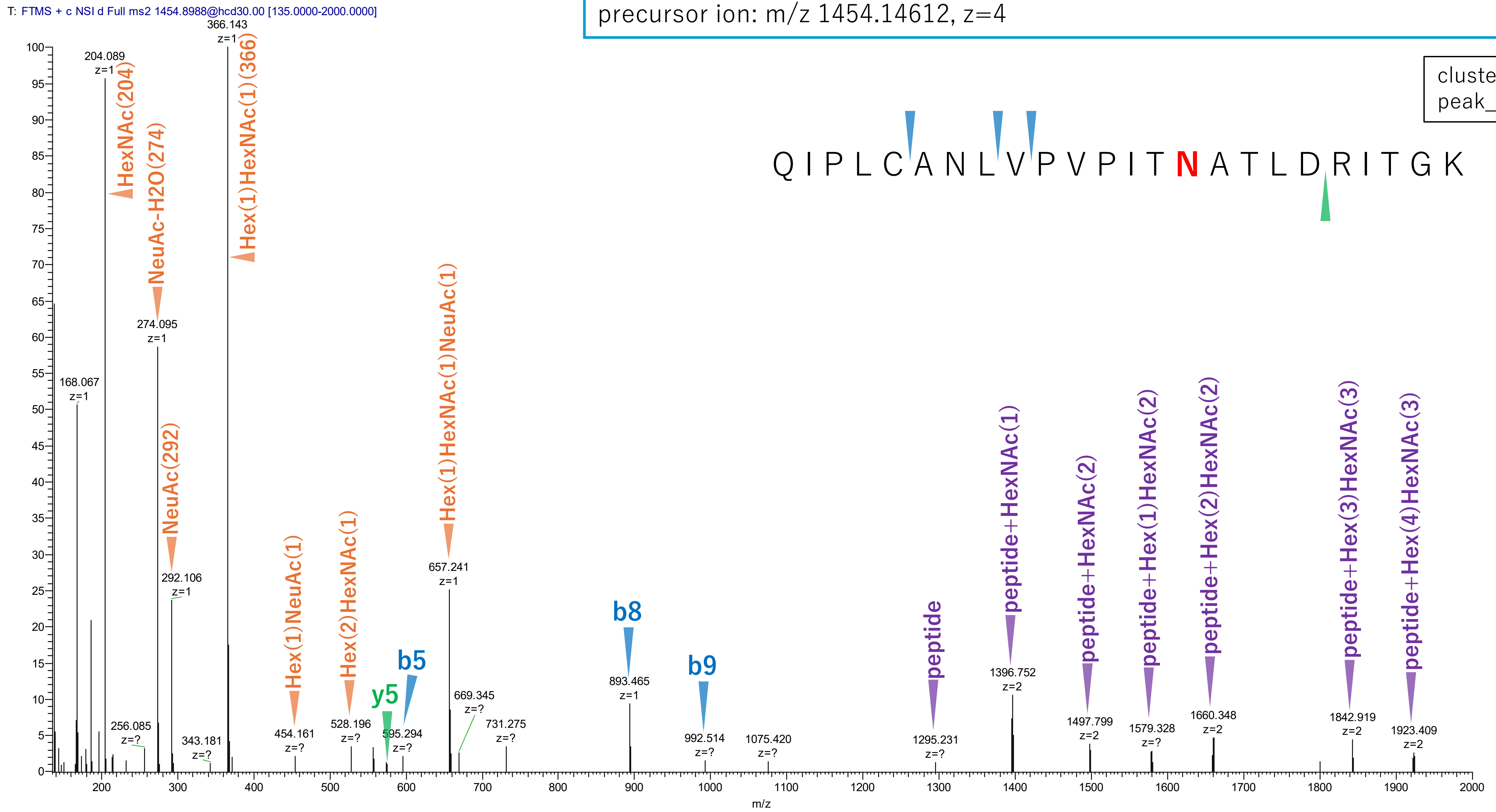

Figure S4-29. MS2 spectra of glycopeptides assigned for hAGP

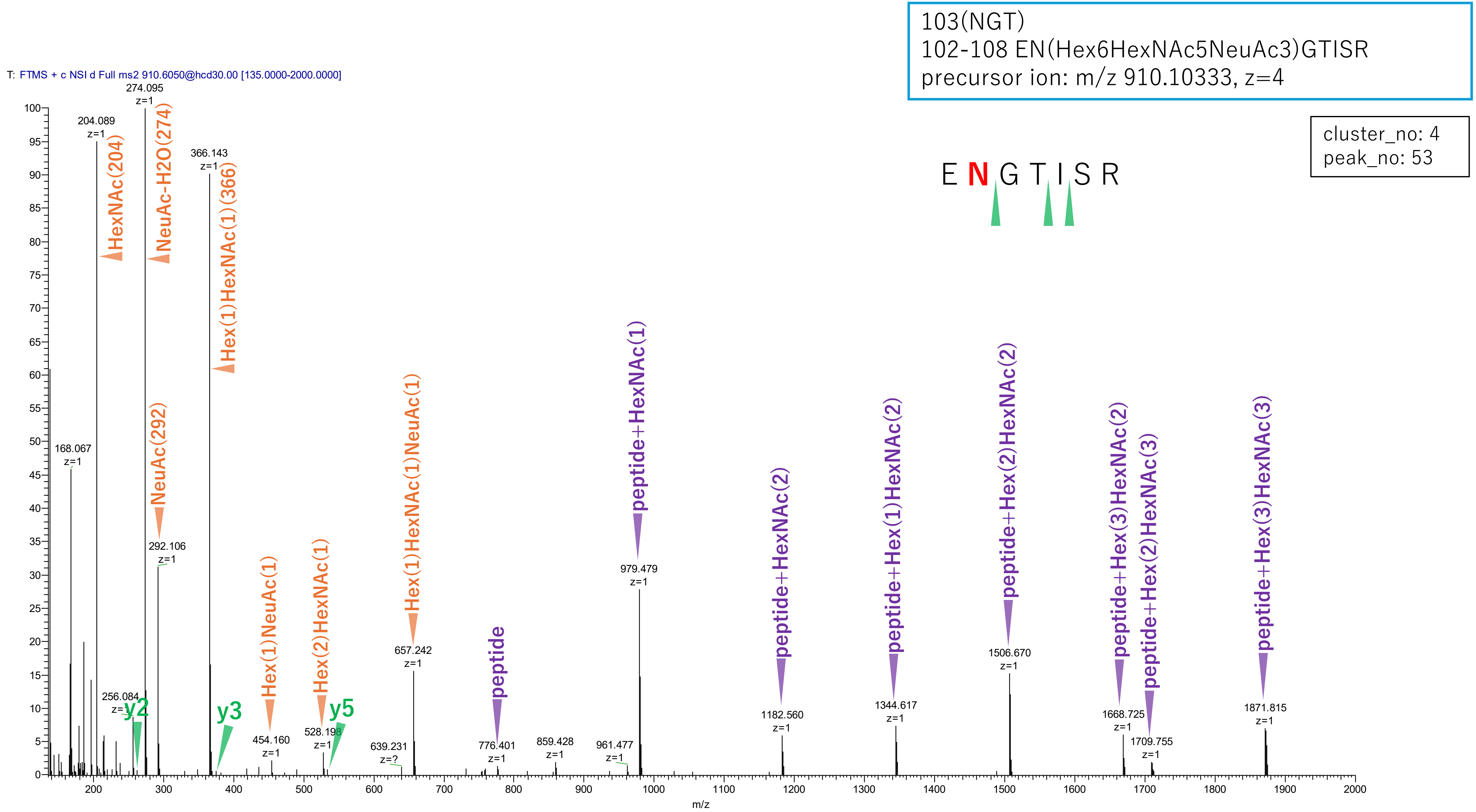

Figure S4-30. MS2 spectra of glycopeptides assigned for hAGP

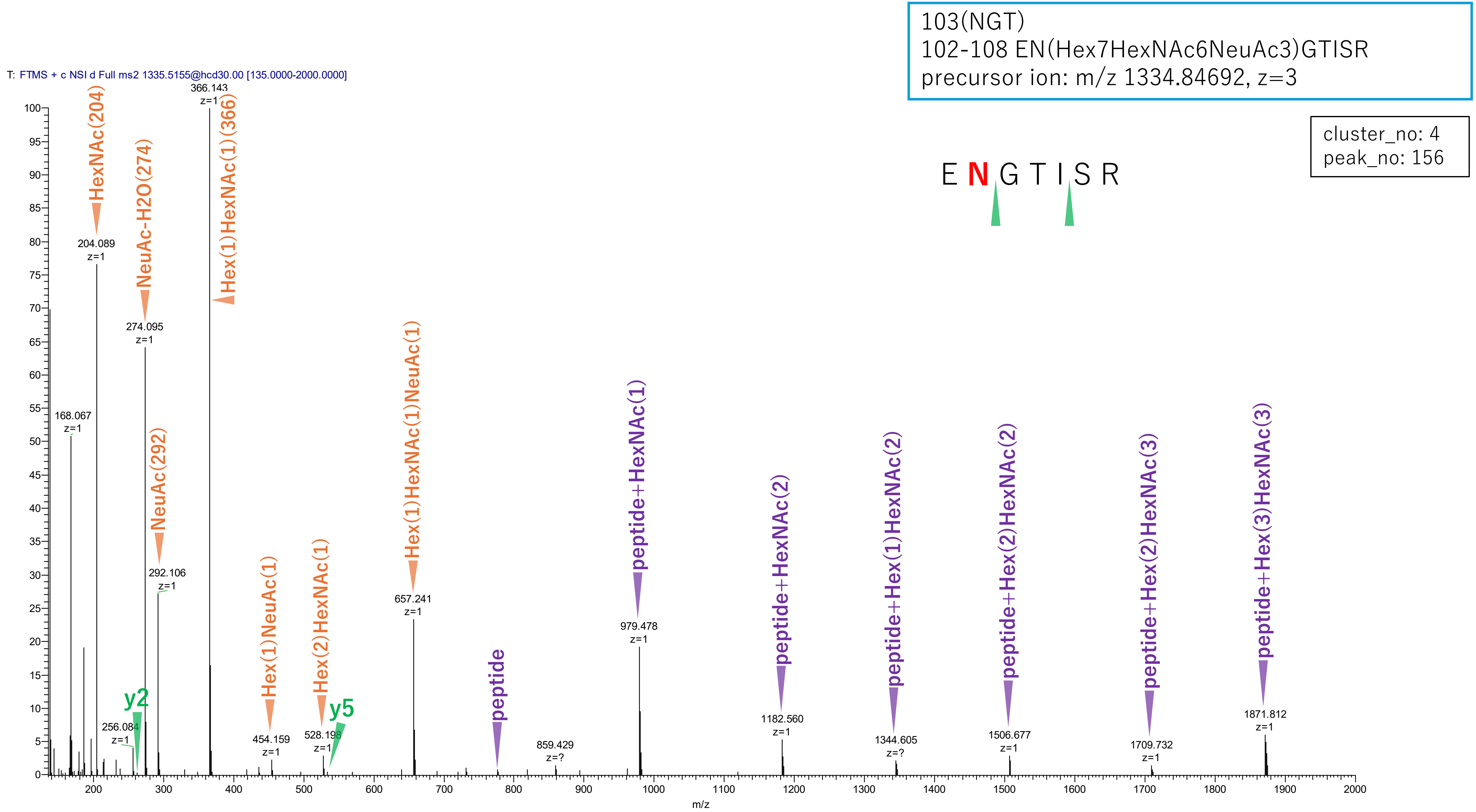

Figure S4-31. MS2 spectra of glycopeptides assigned for hAGP

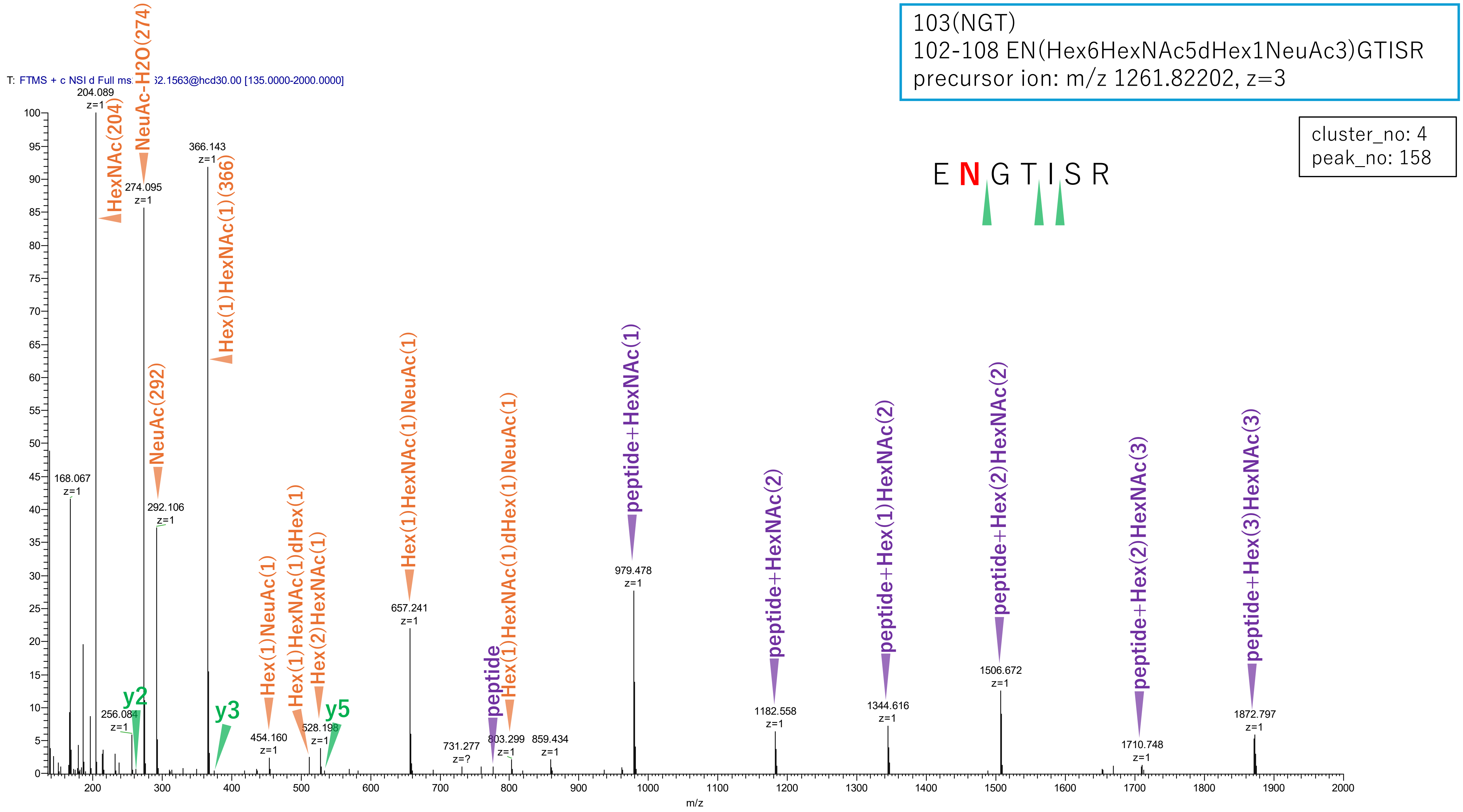

Figure S4-32. MS2 spectra of glycopeptides assigned for hAGP

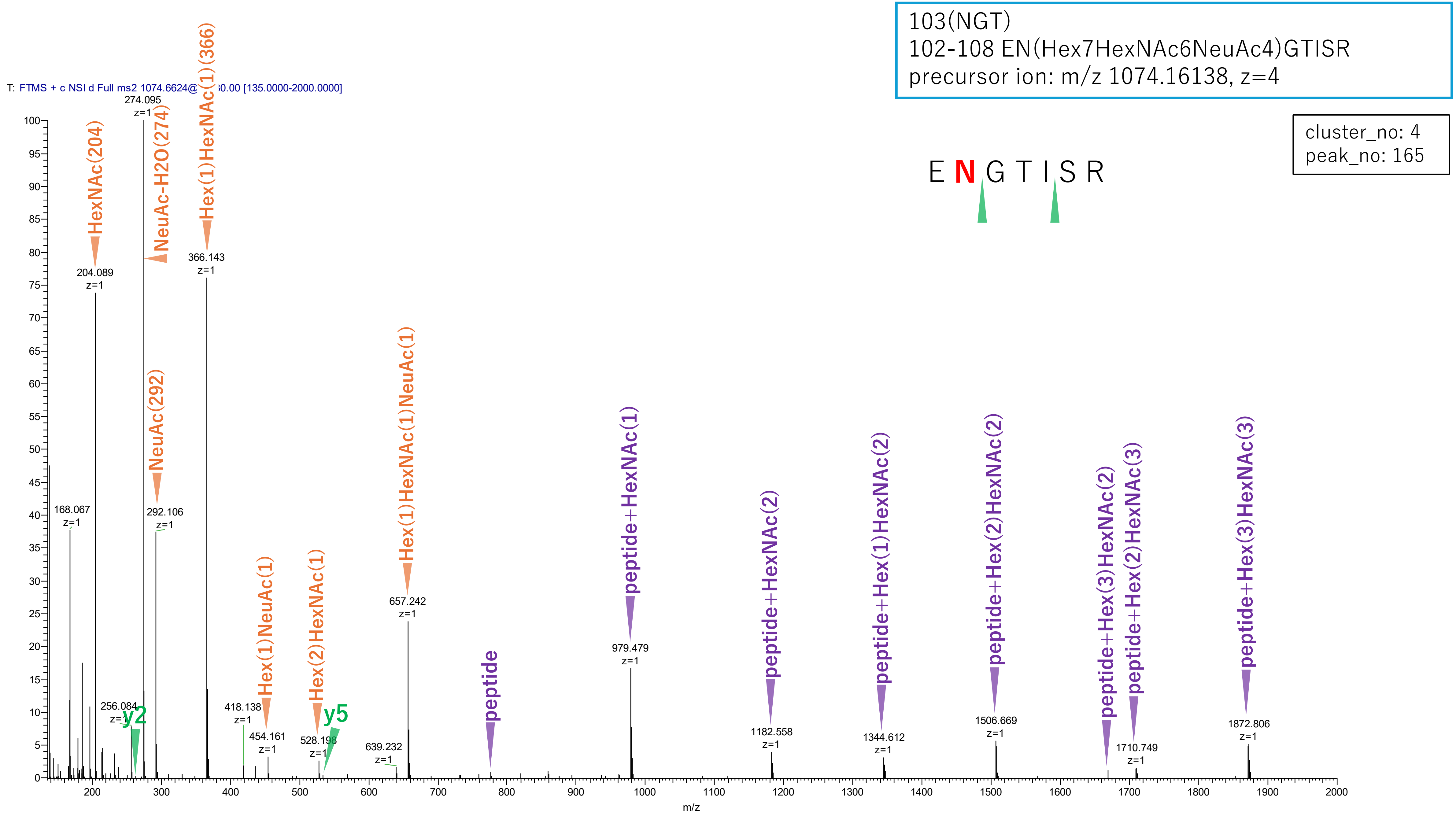

Figure S4-33. MS2 spectra of glycopeptides assigned for hAGP

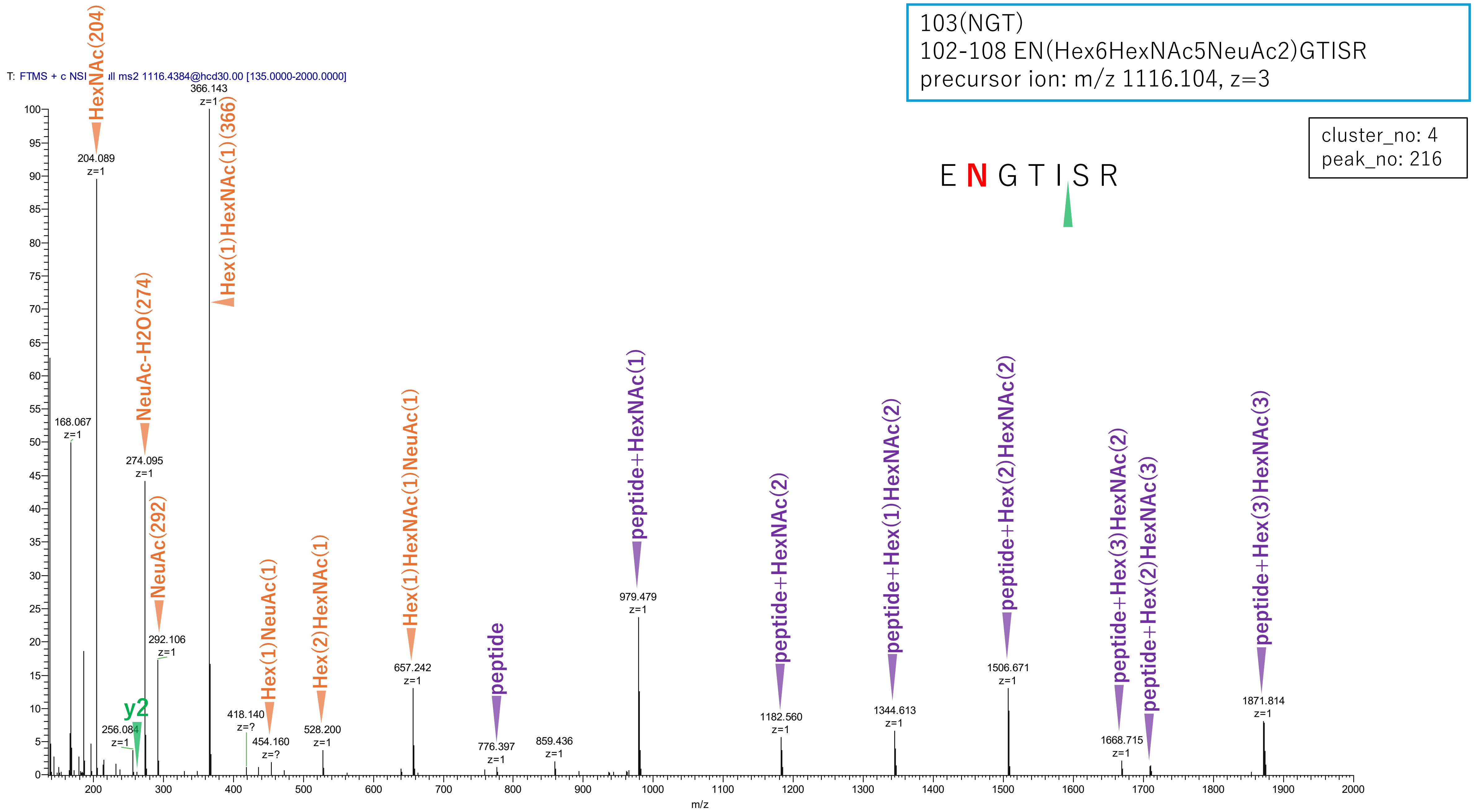

Figure S4-34. MS2 spectra of glycopeptides assigned for hAGP

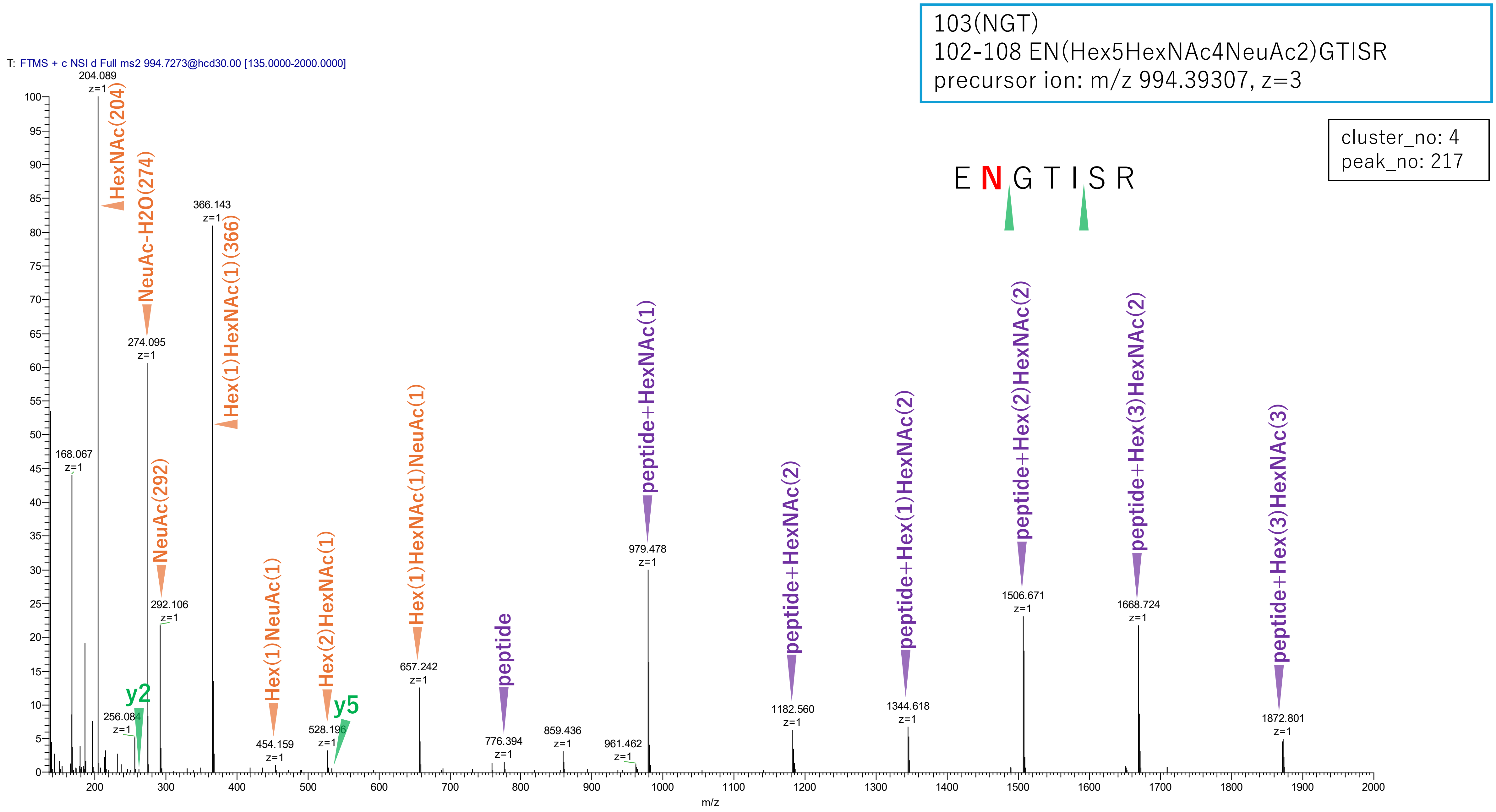

Figure S4-35. MS2 spectra of glycopeptides assigned for hAGP

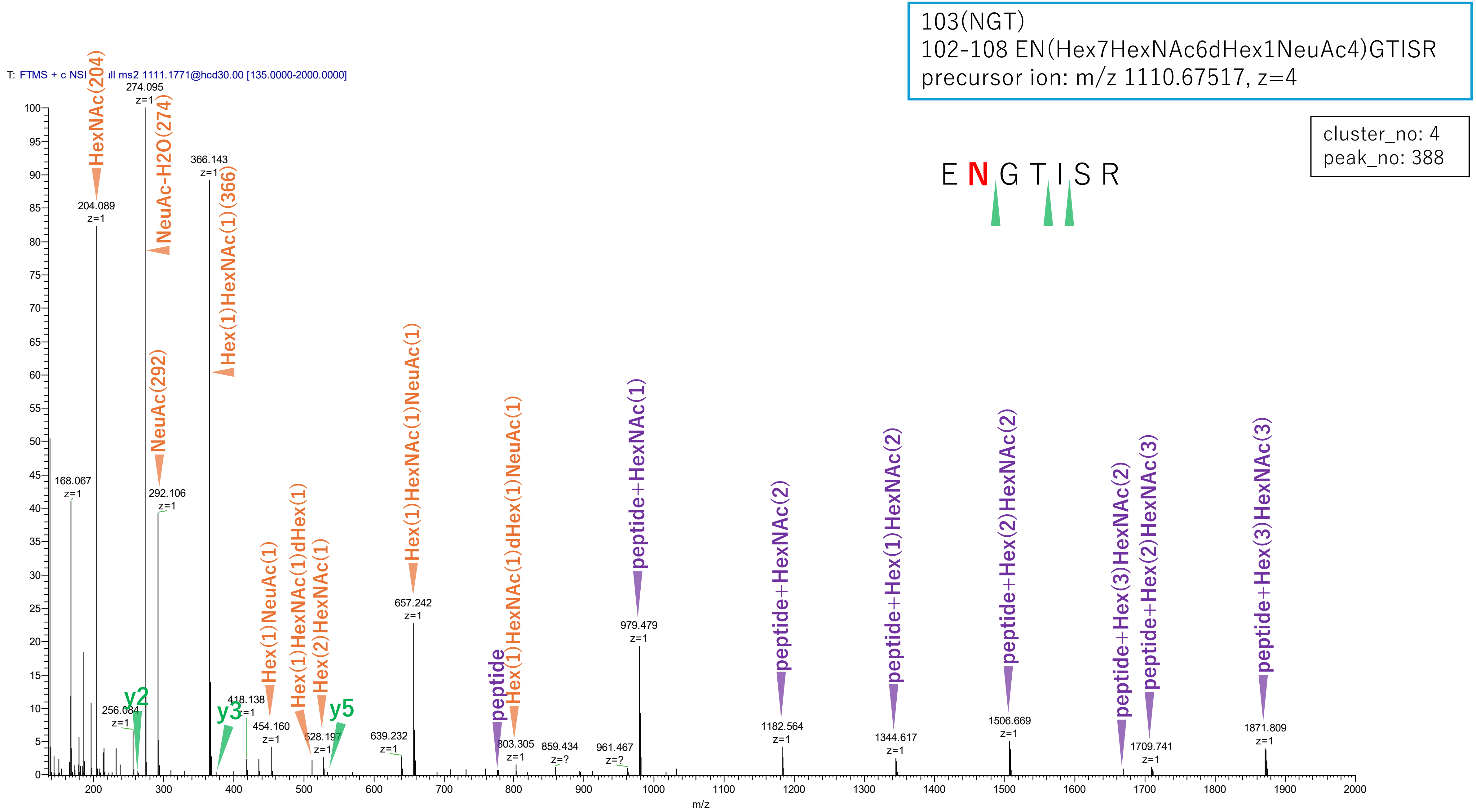

Figure S4-36. MS2 spectra of glycopeptides assigned for hAGP

103(NGT)
102-108 EN(Hex7HexNAc6dHex1NeuAc3)GTISR
precursor ion: m/z 1383.53271, z=3

cluster_no: 4
peak_no: 398

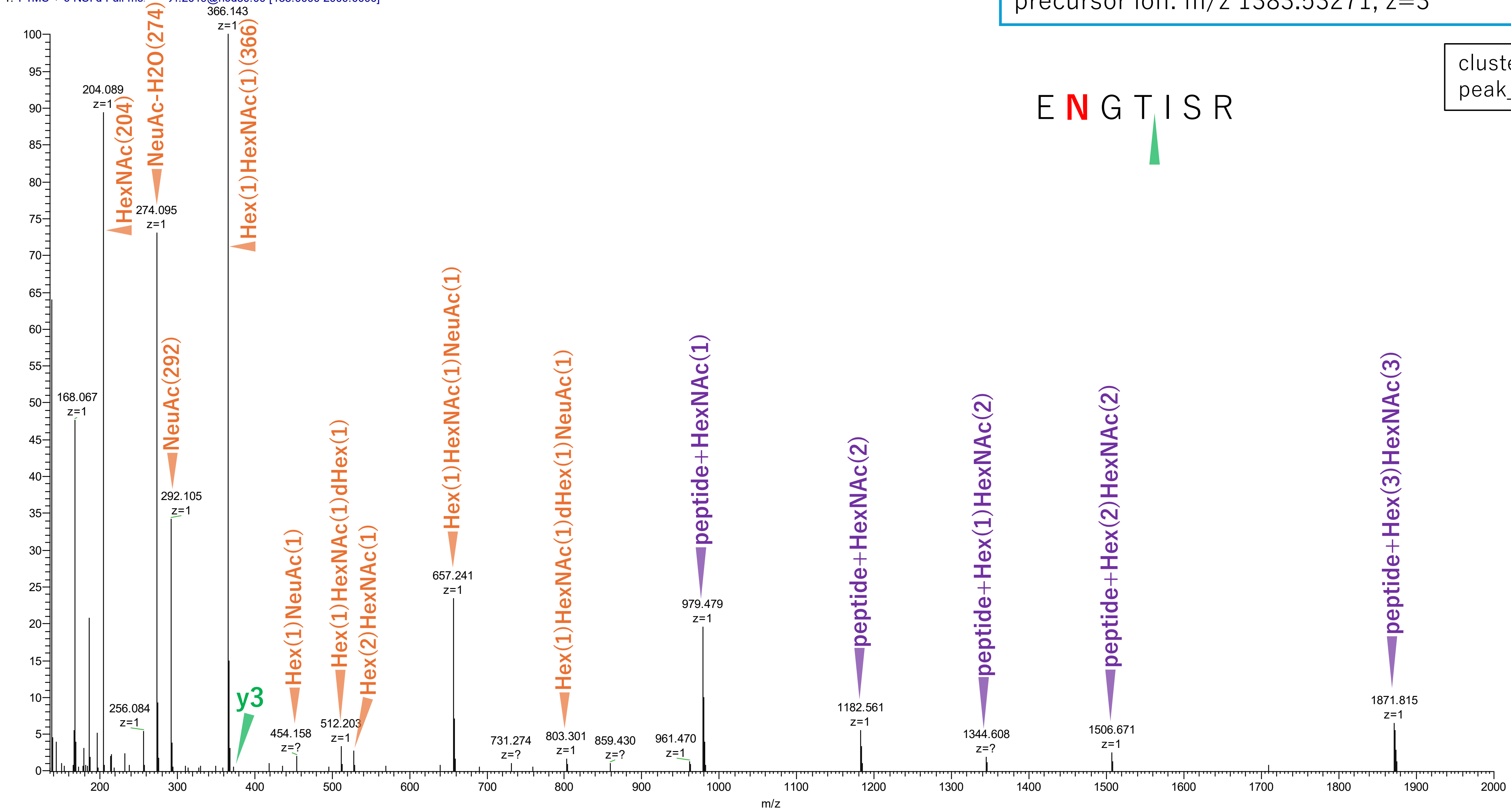

Figure S4-37. MS2 spectra of glycopeptides assigned for hAGP

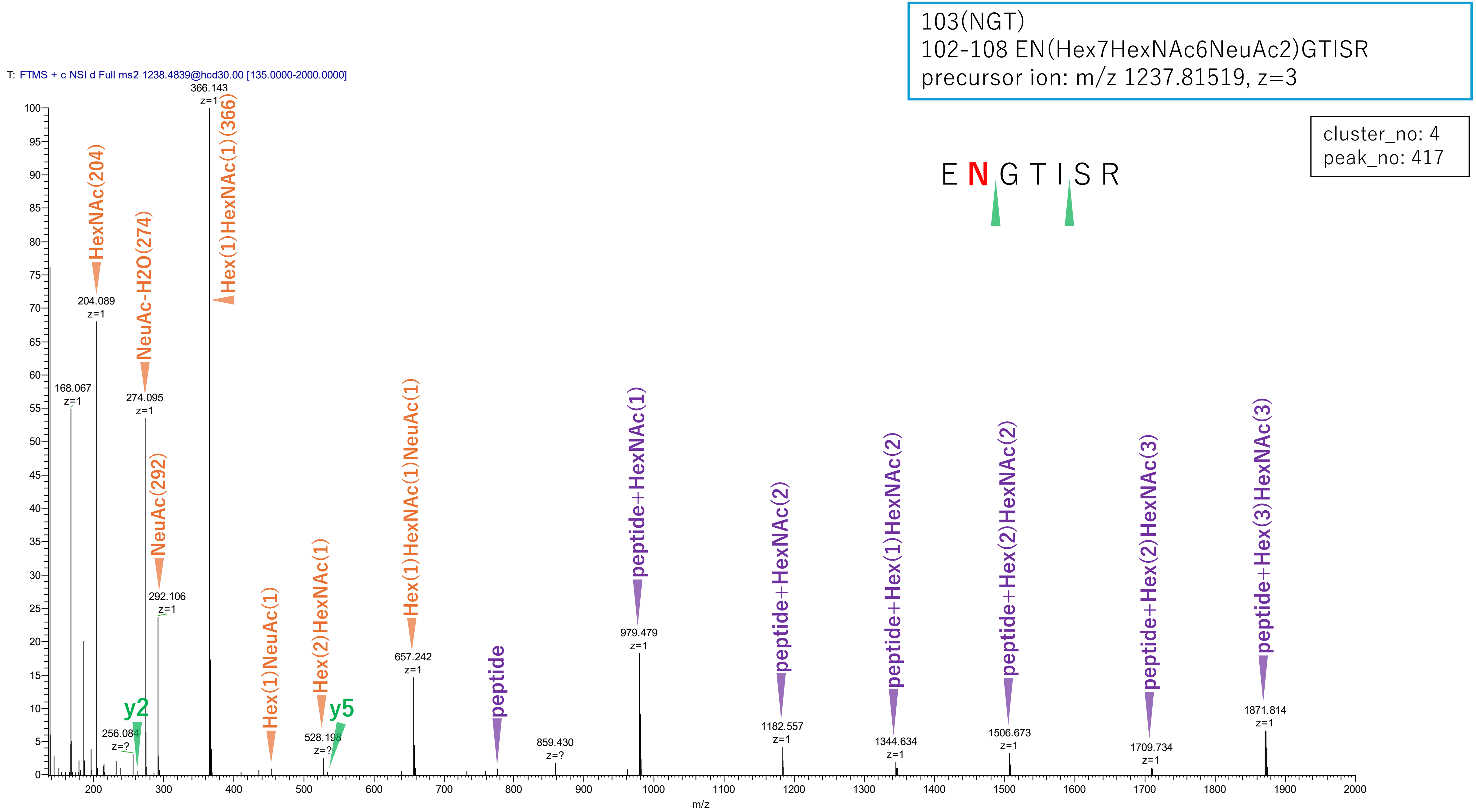

Figure S4-38. MS2 spectra of glycopeptides assigned for hAGP

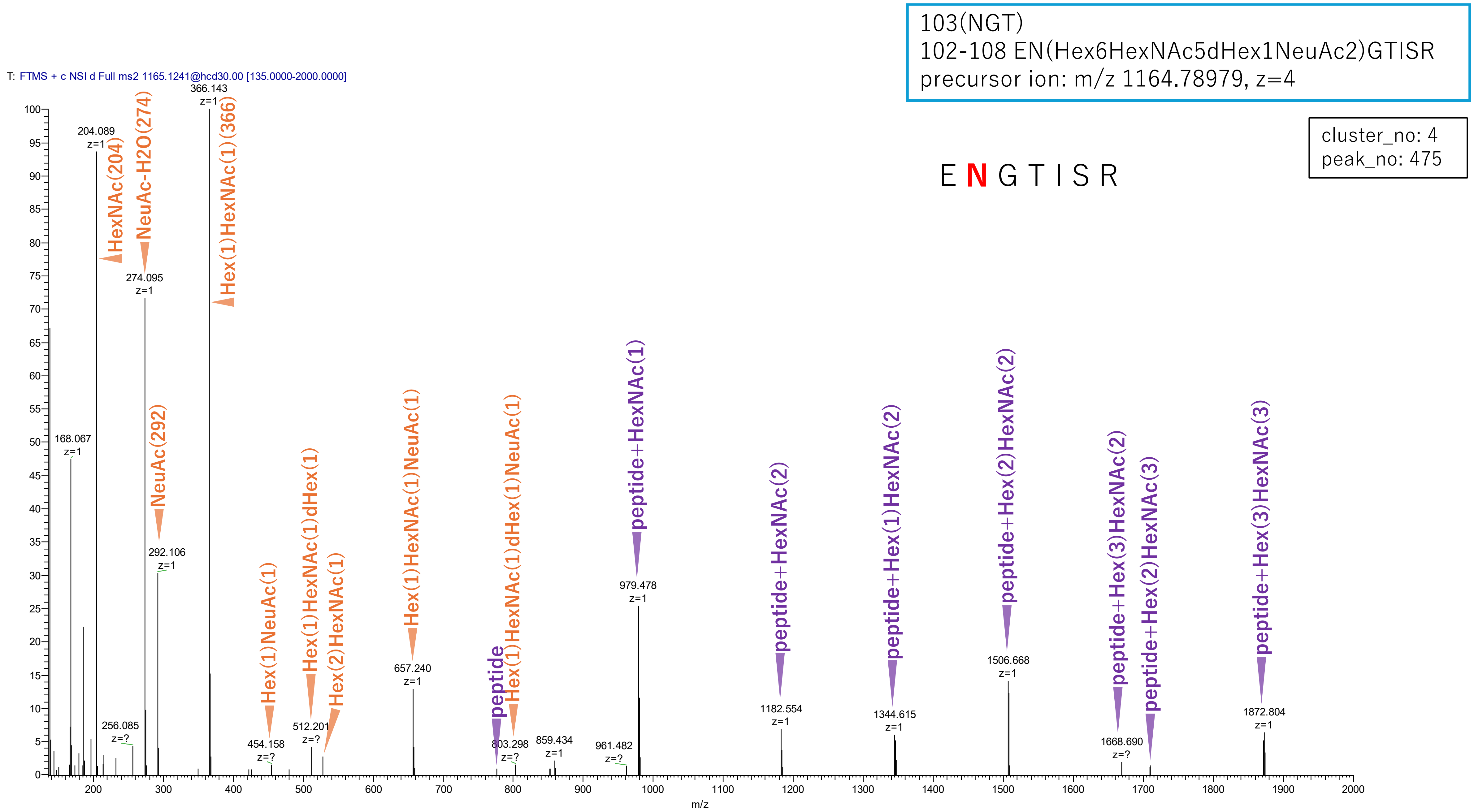

Figure S4-39. MS2 spectra of glycopeptides assigned for hAGP

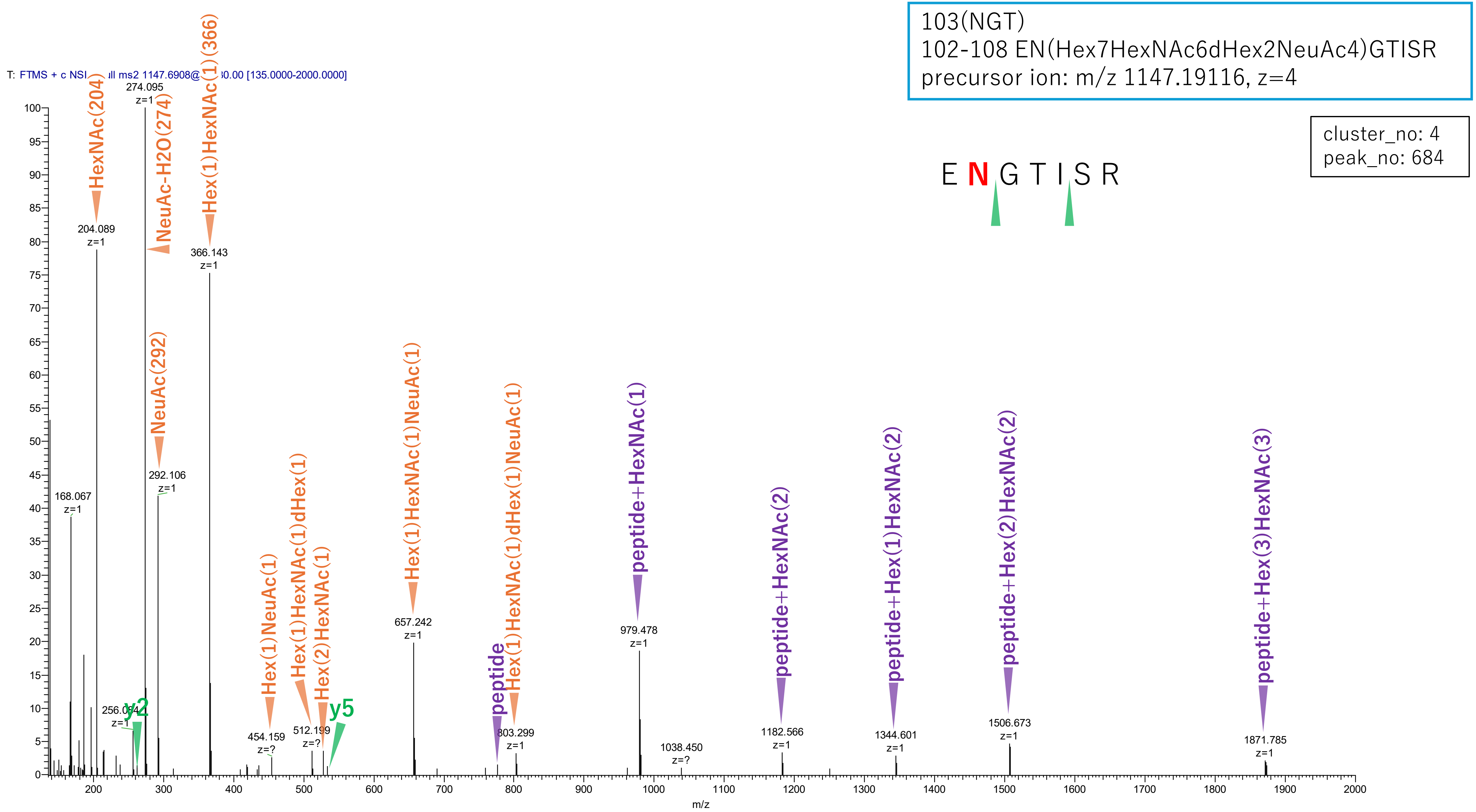

Figure S4-40. MS2 spectra of glycopeptides assigned for hAGP

103(NGT)
102-108 EN(Hex7HexNAc6dHex2NeuAc3)GTISR
precursor ion: m/z 1432.21936, z=3

cluster_no: 4
peak_no: 822

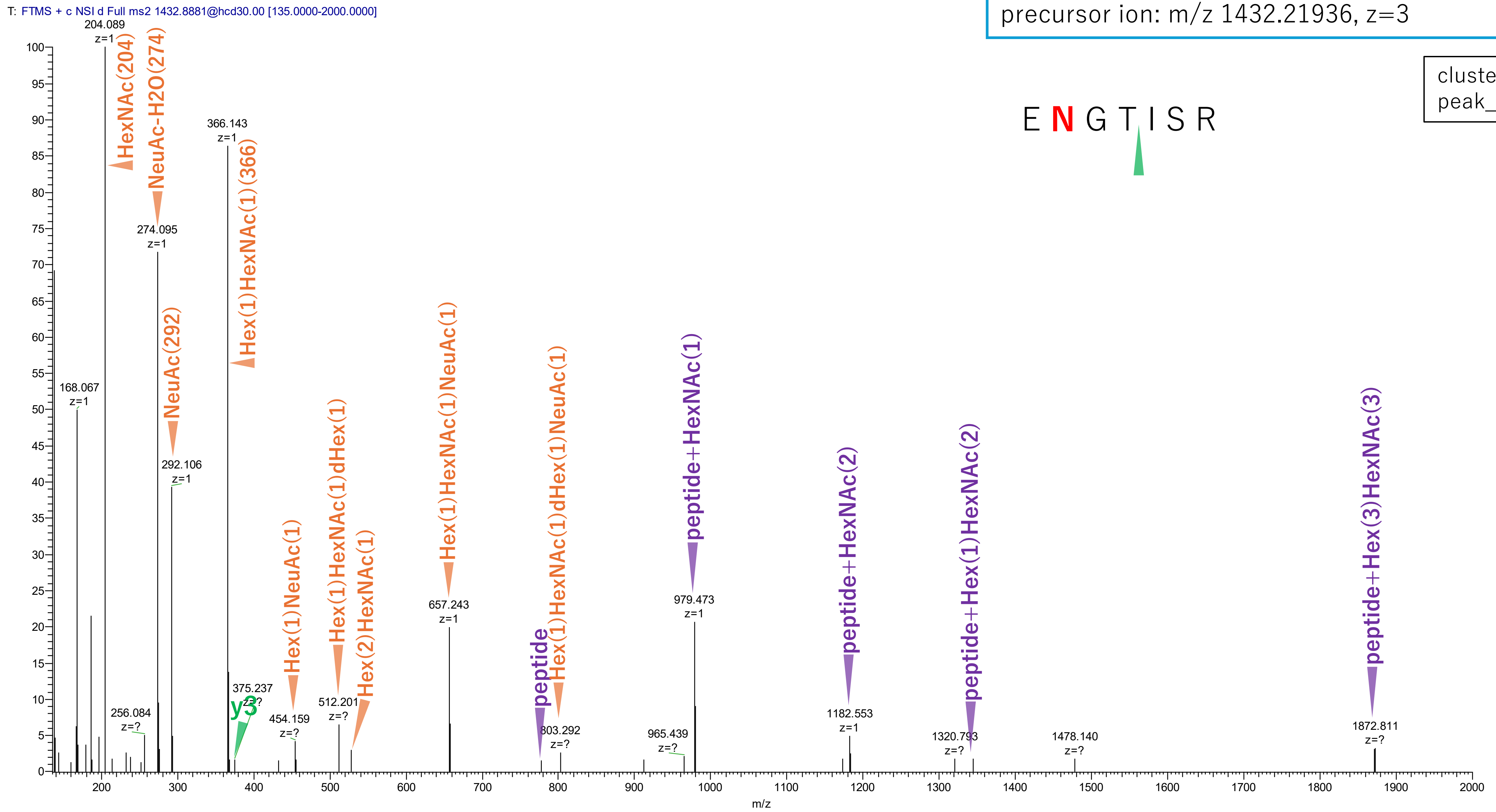

Figure S4-41. MS2 spectra of glycopeptides assigned for hAGP

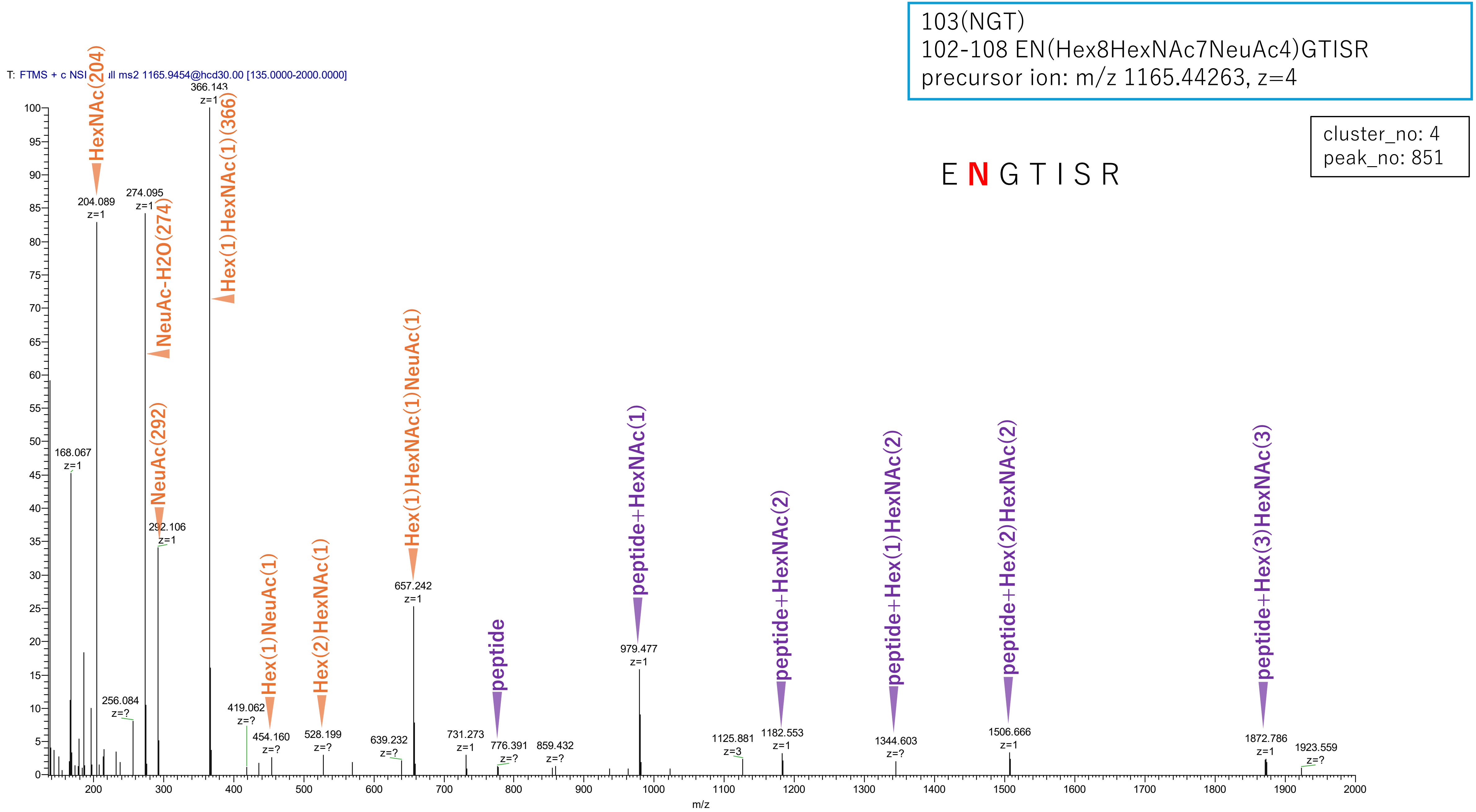

Figure S4-42. MS2 spectra of glycopeptides assigned for hAGP

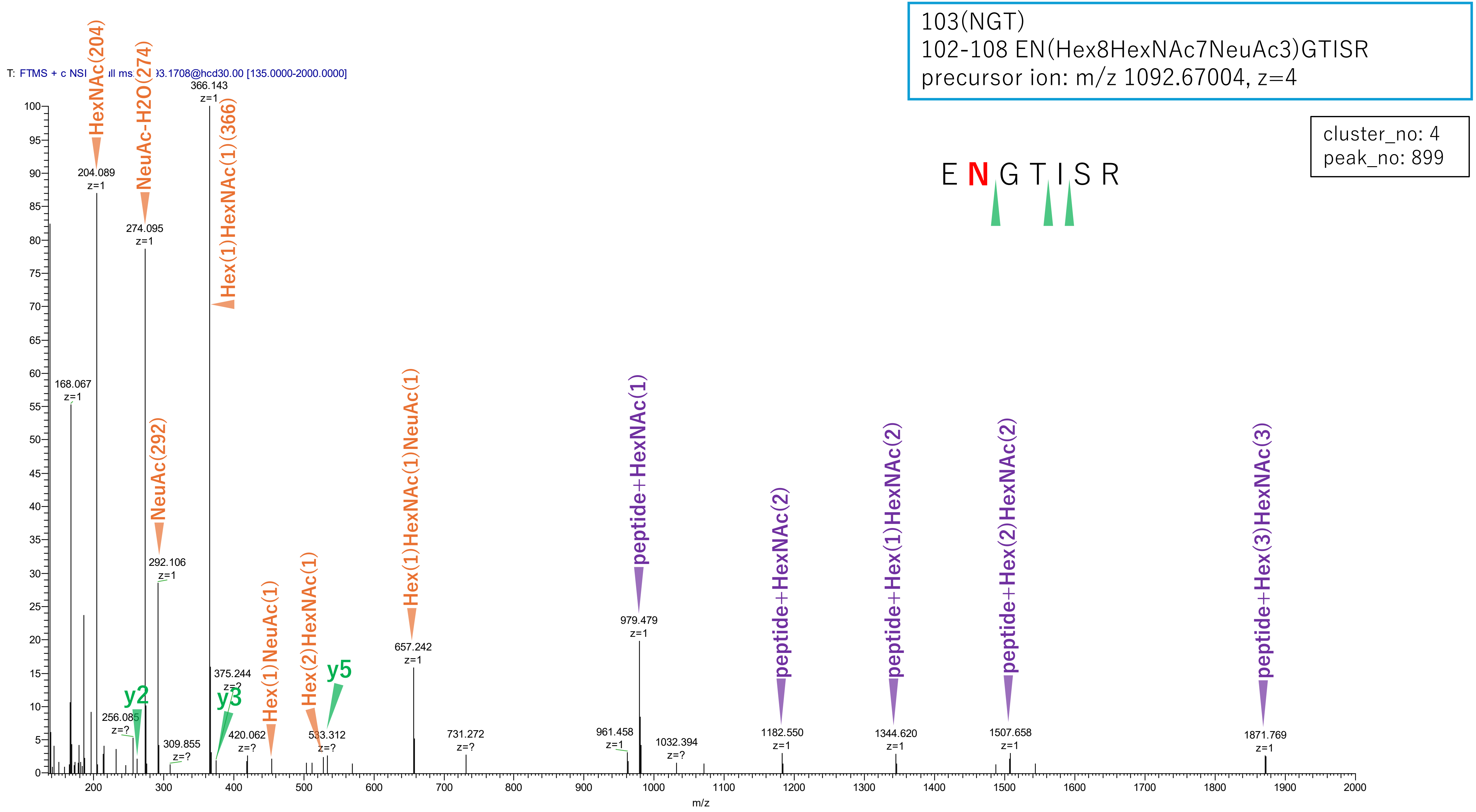

Figure S4-43. MS2 spectra of glycopeptides assigned for hAGP

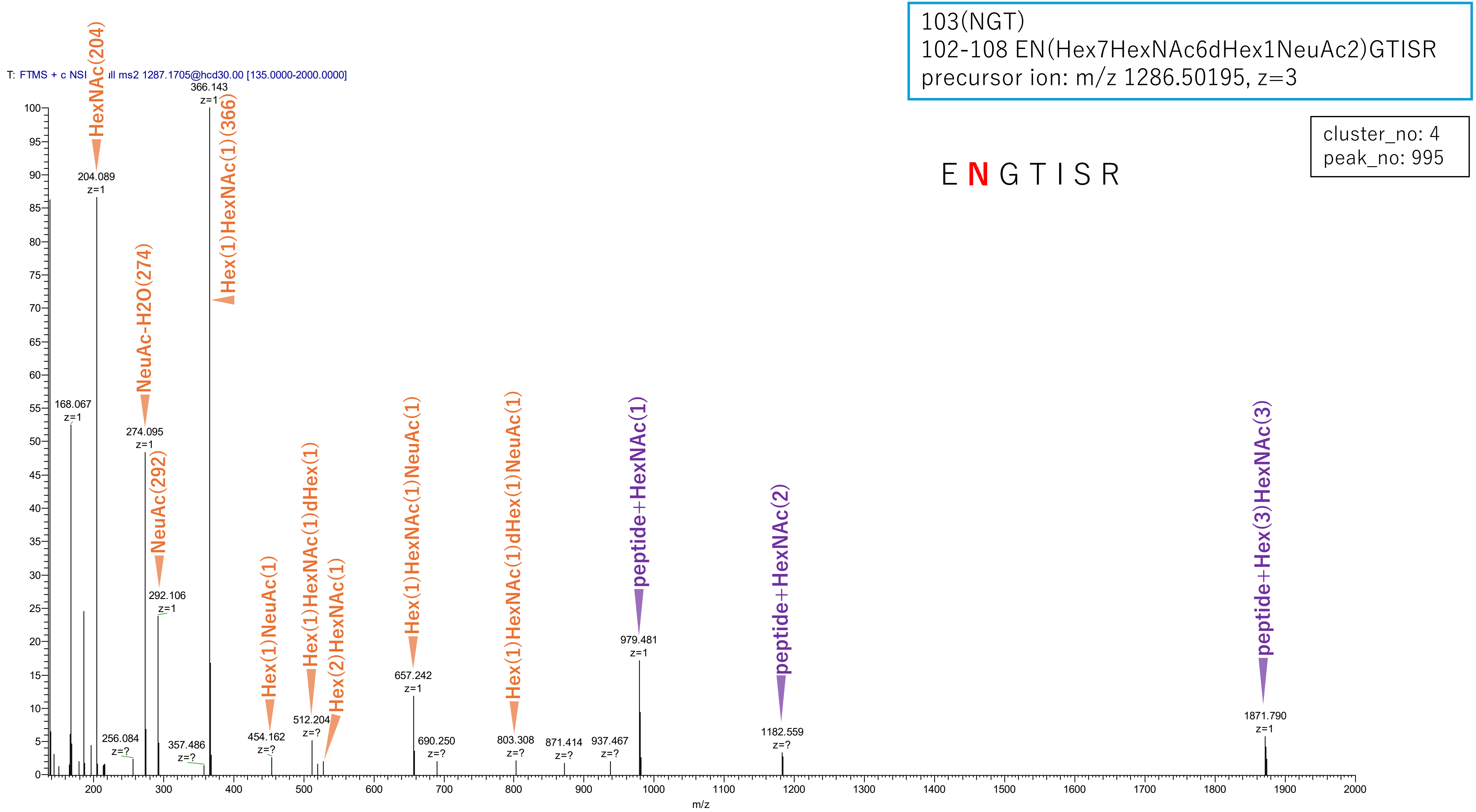

Figure S4-44. MS2 spectra of glycopeptides assigned for hAGP

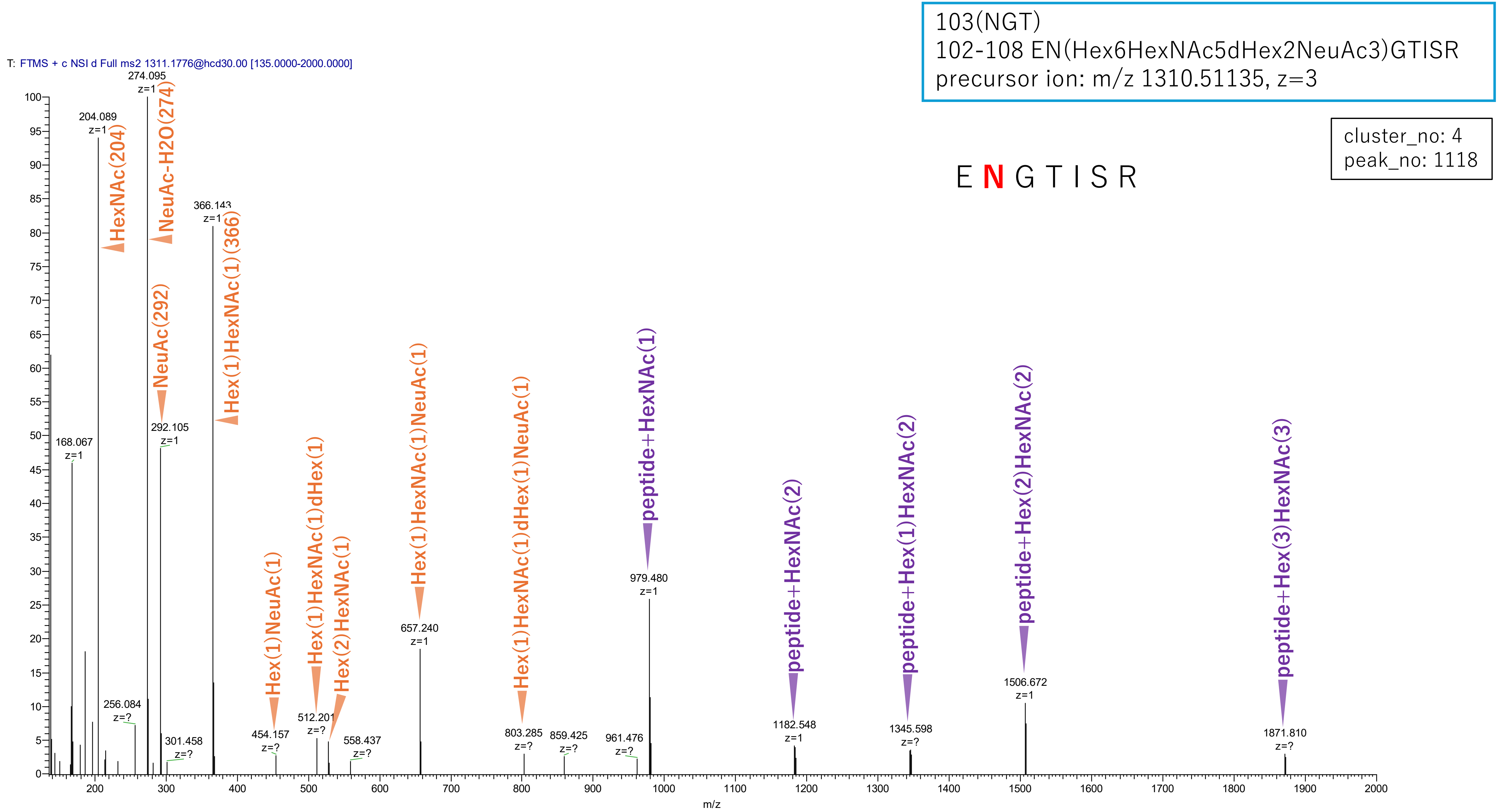

Figure S4-45. MS2 spectra of glycopeptides assigned for hAGP

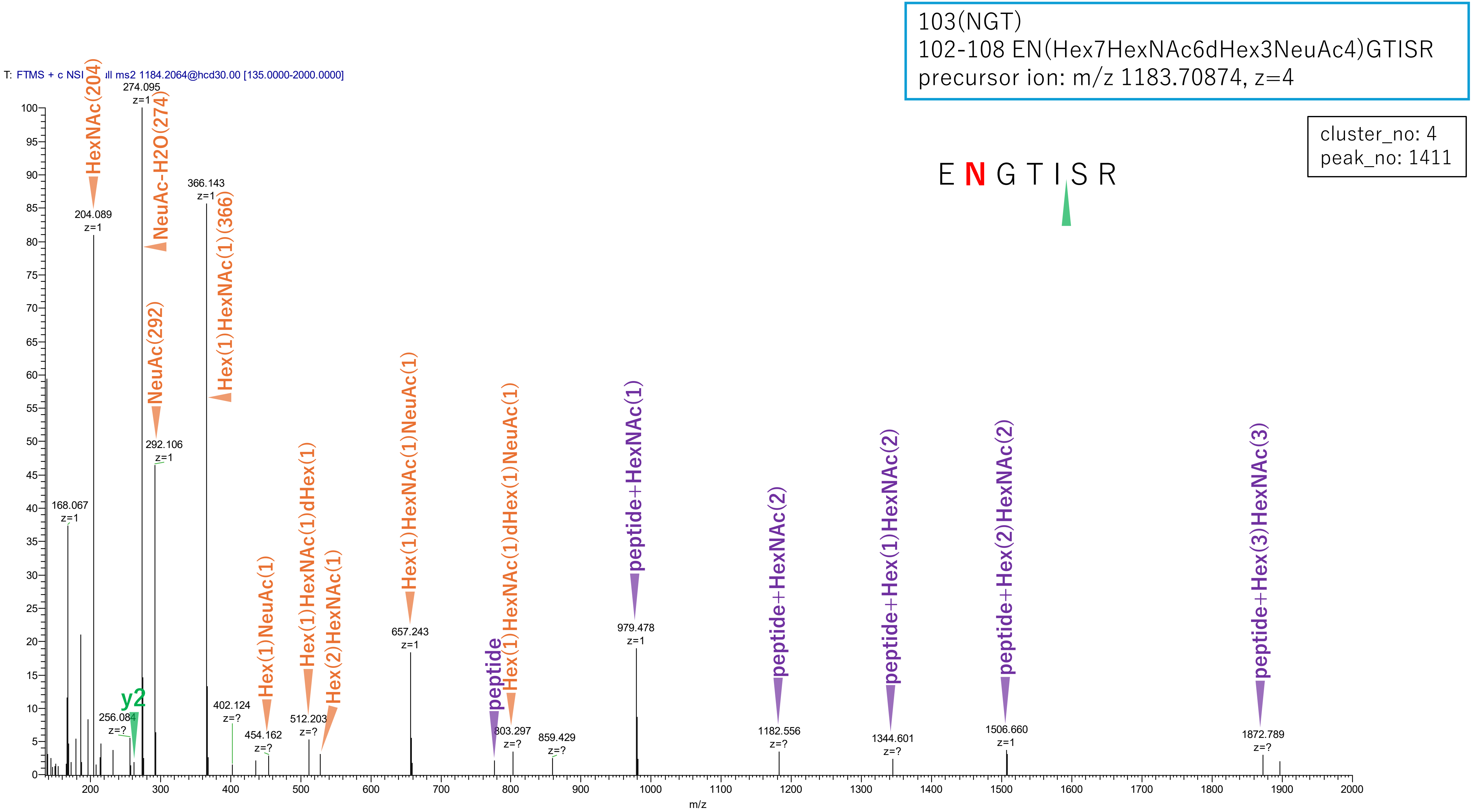

Figure S4-46. MS2 spectra of glycopeptides assigned for hAGP

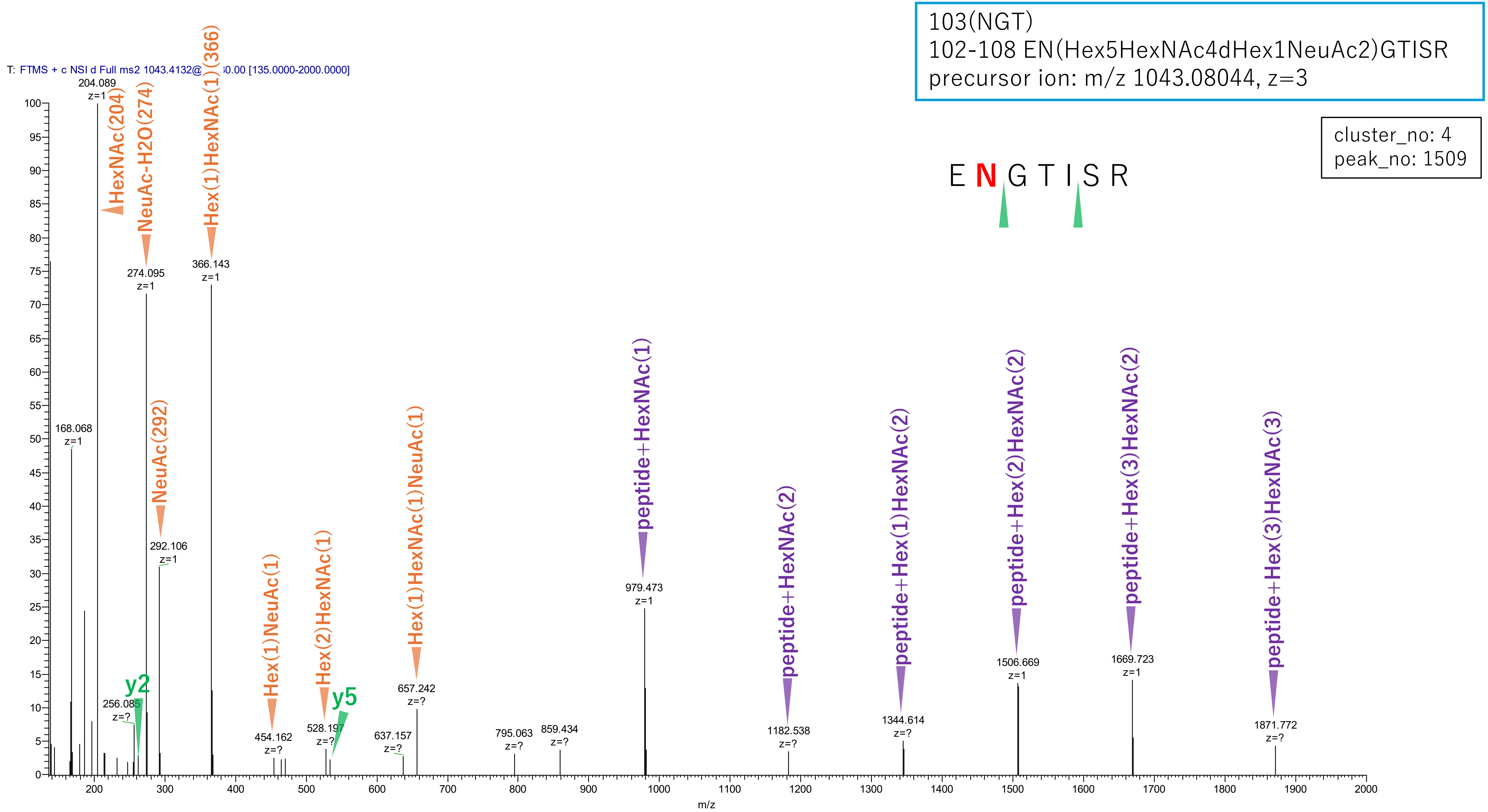

Figure S4-47. MS2 spectra of glycopeptides assigned for hAGP

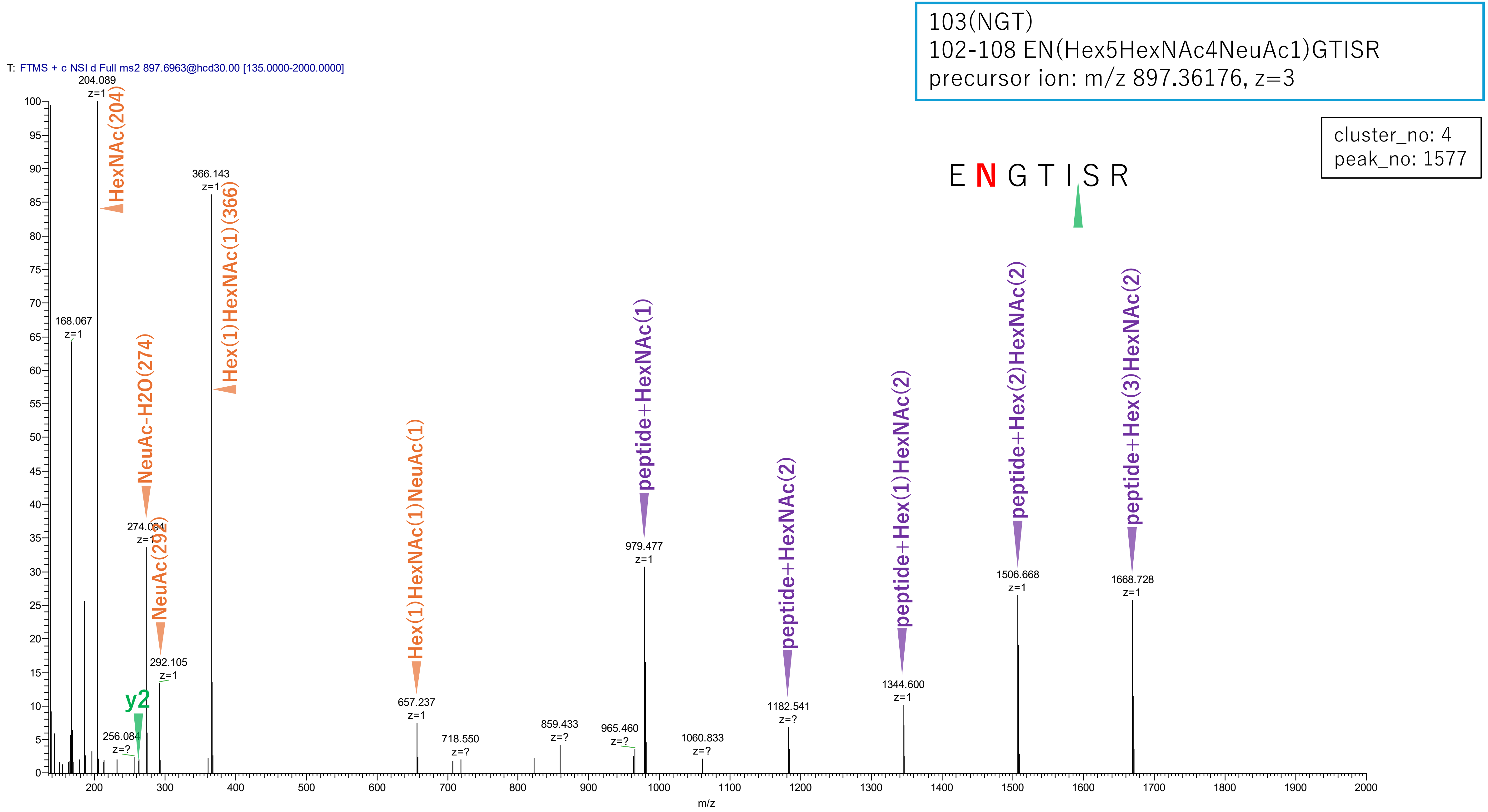

Figure S4-48. MS2 spectra of glycopeptides assigned for hAGP

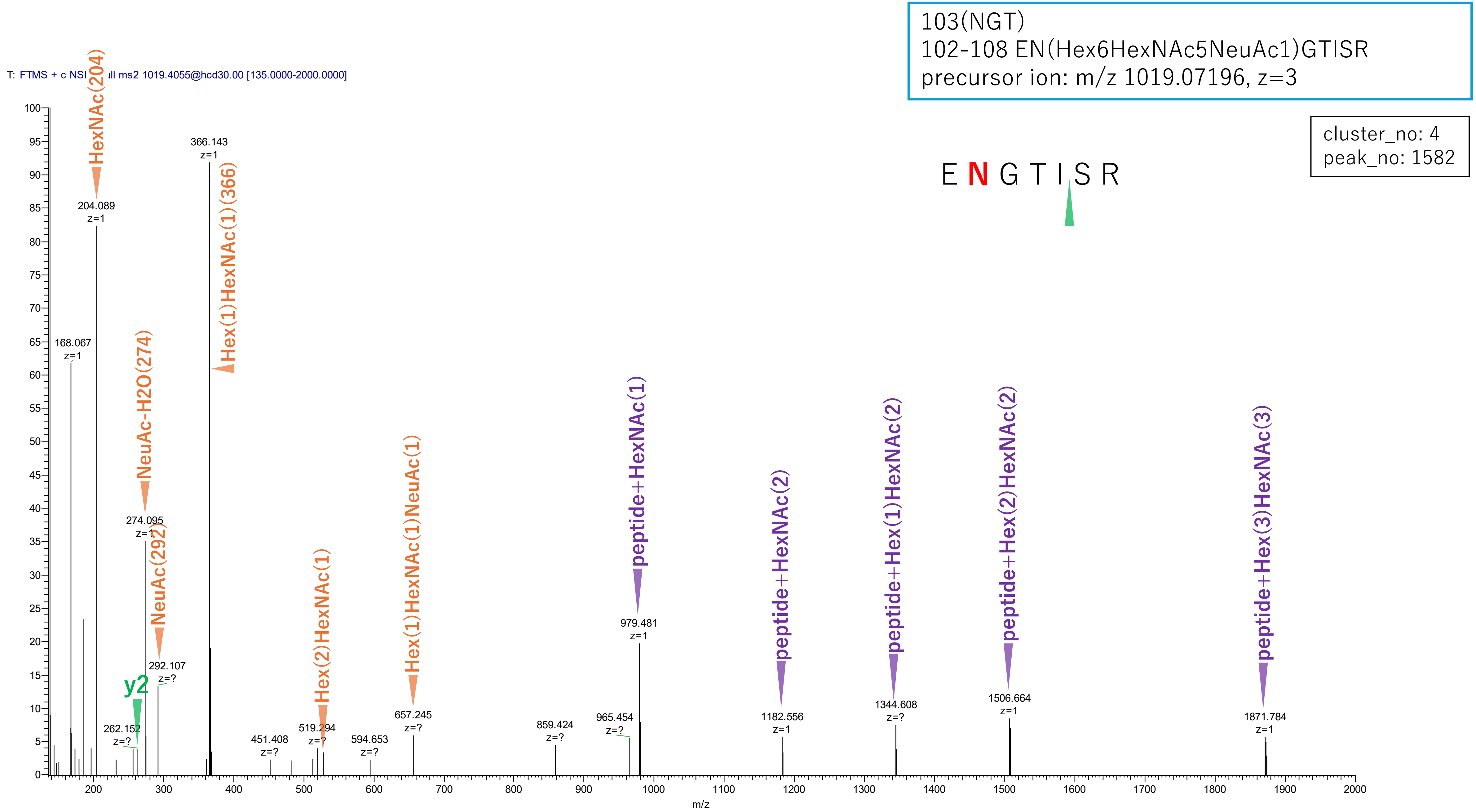

Figure S4-49. MS2 spectra of glycopeptides assigned for hAGP

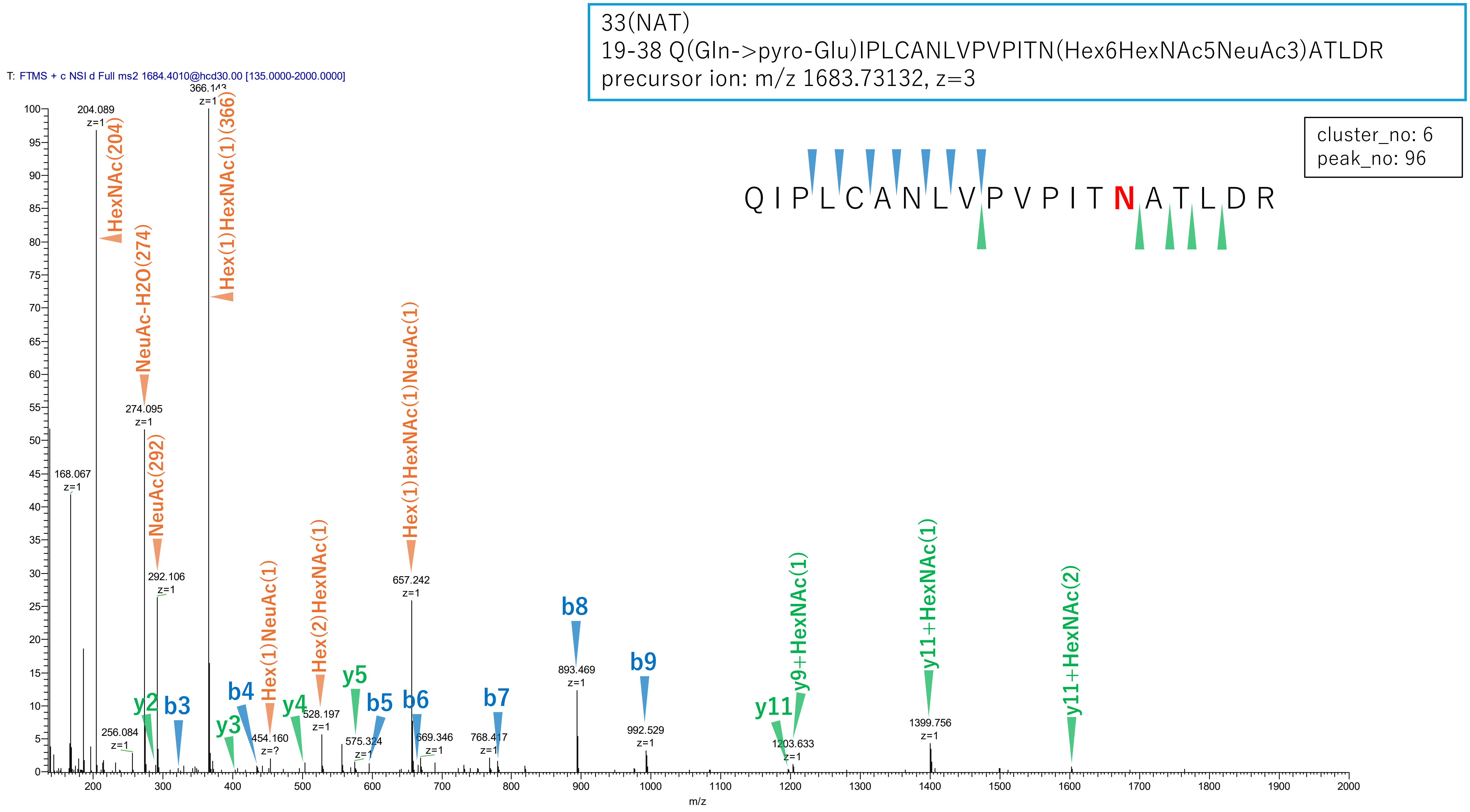

Figure S4-50. MS2 spectra of glycopeptides assigned for hAGP

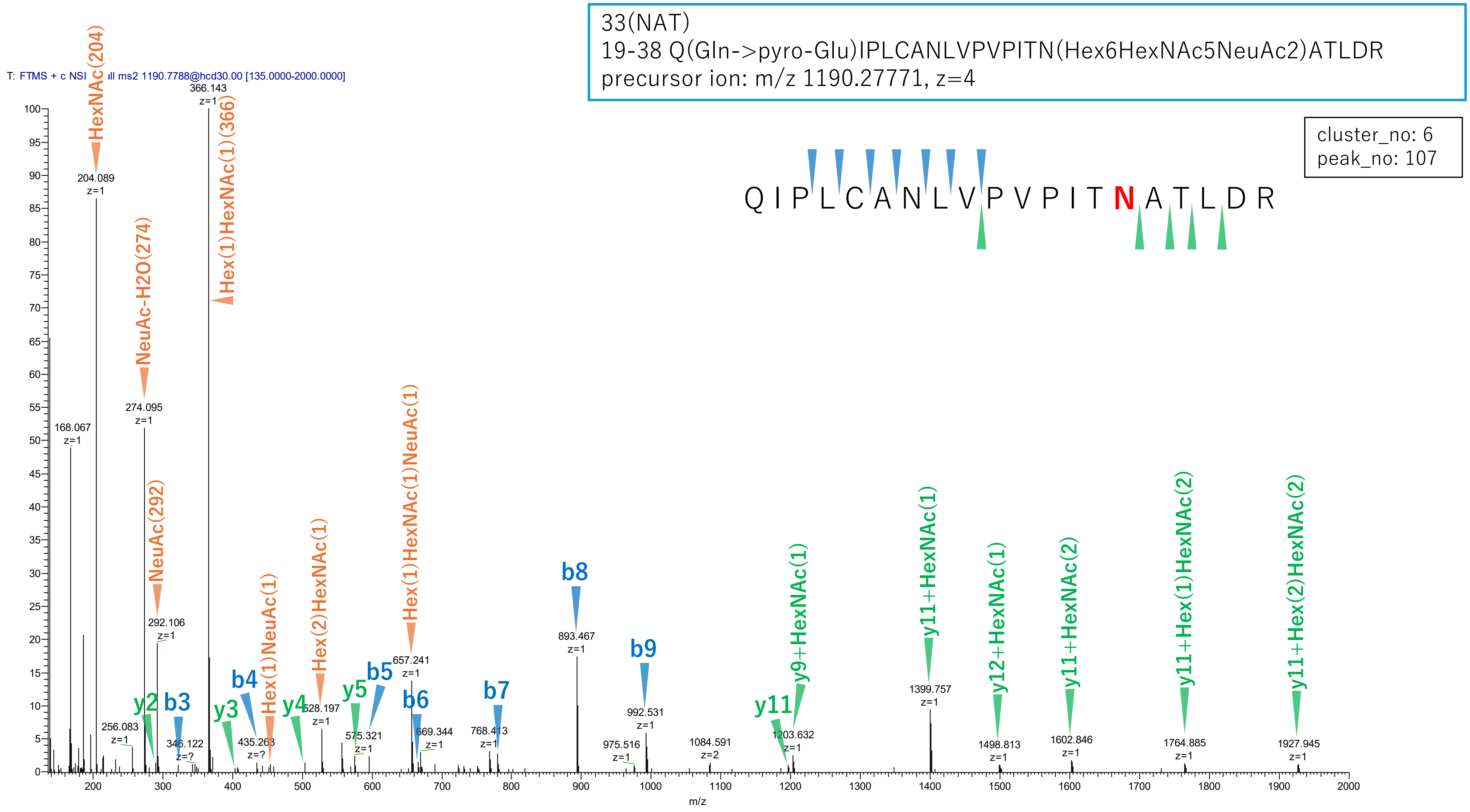

Figure S4-51. MS2 spectra of glycopeptides assigned for hAGP

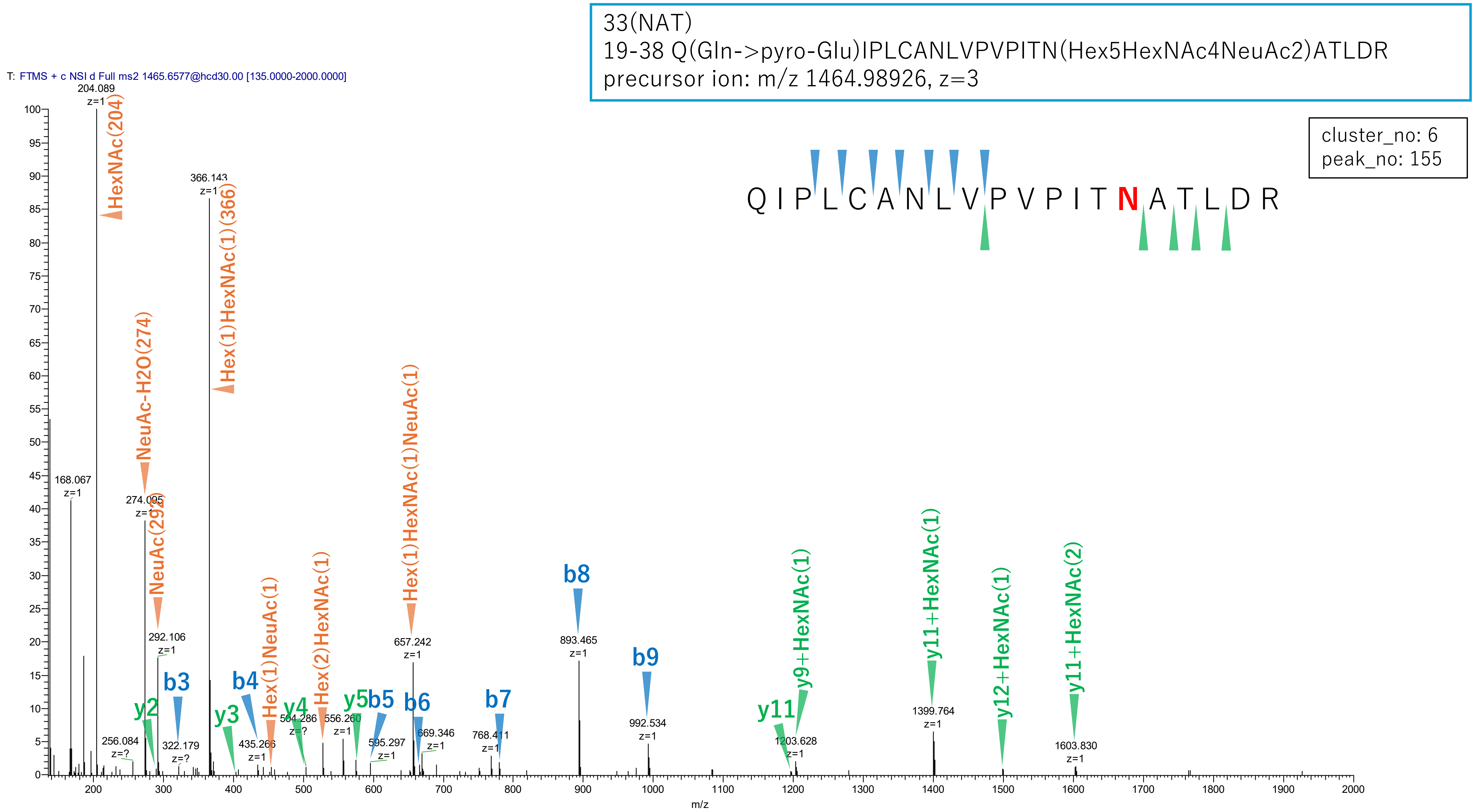

Figure S4-52. MS2 spectra of glycopeptides assigned for hAGP

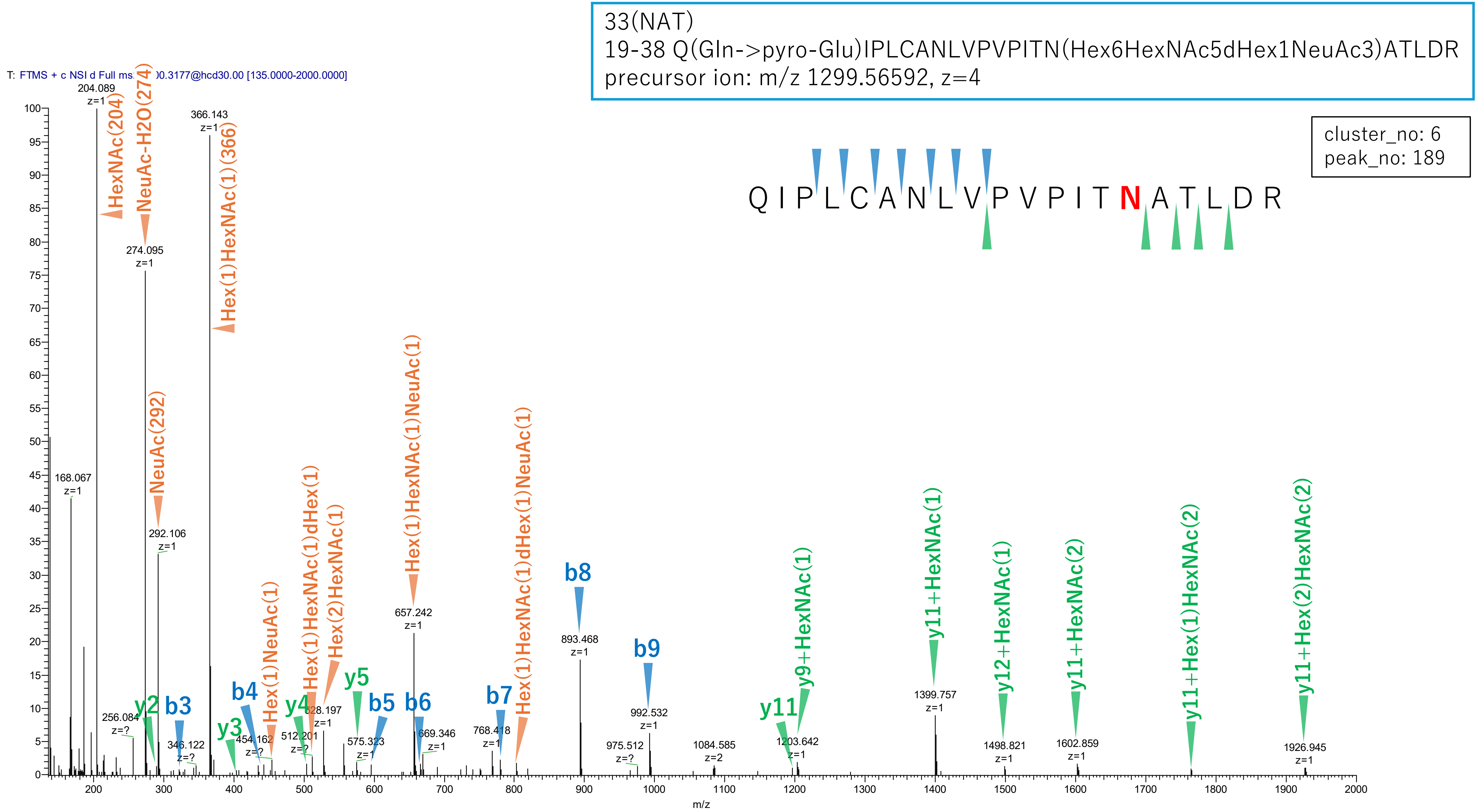

Figure S4-53. MS2 spectra of glycopeptides assigned for hAGP

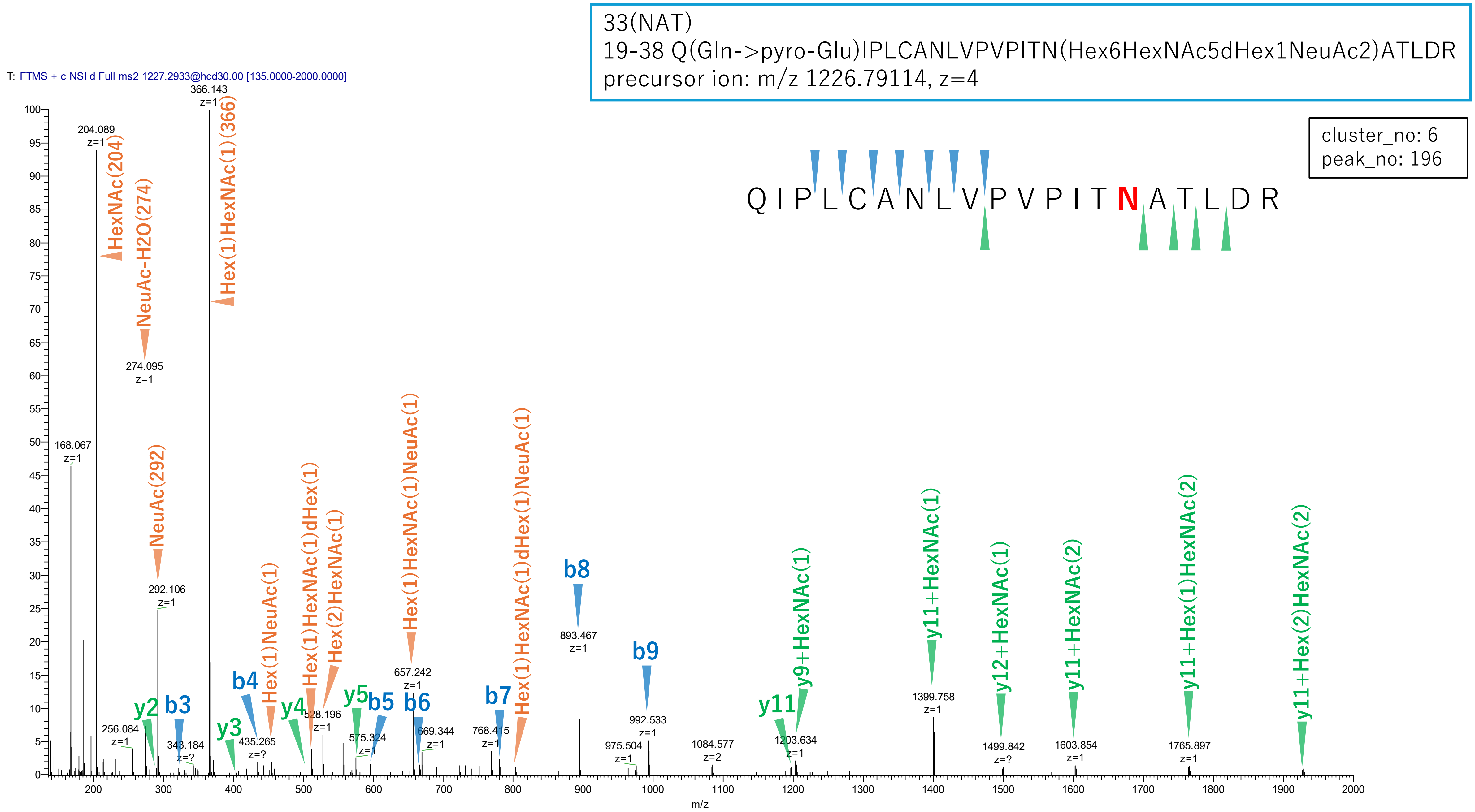

Figure S4-54. MS2 spectra of glycopeptides assigned for hAGP

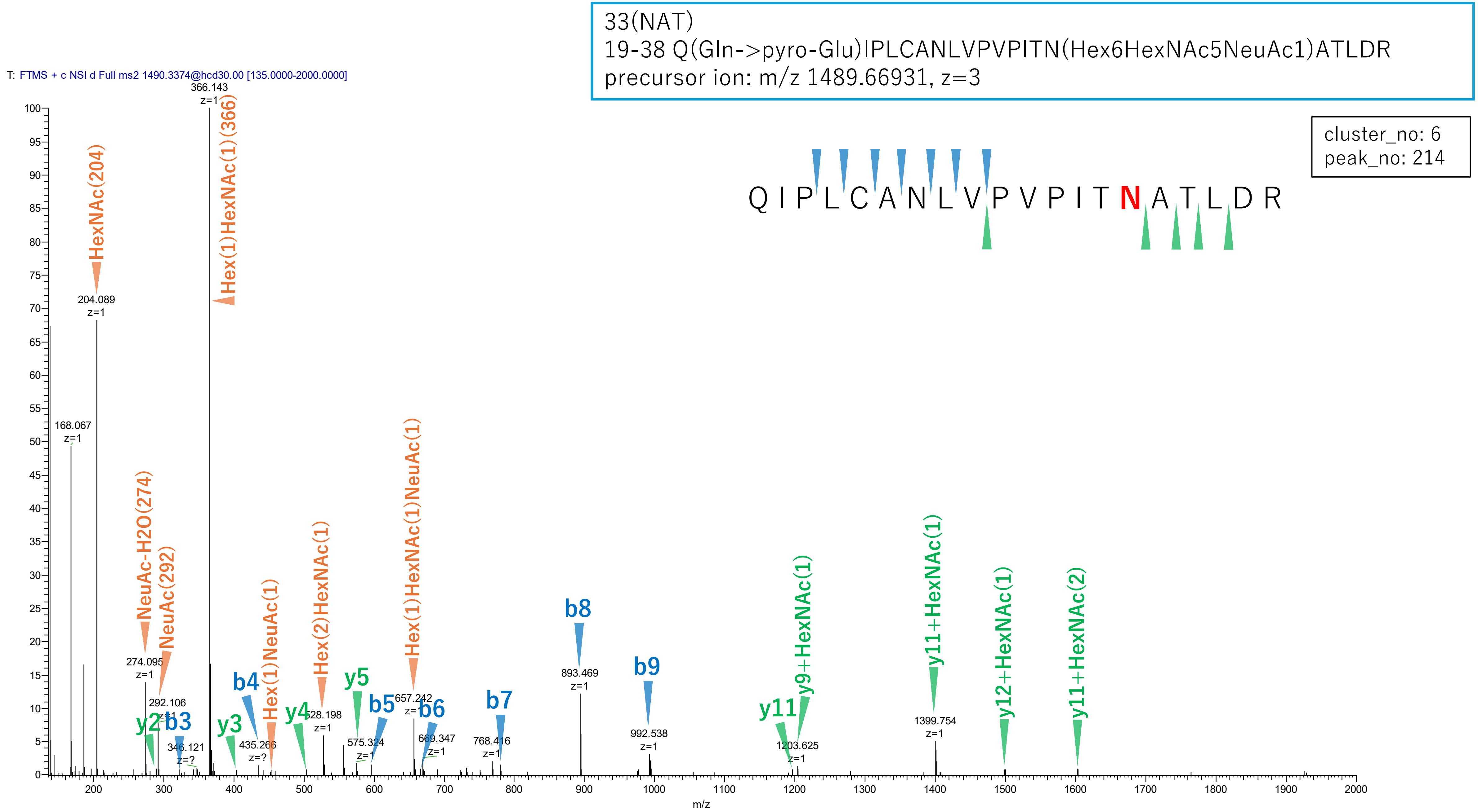

Figure S4-55. MS2 spectra of glycopeptides assigned for hAGP

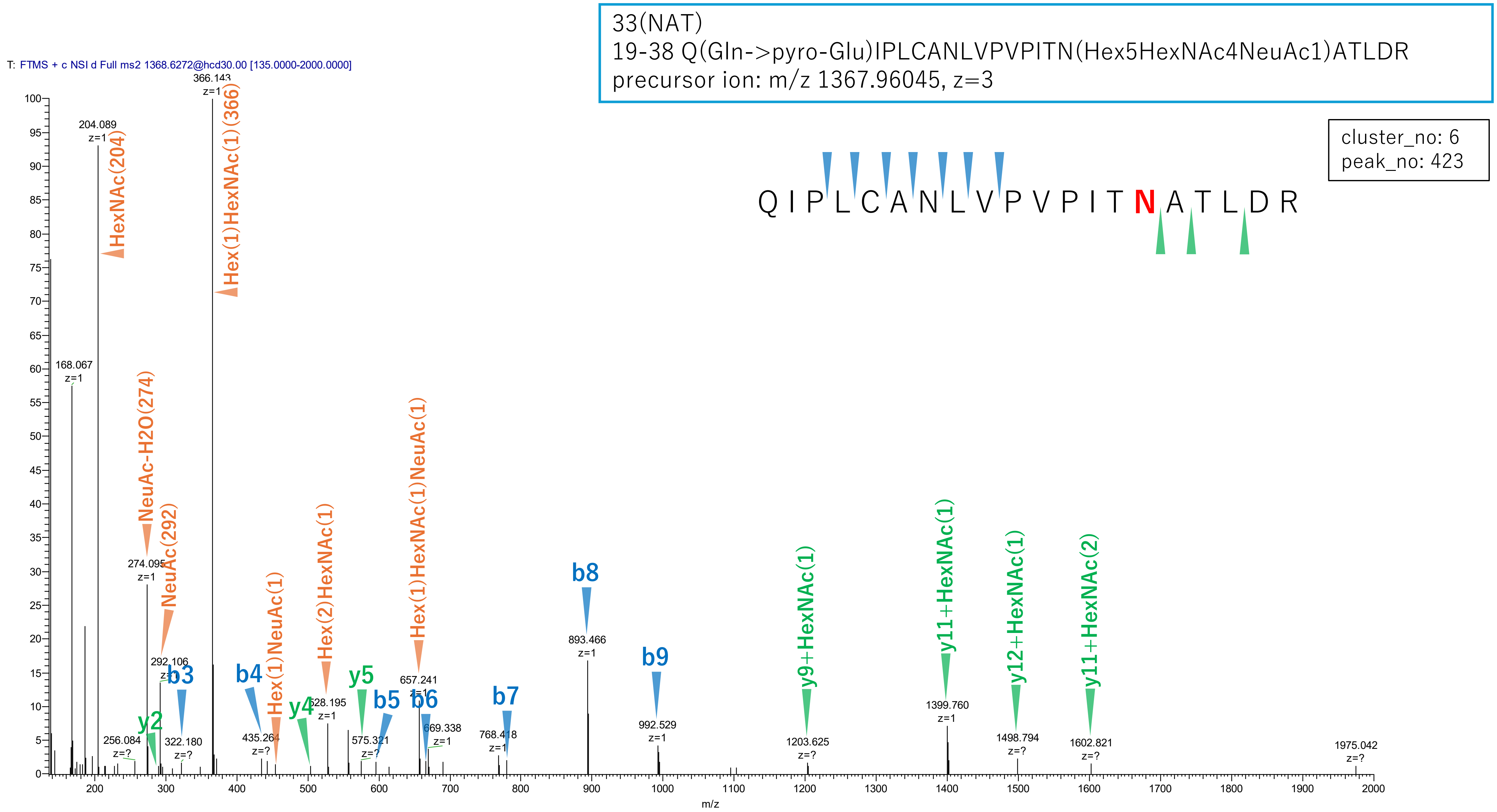

Figure S4-56. MS2 spectra of glycopeptides assigned for hAGP

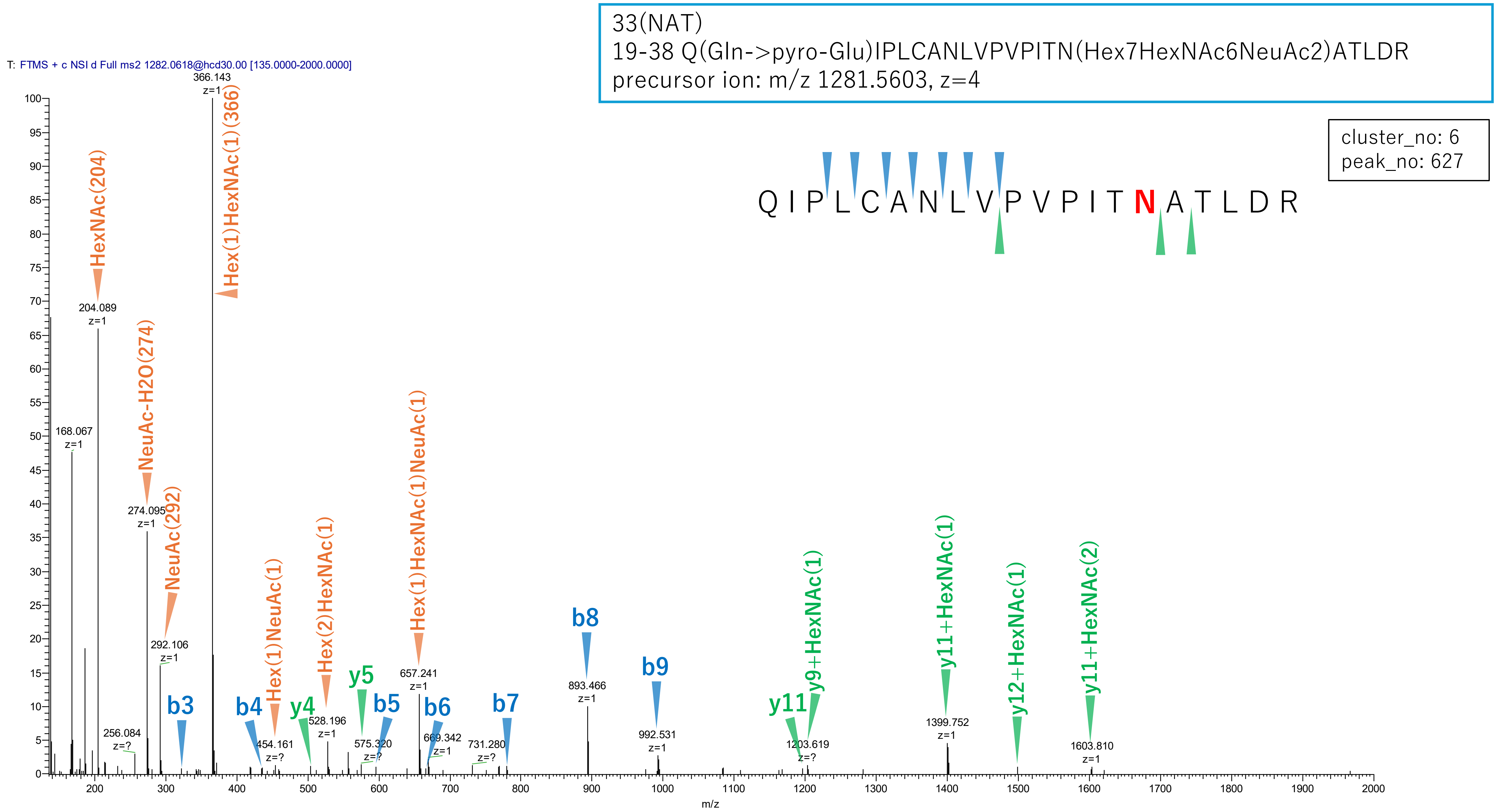

Figure S4-57. MS2 spectra of glycopeptides assigned for hAGP

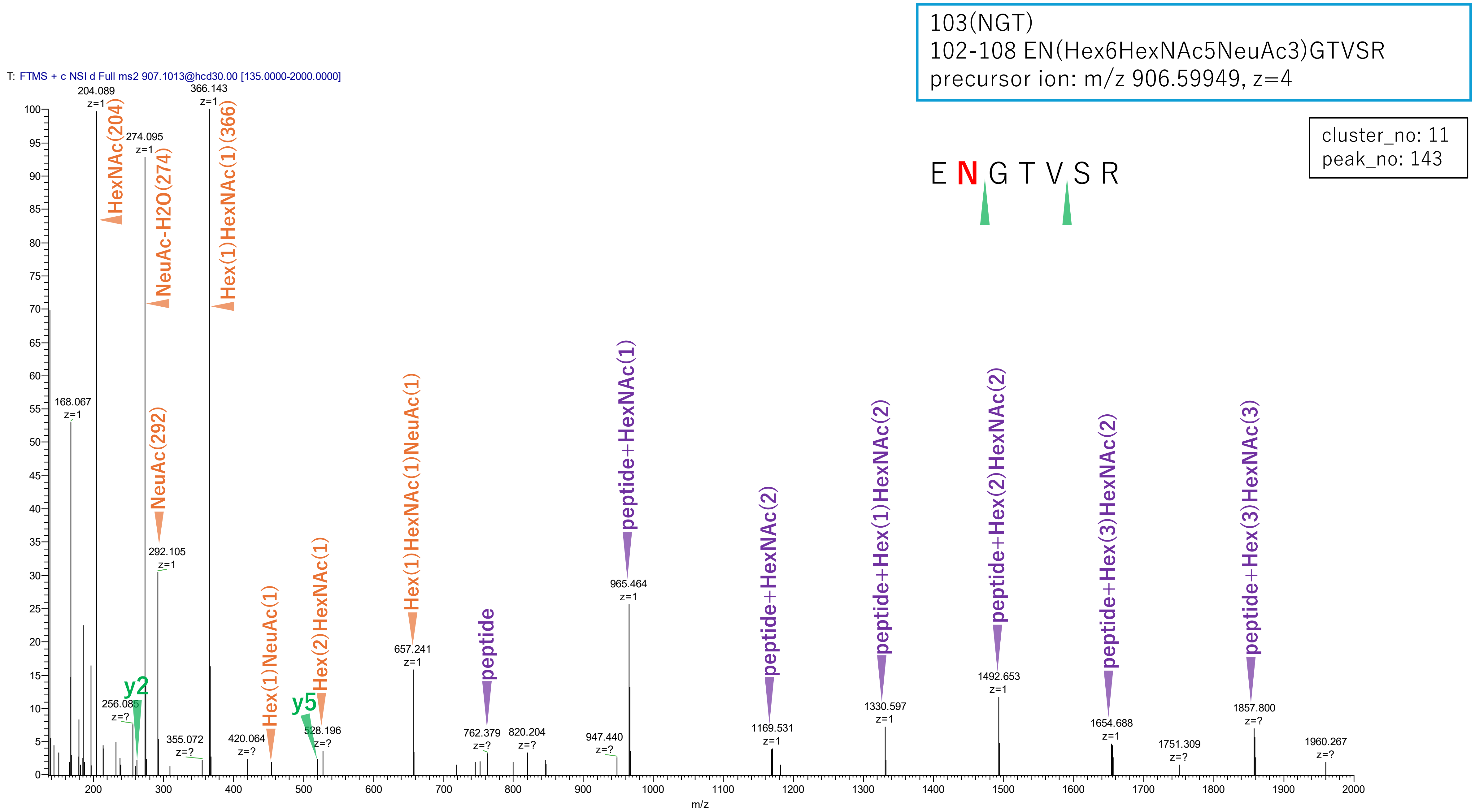

Figure S4-58. MS2 spectra of glycopeptides assigned for hAGP

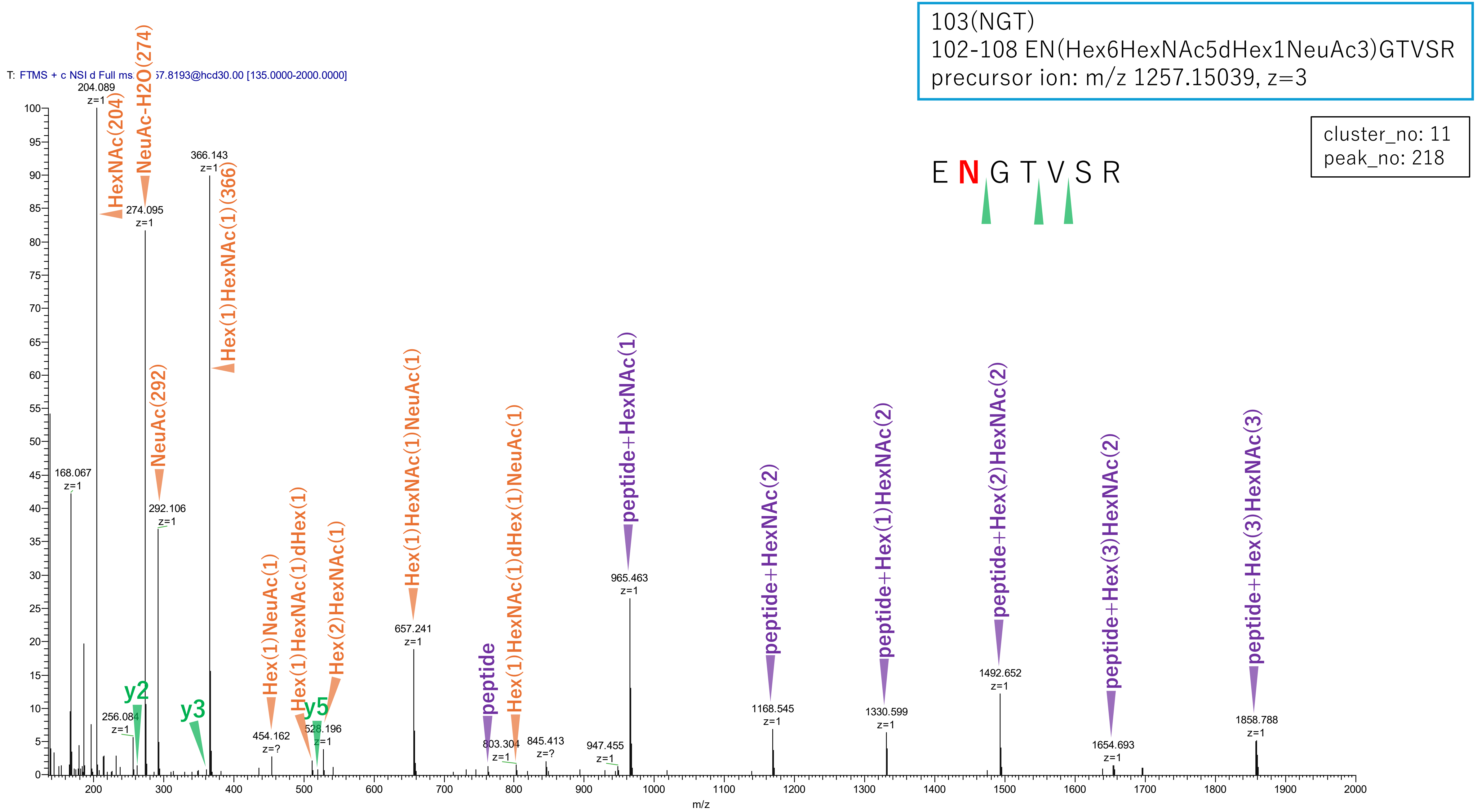

Figure S4-59. MS2 spectra of glycopeptides assigned for hAGP

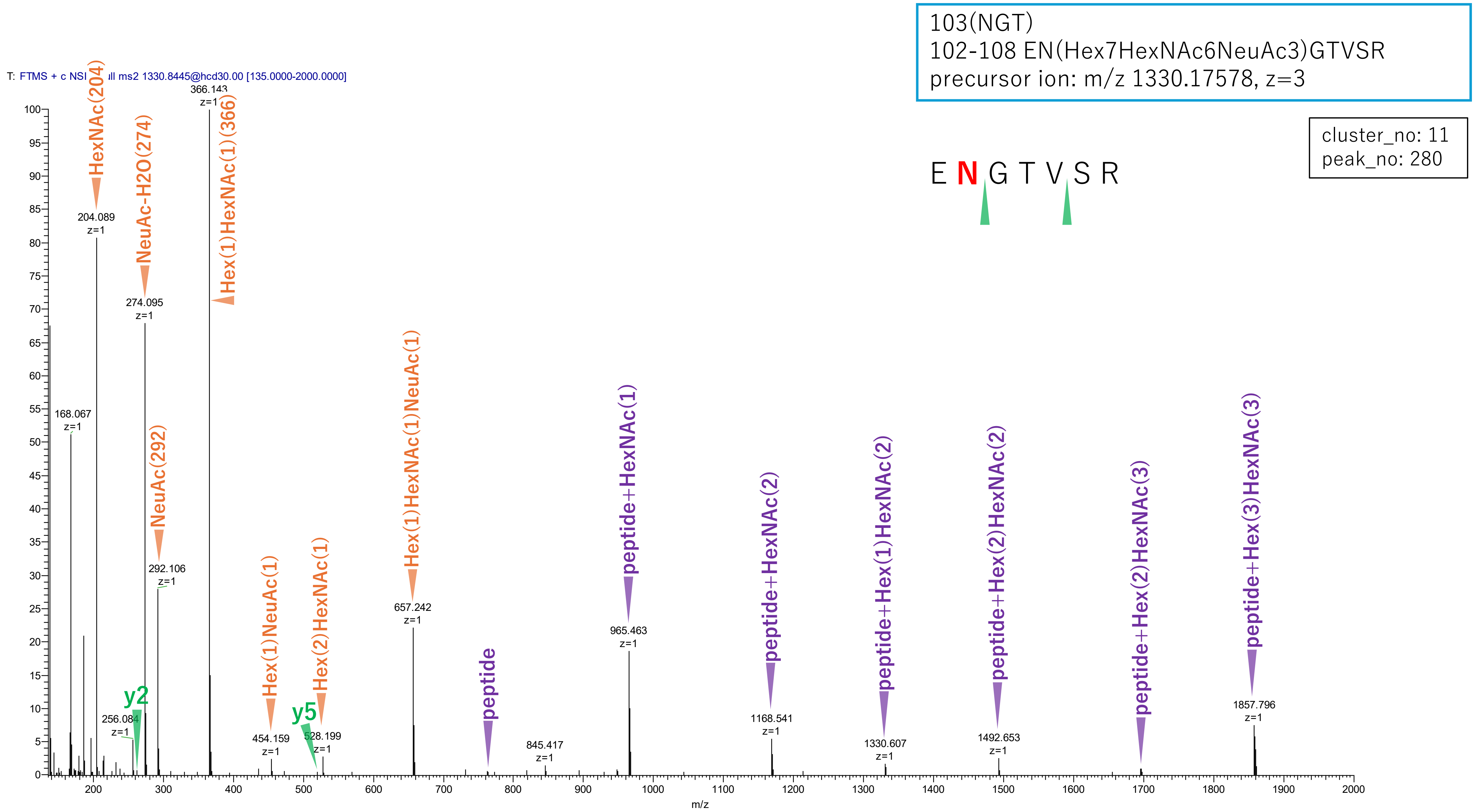

Figure S4-60. MS2 spectra of glycopeptides assigned for hAGP

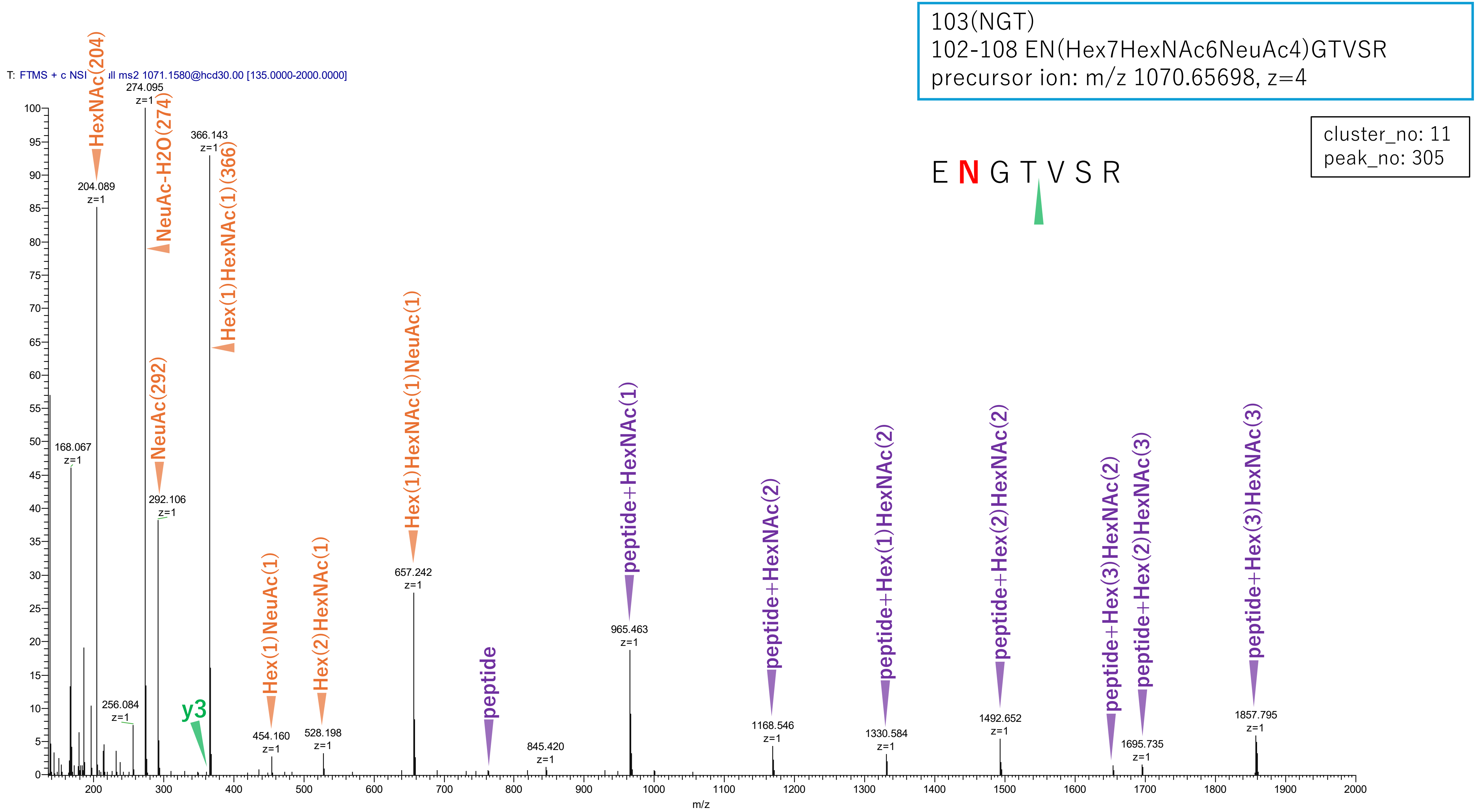

Figure S4-61. MS2 spectra of glycopeptides assigned for hAGP

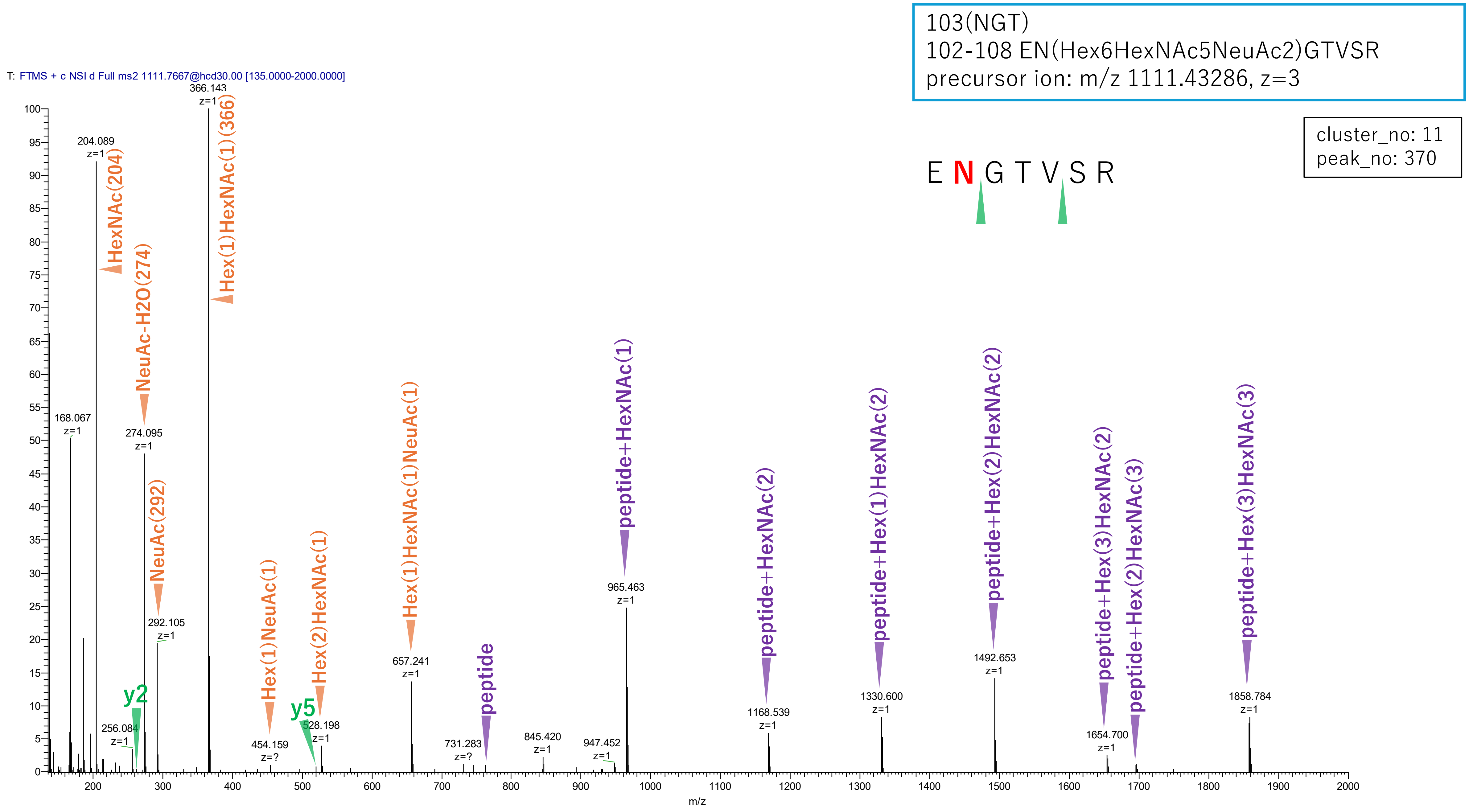

Figure S4-62. MS2 spectra of glycopeptides assigned for hAGP

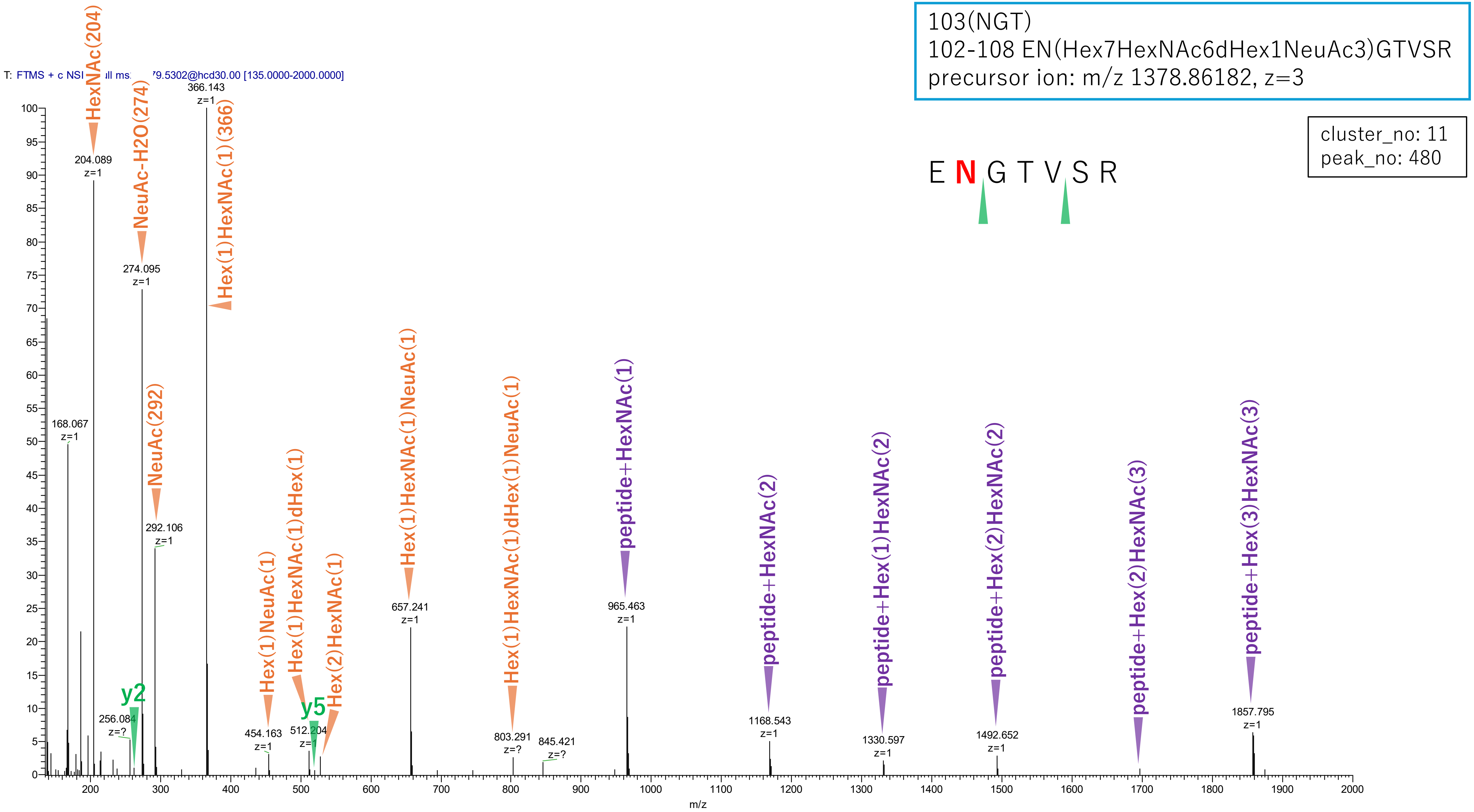

Figure S4-63. MS2 spectra of glycopeptides assigned for hAGP

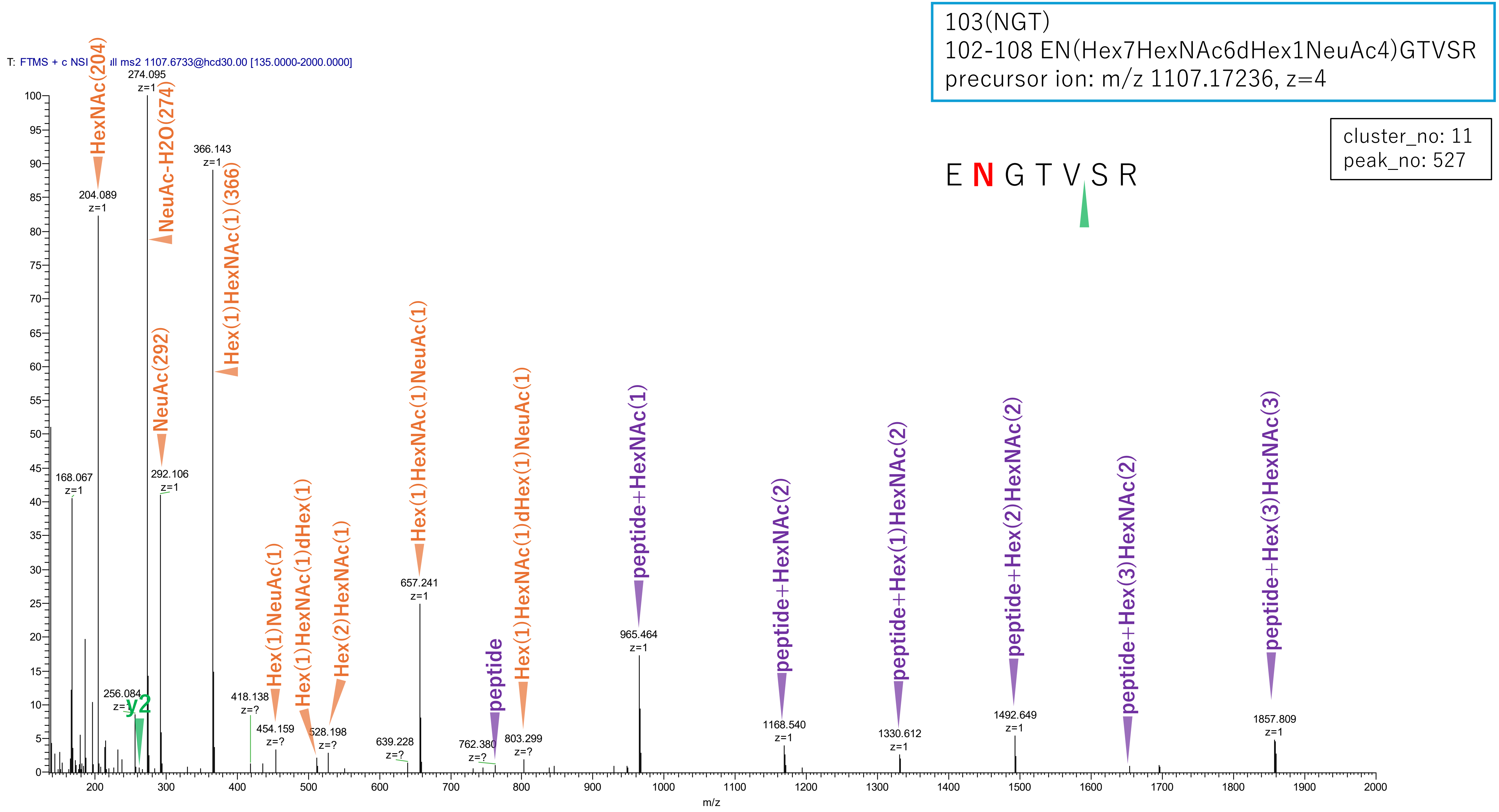

Figure S4-64. MS2 spectra of glycopeptides assigned for hAGP

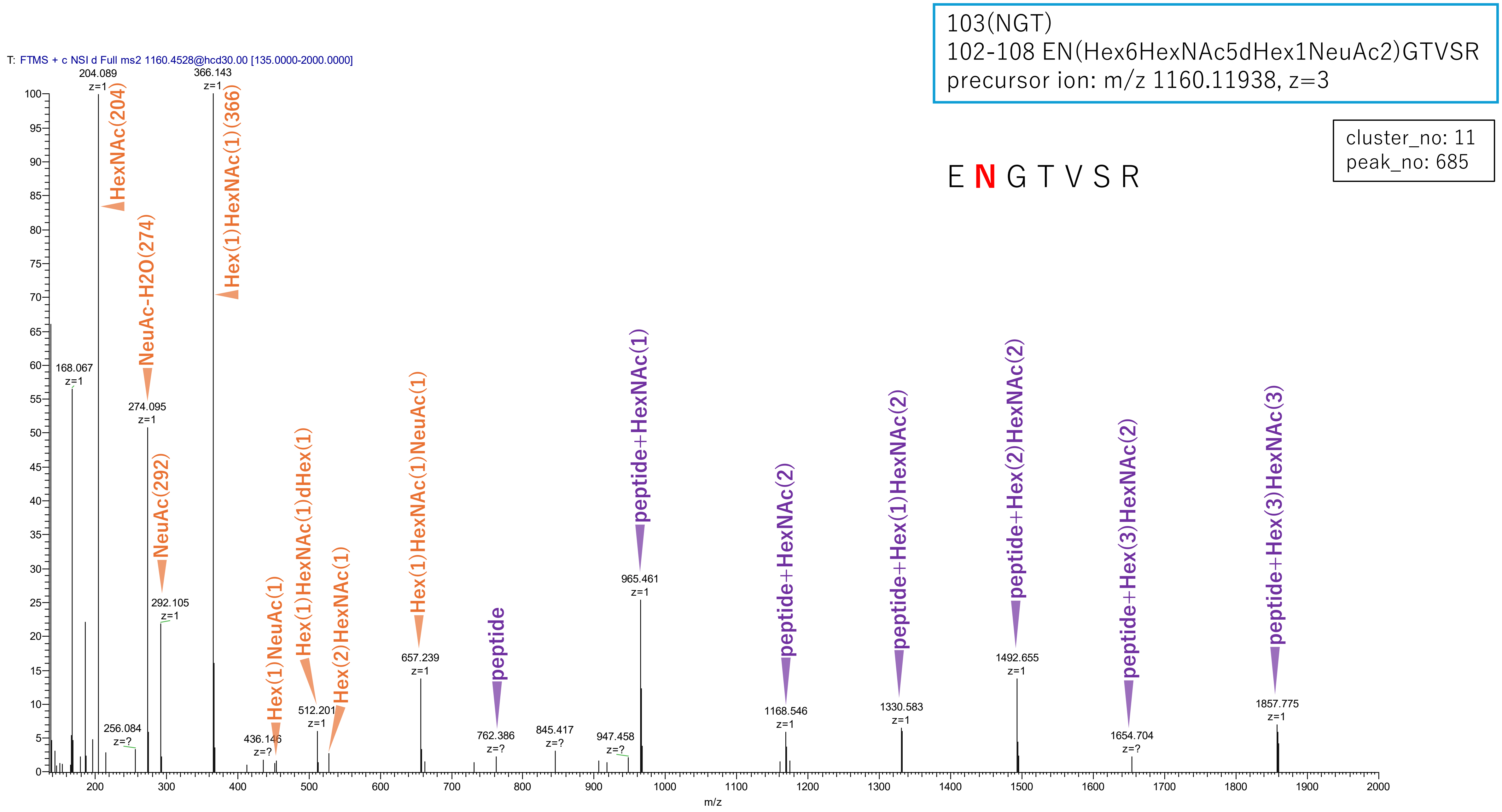

Figure S4-65. MS2 spectra of glycopeptides assigned for hAGP

103(NGT)
102-108 EN(Hex7HexNAc6NeuAc2)GTVSR
precursor ion: m/z 1233.14429, z=3

cluster_no: 11
peak_no: 762

E N G T V S R

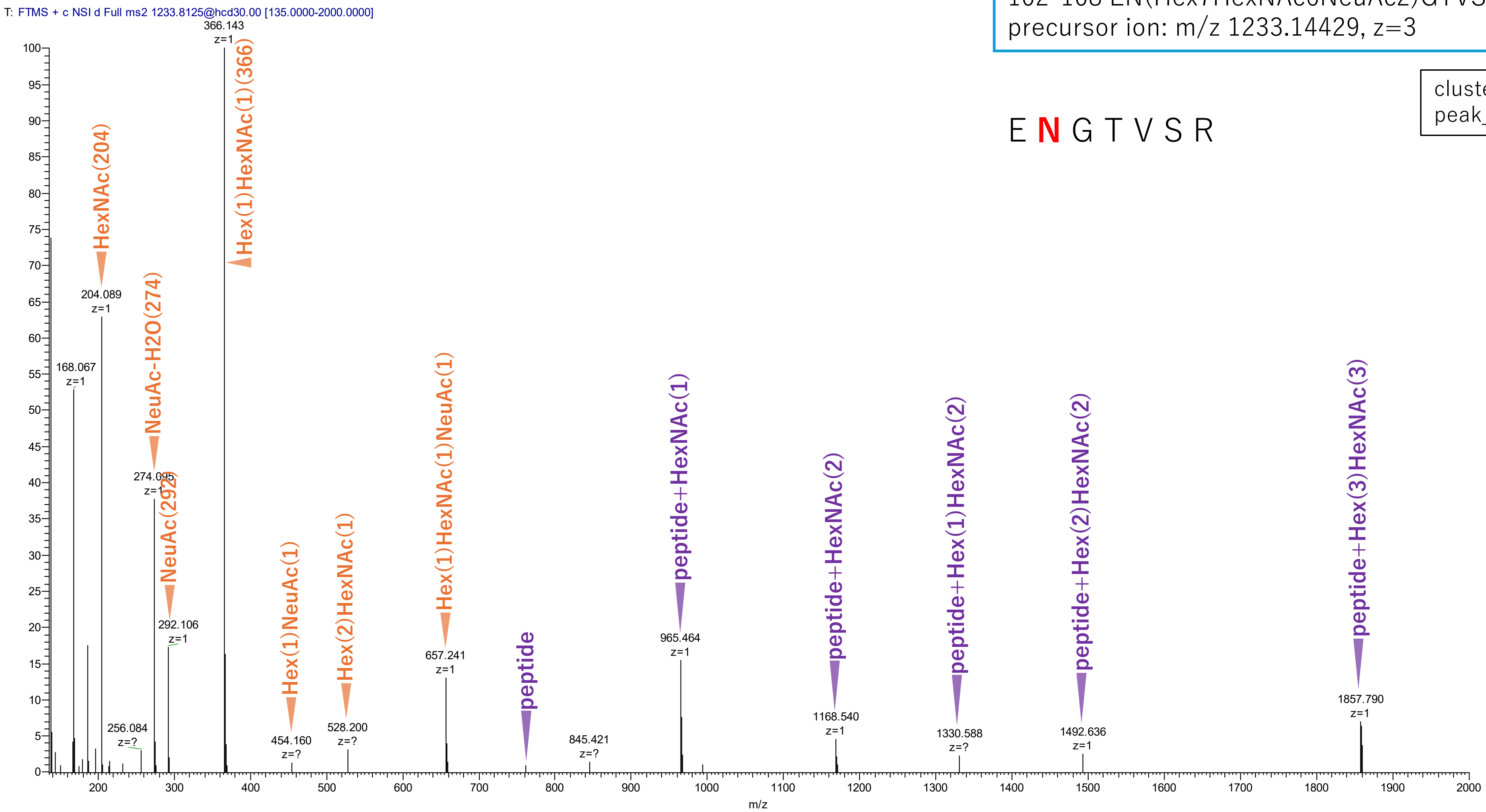

Figure S4-66. MS2 spectra of glycopeptides assigned for hAGP

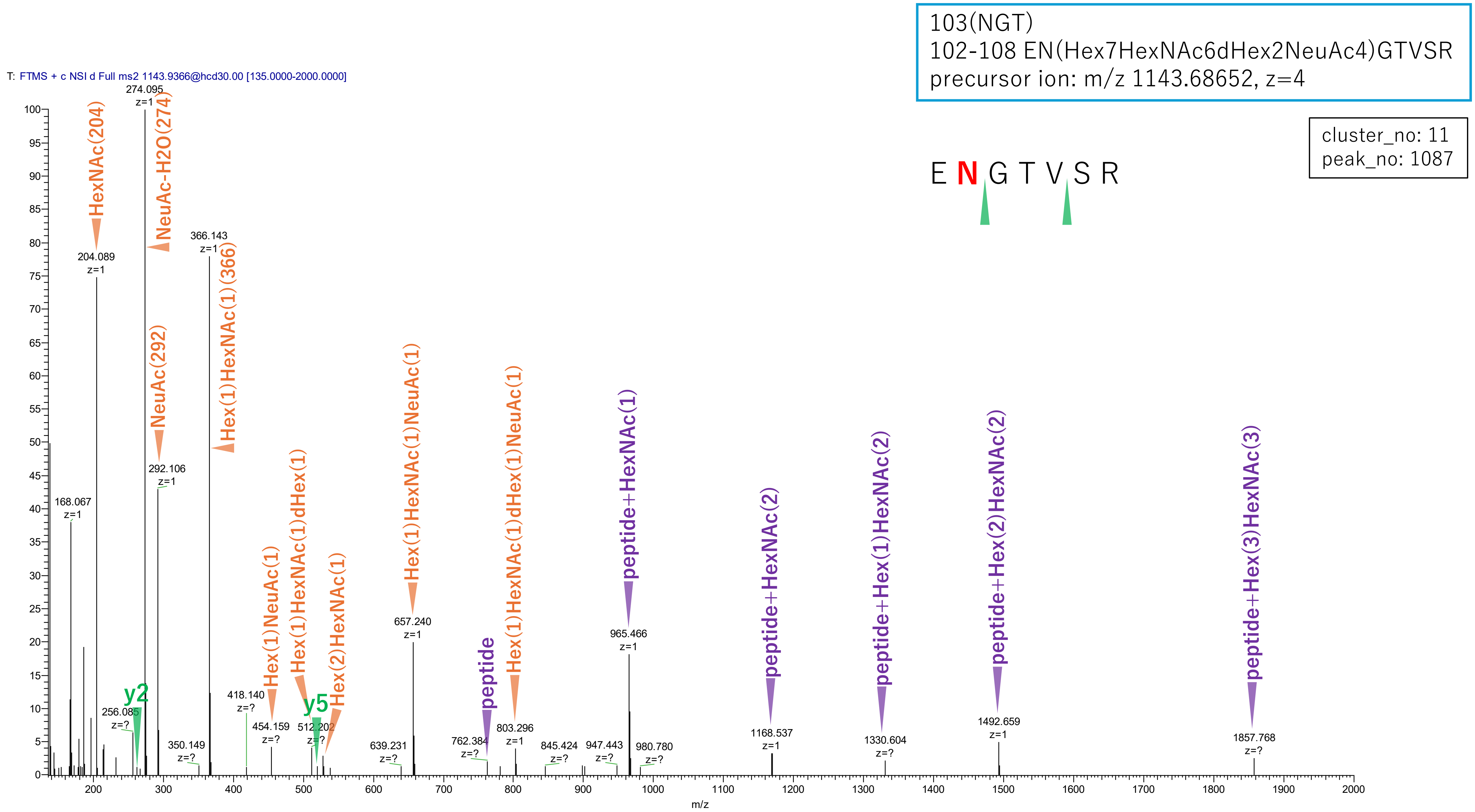

Figure S4-67. MS2 spectra of glycopeptides assigned for hAGP

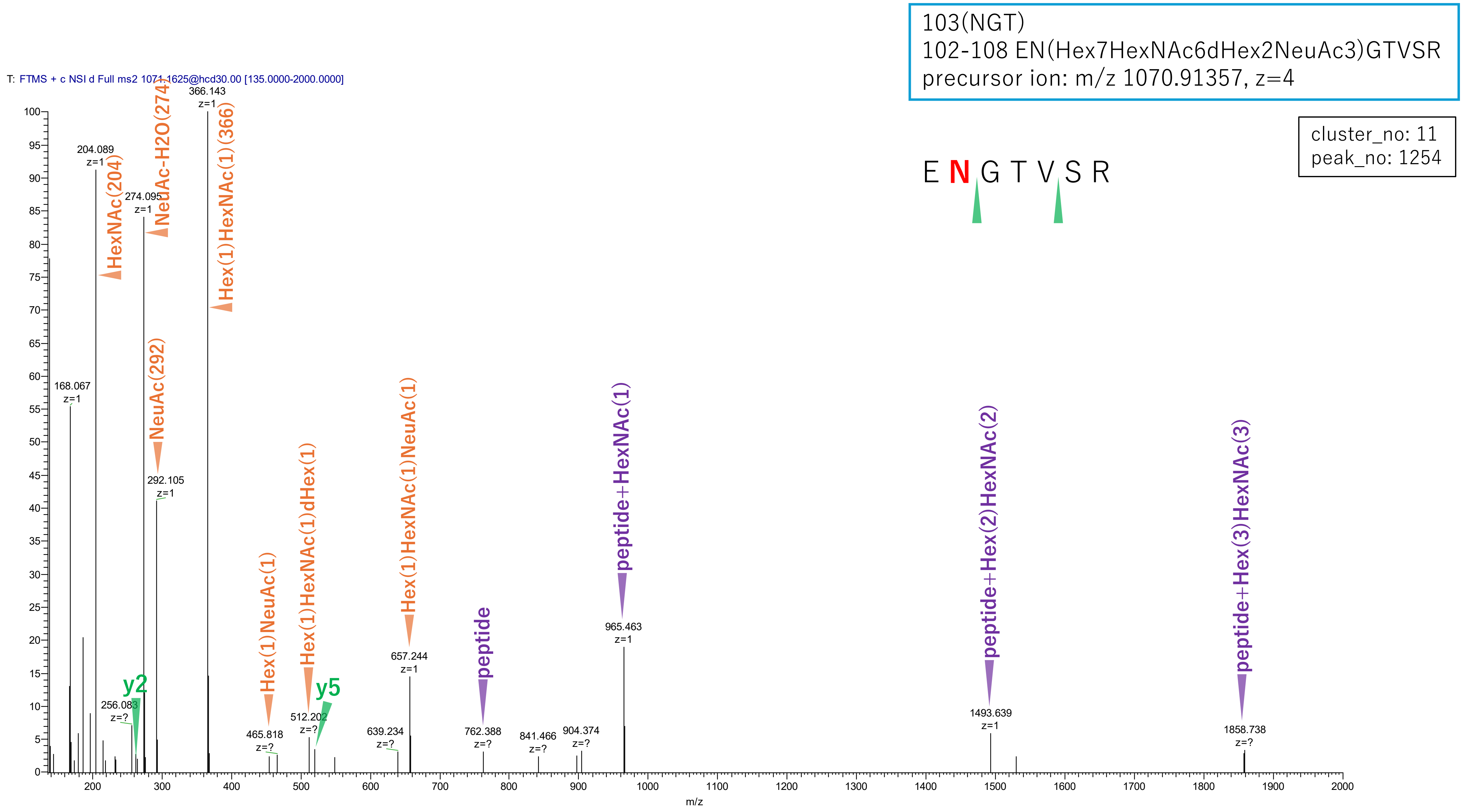

Figure S4-68. MS2 spectra of glycopeptides assigned for hAGP

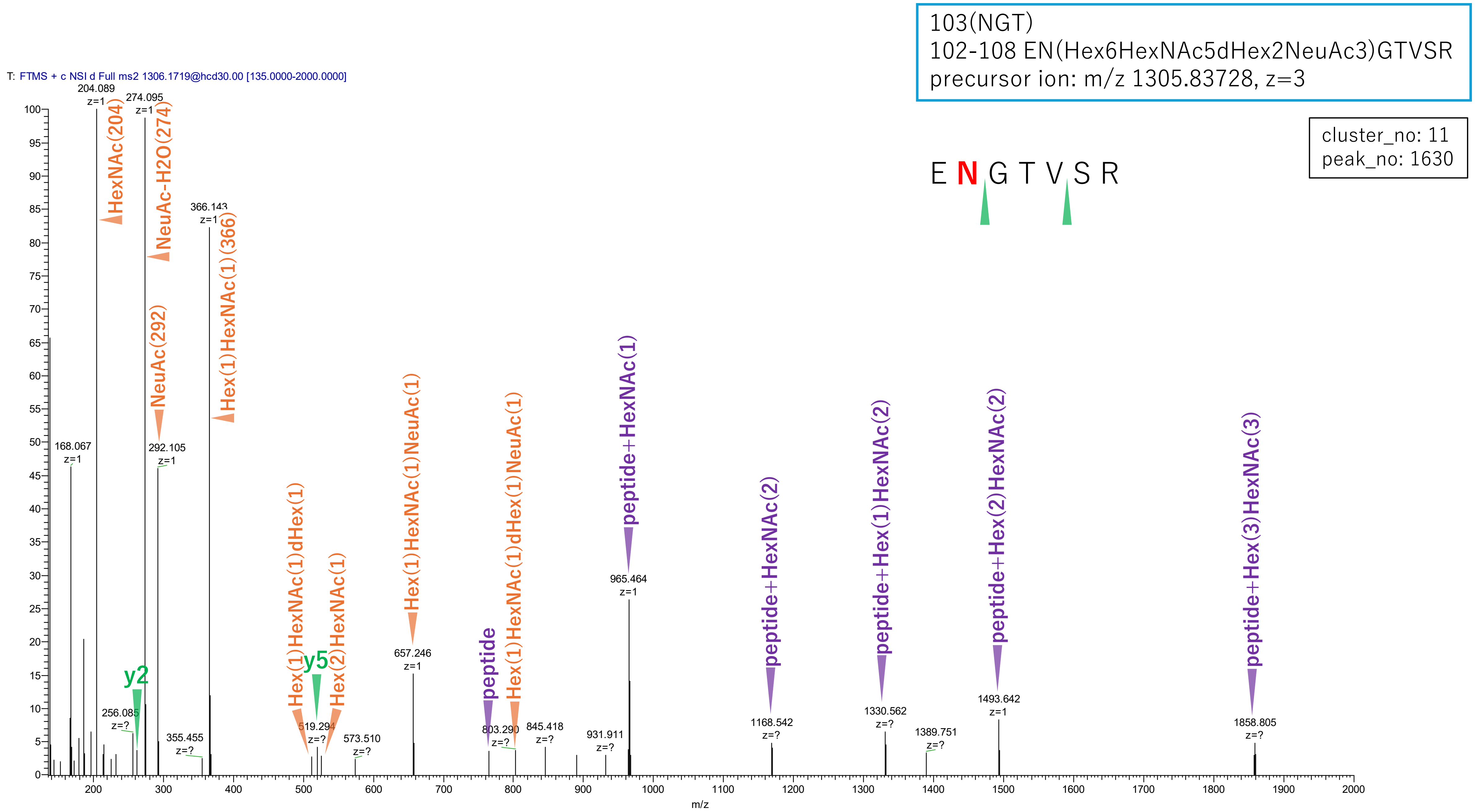

Figure S4-69. MS2 spectra of glycopeptides assigned for hAGP

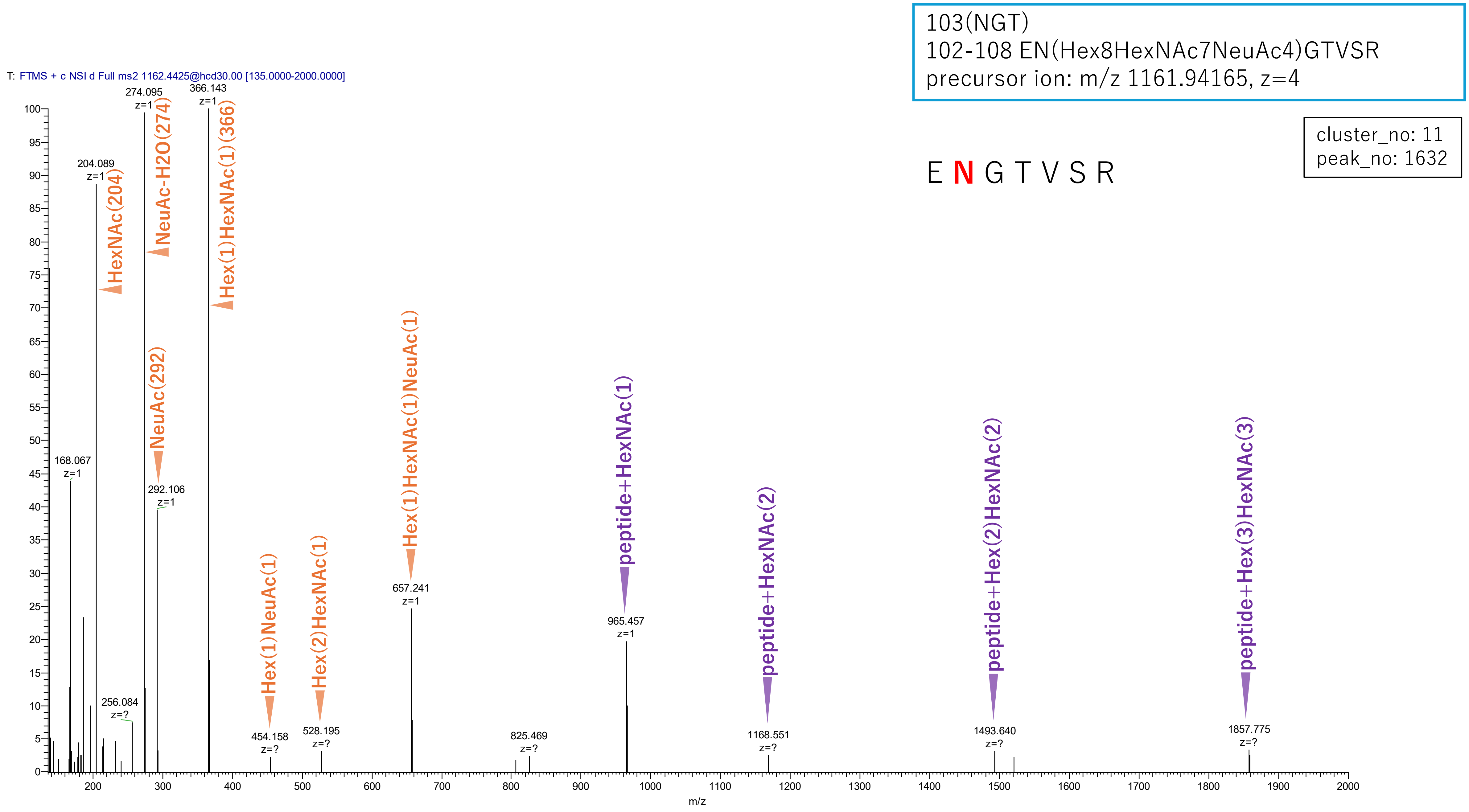

Figure S4-70. MS2 spectra of glycopeptides assigned for hAGP

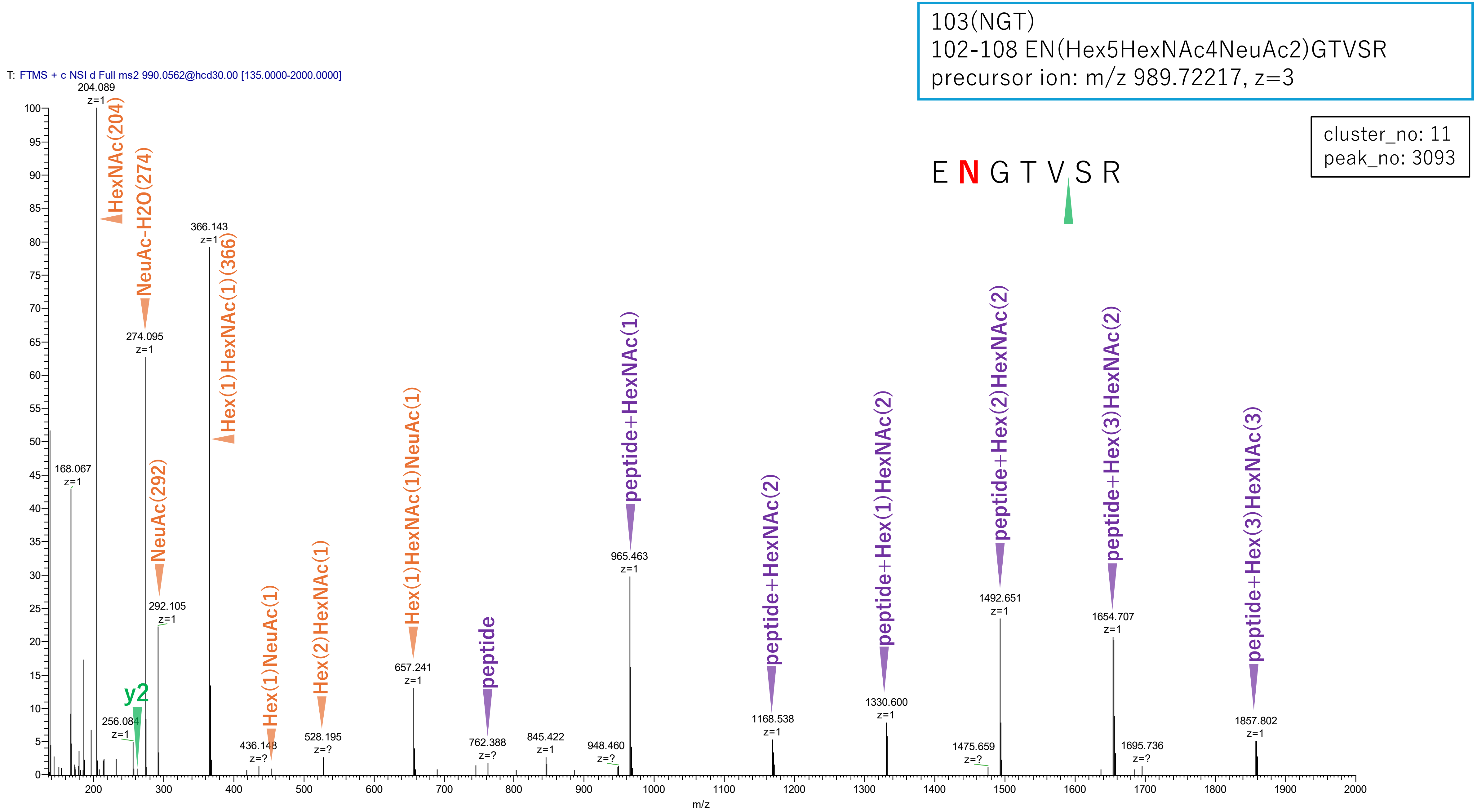

Figure S4-71. MS2 spectra of glycopeptides assigned for hAGP

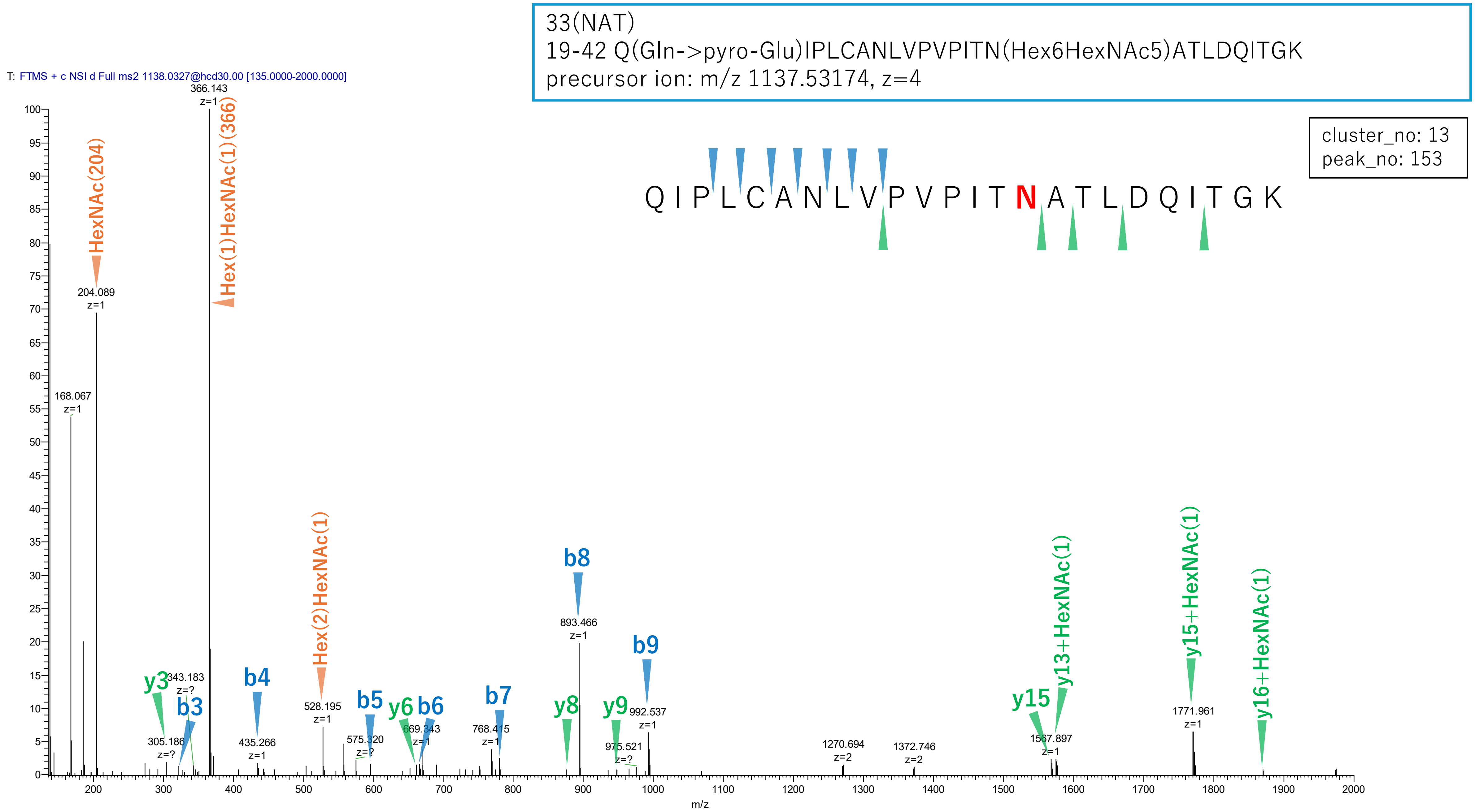

Figure S4-72. MS2 spectra of glycopeptides assigned for hAGP

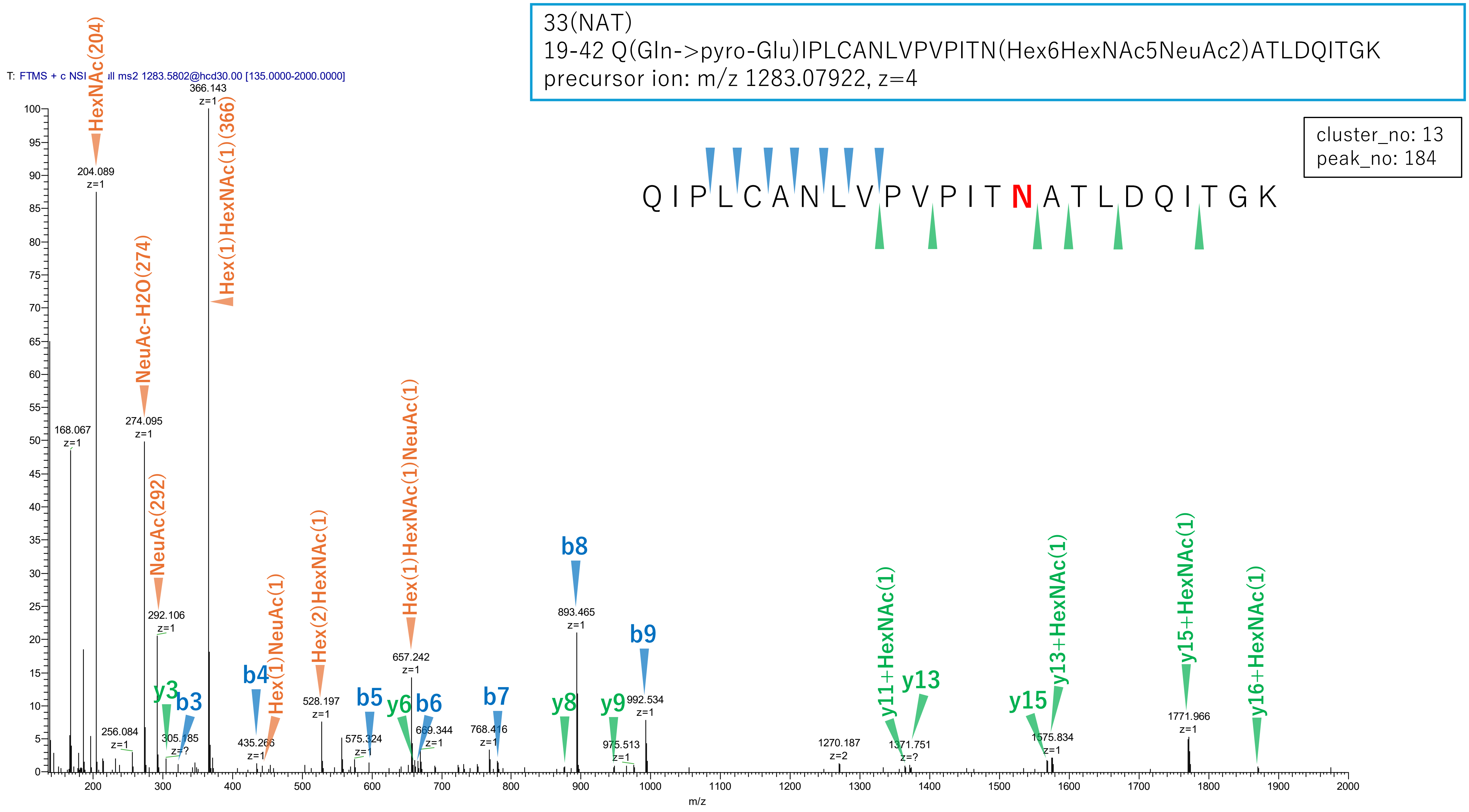

Figure S4-73. MS2 spectra of glycopeptides assigned for hAGP

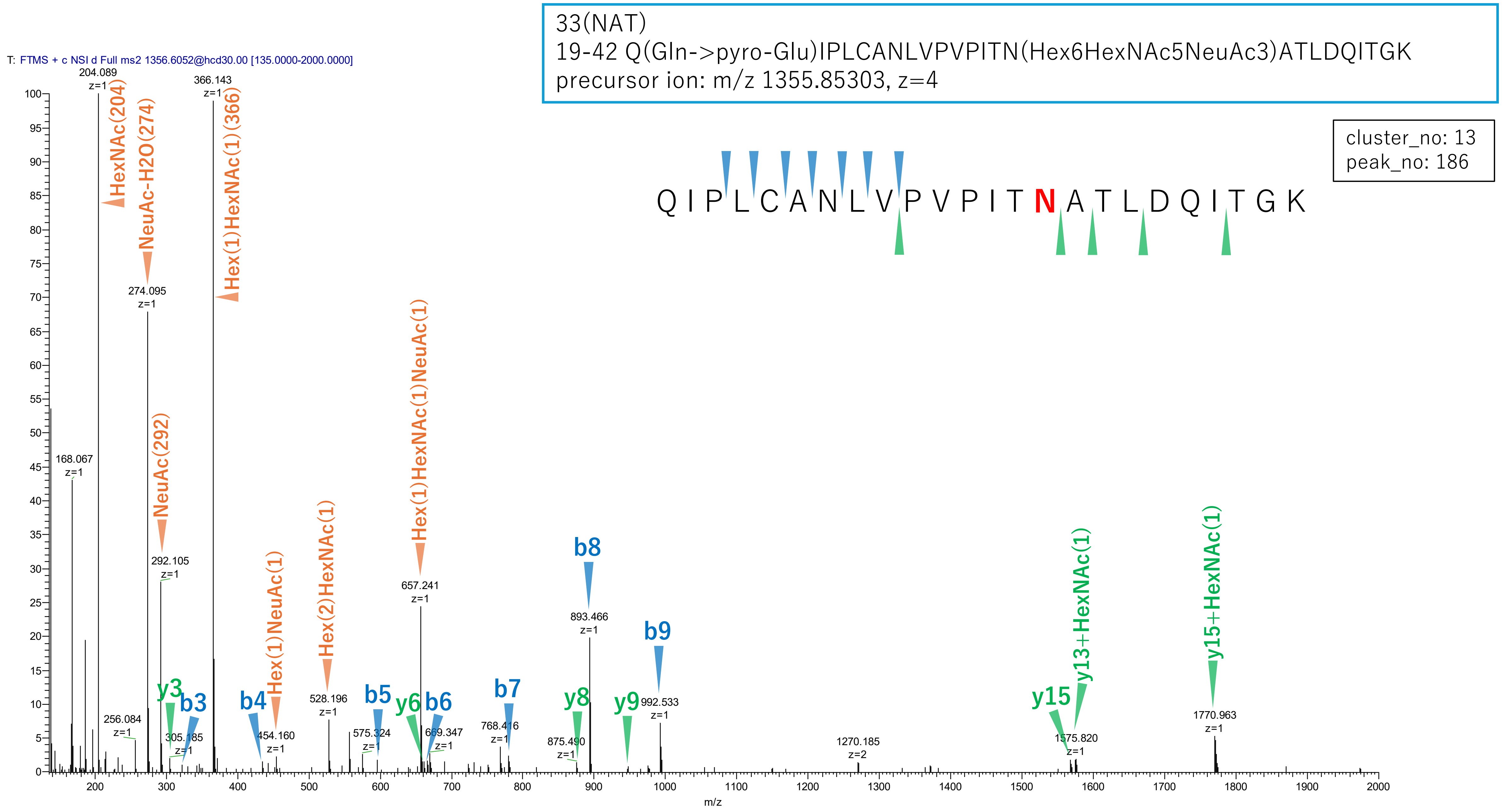

Figure S4-74. MS2 spectra of glycopeptides assigned for hAGP

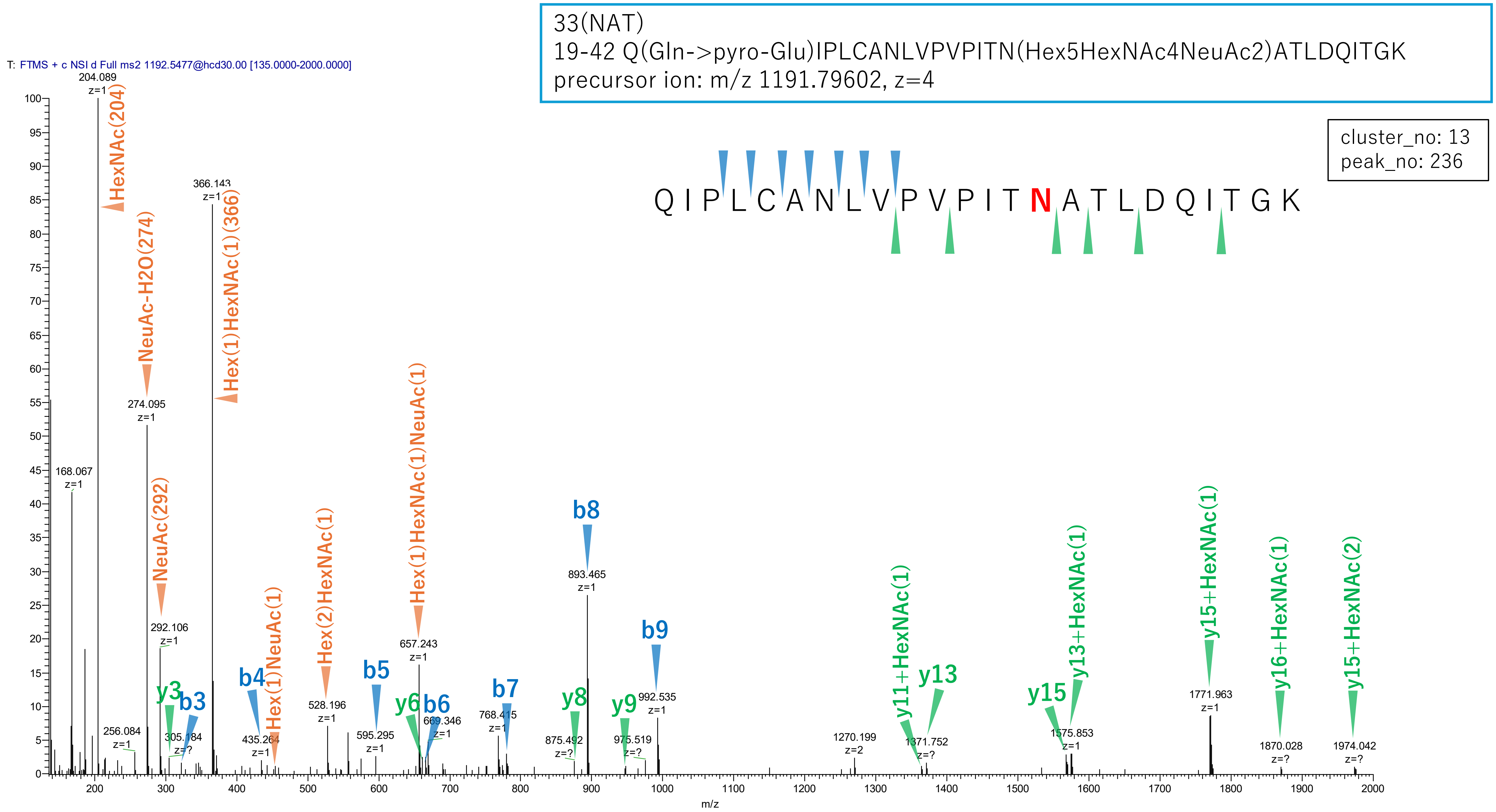

Figure S4-75. MS2 spectra of glycopeptides assigned for hAGP

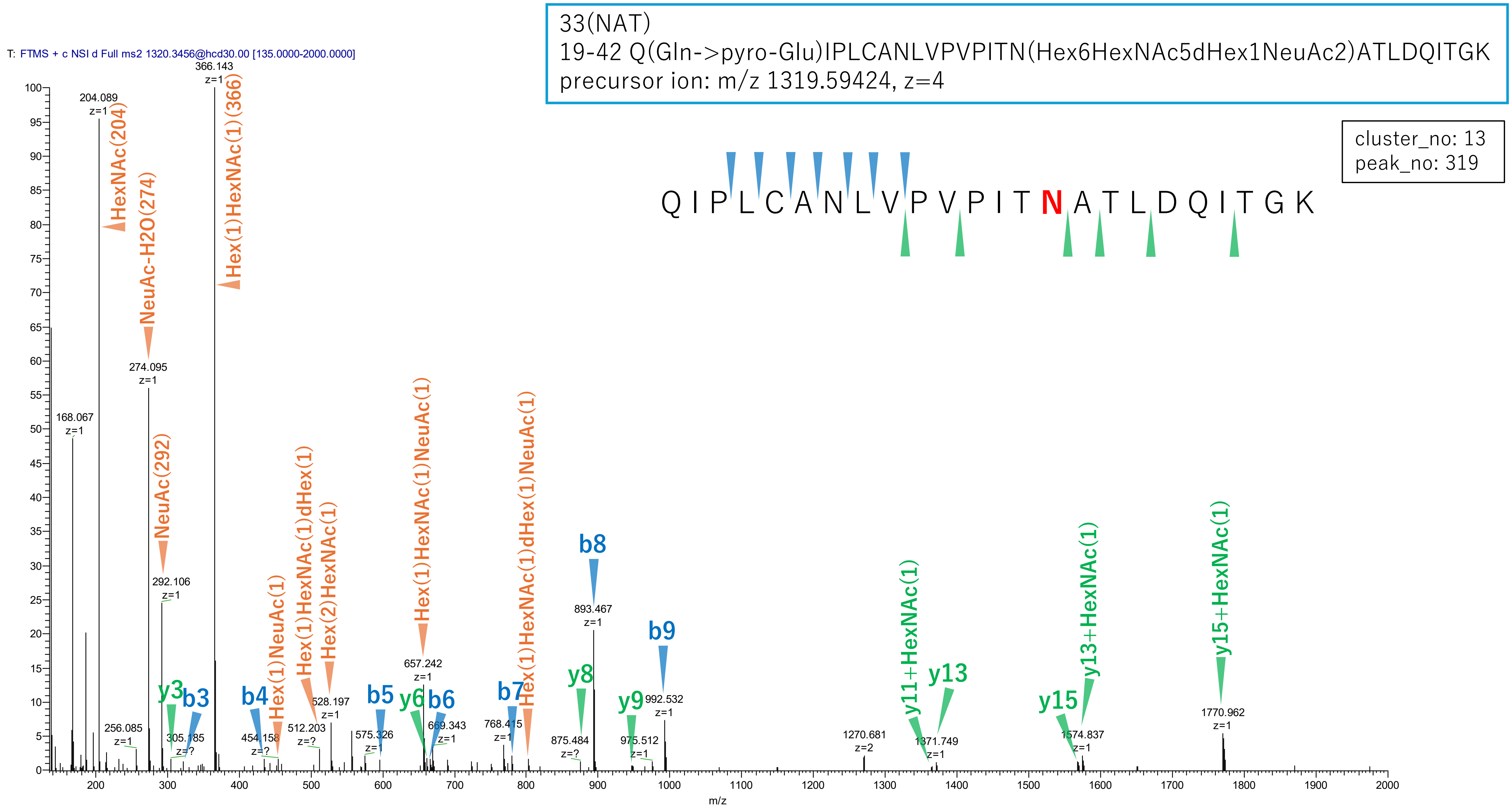

Figure S4-76. MS2 spectra of glycopeptides assigned for hAGP

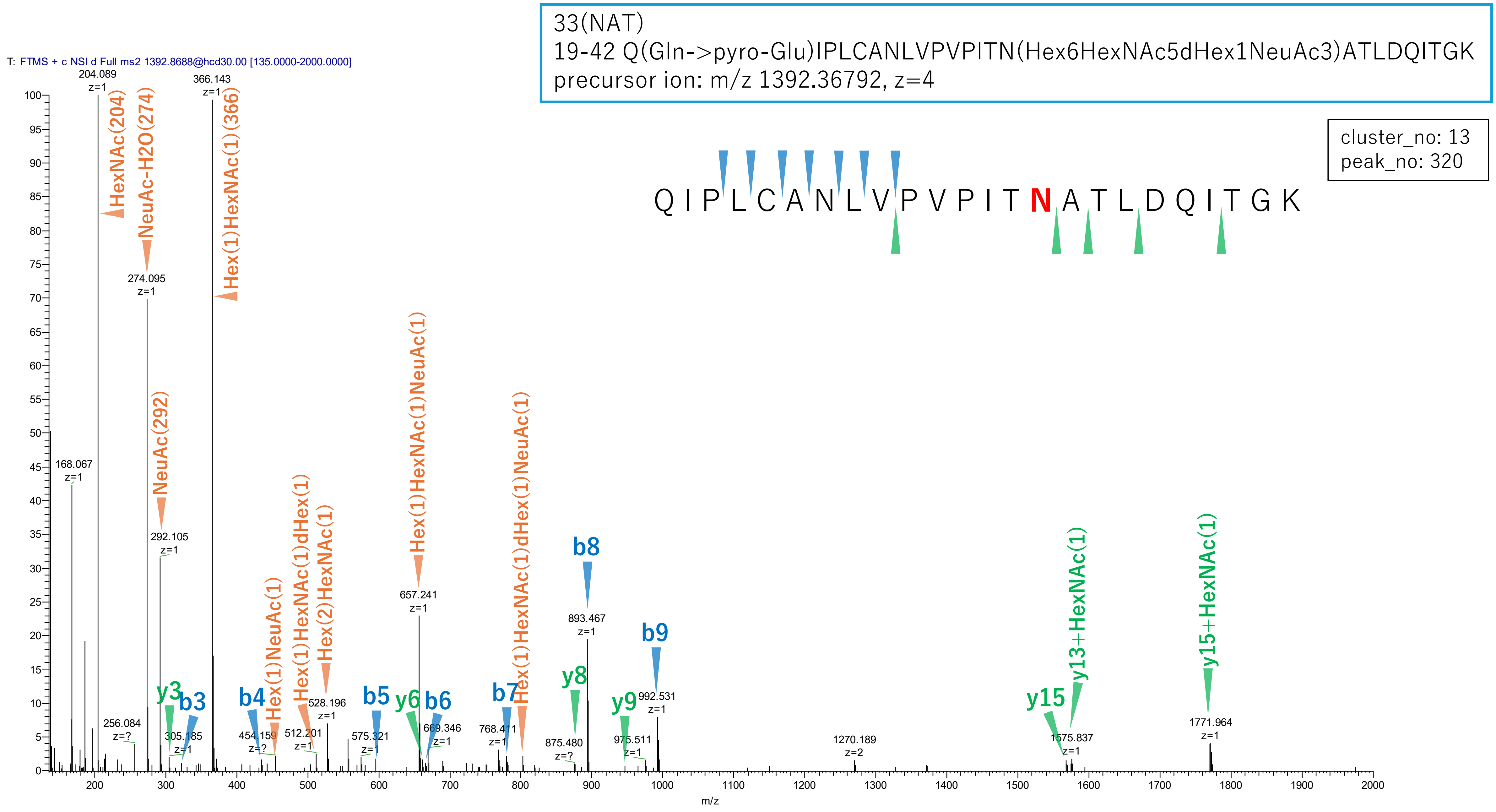

Figure S4-77. MS2 spectra of glycopeptides assigned for hAGP

33(NAT)
19-42 Q(Gln->pyro-Glu)IPLCANLVPVPITN(Hex6HexNAc5NeuAc1)ATLDQITGK
precursor ion: m/z 1210.30457, z=4

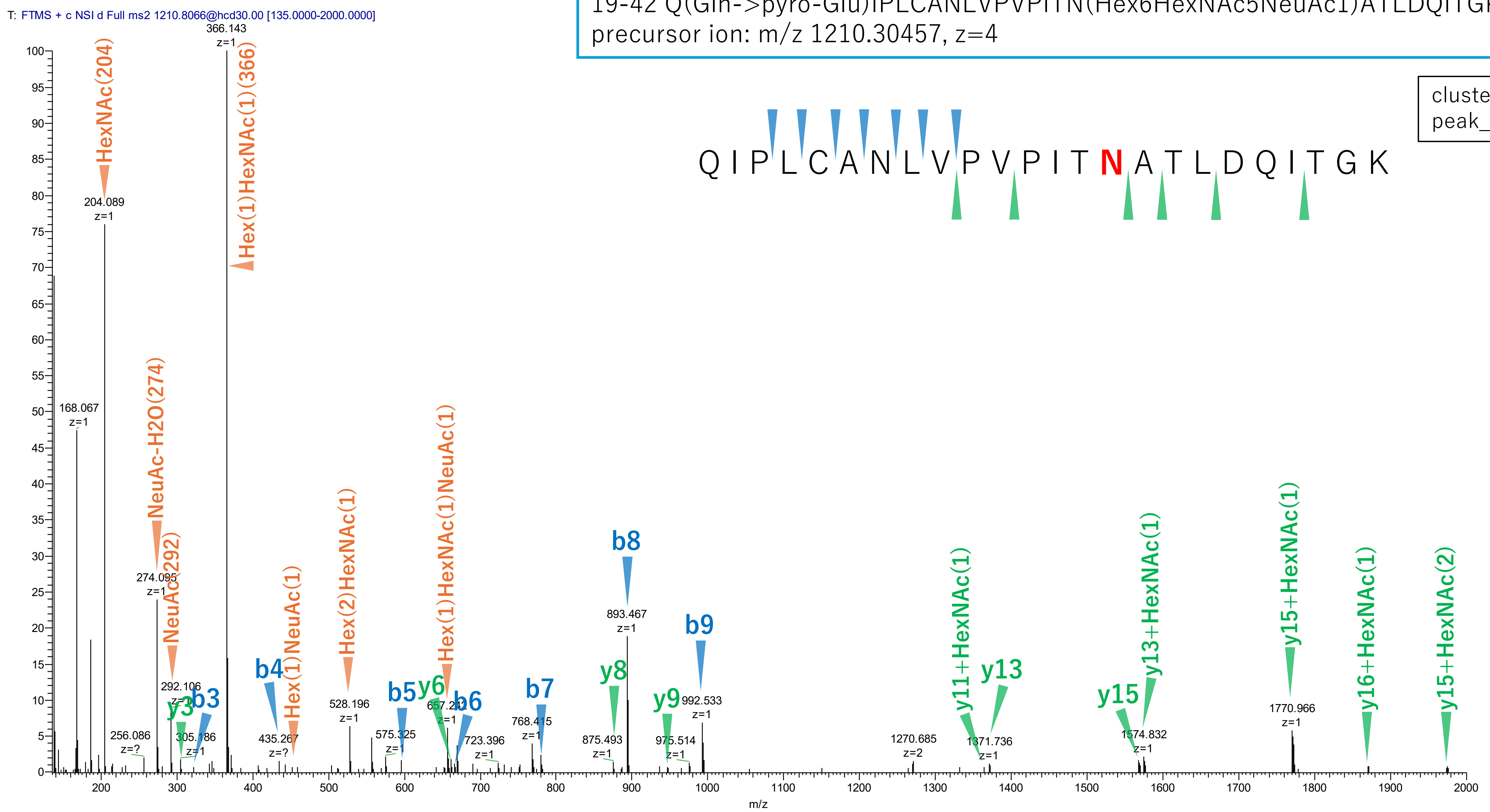

Figure S4-78. MS2 spectra of glycopeptides assigned for hAGP

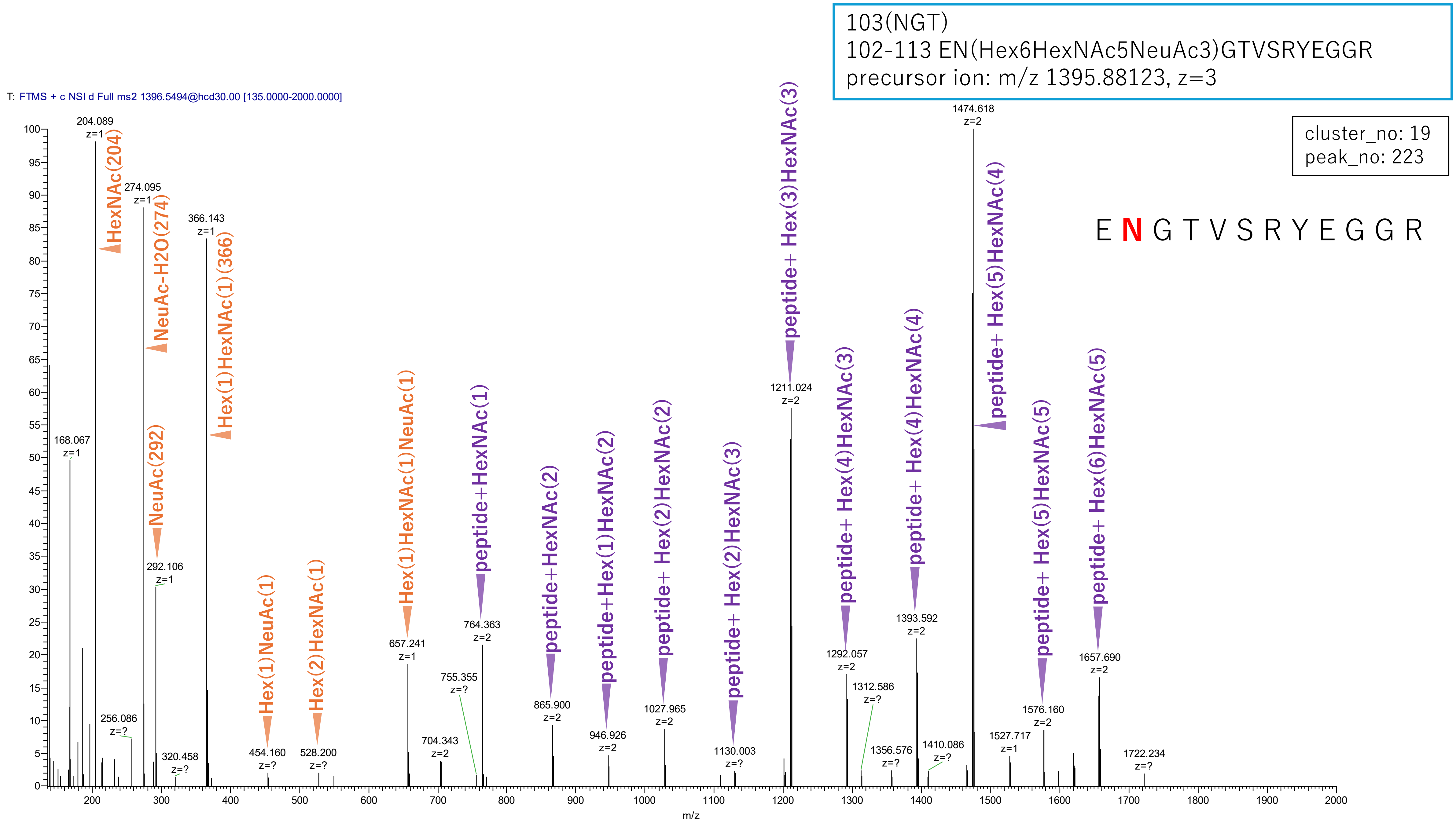

Figure S4-79. MS2 spectra of glycopeptides assigned for hAGP

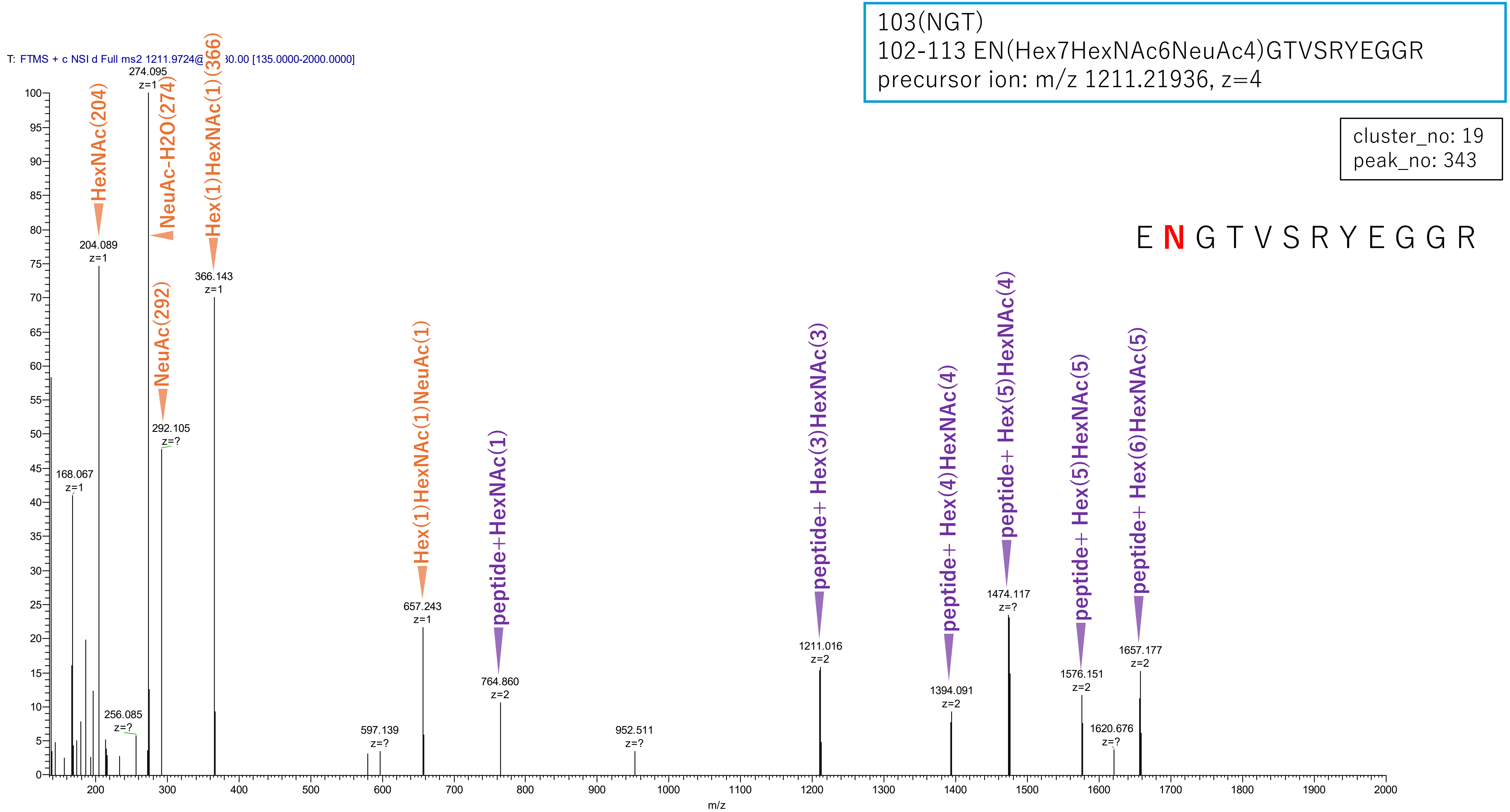

Figure S4-80. MS2 spectra of glycopeptides assigned for hAGP

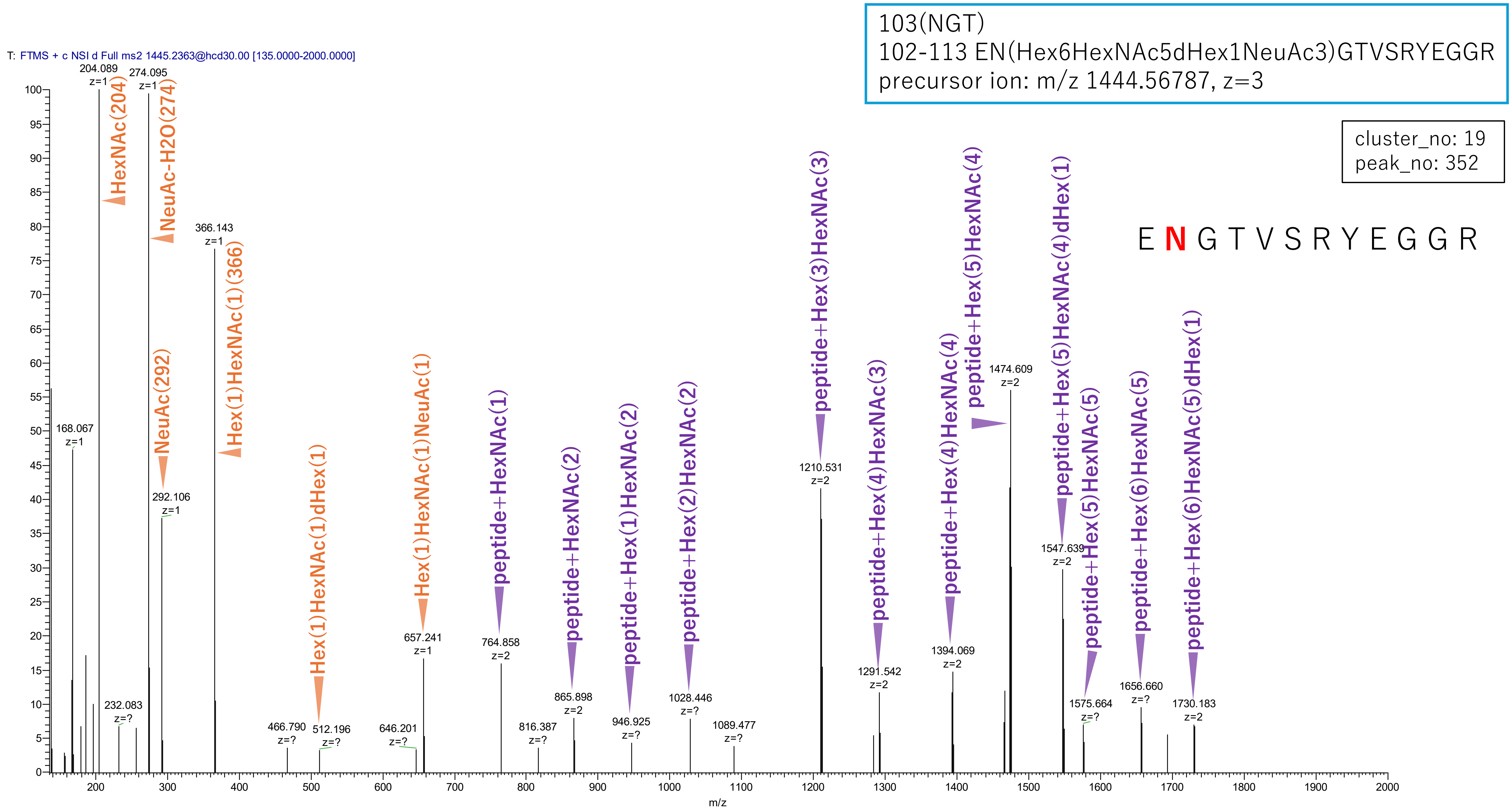

Figure S4-81. MS2 spectra of glycopeptides assigned for hAGP

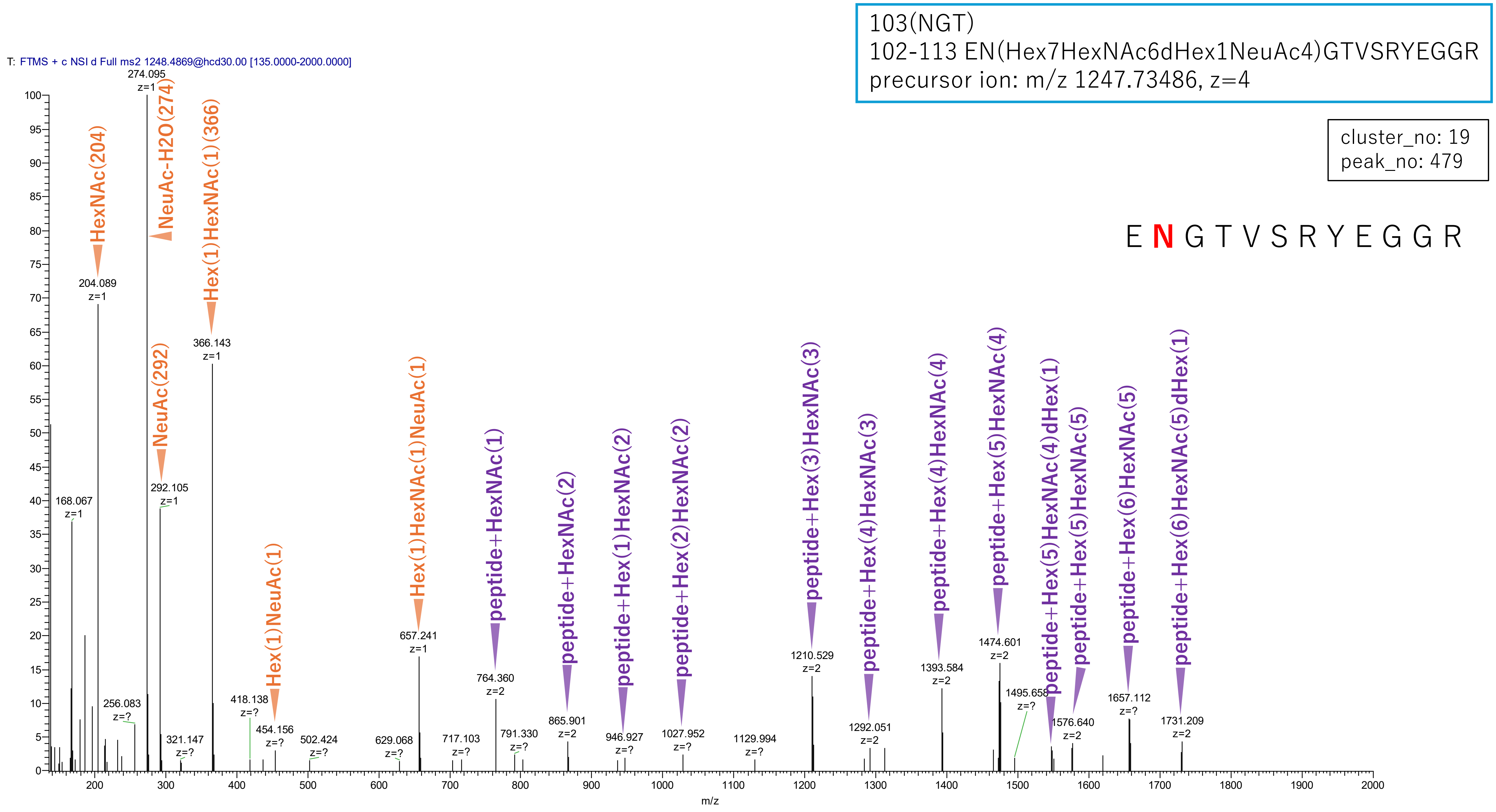

Figure S4-82. MS2 spectra of glycopeptides assigned for hAGP

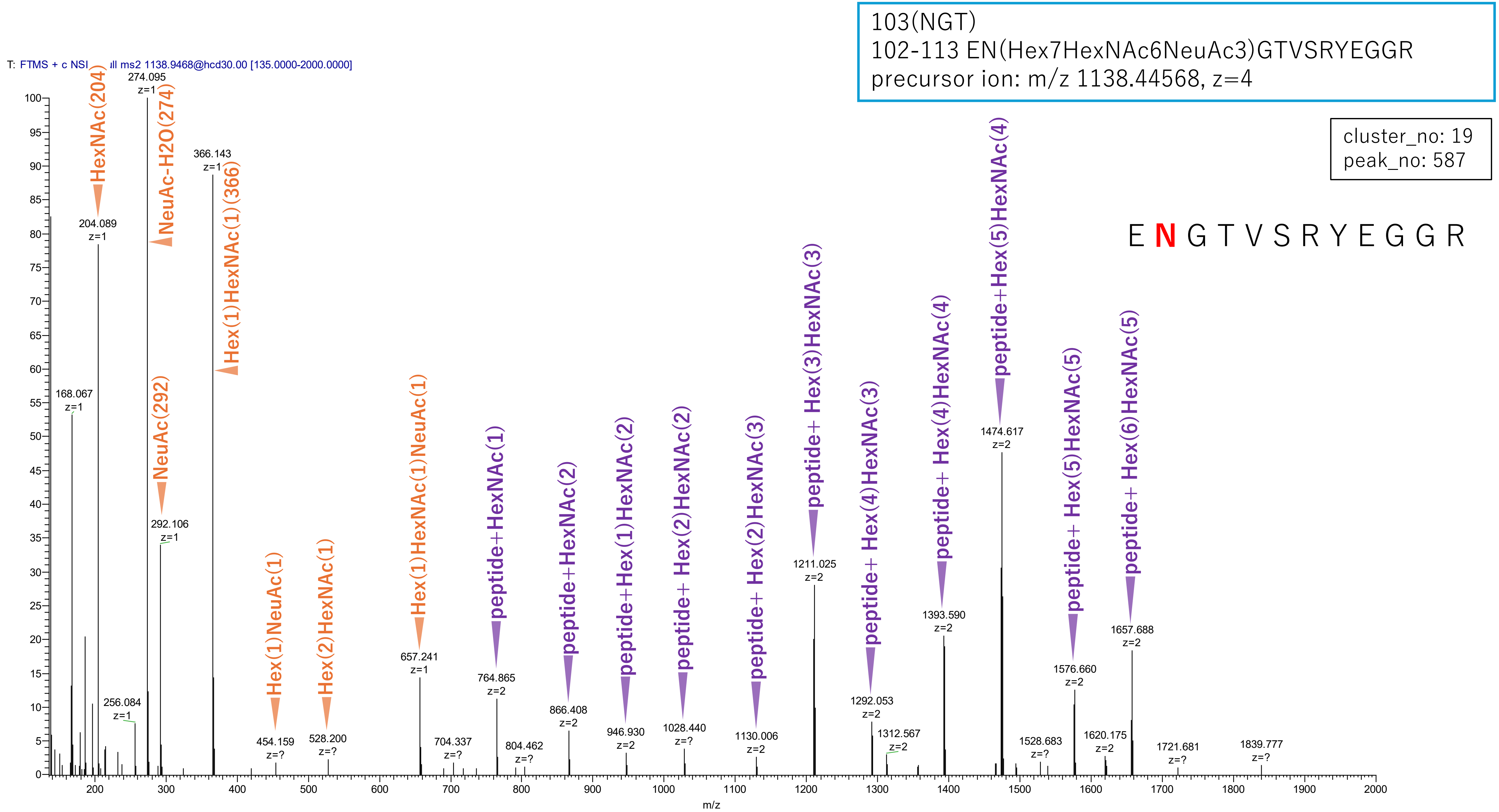

Figure S4-83. MS2 spectra of glycopeptides assigned for hAGP

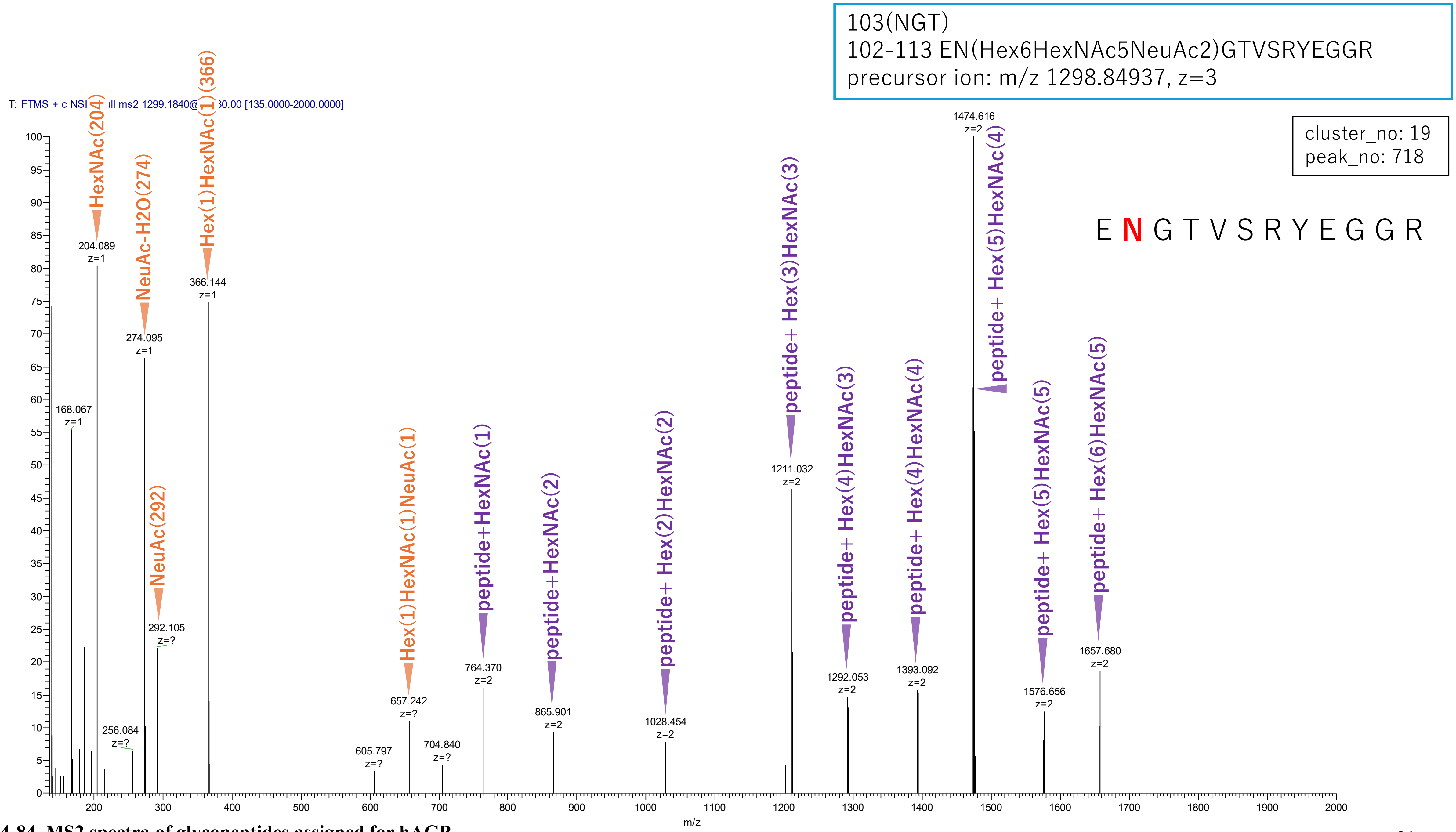

Figure S4-84. MS2 spectra of glycopeptides assigned for hAGP

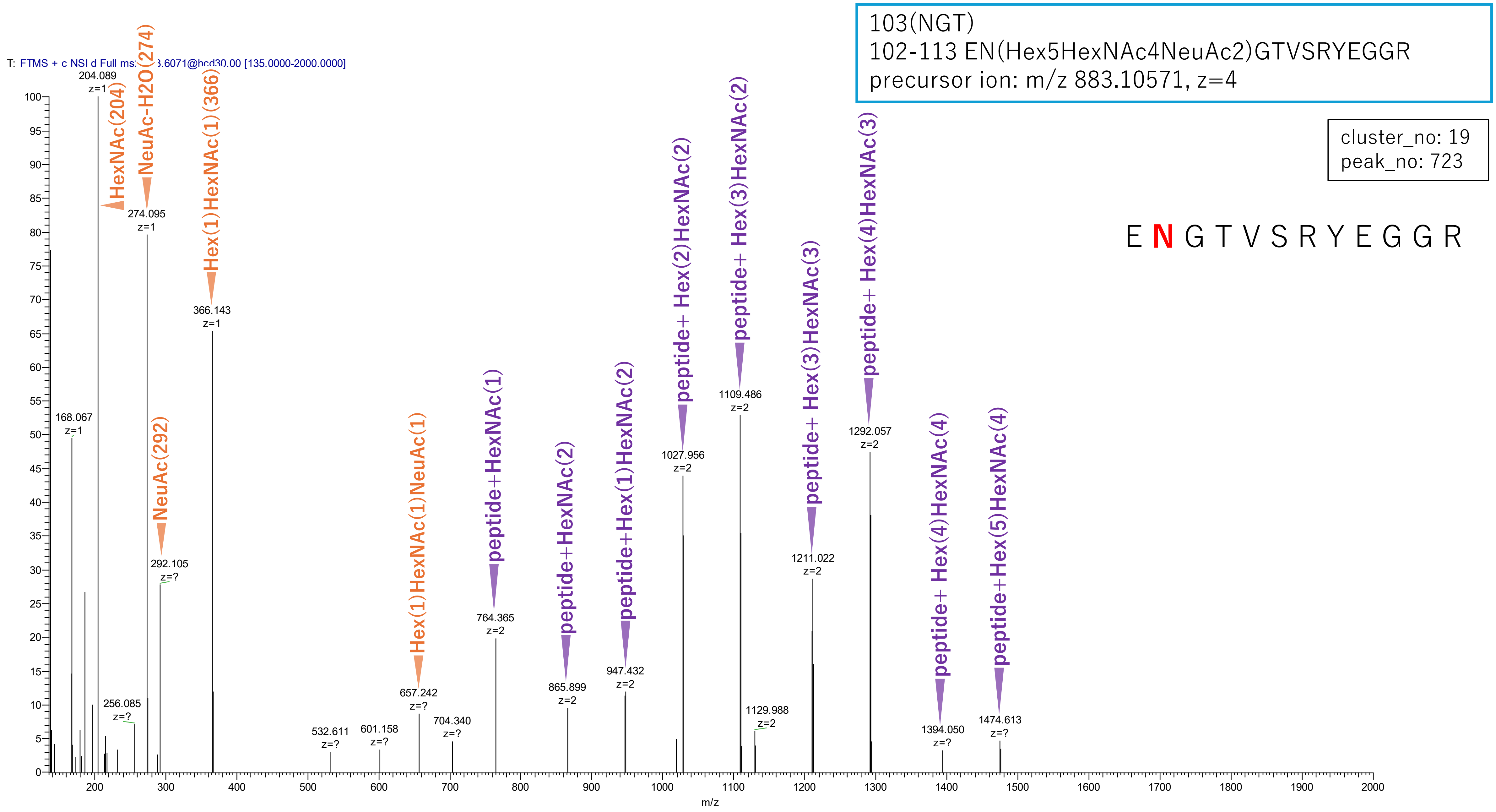

Figure S4-85. MS2 spectra of glycopeptides assigned for hAGP

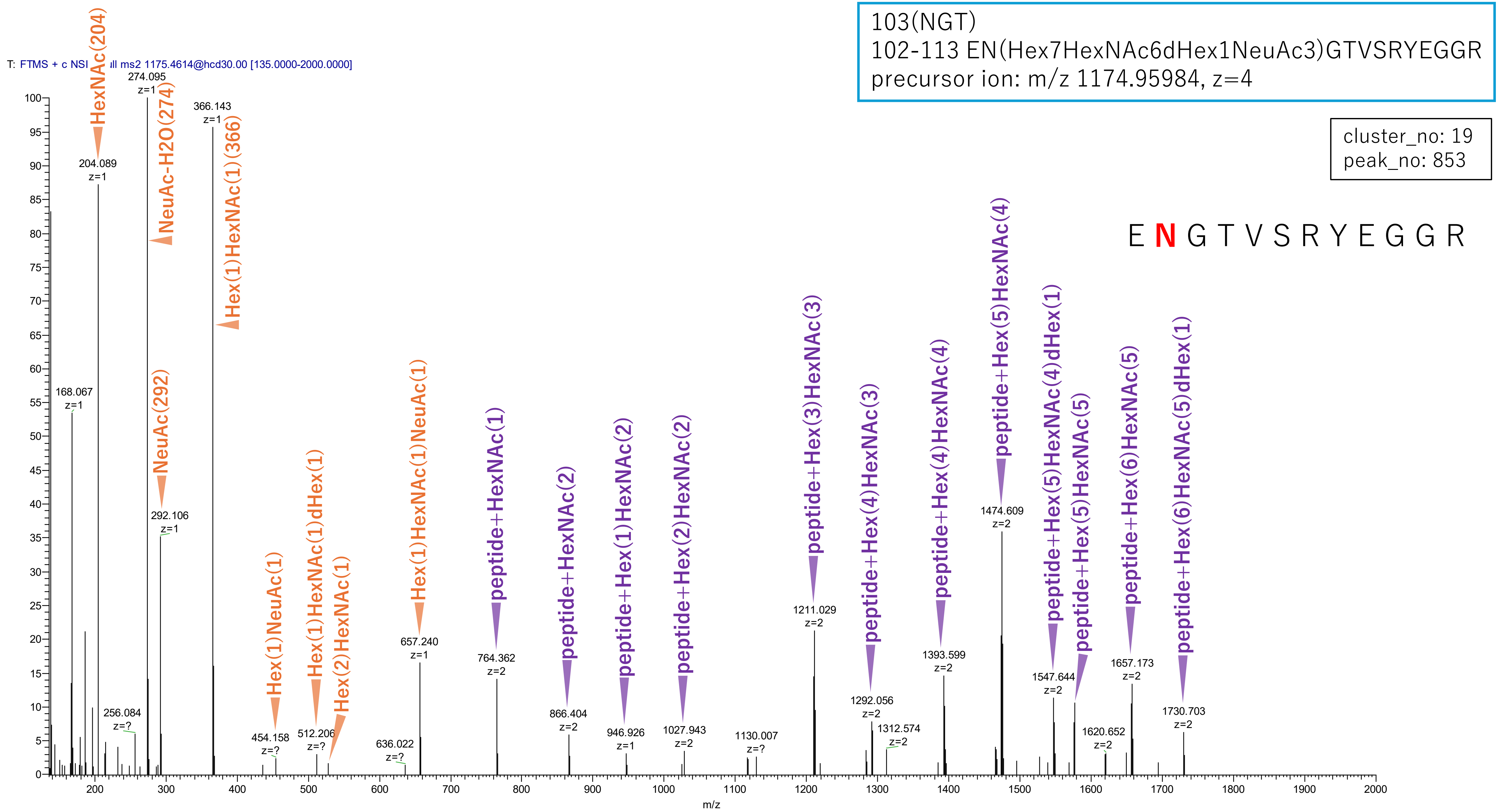

Figure S4-86. MS2 spectra of glycopeptides assigned for hAGP

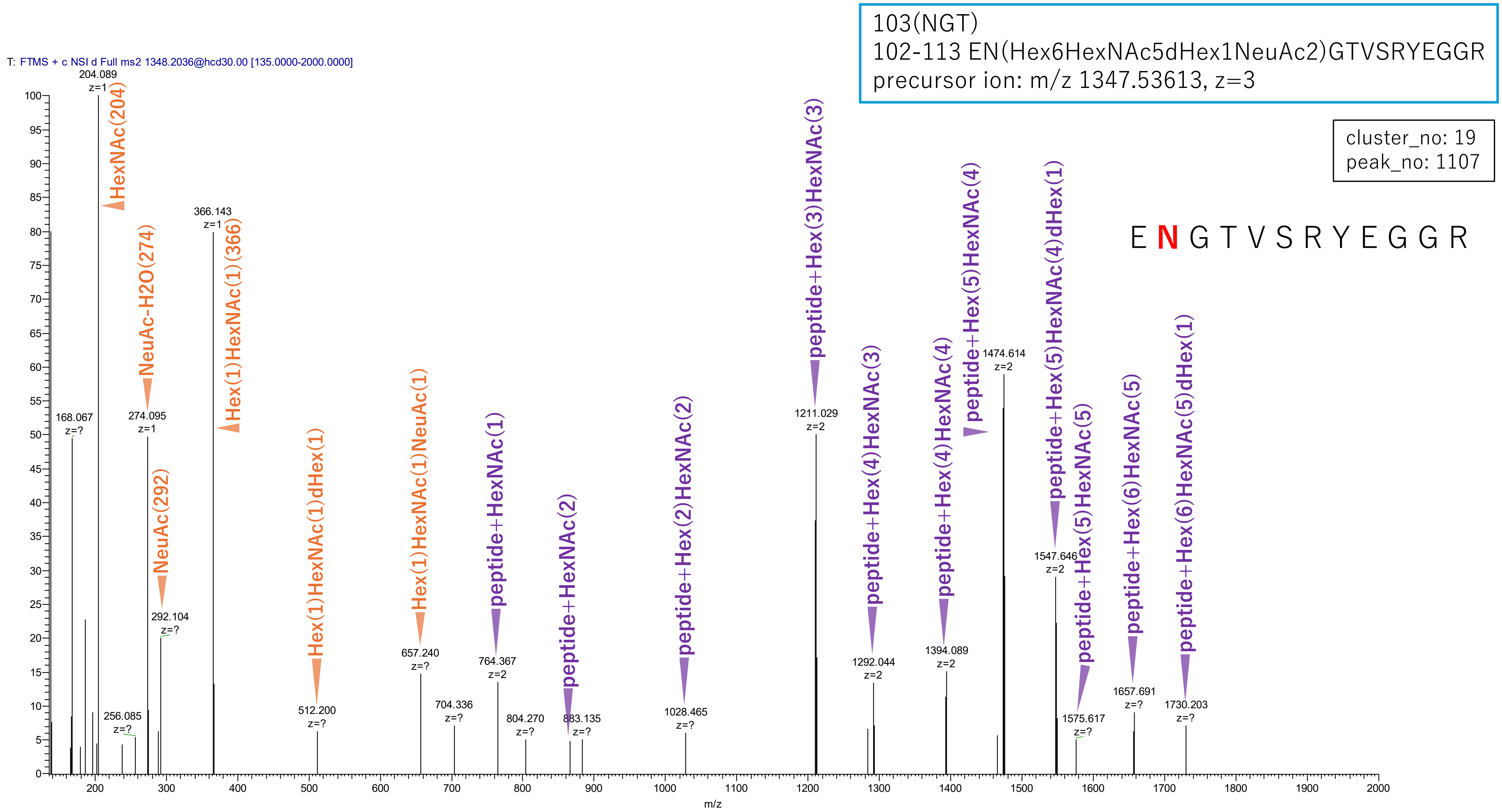

Figure S4-87. MS2 spectra of glycopeptides assigned for hAGP

103(NGT)
102-113 EN(Hex7HexNAc6dHex2NeuAc4)GTVSRYEGGR
precursor ion: m/z 1284.24719, z=4

cluster_no: 19
peak_no: 1131

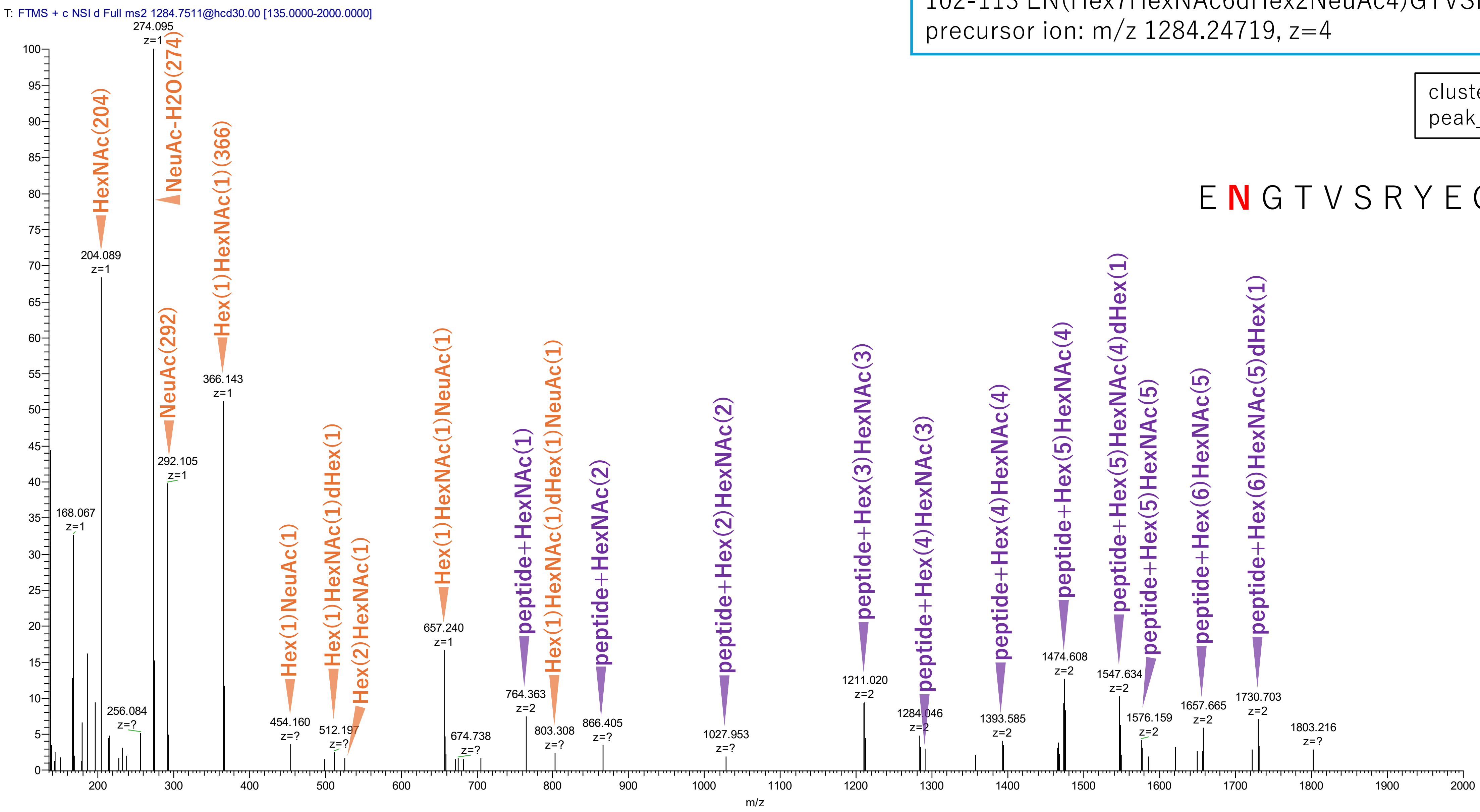

Figure S4-88. MS2 spectra of glycopeptides assigned for hAGP

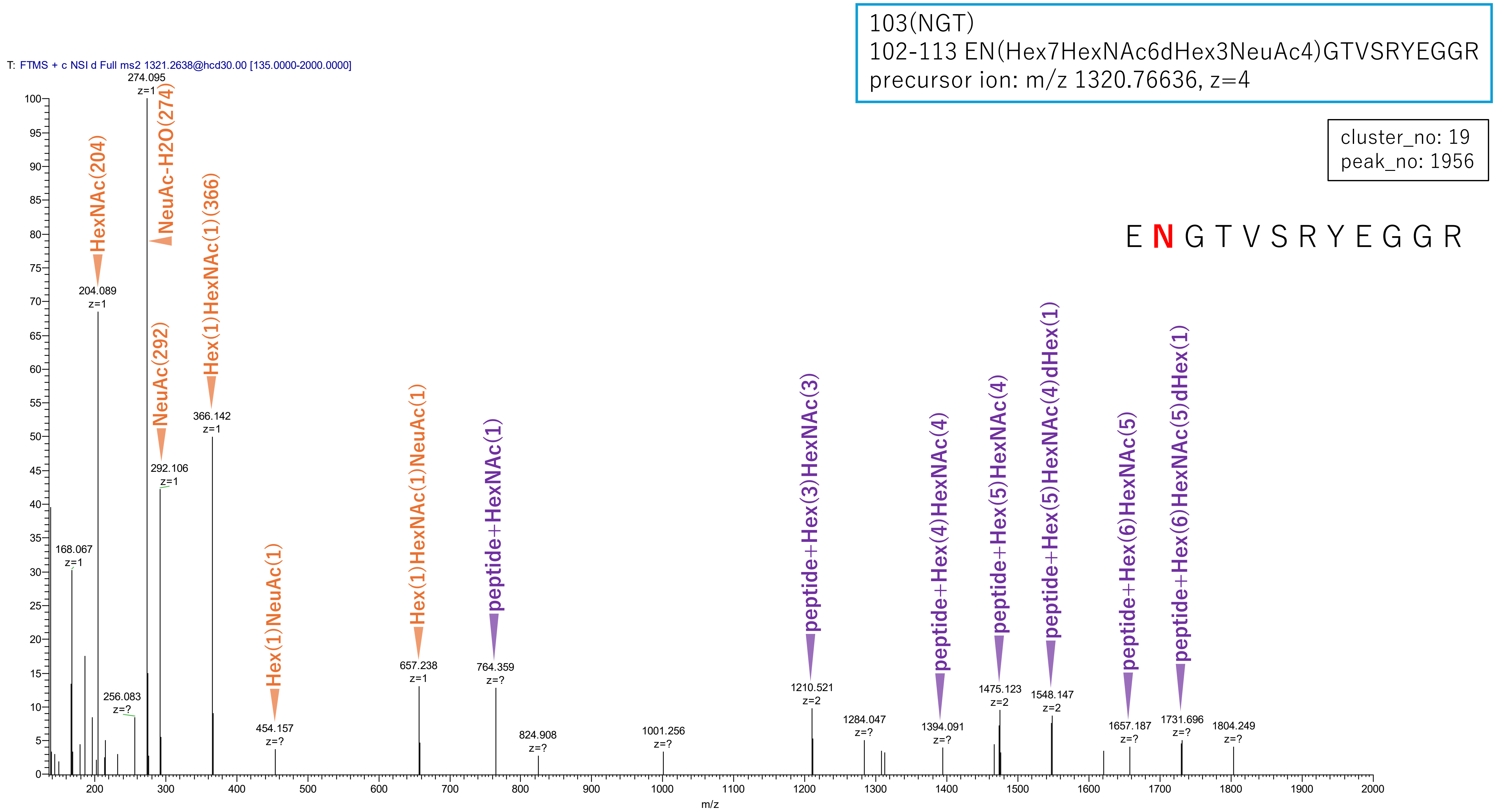

Figure S4-89. MS2 spectra of glycopeptides assigned for hAGP

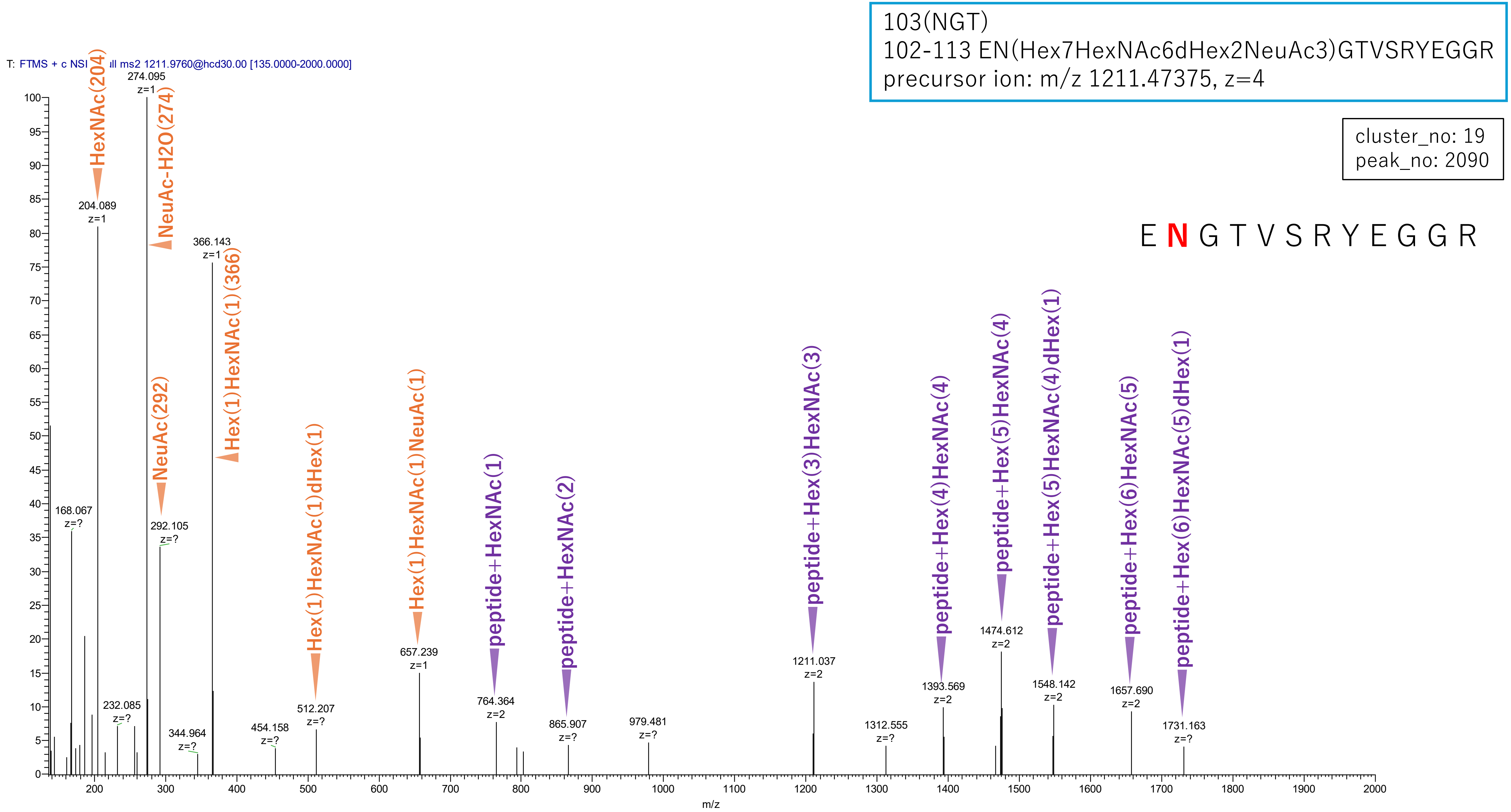

Figure S4-90. MS2 spectra of glycopeptides assigned for hAGP

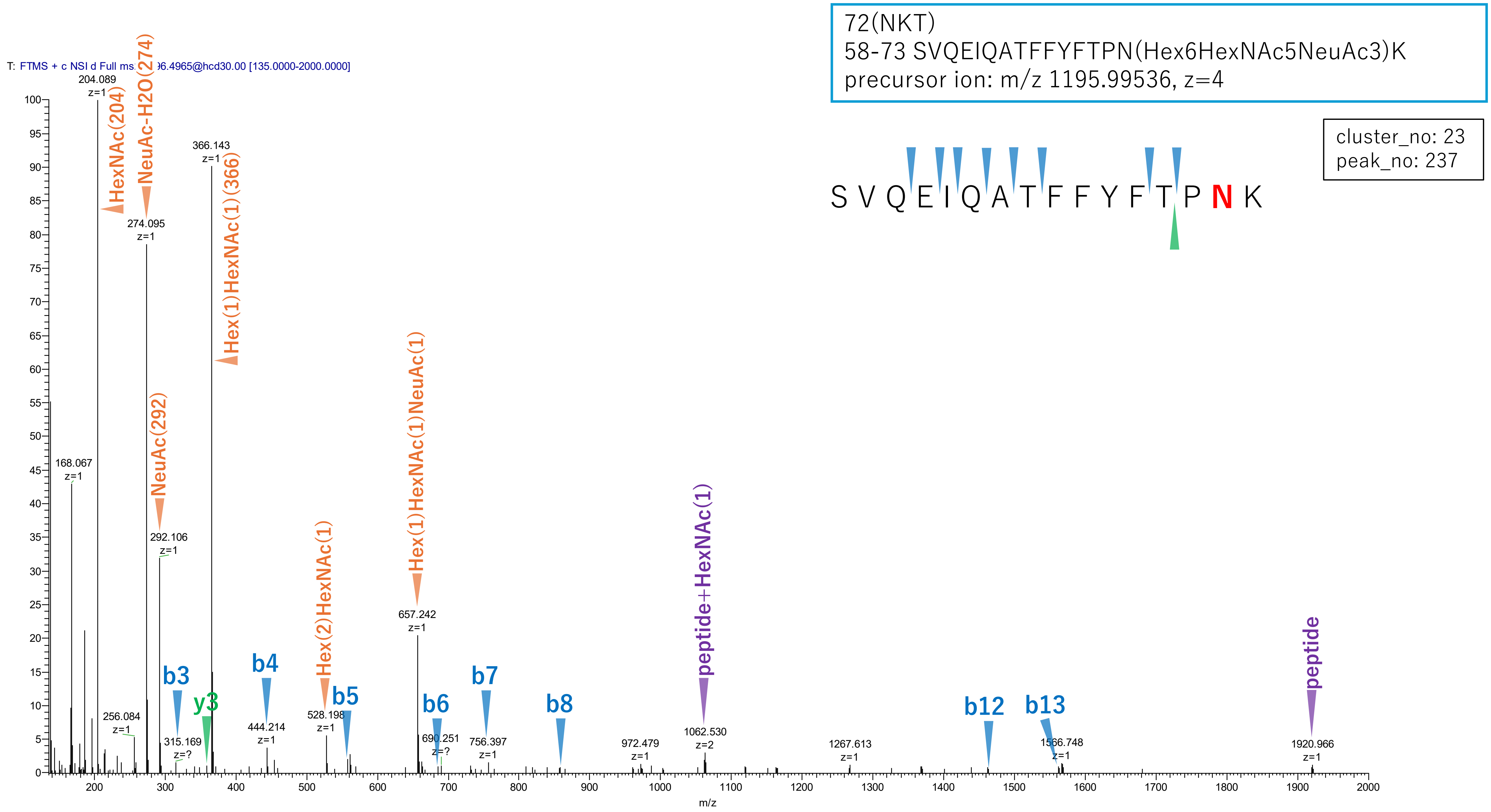

Figure S4-91. MS2 spectra of glycopeptides assigned for hAGP

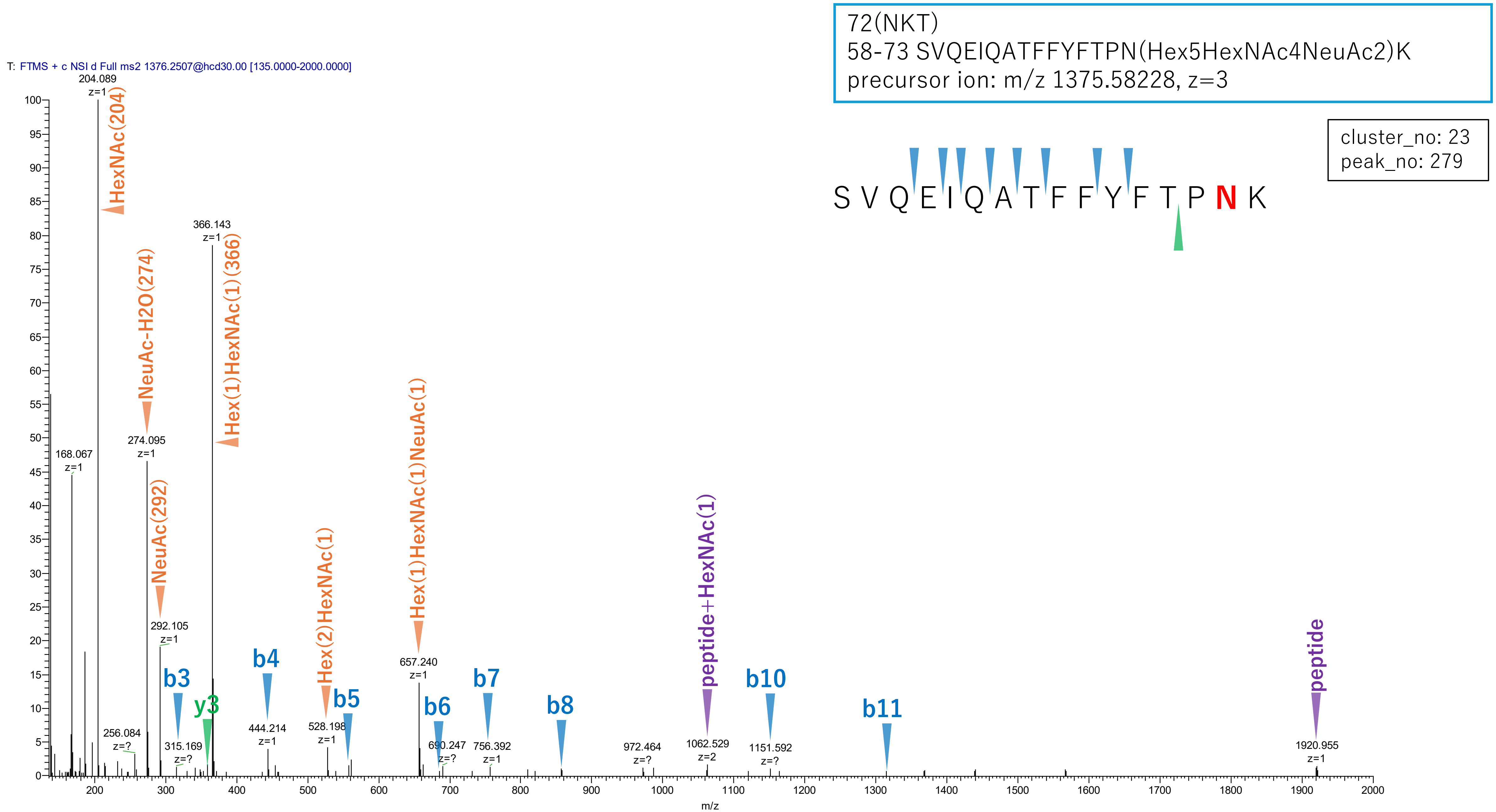

Figure S4-92. MS2 spectra of glycopeptides assigned for hAGP

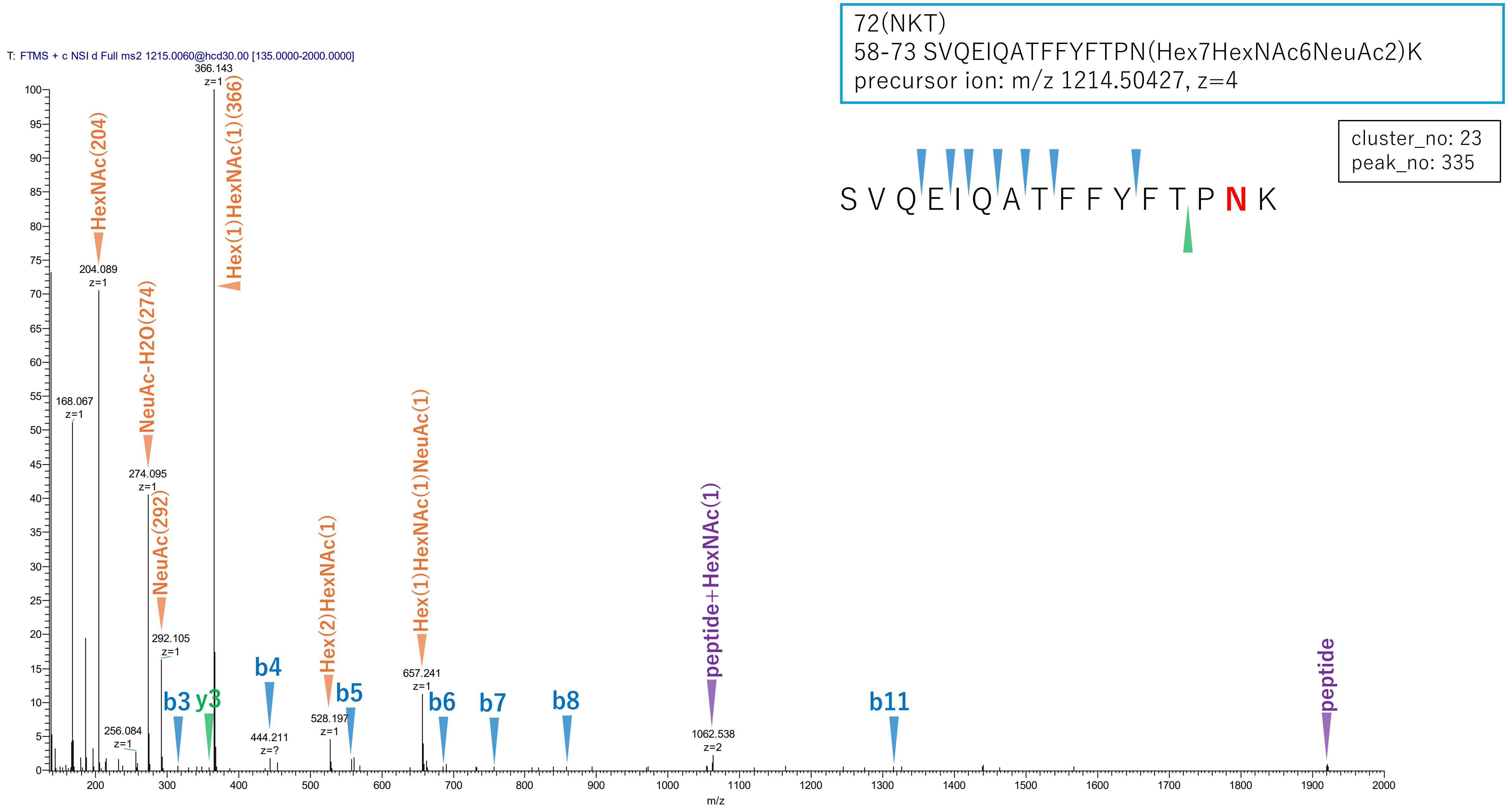

Figure S4-93. MS2 spectra of glycopeptides assigned for hAGP

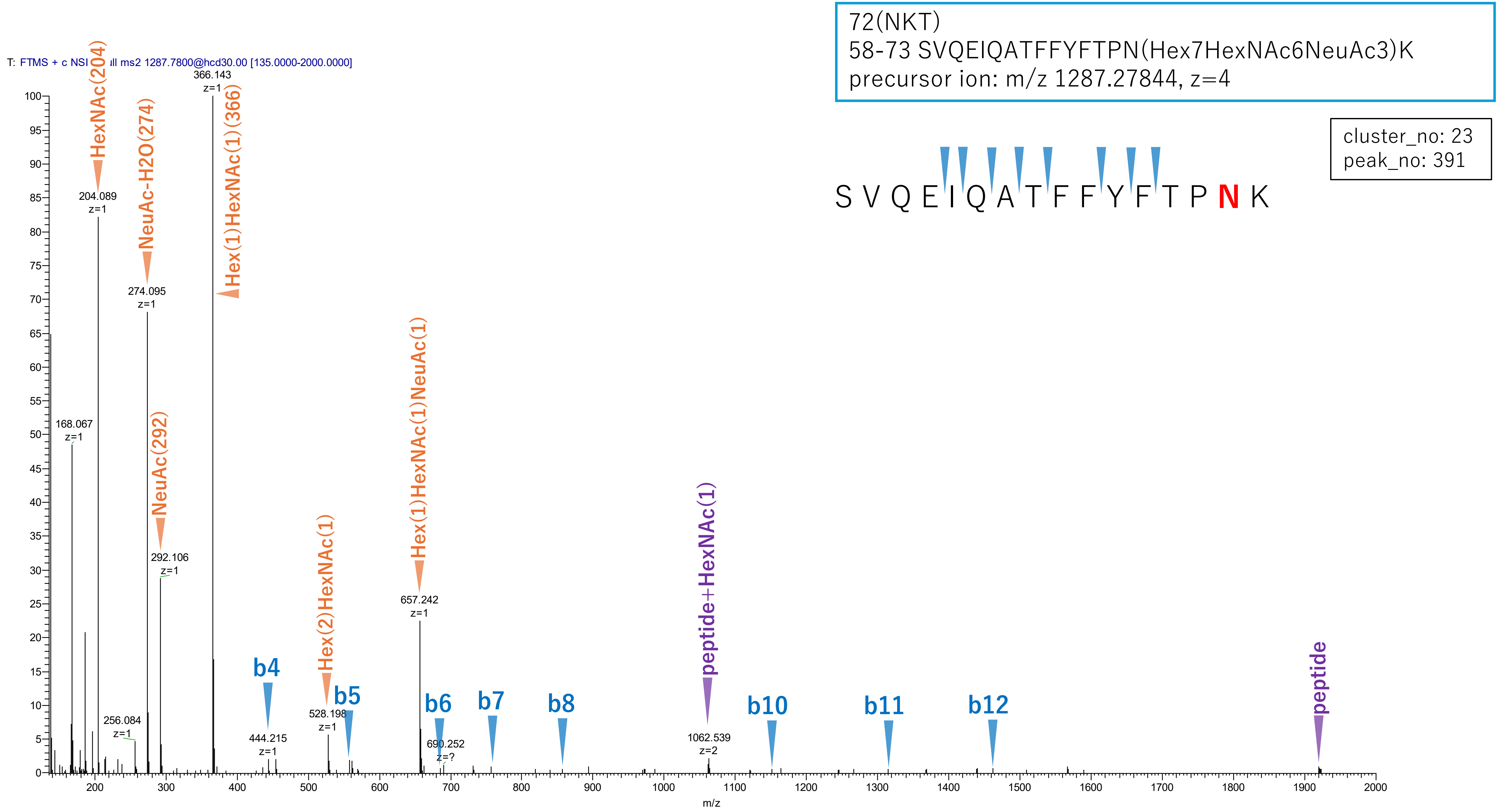

Figure S4-94. MS2 spectra of glycopeptides assigned for hAGP

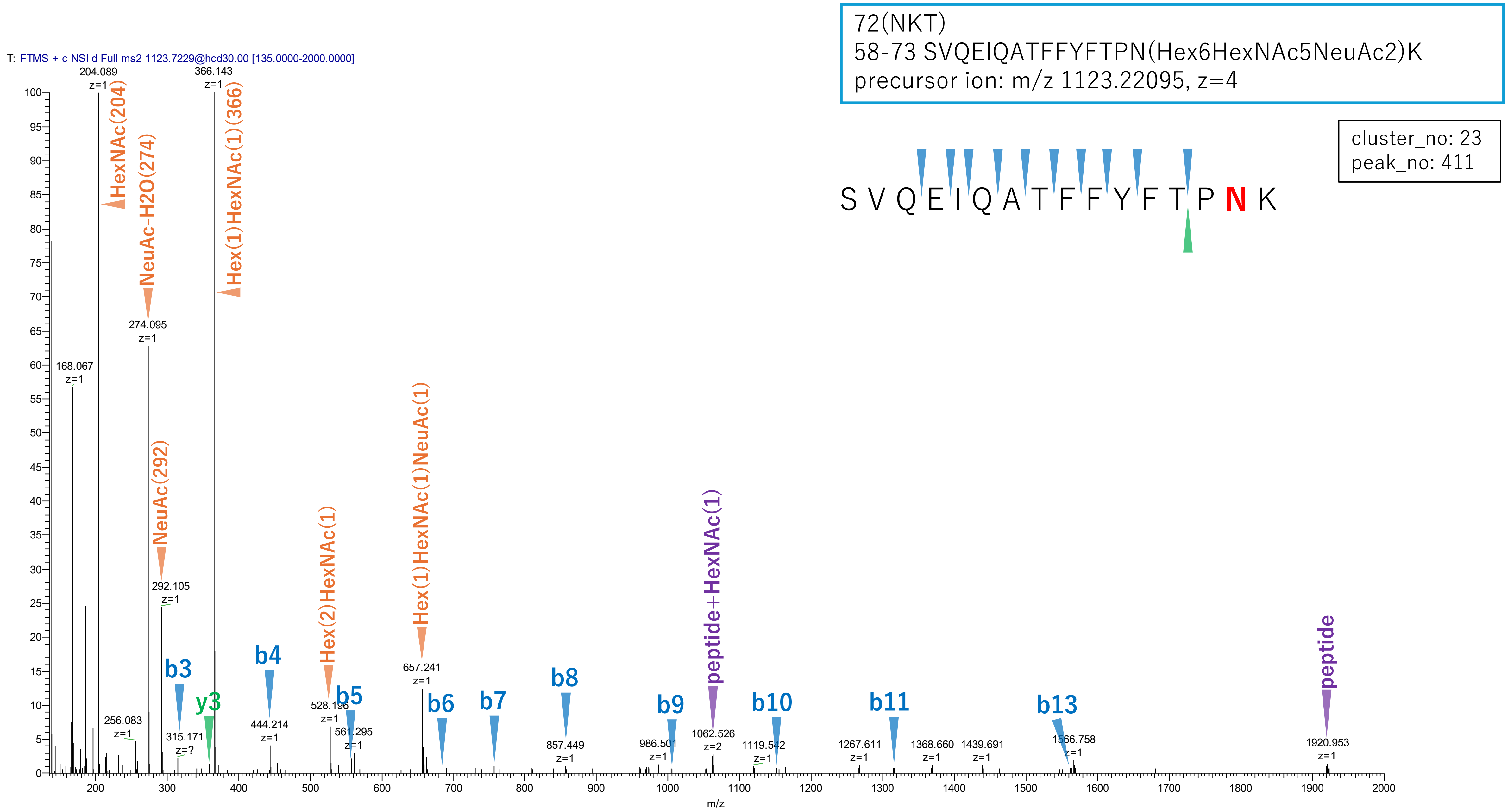

Figure S4-95. MS2 spectra of glycopeptides assigned for hAGP

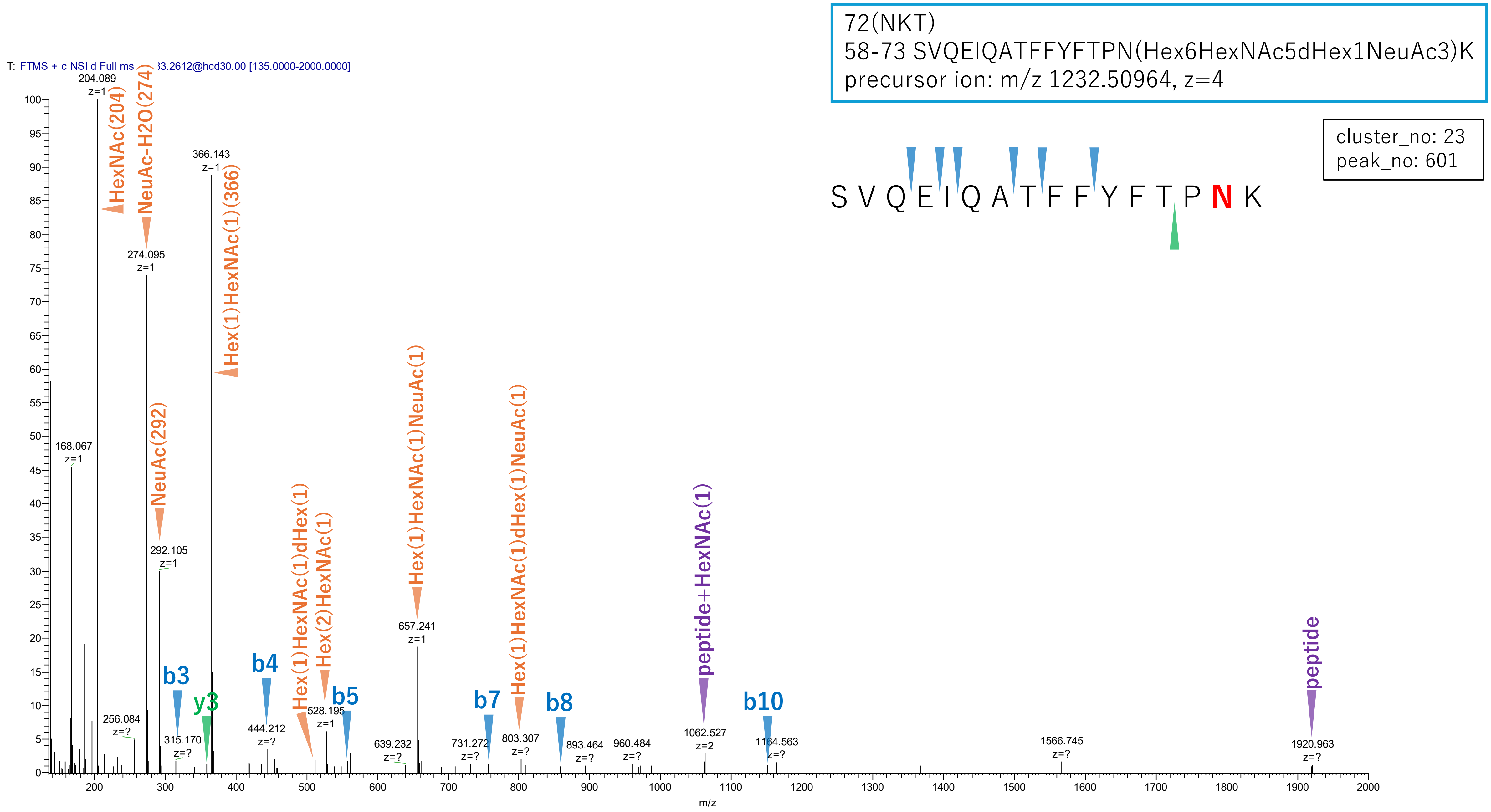

Figure S4-96. MS2 spectra of glycopeptides assigned for hAGP

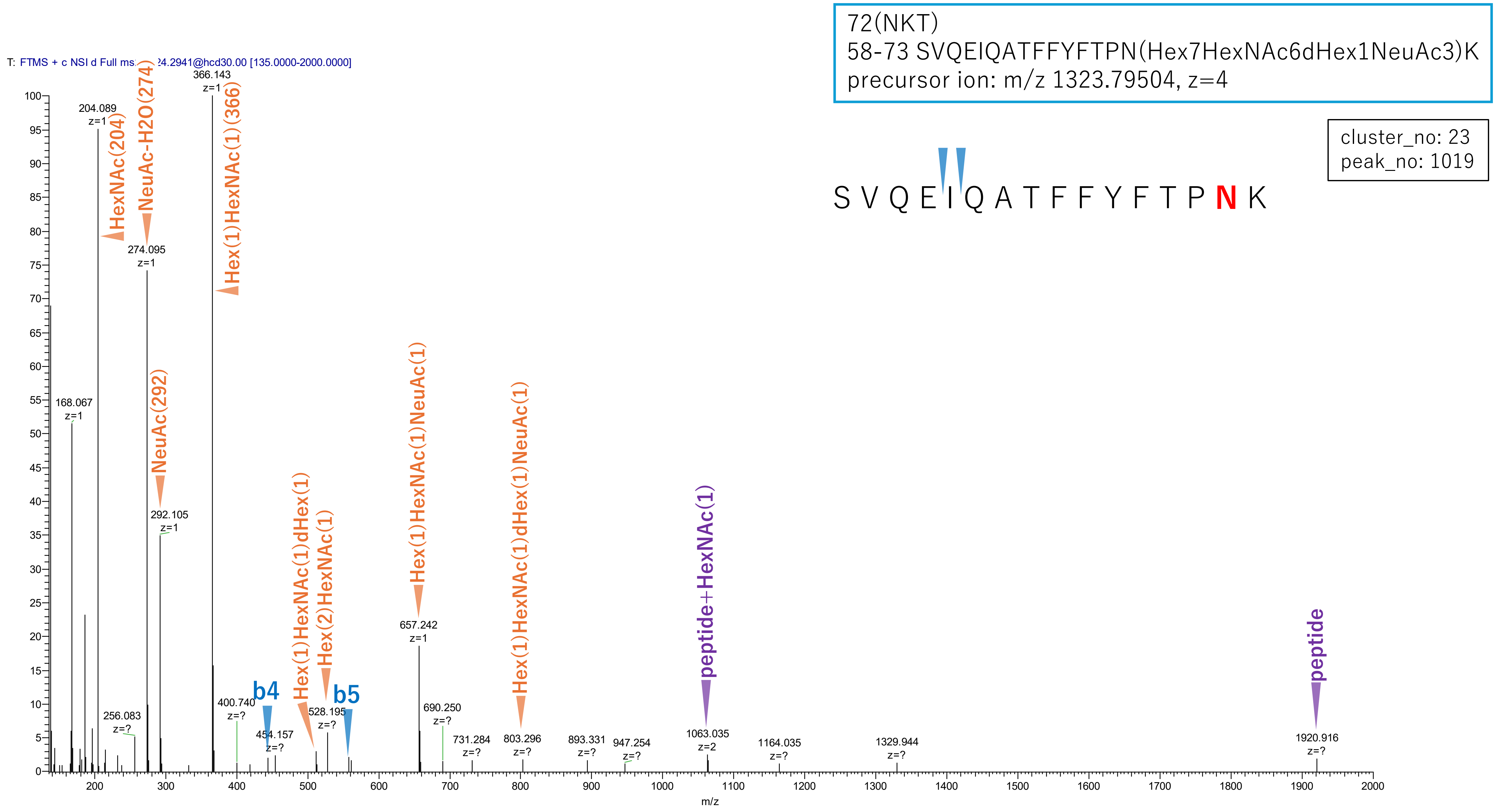

Figure S4-97. MS2 spectra of glycopeptides assigned for hAGP

103(NGT)
102-126 EN(Hex7HexNAc6NeuAc4)GTISRYVGGQEHFAHLLILRDTK
precursor ion: m/z 1062.79517, z=6

cluster_no: 26
peak_no: 266

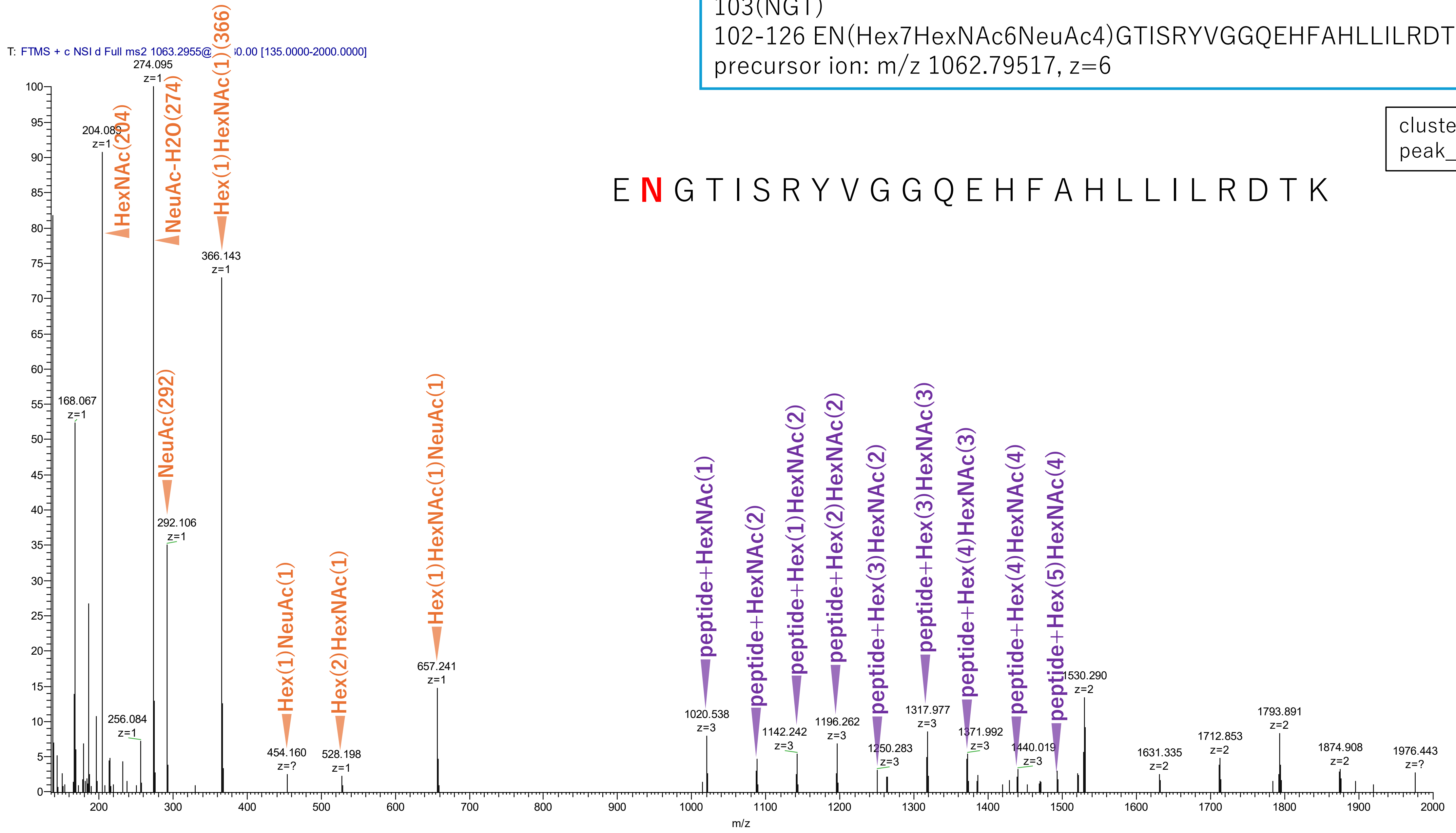

Figure S4-98. MS2 spectra of glycopeptides assigned for hAGP

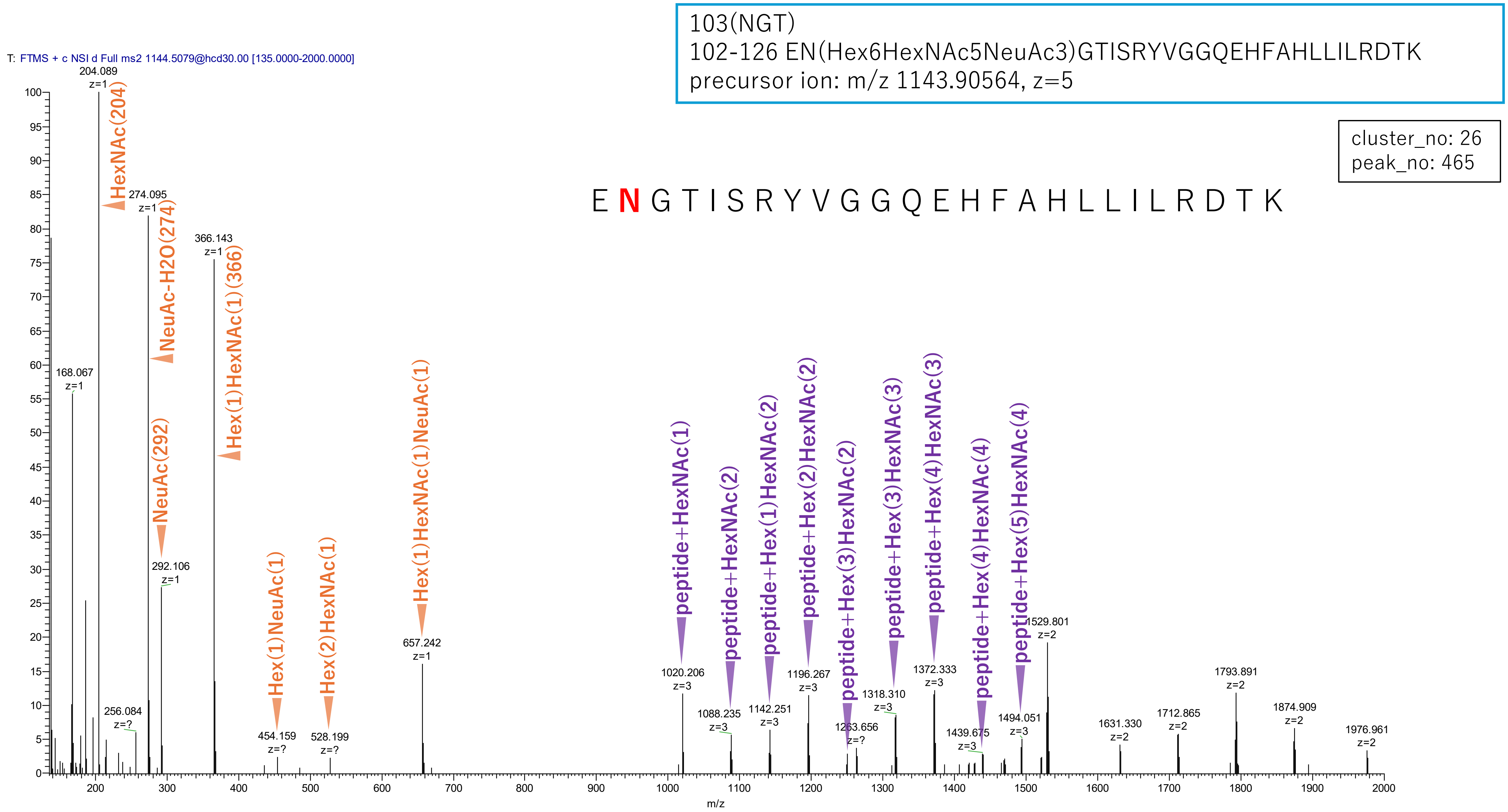

Figure S4-99. MS2 spectra of glycopeptides assigned for hAGP

103(NGT)
102-126 EN(Hex6HexNAc5dHex1NeuAc3)GTISRYVGGQEHFAHLLILRDTK
precursor ion: m/z 977.76563, z=6

cluster_no: 26
peak_no: 892

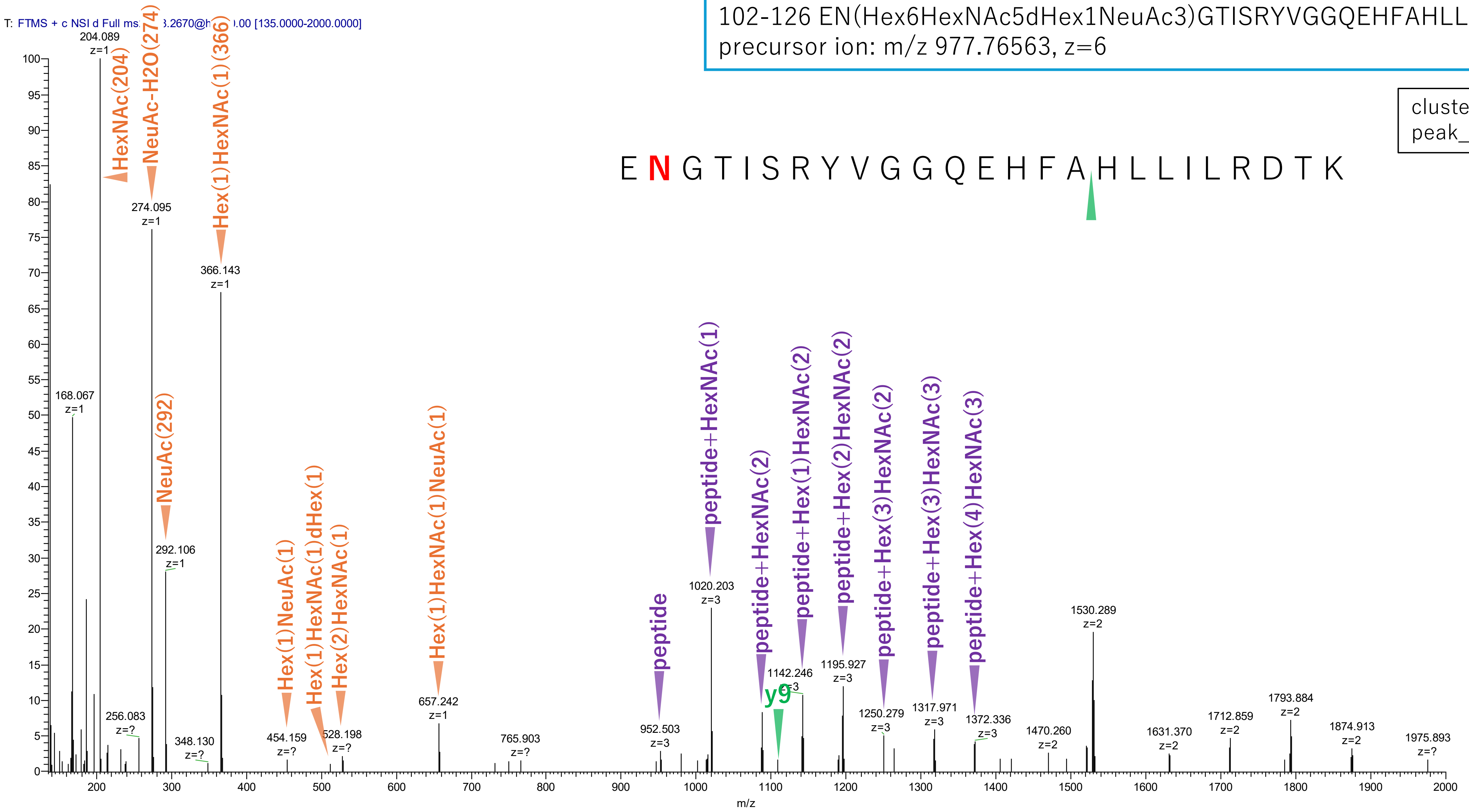

Figure S4-100. MS2 spectra of glycopeptides assigned for hAGP

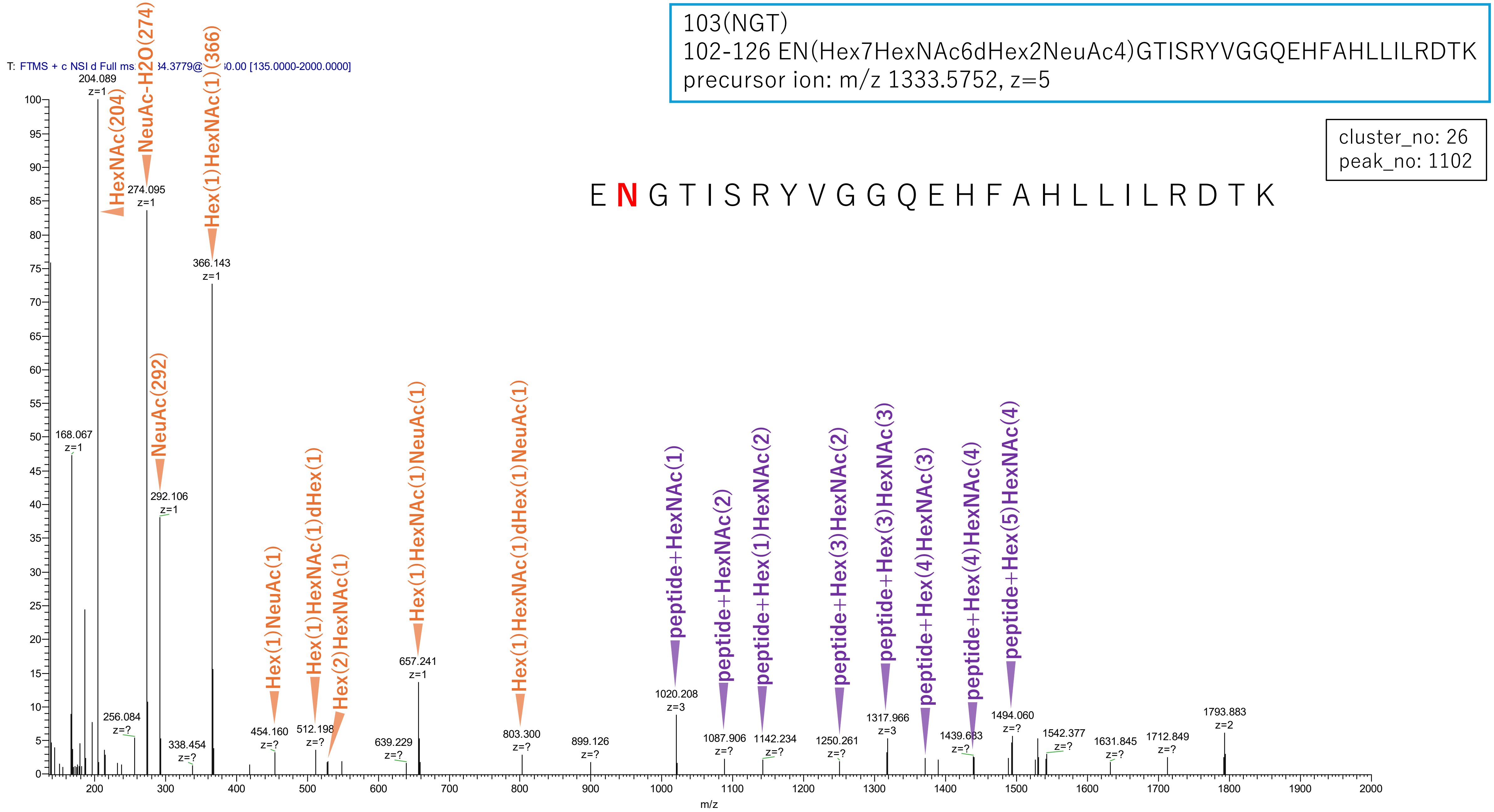

Figure S4-101. MS2 spectra of glycopeptides assigned for hAGP

103(NGT)
102-126 EN(Hex7HexNAc6dHex1NeuAc3)GTISRYVGGQEHFAHLLILRDTK
precursor ion: m/z 1246.14233, z=5

cluster_no: 26
peak_no: 1908

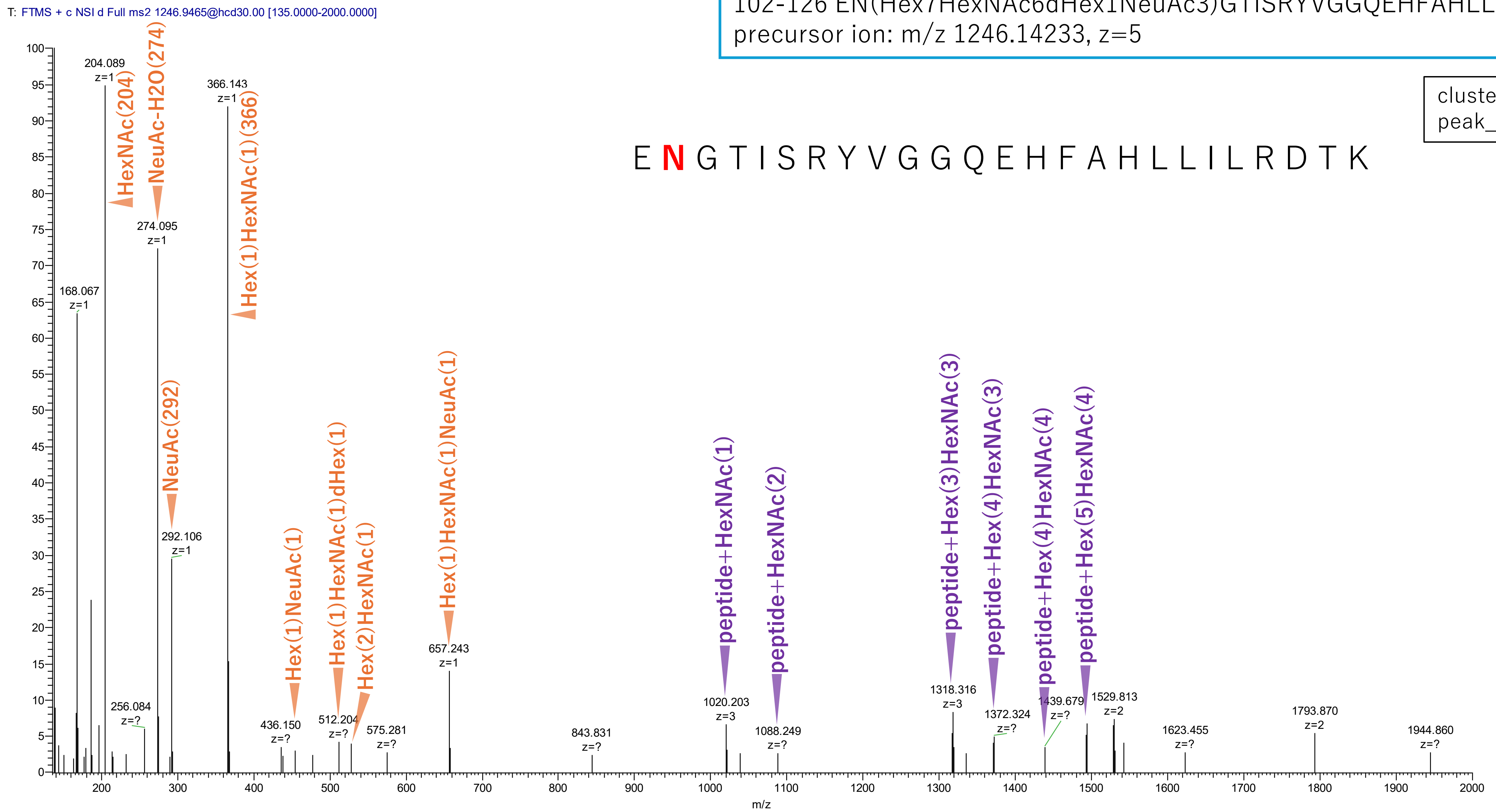

Figure S4-102. MS2 spectra of glycopeptides assigned for hAGP

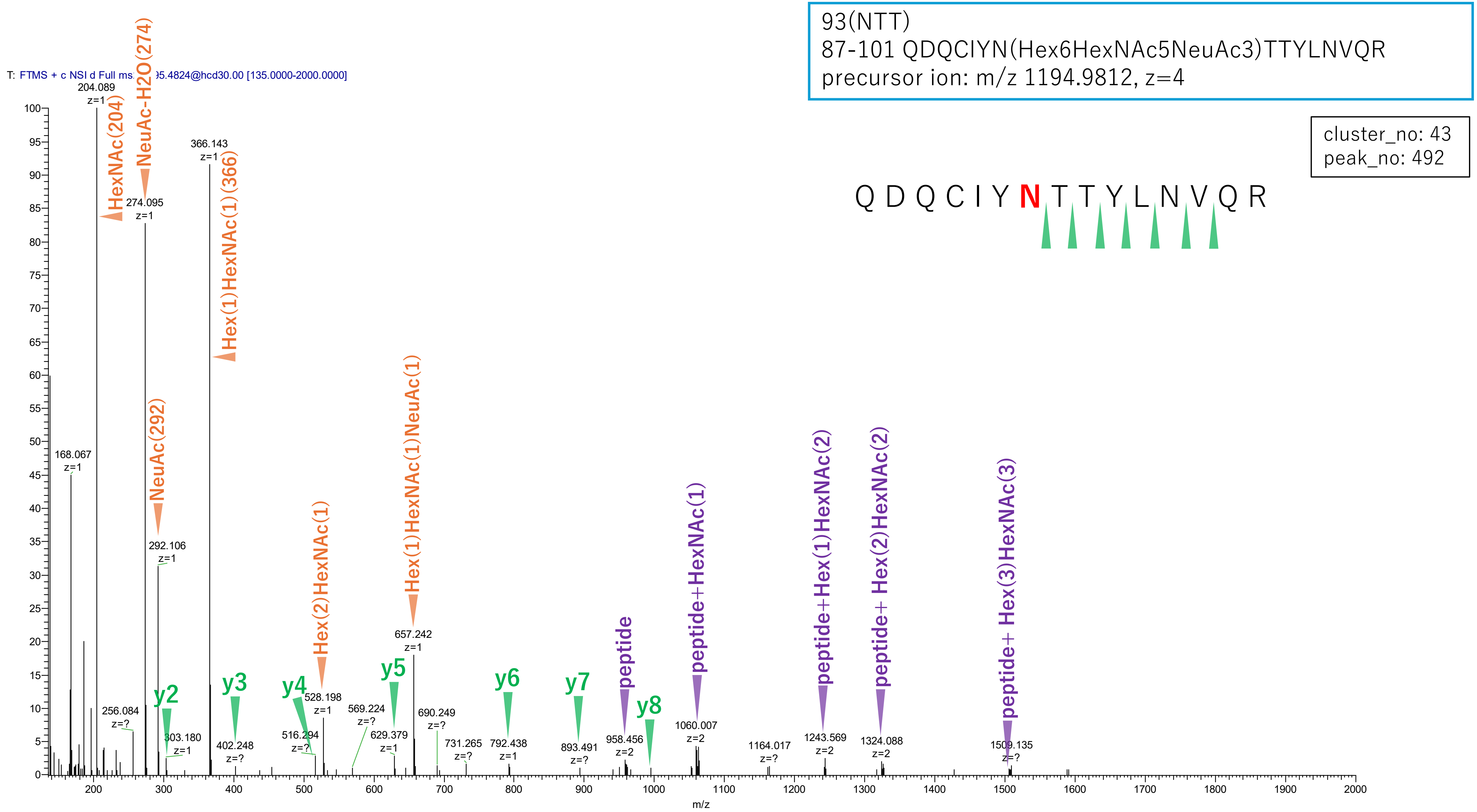

Figure S4-103. MS2 spectra of glycopeptides assigned for hAGP

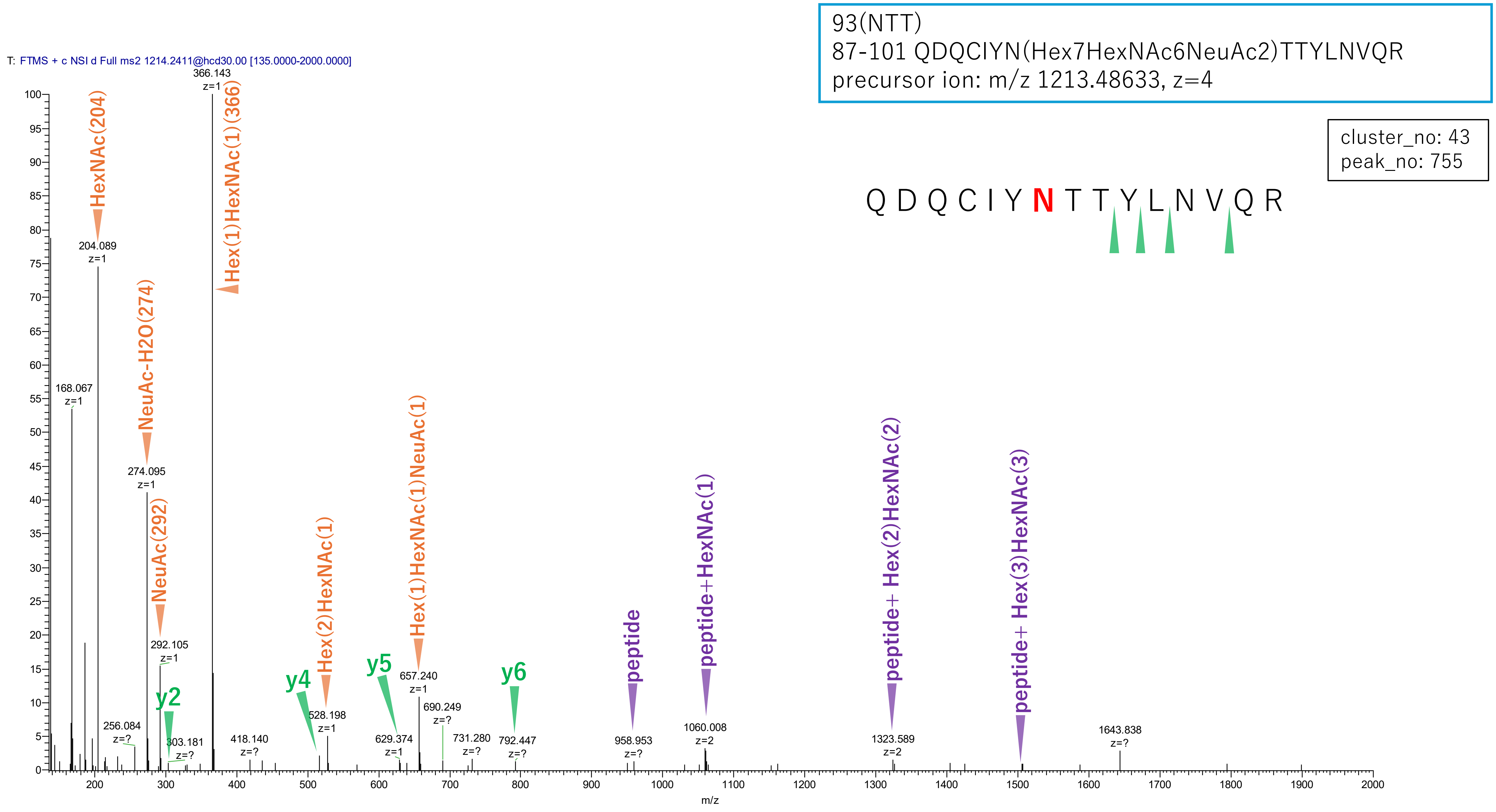

Figure S4-104. MS2 spectra of glycopeptides assigned for hAGP

93(NTT)
87-101 QDQCIYN(Hex6HexNAc5dHex1NeuAc3)TTYLNVQR
precursor ion: m/z 1231.49426, z=4

cluster_no: 43
peak_no: 969

Q D Q C I Y **N** T T Y L N V Q R

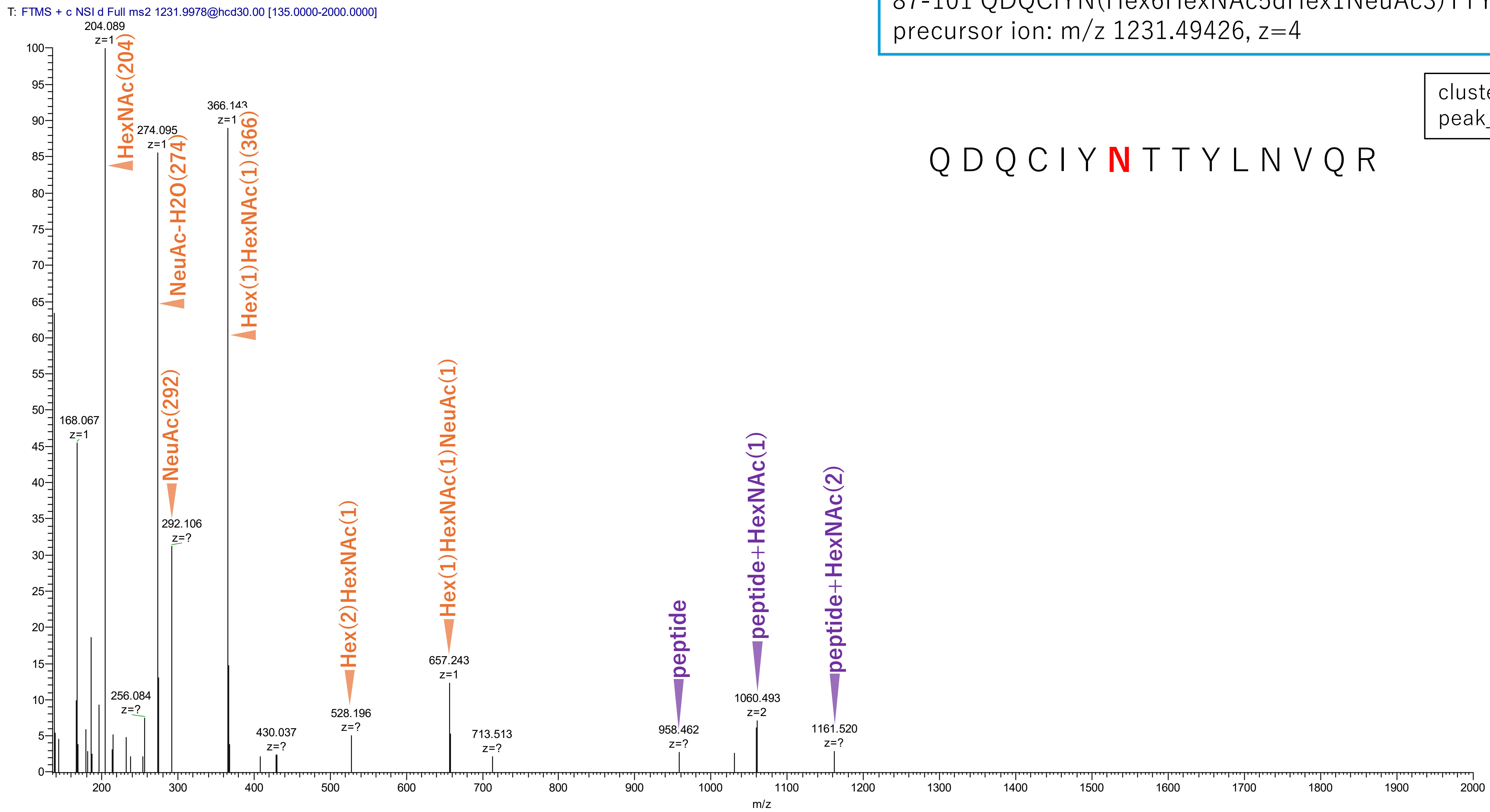

Figure S4-105. MS2 spectra of glycopeptides assigned for hAGP

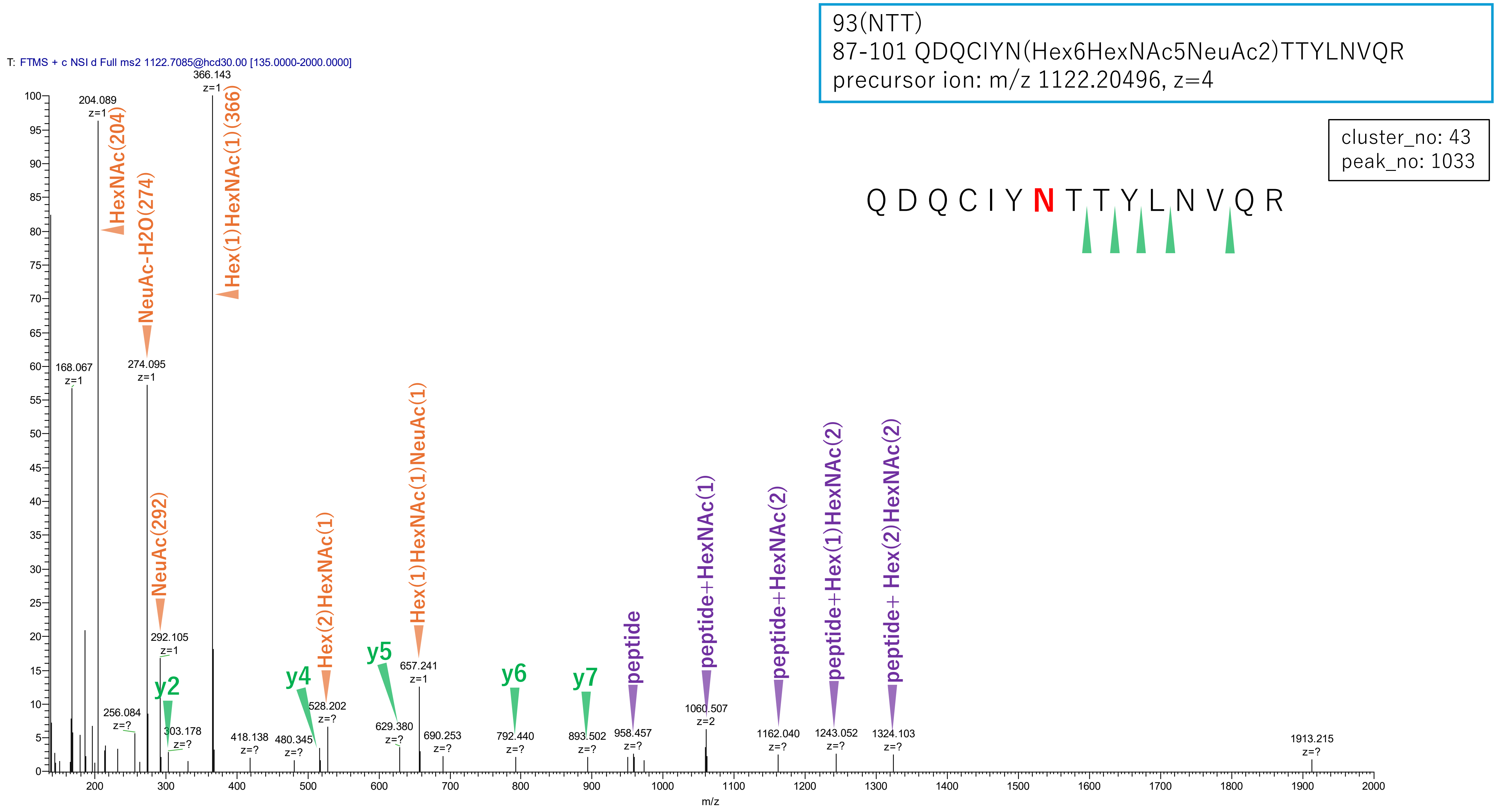

Figure S4-106. MS2 spectra of glycopeptides assigned for hAGP

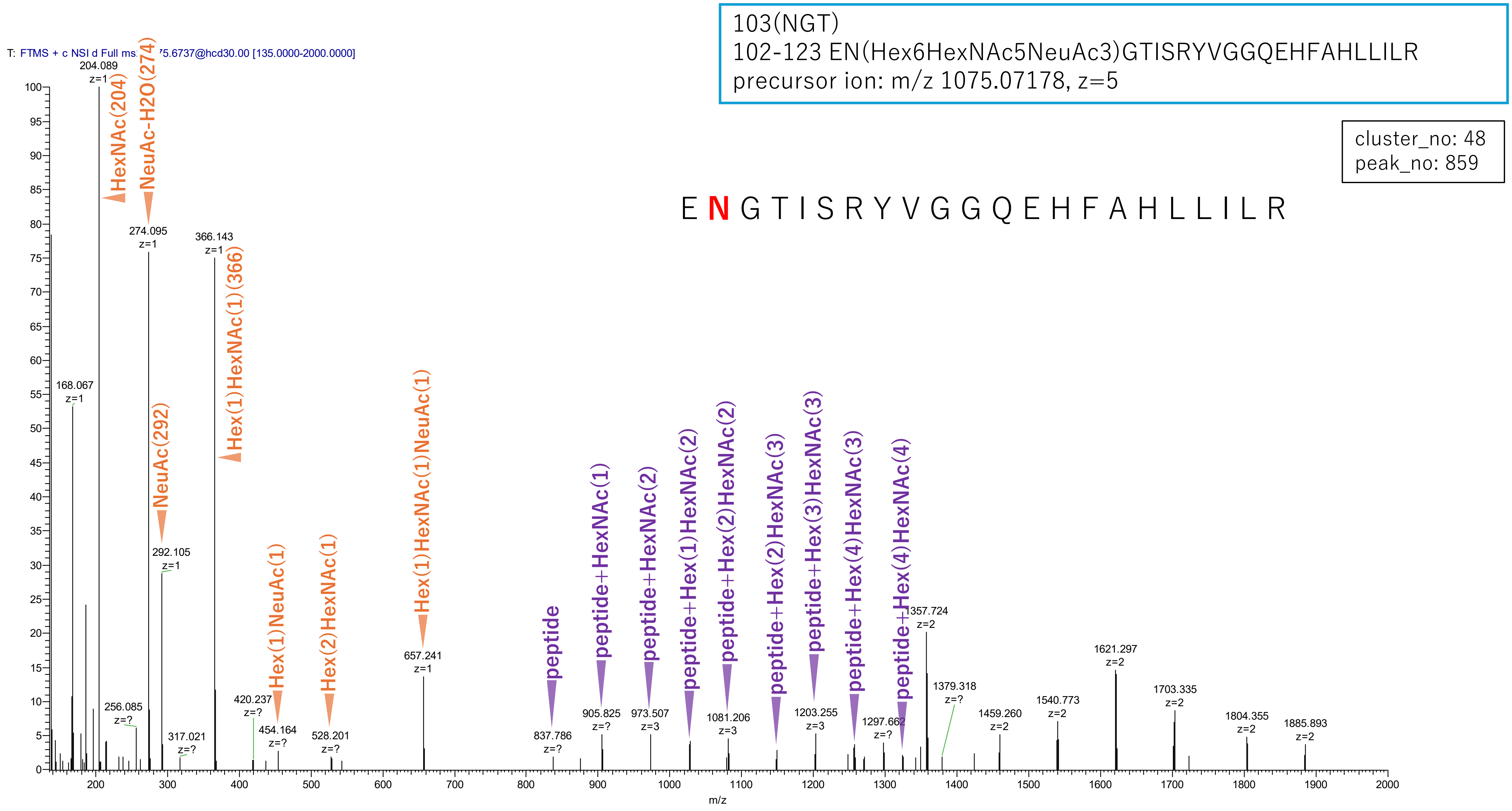

Figure S4-107. MS2 spectra of glycopeptides assigned for hAGP

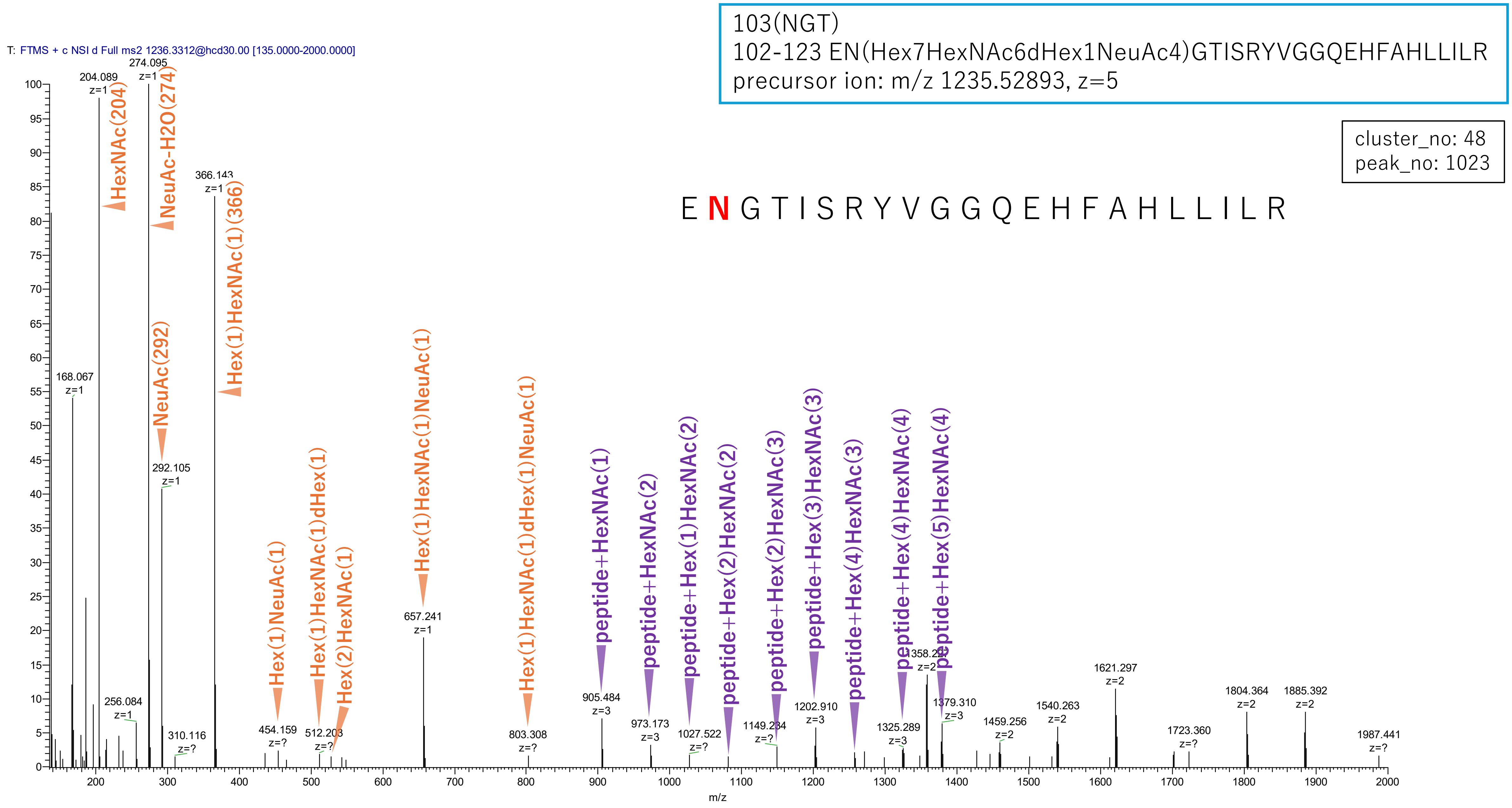

Figure S4-108. MS2 spectra of glycopeptides assigned for hAGP

103(NGT)
102-123 EN(Hex7HexNAc6NeuAc3)GTISRYVGGQEHFAHLLILR
precursor ion: m/z 1148.10168, z=5

cluster_no: 48
peak_no: 1564

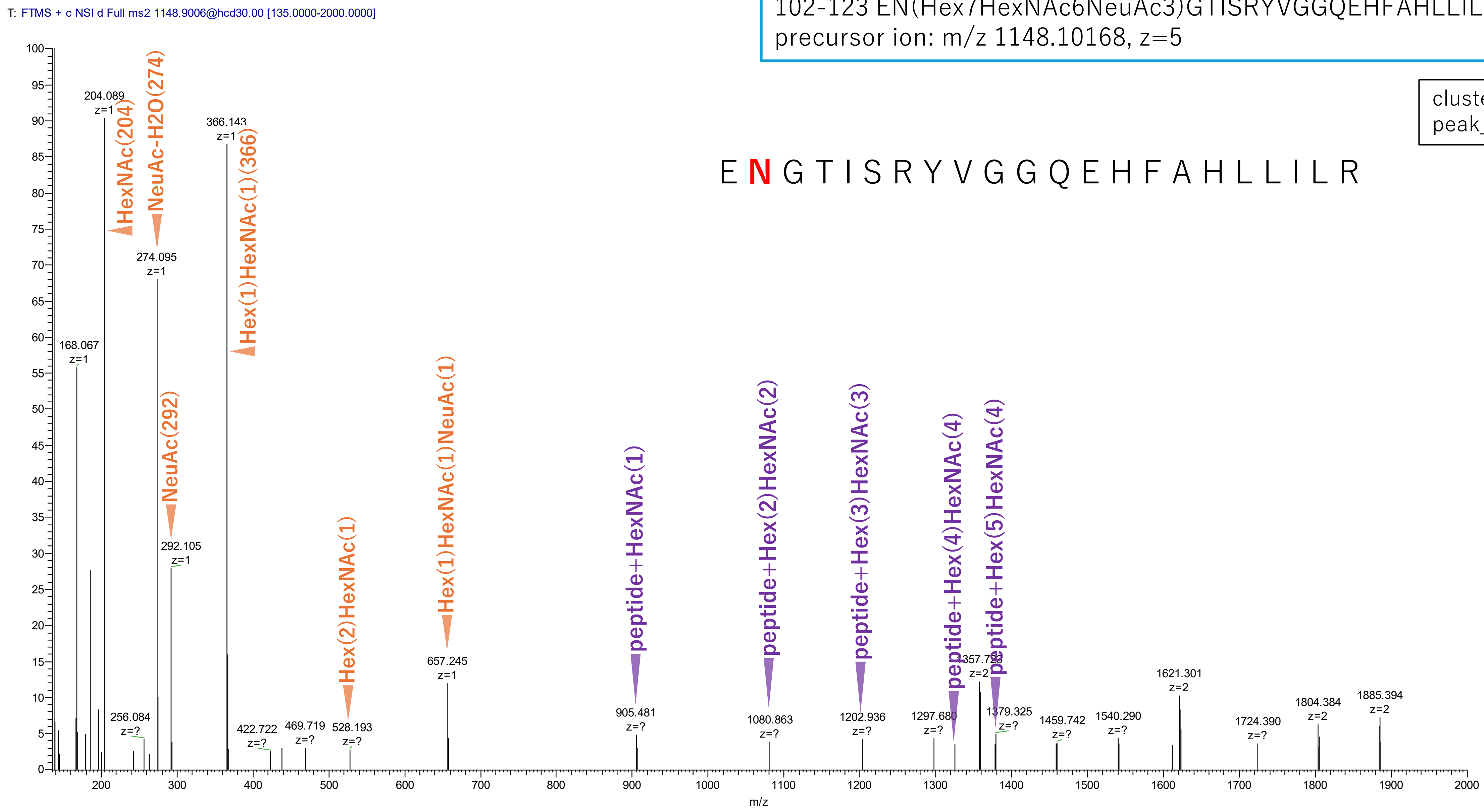

Figure S4-109. MS2 spectra of glycopeptides assigned for hAGP

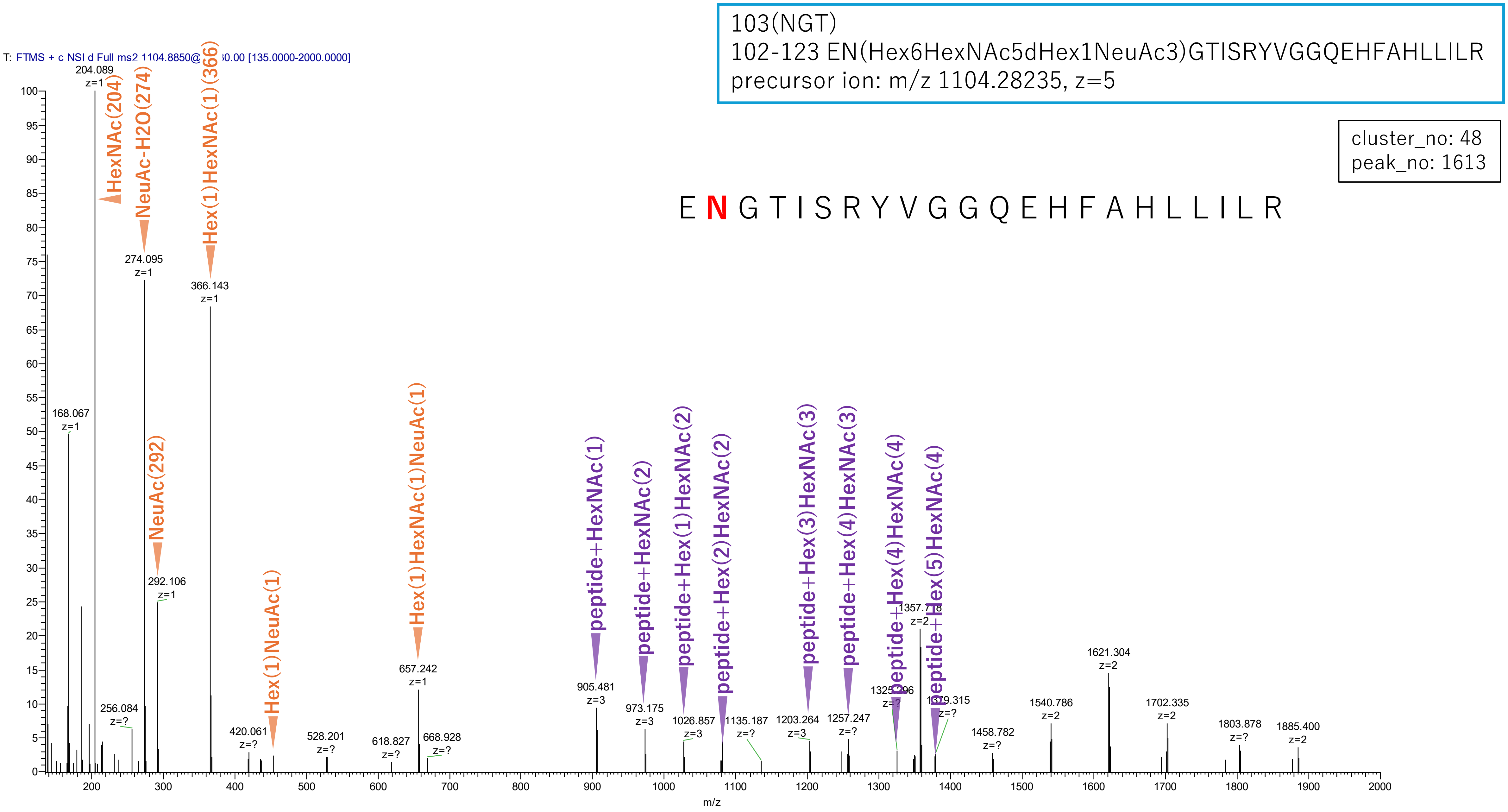

Figure S4-110. MS2 spectra of glycopeptides assigned for hAGP
