# Supplementary material for: GRable Version 1.0: A Software Tool for Site-Specific Glycoform Analysis With Improved MS1-Based Glycopeptide Detection With Parallel Clustering and Confidence Evaluation With MS2 Information
Source: Mol Cell Proteomics. 2024 Aug 23;23(9):100833. doi: 10.1016/j.mcpro.2024.100833 (PMC11421343; doi:10.1016/j.mcpro.2024.100833)
Supplement: Document S1 [file mmc8.pdf]

GRable Version 1.0

User Manual

Last update: June 11, 2024

Table of Contents

1. Outline of GRable 1
1.1. Introduction 1
1.2. Principle of the Glyco-RIDGE method 1
1.3. Contact information 1
2. System overview 2
2.1. Overview of data processing 2
2.2. Requirements for input data 2
2.3. GUI and functionality 3
3. Operation 4
3.1. Login 4
3.2. Language selection 4
3.3. Data registration (Step 1) 4
3.3.1. Functional overview 4
3.3.2. Operation procedure 5
3.4. Range setting (Step 3) 5
3.4.1. Functional overview 5
3.4.2. Operation procedure 6
3.5. Monoisotopic peak picking (Step 4) 6
3.5.1. Functional overview 6
3.5.2. Operation procedure 7
3.6. Clustering (Step 5) 9
3.6.1. Functional overview 9
3.6.2. Operation procedure 9
3.7. Matching (Step 6) 10
3.7.1. Functional overview 10
3.7.2. Operation procedure 11
3.8. Selection (Step 7) 14
3.8.1. Functional overview 14
3.8.2. Operation procedure 15
3.9. Data tree 18
3.10. Viewer 19
3.11. Monoisotopic peak list 20
3.11.1. Functional overview 20
3.11.2. Details of the correction function 22
4. Preparation details of imput files 23
4.1. Core peptide list (.xlsx) 23
4.2. Glycan point list (.xlsx) 25
4.3. LC/MS and LC/MS/MS data of glycopeptides (.mzML and .mgf) 25
5. Details of export files 26

1. Outline of GRable

1.1. Introduction

GRable is a freely available online tool to find site-specific glycoforms of glycopeptides. This tool is unique in that it utilizes an MS1-based glycoproteomic method named "Glyco-RIDGE" (Glycan heterogeneity-based Relational Identification of Glycopeptide signals on Elution profile).

1.2. Principle of the Glyco-RIDGE method

First, this method identifies glycopeptide signals based on the chromatographic properties of glycopeptides and mass differences due to the glycan heterogeneity. That is, glycopeptides having the same core peptide but different glycans elute within a narrow range of retention time (RT), so glycopeptide signals with a similar RT and mass differences corresponding to the masses of glycan units can be assigned as a cluster, without MS2 spectrum analyses. In parallel, core peptides present in the glycopeptide sample are identified by IGOT-LC/MS/MS. Then, considering that the mass value of glycopeptide is the sum of those of core peptide and glycan, the combination of peptide and glycan for each glycopeptide is searched from these three lists: the mass and RT lists of glycopeptides, peptides, and glycans).

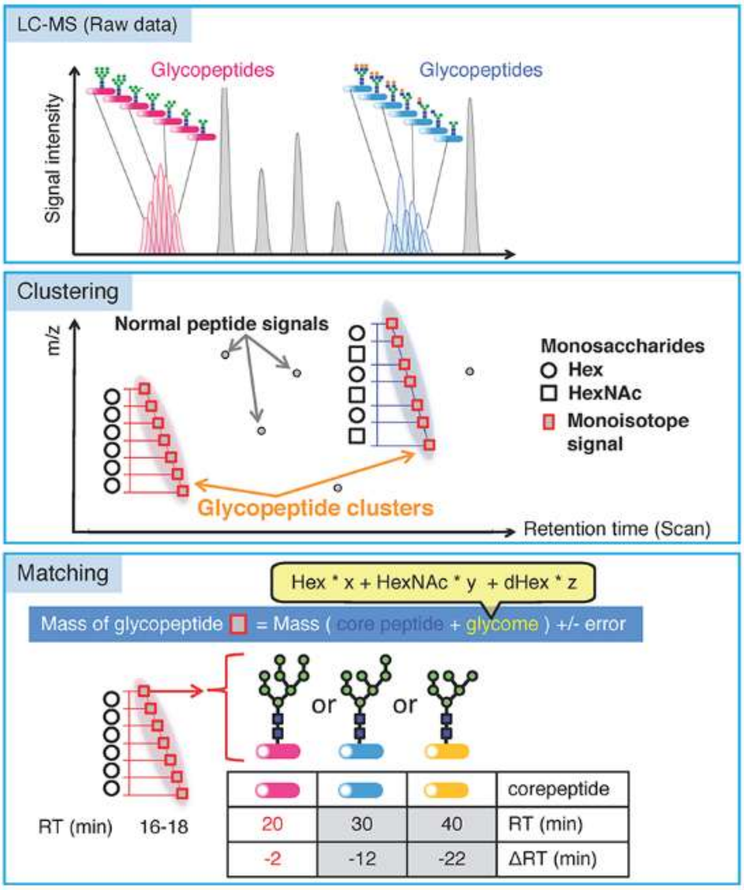

Figure 1. Overview of the Glyco-RIDGE method

(Narimatsu *et al. J Proteome Res,* 2018)

1.3. Contact information

If you belong to an academic research institute, you can use the full version without functional limitations by concluding a joint research agreement between your organization and AIST.

Please contact us: M-GRable-inquiry-ml@aist.go.jp.

2. System overview

2.1. Overview of data processing

This software proceeds along seven steps. In the GRable Version 1.0, Step 2 (deconvolution) is currently unapplicable. Each step except for Step 2 is executed step-by-step after uploading required data and search parameter settings at a main window of the software. The results of Steps 3 to 5 can be visually confirmed at a viewer of the main interface. Detailed results of Steps 4 to 7 can be exported as an Excel file with each setting.

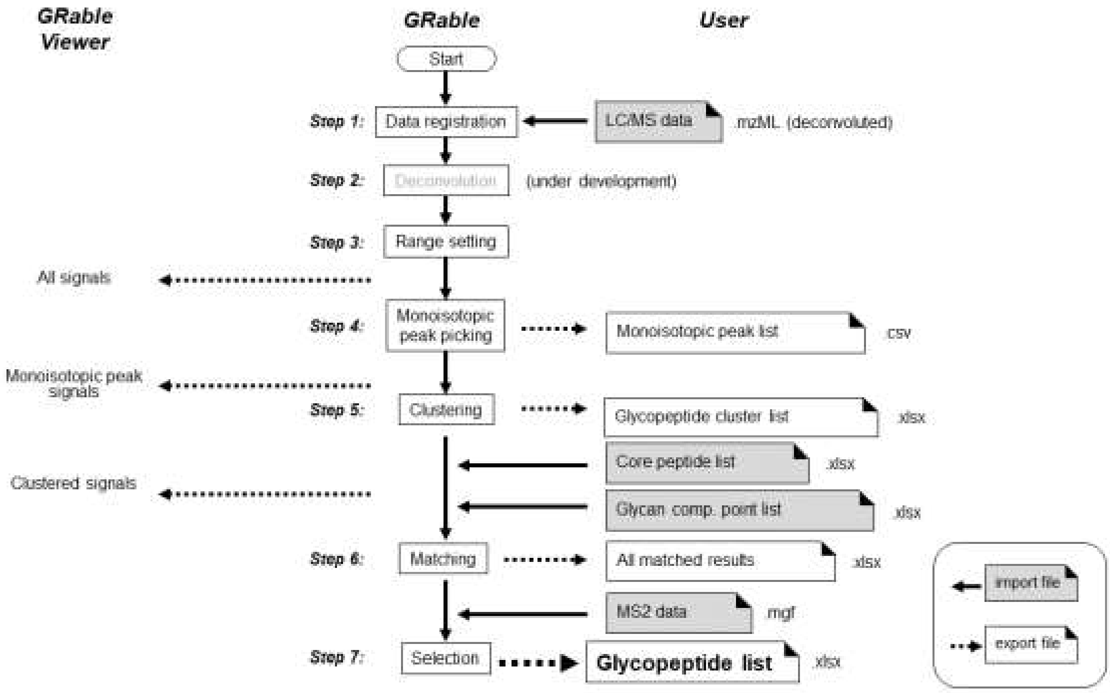

Figure 2-1. Overview of data processing by GRable.

2.2. Requirements for input data

GRable requires the following 4 files [Note 1]. The details of input files are described below (Section 4.).

1) **LC/MS data of glycopeptides (.mzML):** The LC/MS data (.raw) should be deconvoluted and exported as an mzML format using Proteome Discoverer. ([Note 2, 3])
2) **Core peptide list (.xlsx):** This list contains information on existing core peptides identified by PNGase-mediated deglycosylation followed by LC/MS analysis. ([Note 4])
3) **Glycan point list (.xlsx):** This list is used for giving a point to individual member according to matched glycan compositions. Score of a cluster is sum of points obtained by each cluster member. The score will be used primarily to evaluate matching result for the cluster. It is possible to give high points to compositions with a high probability of existence, and to give penalties to compositions that are biosynthetically impossible.
4) **LC/MS/MS data of glycopeptides (.mgf):** Peak list of MS2 spectra extracted from the raw data of 1).

Notes

1. All data should be less than 300 MB each.
2. GRable is developed using Thermo Scientific data. Therefore, data from other manufacturers' equipment are currently not guaranteed. Also, LC/MS using C18 column is assumed.
3. The analytical data require a mass accuracy better than 5 ppm. This is because GRable assigns glycopeptide signals using the accurate mass difference between glycopeptides with the same core

peptide and estimates glycan composition based on the mass difference between the core peptide and the glycopeptide. Low mass accuracy increases the possibility of incorrect matches.

4. This analysis should preferably be performed under the same LC conditions as the measurement of glycopeptide sample. In the case of highly purified protein, presumed peptide sequences can be included in the list.
5. By the GRable algorithm, multiple core peptides may match to single cluster. Then, it uses some surrounding information to select the most plausible match among them, e.g., sum of glycan points of cluster member, RT difference with core peptide, signal intensity of core, etc. In addition, if a cluster member has acquired MS2, the soft will use that information.
6. In the future, we plan to develop an updated version that can handle data from manufacturers other than Thermo Scientific, and that allows individual user management.

2.3. GUI and functionality

Figure 2-2 shows the main window of the GRable Version 1.0 with the numbers of functions listed in Table 2-1. Note that functions that are present but not described in this manual are under construction and thus not guaranteed.

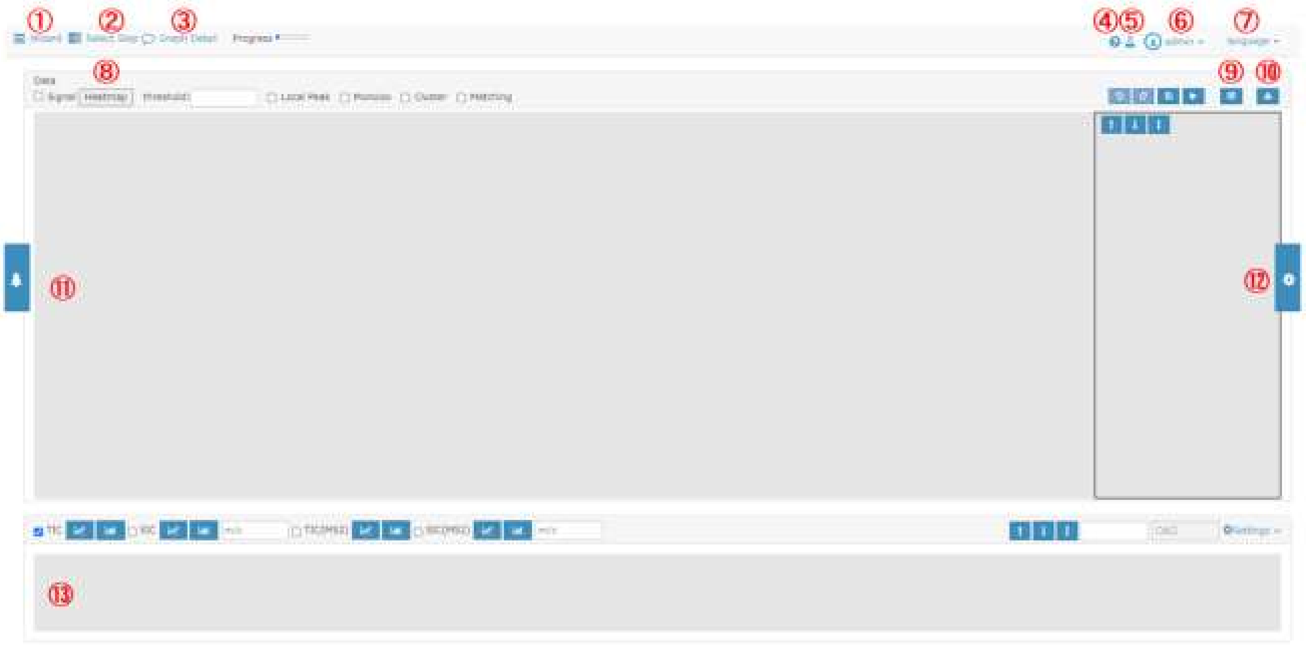

Figure 2-2.　Main window.

Table 2-1.　List of functions.

Function No. is the same shown in Figure 2-2.

No.	Name of function	Description
1	Wizard	Select step you want to do in the module.
2	Select step	Select step you want to do in the dropdown list.
3	Graph Detail	Displays setting parameters of the displayed graph.
4	System information	Displays system update information.
5	Monoisotopic Mass Table	Displays a Monoisotopic Mass Table.

No.	Name of function	Description
6	User/project management	Manages users and projects and log out.
7	Language	Selects display language (Japanese or English).
8	Heatmap	Displays signal intensity as a heatmap.
9	Option	Displays 3D display or monoisotopic peak list.
10	Download	Downloads graph image data, clustering analysis results, and matching results.
11	Tree	Displays data and analysis results in a tree format and performs display, analysis, and reanalysis.
12	Settings	Displays the setting panel for heatmap operations (enlargement/reduction, etc.).
13	Chromatograms	Displays the Chromatograms graph.

3. Operation

3.1. Login

To log in to the system, you need to enter a login ID and password on the login window (Figure 3-1). When both are appropriate, you can low in the software.

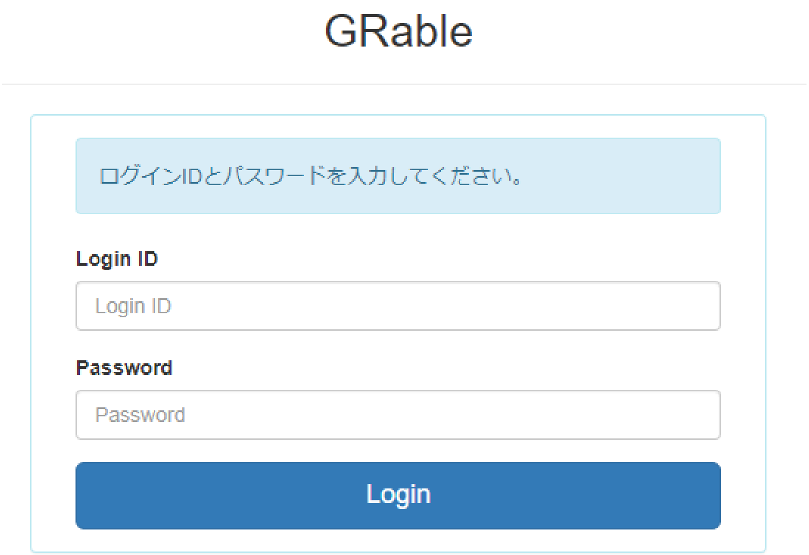

Figure 3-1. Login window.

3.2. Language selection

The display can be switched between in Japanese and English using the language selection menu (Figure 2-2⑦) on the upper right of the main window.

3.3. Data registration (Step 1)

3.3.1. Functional overview

GRable allows the registration of LC/MS data of glycopeptides in mzML format, which ensures great versatility and ease of data processing. This software is also designed to deal with charge-deconvoluted and centroid spectra of each scan. This software is tested and optimized using the mzML data prepared with

Proteome Discoverer (version 2.4 or later; Thermo Fisher Scientific), in which deconvolution is performed with a workflow consisting of Xtract. To do the Glyco-RIDGE analysis, high accuracy of mass measurement is strongly required, e.g., mass resolution of MS1 by orbitrap analyzer is 120,000 or 240,000 (at m/z 200) and a lock mass at 445.12003 is necessary.

3.3.2. Operation procedure

To register data for analysis, press the wizard button (Figure ①) on the main window to display the data registration module (Figure 3-2).

① Select the type of data to be registered. Select “LC/MS” for uploading mzML format files after deconvolution.
② Enter the analysis data set name.
③ Select data. After data uploading, the check will be added.
④ Press the Register button.
⑤ When "Registration completed" appears, the process is finished. Proceed the next step.
⑥ If you want to cancel this step in progress, press the "Cancel" button.

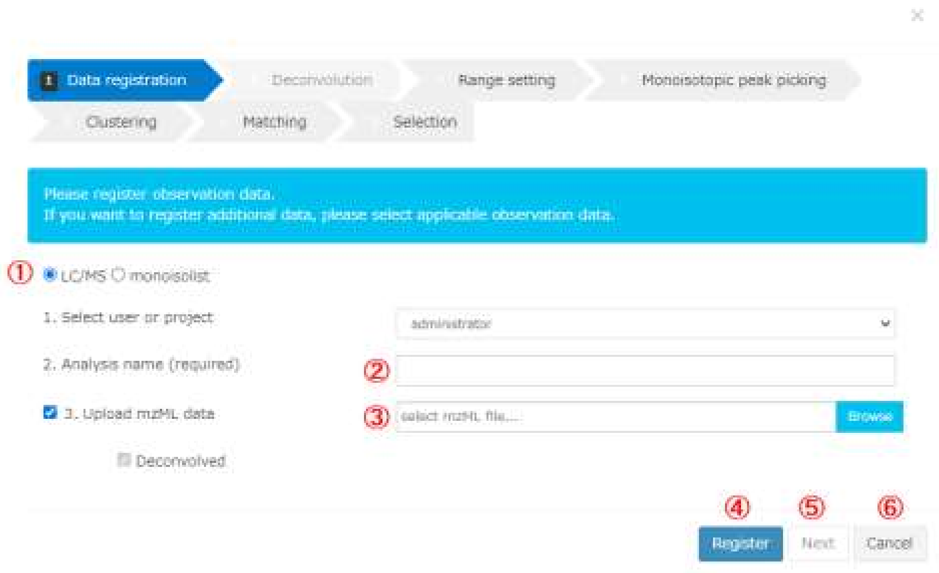

Figure 3-2. Data registration module.

3.4. Range setting (Step 3)

3.4.1. Functional overview

This step (Figure 3-3) is designed to set the RT range, mass range, and minimum signal intensity (threshold) over which the analysis will be performed. Analysis for a data of long gradient elution and broad mass ranges are time consuming. The signals selected in this step (all signals) can be seen as a heatmap in the viewer main window. The resulting peak list is exported as a CSV (comma-separated values) file because of the huge size of the data. (In the subsequent steps, the setting parameters and the results were exported as an Excel (.xlsx) file.)

3.4.2. Operation procedure

To perform range setting, press the wizard button (Figure 2-2①) from the main module to display the range setting module (Figure 3-3).

① Enter an analysis name.
② Set parameters (Table 3-1). Note that "Load settings" is unapplicable in this version.
③ Select whether the setting is saved.
④ Click the "Run" button to start analysis.
⑤ When "Range setting completed" is displayed, the process is finished. Please proceed the next step.
⑥ If you want to cancel this step in progress, press the "Cancel" button.

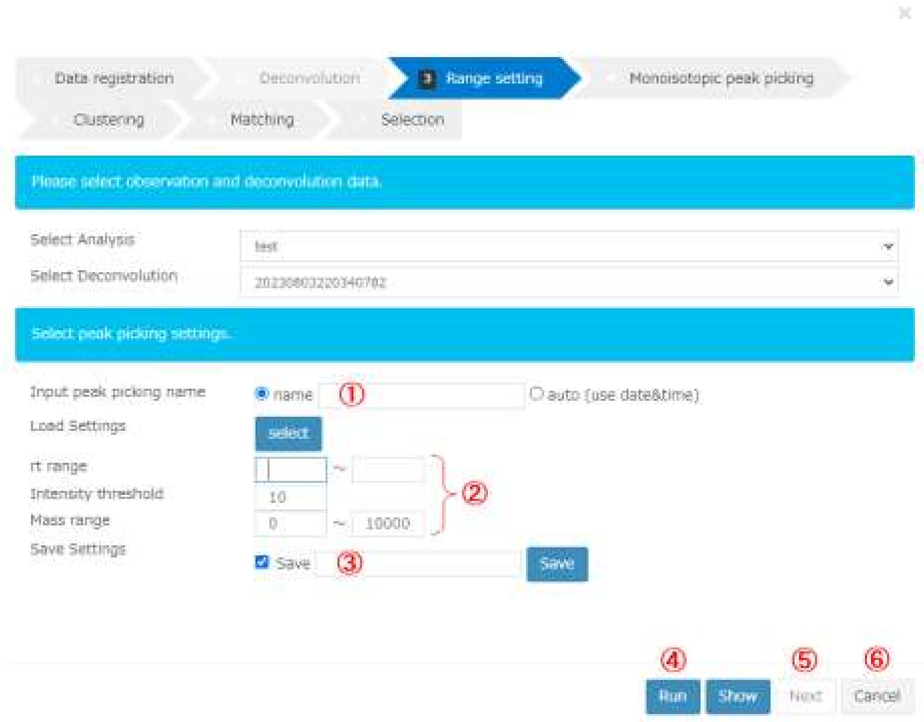

Figure 3-3. Range setting module.

Table 3-1. Details of range settings.

No.	Parameter	Description
1	rt range	Set RT range (min) of data used for analysis
2	Intensity threshold	Set signal intensity threshold of data used for analysis
3	Mass Range	Set mass range (upper and lower limits) of data used for analysis

3.5. Monoisotopic peak picking (Step 4)

3.5.1. Functional overview

This step (Figure 3-4) is intended to find and group signals of the identical ion based on three parameters of time (scan), mass (MH+), and intensity, and to obtain the monoisotopic mass of each signal group at the peak time, using our unique algorithms. At the previous step, signals without isotope signals were removed as noise, so all signals have one isotope signal at least. First, all signals within the analysis range are surveyed to find local peak signal. If a peak signal is found, the same signals within the error tolerance (set at 5 ppm typically) are repeatedly searched from the neighboring scans (toward both sides of RT) and grouped together. The same searches are performed for other isotope signals of the peak signal and obtain

a single signal group. The minimum size of the signal group is set to 3 scans × 4 isotope signals as default. Three scan spectra centered on each group peak scan are then accumulated and the resulting spectra are analyzed by the shape analysis module. In this module, monoisotopic signals are searched from the highest signal toward lower mass. The monoisotopic signal threshold (lowest intensity) to the highest signal is calculated from the oligomer of carbamidomethylcysteine with the lowest number of carbons per 100 masses (=3.1, mean = 4.3, maximum = 6.1 (Phe)). Default values are shown in a setting module (Figure 3-5). If the relative intensity of a candidate monoisotopic signal is less than this value, the signal is not considered as monoisotopic and the search ends. After this search, a monoisotopic peak list of all ions is created (which can be exported as a table consisting of peak RTs, monoisotopic masses, and peak intensities). The picked monoisotopic peak signal can be seen in the viewer overlaid on all signals.

3.5.2. Operation procedure

To perform monoisotopic peak picking, press the wizard button (Figure 2-2①) from the main window to display the monoisotopic peak picking module (Figure 3-4). Recommended parameters are set as a default.

① Select the “Monoisotopic peak picking” tab.
② Enter an analysis name.
③ Set parameters (Table 3-2). Note that “Load settings” is unapplicable in this version.
④ Select whether the setting is saved.
⑤ Click the “Start” button to start analysis. When “Analysis complete” is displayed, the process is finished.
⑥ If you want to cancel this step in progress, press the "Cancel" button.

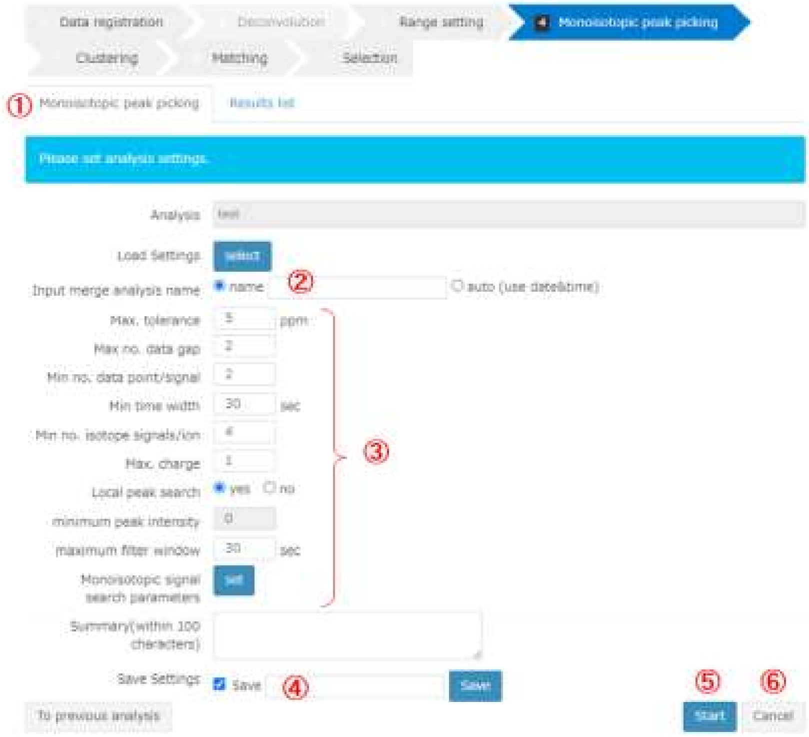

Figure 3-4. Monoisotopic peak picking module.

Table 3-2. Details of monoisotopic peak picking settings.

No.	Parameter	Description
1	Max. tolerance	Sets the maximum mass tolerance among a series of scans for treating signals as ones derived from the identical ions.
2	Max no. data gap	Sets the number of allowable lack of scans for treating signals within a single ion group.
3	Min no. data point/signal	Sets the minimum number of scans of a single group.
4	Min time width	It is unnecessary to set this parameter because it is not utilized for data processing in this version.
5	Min no. isotope signals/ion	Sets the minimum number of isotopes.
6	Max. charge	This parameter should be set as 1, because only deconvoluted data is used for analysis.
7	Local peak search	If "yes" is selected, local peak detection is performed using the Maximum filter and uses it as the starting point for the monoisotopic peak search. If "NO" is selected, all signals are used as the starting point for monoisotopic peak search in descending order of intensity. Note that it is recommended to select "yes", because this process takes much time without the Maximum filter.
8	minimum peak intensity	The minimum intensity of signal as a starting point for monoisotopic peak search.
9	maximum filter window	Specifies the filter size (time width) of the Maximum filter for local peak search.
10	Monoisotopic signal search parameters	Define the parameters for determination of the monoisotopic peak position based on the relative intensities of isotopes in a dialog box (Figure 3-5).

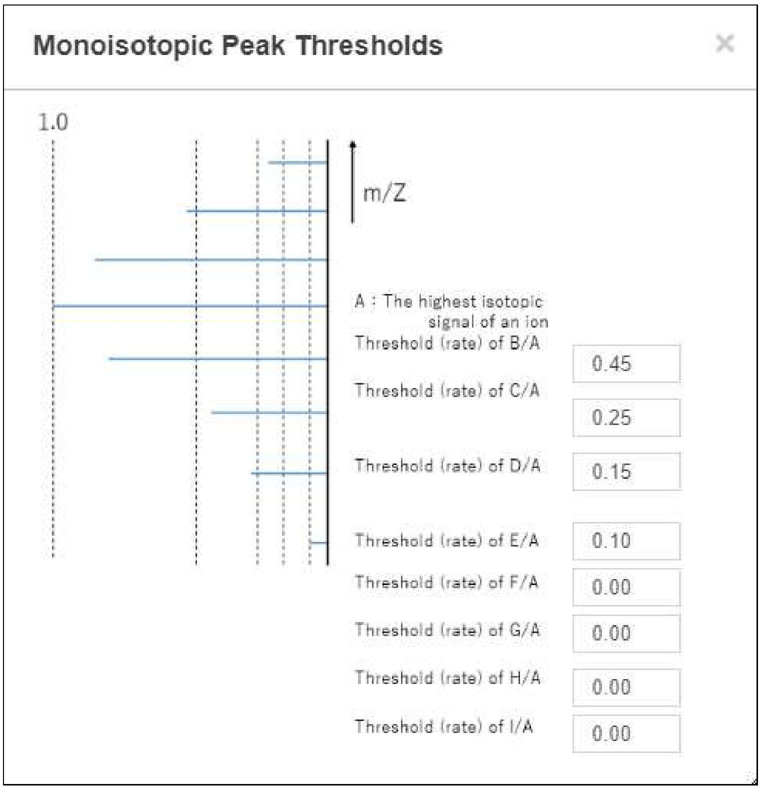

Figure 3-5. Setting window for monoisotopic signal search parameters.

3.6. Clustering (Step 5)

3.6.1. Functional overview

In this step (Figure 3-6), a series of signals of glycopeptide group are found as a cluster based on the elution behavior and mass difference of its members. By setting maximum RT difference and maximum error of mass difference for glycan units (such as Hex, HexNAc, and dHex), a pair of putative glycopeptide group signals is searched for all monoisotopic peaks. If a single signal (node) has multiple relations (edges), the pairs are combined to form a cluster. By repeating similar search, a group with at least a user-defined number of members is considered as a single glycopeptide cluster. The minimum number of members in a cluster is set to 4 as a default.

3.6.2. Operation procedure

To perform clustering, press the wizard button (Figure 2-2①) from the main window to display the clustering module (Figure 3-6).

① Select the “Clustering” tab.
② Enter an analysis name.
③ Set parameters (Table 3-3). Note that “Load settings” is unapplicable in this version.
④ Select whether the setting is saved.
⑤ Click the “Start” button to start analysis. When “Analysis complete” is displayed, the process is finished.
⑥ If you want to cancel this step in progress, press the "Cancel" button.

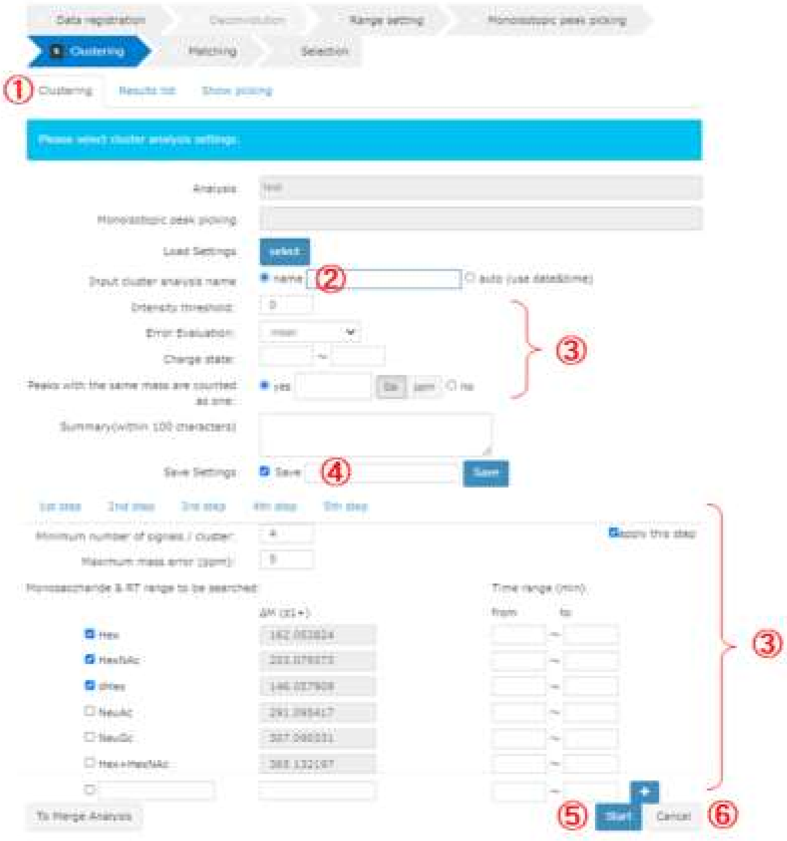

Figure 3-6. Clustering module.

Table 3-3. Details of clustering settings.

No.	Parameter	Description
1	Intensity threshold	Sets the minimum intensity threshold.
2	Error Evaluation	Sets the error evaluation method ("mean" or "sum SQ of SQ"). The default setting is "mean", which is a stricter setting. GRable calculates the difference between M1 and M2 (masses of two monoisotopic signals). To calculate the difference in ppm, the difference (ΔΔM obs-calc) between the observed mass difference (ΔM obs=M2-M1) and calculated mass value of any glycan unit (e.g., M(Hex); set by user as shown below) is divided by either value of:<br>- mean: (M1+M2)/2<br>- sum SQ of SQ: $\sqrt{(M1)^2 + (M2)^2}$<br>If the difference (ppm) is smaller than the maximum mass tolerance (set by user as shown below), the two monoisotopic signals are considered in the relationship with a difference of the glycan unit.
3	Charge state	Note that this parameter should remain blank, because only deconvoluted data is used for analysis.
4	Peaks with the same mass are counted as one	Selects "yes" or "no". If "yes" is selected (by default), peaks with the same mass, but divided to multiple peaks, are considered as single peak (composition) when the number of members of a cluster is evaluated. Note that peaks with the same mass are highlighted in yellow in an exported sheet.
5	1st step (2nd step…)	Sets the parameters for each step of parallel clustering.<br>By clicking, you can switch tabs and enter settings for each step.
6	apply this step	Checks the checkbox for each step whether the step is included in parallel clustering. Users can apply up to five steps in one analysis.
7	Minimum number of signals / cluster	Sets the minimum number of signals for one cluster.
8	Maximum mass error(ppm)	Sets the maximum mass tolerance.
9	Monosaccharide & RT range to be searched	Specifies types of glycan units (mono- and oligo-saccharides) along with their masses and RT ranges to be considered. Hex, HexNAc, dHex, NeuAc, NeuGc, Hex+HexNAc can be entered by default, and the number of free entry fields can be increased up to 10.

3.7. Matching (Step 6)

3.7.1. Functional overview

In this step (Figure 3-7), GRable searches combination of core peptide and glycan composition that match with the mass of putative glycopeptide within the allowed mass error (user setting), according to the following equation:

Observed M(glycopeptide) = calculated M(core peptide identified) + M(Hex)*i + M(HexNAc)*j + M(dHex)*k + M(NeuAc)*l (where M is a mass value, and i, j, k, and l are integers)

To do this, a list of core peptide candidates is required. The list can be prepared by the PNGase-mediated deglycosylation followed by LC/MS analysis of the same glycopeptide sample. Identification of deglycosylated peptides by LC/MS is one to two orders of magnitude more sensitive than glycopeptide identification, allowing core peptide listing using only 5-10 % of the sample used for glycopeptide analysis. As described in Selection step below, RT differences between glycopeptides and deglycopeptides are one of clue for selecting correct match, so LC/MS data is recommended to be obtained on the same day and sequential runs of analysis. In addition, a glycan point list is also needed. Glycan compositions considered for matching are given as the number range of each glycan units (user setting). As default, the ranges of Hex, HexNAc, dHex, and NeuAc are set to 0-10, 1-10, 0-4, and 0-4, respectively. Then, using the set of 3 masses of glycopeptides, core peptides, and glycans, matched combination are searched according to the equation 1. For each glycopeptide, point is given according to the glycan composition matched. The points are set in the glycan point list (given by user). Glycan points are classified into three kinds; 1 = compositions produced by common/familiar biological glycan processing, 0 = possible but not common, and -1 = unusual from common biological pathway. Users can set this allocation of points, e.g., higher points are assigned to the compositions which were observed in actual glycome analysis. All matched results can be obtained as an Excel file.

3.7.2. Operation procedure

To perform matching, press the wizard button (Figure 2-2①) from the main window to display the matching module (Figure 3-7).

① Select the “Matching” tab.
② Enter an analysis name.
③ Set parameters (Table 3-4). Note that “Load settings” is unapplicable in this version.
④ Select whether the setting is saved.
⑤ Click the “Start” button to start analysis. When “Analysis complete” is displayed, the process is finished.
⑥ If you want to cancel this step in progress, press the "Cancel" button.

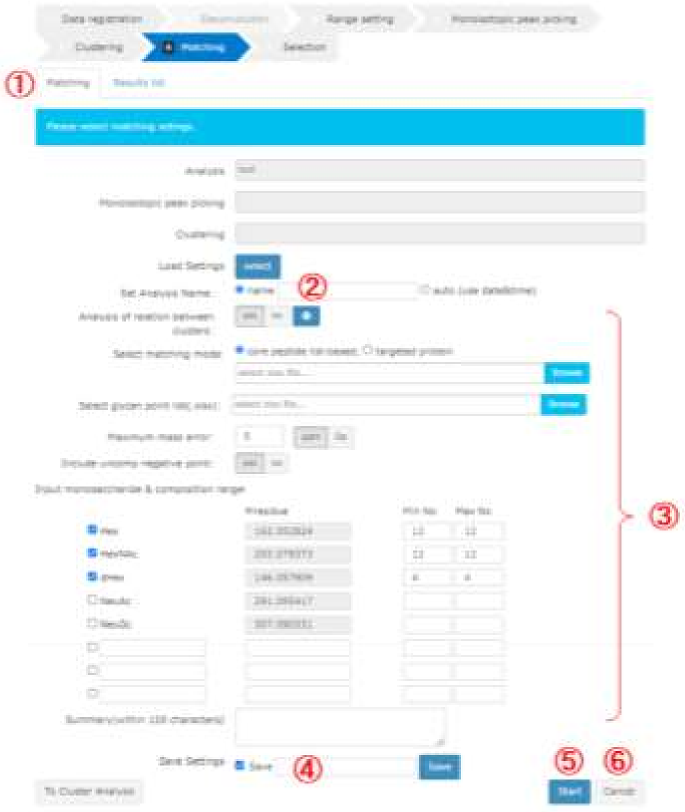

Figure 3-7.　Matching module.

Table 3-4.　Details of matching settings.

No.	Parameter	Description
1	Analysis of relation between clusters	Selects whether inter-cluster analysis is executed. If "yes" is selected, enter the settings by clicking on the button on the right side to display the setting dialog (Figure 3-8). In the search difference setting, specify types of relations to be considered along with mass tolerance (Da). "Delta RT (min)" is a RT range for evaluating other cluster members. "Minimum rate of related members" is a threshold of the parentage of members detected in this search; when the members over the threshold are detected, reference and target clusters are treated as "related" and the target cluster is indicated in a export file.
2	Select matching mode	Select "core peptide list-based" mode and upload a core peptide list (in a fixed format). Note that "target protein" mode is not available in this version.
3	Select glycan point list	Uploads a glycan point list.
4	Maximum mass error	Sets the maximum mass tolerance (ppm or Da).
5	Include uncomp negative point	Selects whether negative points are given for unusual glycan compositions defined in a glycan point list (in a fixed format).
6	Input monosaccharide & composition range	Specifies types of glycan units (mono- or oligo-saccharide) along with their masses and minimum/maximum numbers to be considered. Hex, HexNAc, dHex, NeuAc, and NeuGc can be entered by default, and there are also three free entry fields. Be sure that the glycan units are totally identical to the ones written in a glycan point list; unless an alert will appear.

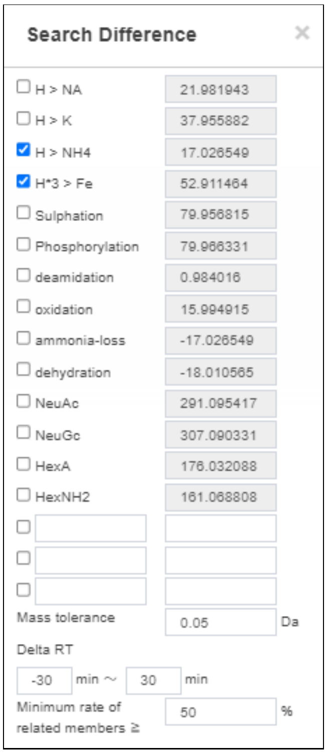

Figure 3-8. Setting dialog of inter-cluster analysis.

3.8. Selection (Step 7)

3.8.1. Functional overview

In the matching step, multiple combinations are often suggested for one glycopeptide cluster. To select the most plausible combination among the candidates, GRable collects the following information from the results and additional MS2 information to evaluate their reliability at the cluster level as well as the single glycopeptide level.

Information

1) **Core peptides abundance:** The number of core peptides identified from a small aliquot of glycopeptide sample by the IGOT-LC/MS/MS method greatly exceeds the number of clusters detected by the Glyco-RIDGE method. This suggests that detected glycopeptide clusters are likely to have core peptides with high abundance or high ionization efficiency among the peptides contained in the sample. Accordingly, if multiple core peptides were matched to single glycopeptide group (cluster), the most abundant core peptide is most plausible, and thus the results of the most abundant core are displayed at the top of the list.
2) **Glycan abundance:** In the process of IGOT-LC/MS/MS for preparing a core peptide list, the major glycan compositions (glycomes) of the sample can be determined by collecting the released glycans and analyzing them, for example, by MALDI-MS. As is the case with core peptide abundance, it is expected that the major glycan compositions are likely to attach to major glycopeptides. Thus, GRable utilize a glycan point list, in which glycan compositions that are detected actually in the glycome of sample are given a point. In the matching module, the sum of the points within the cluster is calculated as a total score, which is used for selection of the plausible match. That is, a match with higher score is considered better among candidate matches for each cluster. When an actual glycan composition list is unavailable, a glycan point list can be made considering the N-glycan processing pathways; in the list, common and unusual compositions have positive and negative points, respectively. In this selection module, users can select whether negative points are considered or not.
3) **RT difference between glycopeptides and corresponding core peptides:** Since the RT of a core peptide is close to those of neutral glycopeptides and often delayed behind them, core peptides with the RT outside the setting are unlikely to be true. However, when a sialic acid is added to a glycopeptide, the RT becomes longer, which is contrast with the case when a neutral glycan is added. Thus, if the matched glycans are sialylated, it should be paid attention to evaluate using RT difference.
4) **Mass accuracy:** In the clustering and matching steps, mass tolerance is usually set to 5 ppm, which is slightly tighter than that used for database searches in proteome analysis (i.e., 7 ppm). To guarantee highly reliable results, in the selection step, delta mass is usually set to 2 ppm. For this reason, MS1 should be acquired under the higher resolution, as described above (Section 2.2).
5) **Utilization with MS2 information:** Although this Glyco-RIDGE method is performed only with MS1 information in the clustering and matching steps to listing the glycopeptide candidates, MS2 information is also utilized for selecting the most plausible matching. In the selection step, it is checked whether MS2 information is obtained for each cluster member. In this module, users can select whether MS2 information is used for analysis, and if it is acquired, the following information is retrieved and used for selection.

i. Presence of diagnostic ions: The presence or absence of diagnostic ions derived from glycans (e.g., HexNAc(204) and Hex+HexNAc(366)) and their signal intensity are obtained automatically for all the MS2 scans. This is supportive information for each matching result, confirming that the ion is derived from a glycopeptide.

ii. Presence of glycopeptide ions: GRable checks the presence of ions of a peptide and a peptide remaining the innermost GlcNAc of N-glycan, called Y0 and Y1, respectively, and ions that occur frequently around them (e.g., Y1+Fuc and Y2). If two of these ions are found and the mass of observed or calculated Y0 is identical to that of a matched peptide within a given mass tolerance, the result is a strong endorsement of the matching result.

iii. DB search results: GRable does not identify a glycopeptide directly based on MS2 spectrum analysis, but it collects the MS2-based search results for the evaluation of the matching results. In a selection result sheet, a peptide sequence corresponding the Y0 mass in a core peptide list is provided, which facilitates easy evaluation of the result.

3.8.2. Operation procedure

To perform selection, press the wizard button (Figure 2-2①) from the main window to display the selection module (Figure 3-9).

① Select the “Selection” tab.
② Enter an analysis name.
③ Set parameters (Table 3-5). Note that “input Analysis Setting” is unapplicable in this version.
④ Select whether the setting is saved.
⑤ Click the “Start” button to start analysis. When “Analysis complete” is displayed, the process is finished.
⑥ If you want to cancel this step in progress, press the "Cancel" button.

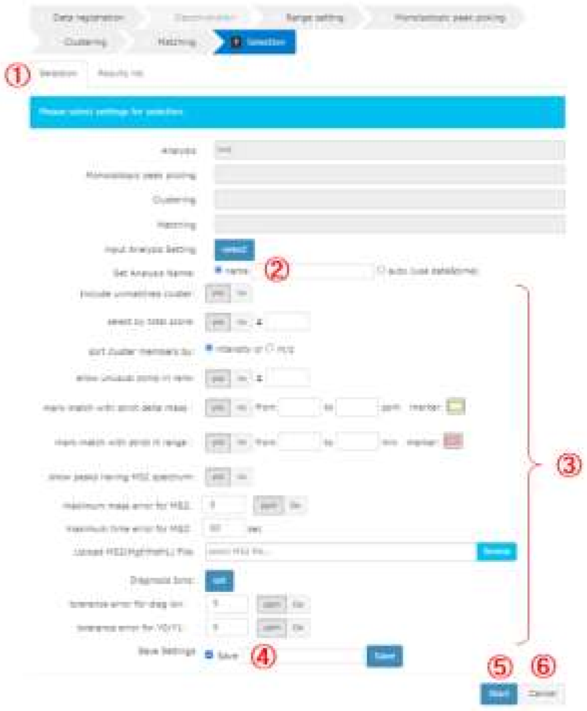

Figure 3-9. Selection module.

Table 3-5. Details of Selection setting.

No.	Parameter	Description
1	Include unmatched cluster	If "yes" is selected, only the cluster information will be included in matching results for clusters that did not match at all.
2	select by total score	Selects whether a threshold of total score is used for selection. If "yes" is selected, matching results contain only matches whose total score is greater than or equal to the threshold value set by the user. For clusters that have no matches above the prescribed score, only the cluster information will be included in matching results.
3	sort cluster members by	Selects the sorting method for cluster members.<br>- "intensity": sort in descending order of intensity<br>- "m/z": sort in descending order of mass
4	allow unusual comp in rank	If "no" is selected, signals with rank equal to or greater than the user's specified number and with unusual comp will be not contained in selection results.
5	mark match with strict delta mass	If "yes" is selected, matches that have difference of masses (delta(ppm)) between observed and theoretical glycopeptides within the mass range (specified by user in ppm) will be considered "plausible" and marked with color (specified by user) in an export file. Note that this parameter is used only for marking to allow manual inspection, and thus all the selected glycopeptide signals (even with out of range) are included in the selection results.
6	mark match with strict rt range	If "yes" is selected, matches that have difference of RTs (delta(RT)) between a glycopeptide and core peptide within the RT range (specified by user in min) will be considered "plausible" and marked with color (specified by user) in an export file. Note that this parameter is used only for marking to allow manual inspection, and thus all the selected glycopeptide signals (even with out of range) are included in the selection results.
7	show peaks having MS2 spectrum	To utilize MS2 information, selects "yes" and specifies the subsequent parameters. If "yes" is selected, glycopeptide signals with MS2 information will be marked with "1" in a "MS2?" column of a selection result sheet.
8	maximum mass error for MS2	Sets the maximum mass tolerance (ppm or Da) for searching corresponding MS2 spectra for each clustered glycopeptide. If the difference of masses (deltaMH+(Da)) between a clustered glycopeptide (m/z) and calculated one (MH+calc) based on MS2 information (i.e., charge and precursor mass) are smaller than the threshold, the corresponding MS2 information will be indicated in a "MS2 info for cluster" sheet.
9	maximum time error for MS2	Sets the maximum RT tolerance (sec) for searching corresponding MS2 spectra for each clustered glycopeptide. If the difference of RTs (delta rt) between a clustered glycopeptide (rt) and MS2 signal

No.	Parameter	Description
		(rt(min)) are smaller than the threshold, the corresponding MS2 information will be indicated in a "MS2 info for cluster" sheet.
10	Upload MS2(Mgf/MzML) File	Uploads MS2 data (in mgf or mzML format). When a mzML format file is used, be sure to use MS2 data before deconvolution.
11	Diagnosis Ions	Specifies glycan fragment ions used as diagnostic ions to confirm that the MS2 spectra is surely derived from a glycoprotein. The panel that appears with the "set" button allows setting type of diagnostic ions with their mass (Da) (Figure 3-10).
12	tolerance error for diag ion	Sets the mass tolerance (ppm or Da) between theoretical and observed fragment ions for evaluating the presence of diagnostic ions specified by user as above.
13	tolerance error for Y0/Y1	Sets the mass tolerance (ppm or Da) between theoretical and observed fragment ions for evaluating the presence of Y0 and its related ions.

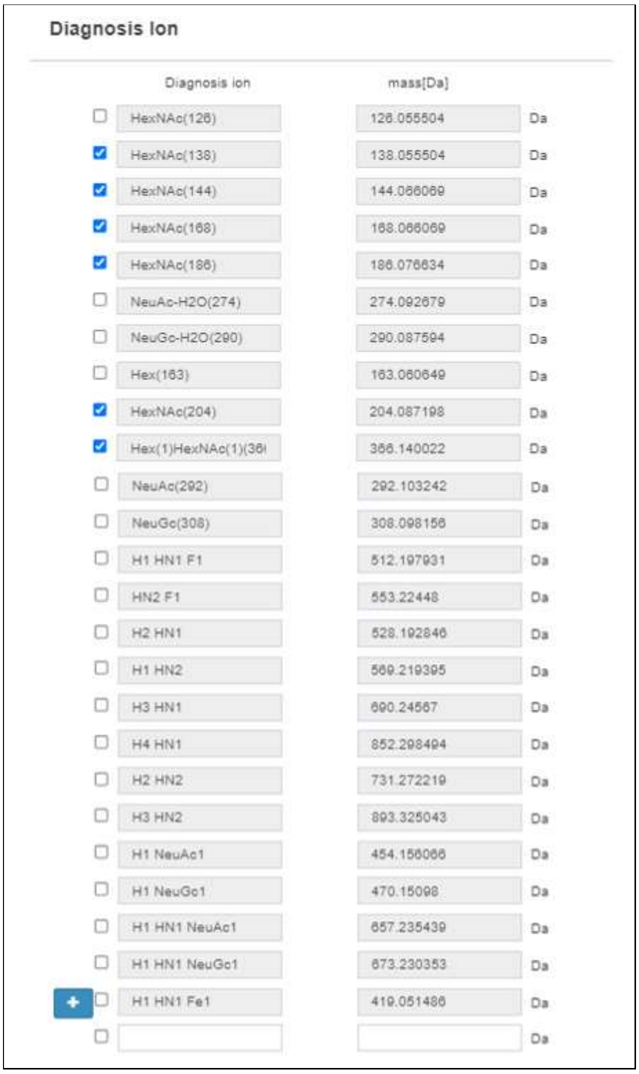

Figure 3-10. Setting dialog of diagnosis ion.

3.9. Data tree

Data tree displays results of each step for one Analysis project in a tree-view format. To display the tree, move the cursor to the tree button on the left of the main window (Figure 2-2⑪) to display the data tree (Figure 3-11). By right clicking data of interest in the tree, a menu will appear for executing functions (Table 3-6).

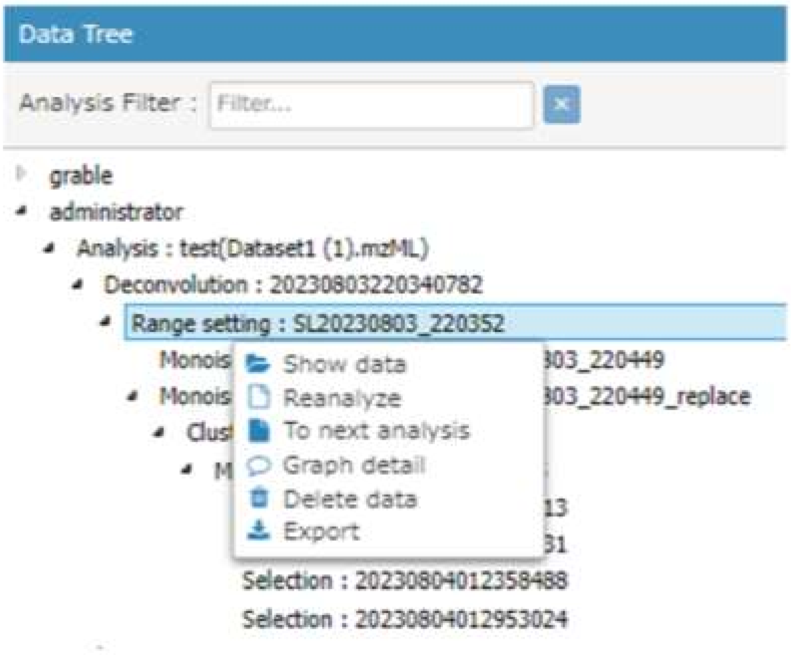

Figure 3-11. Representative image of data tree.

Table 3-6. Details of data tree functions.

No.	Function	Description
1	Show data	This function is to display the selected result data on a viewer in the main window. Details of the viewer are described in Section 3.10. This operation is valid only for results of “range setting”, “monoisotopic peak picking”, “clustering”, and “matching” steps.
2	Reanalyze	This function is to re-analyze the selected data with another different setting in a selected step. The parameters shown in the setting dialog are the same ones with the selected data.
3	To next analysis	This function is to proceed to next step using the selected result data.
4	Graph detail	This function is to display the settings of selected analysis in a separate dialog.
5	Delete data	This function is to delete selected data.
6	Export	This function is to export input data and analysis results along with search parameter settings. To export them, check the items to be exported in the download dialog (Figure 3-12) and click on the “download” button. Details of exported files are described in Section 5. Note that the file size of peak list is large and thus it is recommended to uncheck for the peak list unless necessary. The peak list is separately exported as a .csv file. The other selected items are exported in one Excel file.

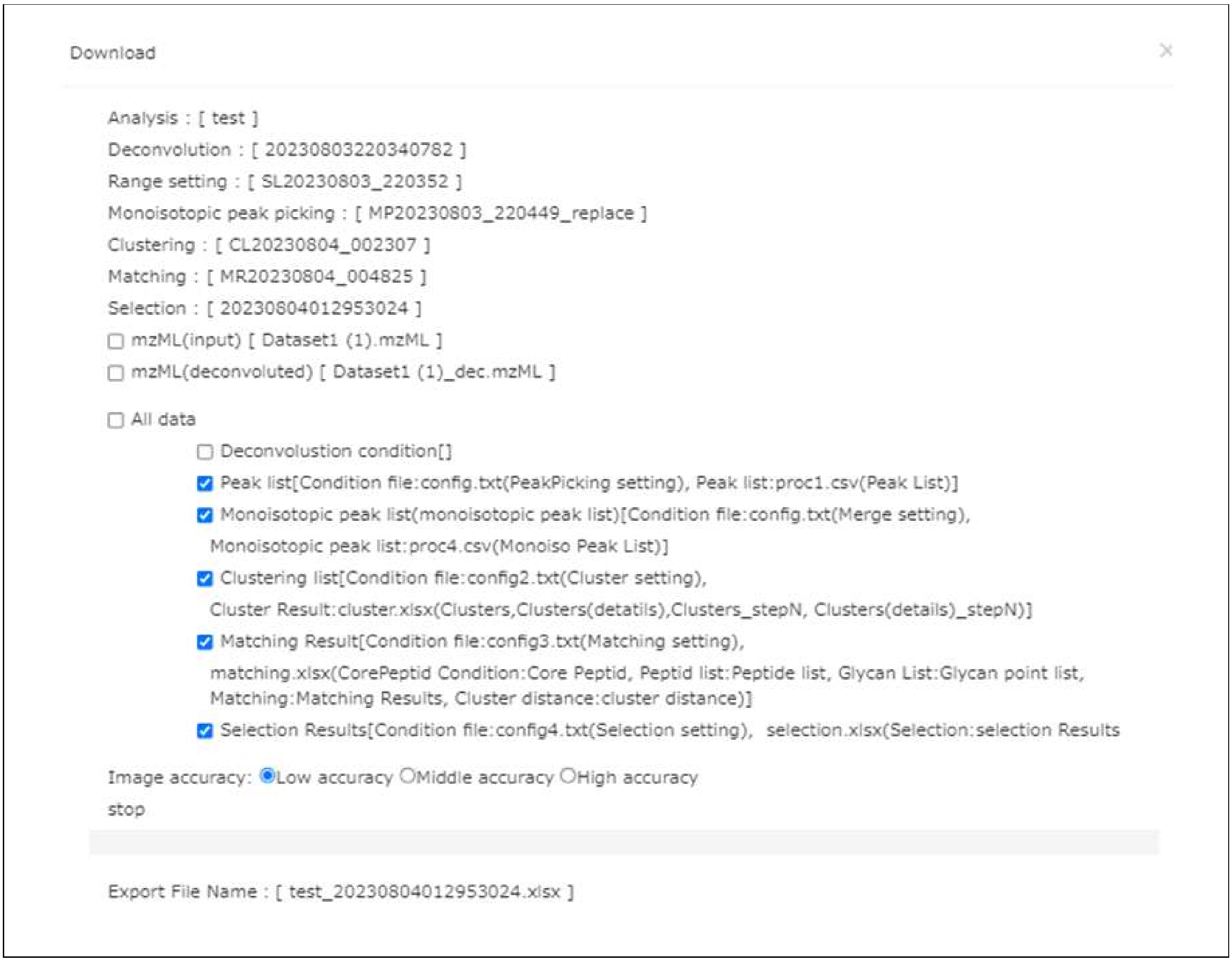

Figure 3-12. Download dialog.

3.10. Viewer

The viewer in the main window (Figure 3-13) is equipped for a graphical visualization of analysis results. The data to be shown can be selected in a tree view as documented above (Section 3.9) and its visualization can be optimized using functional buttons (Table 3-13).

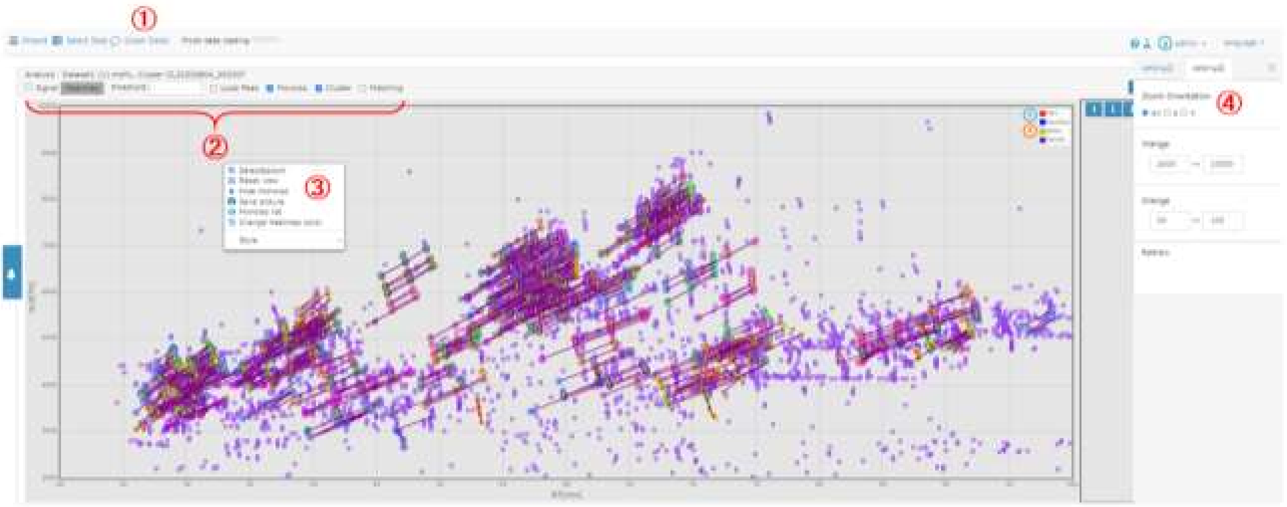

Figure 3-13. Viewer in the main window.

Table 3-13. Details of viewer functions.

No.	Function	Description
1	Graph Detail	This function is to display the settings of selected analysis in a separate dialog.
2	Panels	"Analysis" indicates analysis name along with the name of selected results. A "Heatmap" button and threshold entry form are valid only when the "Signal" is checked. User can the display mode of data among "Local Peak", "Monoiso", "Cluster", and "Matching".<br><br>- **Local Peak**: Displays all the detected local peaks.<br>- **Monoiso**: Displays all the detected monoisotopic peaks in black circle.<br>- **Cluster**: Displays all the detected glycopeptide clusters, in which line colors indicate the relationship of glycan units between two glycopeptide signals.<br>- **Matching**: Displays only the matched glycopeptide clusters when the "Cluster" is unchecked.<br><br>Note that display of "Signal" and "Local Peak" is slow and thus it is recommended to uncheck these buttons.
3	Select&zoom	This function is to display the enlarged graph within range specified by using a mouse.
3	Reset view	The function is to return to default setting.
3	Hide monoiso	This function is to display only monoisotopic peaks by hiding clusters.
3	Save picture	This function is to save the image of viewer as a PNG file.
3	Monoiso list	This function is to display a dialog of monoisotopic peak list. Details of the operation of this list are described in Section 3.11.
3	Change heatmap color	This function is unapplicable in this version.
3	Style	Using this function, the style of monoisotopic peaks can be changed.
4	Zoom Orientation	Specifies the orientation when the viewer is enlarged and reduced using a mouse wheel.
4	Y range / X range	Specifies the range of data (X: RT and Y: m/z) to be visualized. When the "Redraw" button is clicked, the setting values will be applied.

3.11. Monoisotopic peak list

3.11.1. Functional overview

The monoisotopic peak list (Figure 3-14) displays monoisotopic peaks obtained by the monoisotopic peak picking (Step 4) in a dialog format. To display this list, show the analysis results after Step 4 according to the aforementioned instruction (Section 3.10) and then press the "option" button in the main window (Figure 2-2⑨). This dialog is moveable, expandable and contractable. The items of the list are summarized in Table 3-14.

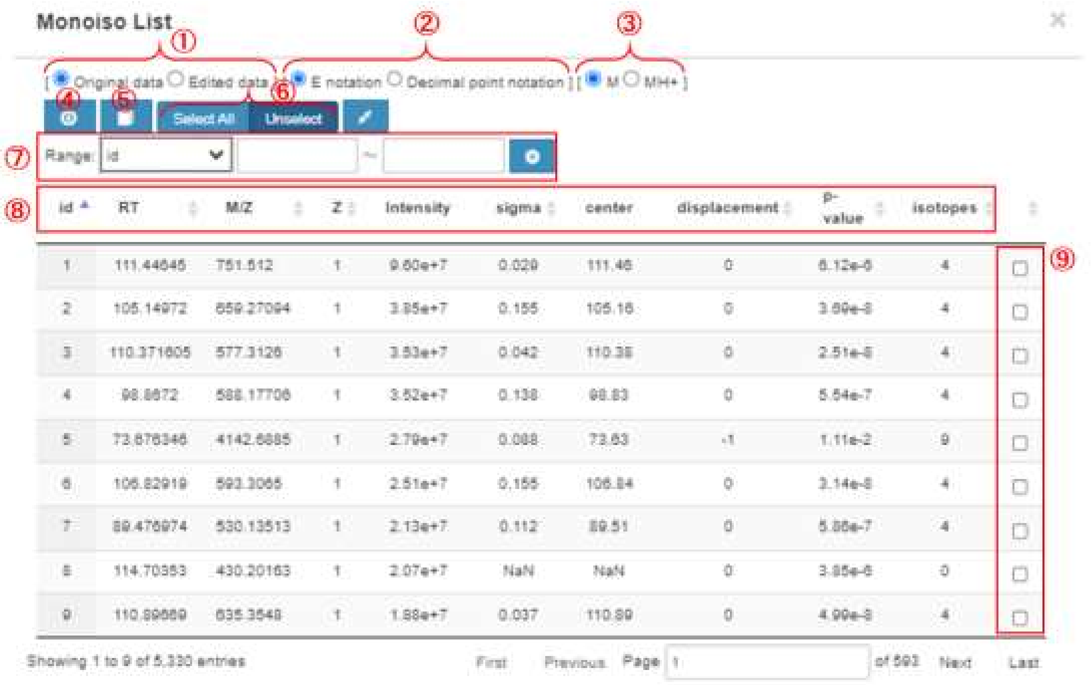

Figure 3-14. Monoisotopic peak list.

Table 3-14. Details of monoisotopic peak list items.

No.	Function / Item	Overview
1	Data switching	This function is to switch between original data and edited data. In an initial state, original data are displayed.
2	Decimal display switching	The display of “intensity” can be switched between E format and decimal format. An initial state is the display in the E format.
3	M / MH^+ switching	The display of “M/Z” can be switched between M and MH^+. An initial state is the display in the M format.
4	Data download	The monoisotopic peak list can be downloaded in .csv format.
5	Save edits	The modified list can be saved. This button is used for data correction function described below.
6	Select All / Unselect	All signals in the list can be selected or unselected together. This button is used for data correction function described below.
7	Filter	Signals in the list can be filtered for each item with ranges specified by user. To use this function, select an item in the dropdown list and then enter the range you want to display in the textbox. The filtering can be reset by press the “x” button. Note that only half-width numbers can be entered. This button is used for data correction function described below.
8	id	ID number of the isotope group
8	RT	RT of the isotope group
8	M/Z	m/z of the monoisotopic peak
8	Z	Charge state of the monoisotopic peak.
8	Intensity	Signal intensity of the monoisotopic peak
8	Sigma	Width of chromatographic distribution (average approximately 0.1)
8	Center	RT of the monoisotopic peak.

No.	Function / Item	Overview
8	displacement	The degree of monoisotopic shift expressed as a difference from a multinomial distribution fitting curve in the spectrum. The theoretical spectrum is created based on abundance ratios of four isotopes including C, N, O, and S.
8	p-value	The *P*-value for the fitting of an observed spectrum to its theoretical spectrum.
8	isotopes	The number of isotopes
9	Check box	Each ID can be selected/unselected individually by checking/unchecking this check box. This function is used for data correction function described below.

3.11.2. Details of the correction function

The data correction function can be used in the monoisotopic peak list (Figure 3-15) according to the following procedure.

① Select “replace” in the dropdown list.

② Enter the range of “displacement” values to be corrected. At once the range is entered, the list is updated.

③ Click the “Select All” to check all the selected IDs. Note that this step will take time.

④ When the update is finished, “SelectAll Complete” will be appeared and all the check box in the list will be checked.

⑤ Click the “Save edits” button, enter an analysis name, and then click the “create” button to save the edited data as a new one. Note that this step will take time.

⑥ The updated data will appear in the data tree of the analysis (Section 3.9).

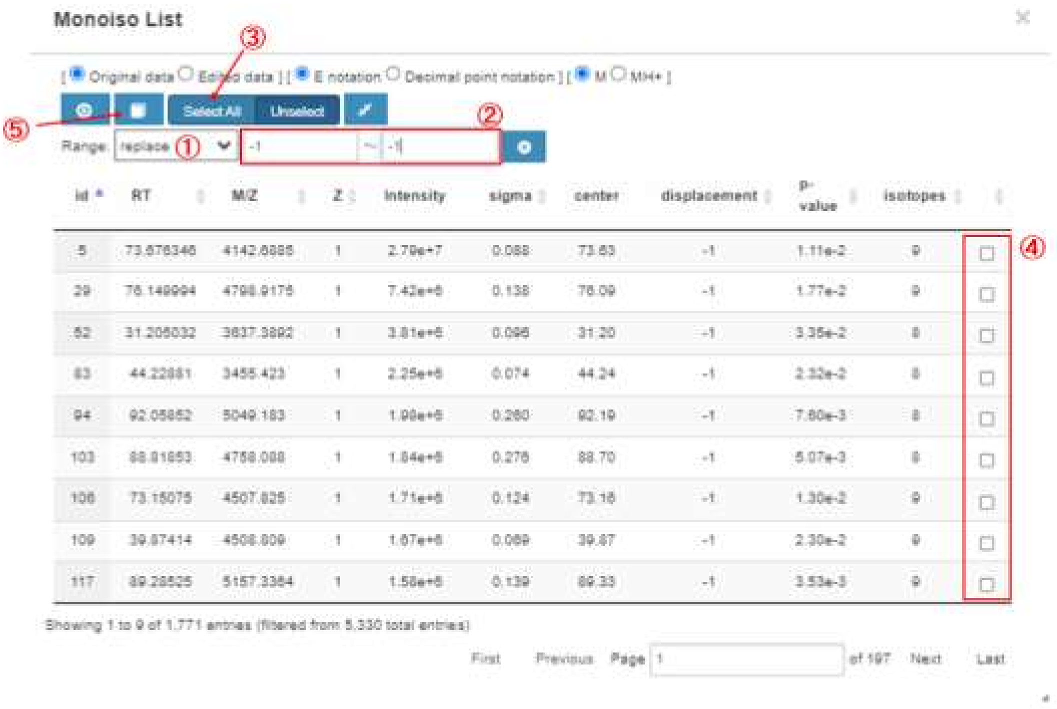

Figure 3-15. Procedure of correction function.

4. Preparation details of imput files

4.1. Core peptide list (.xlsx)

To prepare the core peptide list used for matching (Step 6), we recommend performing isotope-coded glycosylation site-specific tagging (IGOT)-LC-MS/MS as follows. An aliquot of glycopeptides is treated with peptide-*N*-glycosidase F (PNGase F) in ^{18}O-labeled water to remove *N*-glycan and to label deglycosylated Asn as ^{18}O-labeled Asp (i.e., IGOT). The IGOT-treated deglycopeptides is analyzed by LC/MS/MS and the MS2 data were used for database search using Mascot. Using Mascot export file, the core peptide list is created in a fixed format (Figure 4-1). In the list, some items should be manually added to Mascot export file, some of which are mandatory for analysis (Table 4-1). If IGOT-LC-MS/MS is unavailable, the core peptide list can be creased based on proteome results. For analysis of a highly purified glycoprotein, it is also possible to list up peptides predicted based on protease digestion patterns, albeit there is a caution for RT (See Table 4-1). Note that unknown parameters should be indicated as “0” (not to remain blank) to ensure correct performance.

Note that items minimally required for preparing a core peptide list are the calculated mass, retention time of each peptide and at least one peptide identifier such as CP (core peptide) number. Accordingly, other information on core peptides including protein accession number, protein description, peptide position, modification, signal intensity, and protein sequence, can be liked to assigned glycopeptides after analysis. To simplify this operation, we provide a format of core peptide list for including these information in advance.

	A	B	C	D	E	F	G	H	I	J	K	L	M	N
1	CP No.	prot_acc	prot_desc	pep_star	pep_end	pep_seq	pep_var_mod_pos	Siteseqp	Nigot	pep_calc	Mpep	prot_seq	intensity	RT
2	1	P19652	>sp|P19652|A1AG:	19	38	QIPLCANLVPVPITNATLDR	3.0000000000000000200000.0	33(NAT)/	1	2190.16	2187.17	MALSWV	1.1E+08	89.9836
3	2	P02763	>sp|P02763|A1AG:	19	42	QIPLCANLVPVPITNATLDQITGK	3.000000000000000200000000.	33(NAT)/	1	2561.37	2558.38	MALSWV	3.5E+07	95.6326
4	3	P19652	>sp|P19652|A1AG:	19	38	QIPLCANLVPVPITNATLDR	0.0000000000000000200000.0	33(NAT)/	1	2207.19	2204.2	MALSWV	3E+07	89.9673
5	4	P19652	>sp|P19652|A1AG:	19	42	QIPLCANLVPVPITNATLDRITGK	3.000000000000000200000000.	33(NAT)/	1	2589.41	2586.42	MALSWV	7390537	90.2897
6	5	P19652	>sp|P19652|A1AG:	102	113	ENGTVSRYEGGR	0.02000000000.0	103(NGT	1	1326.61	1323.62	MALSWV	6000682	34.6546
7	6	P00738	>sp|P00738|HPT_H	236	249	VVLHPNYSQVDIGL	0.000002000000000.0	241(NYS)	1	1555.81	1552.82	MSALGA	4919720	69.4705
8	7	P02765	>sp|P02765|FETUA	145	165	VCQDCPLLAPLNDTRVVHAAK	0.000000000000200000000.0	156(NDT	1	2379.19	2376.2	MKSLVLL	3312263	63.1591
9	8	P19652	>sp|P19652|A1AG:	87	101	QNQCFYNSSYLNVQR	0.0000002000000000.0	93(NSS)/	1	1922.85	1919.86	MALSWV	2250460	60.5409
10	9	P19652	>sp|P19652|A1AG:	19	42	QIPLCANLVPVPITNATLDRITGK	0.000000000000000200000000.	33(NAT)/	1	2606.44	2603.45	MALSWV	1832450	90.0245
11	10	P19652	>sp|P19652|A1AG:	26	38	LVPVPITNATLDR	0.0000000200000.0	33(NAT)/	1	1410.8	1407.81	MALSWV	1713041	64.1101

Figure 4-1. Representative image of core peptide list.

Table 4-1. Details of core peptide list.

No.	Manually addition	Mandatory	Item	Description
1	Required	No, but reccomended*	CP No.	Core peptide number. *Note that this information is not required for matching but recommended to be included as a peptide identifier. It is also recommended to be manually numbered in descending order of signal intensity, as an indicator of the relative abundance of signal intensity.
2			prot_acc	Protein accession No.
3			prot_desc	Protein description
4			pep_start	Start position of peptide within a protein
5			pep_end	End position of peptide within a protein

No.	Manually addition	Mandatory	Item	Description
6		No, but reccomended*	pep_seq	Peptide sequence. *Note that this information is not required for matching but used in the Selection step; when a peptide having the identical mass to predicted Y0 mass is found in a core peptide list, the peptide sequence will be shown in the Selection results sheet.
7			pep_var_mod_pos	Position of the following variable modifications:<br>1: ammonia-loss (peptide N-term, carbamidomethyl C)<br>2: Delta:H(-1)N(-1)18O(1)(N)<br>3: Gln->pyro-Glu (peptide N-term, Q)<br>4: oxidation (M)
8	Required		Siteseqpos	Position of IGOT-labeled Asn residue (+2.98822096000004) within a protein along with its consensus sequence
9	Required		Nigot	Number of IGOT-labeled Asn residues. Note that this parameter is not mandatory for analysis but used for calculation of glycan compositions after subtraction of Hex(3)HexNAc(2) (trimannosyl core) in the Matching results sheet.
10			pep_calc_mr	Calculated mass of peptide
11		yes	Mpep	Calculated mass of peptide without IGOT labeling. Note that this information is the most important to obtain results.
12			prot_seq	Protein sequence
13		No, but reccomended*	intensity	Signal intensity. *Note that this information is not required for matching but recommended to be included as an indicator of the abundance of peptide. When candidate core peptides are identified using Mascot. MS2 total ion current can be used because of the ease of availability.
14	Required	yes*	RT	RT (min) of MS2 fragment ion. Note that RT (sec) in mascot export files should be converted into RT (min). *Note that for analysis of purified glycoproteins, analysis may work well without RT, although it causes a decrease in the reliability of results.

4.2. Glycan point list (.xlsx)

The glycan point list used for the matching (Step 6) can be prepared based on glycans observed in glycome analysis of the same glycoprotein sample and/or the biosynthetic pathway of glycans. In the glycan point list (Figure 4-2), glycan compositions (highlighted in pink) are listed along with a given point in a "point" raw. Unusual glycan compositions can be also specified by listing along with a negative point in a "unusual" raw. These unusual glycan compositions are used for analysis only when the "uncomp negative point" parameter is "yes" in the matching setting (Table 3-4). Note that the name of glycan unit (e.g., Hex, HexNAc, dHex, and NeuAc) should be the same as those in the matching setting (Table 3-4).

	A	B	C	D	E	F	G
1		point	Hex	HexNAc	dHex	NeuAc	
2	point	1	0	1	0	0	
3	point	1	0	1	1	0	
4	point	1	0	2	0	0	
5	point	1	0	2	1	0	
6	point	1	1	2	0	0	
7	point	1	1	2	1	0	
8	point	1	2	2	0	0	
9	point	1	2	2	1	0	
10	point	1	3	2	0	0	
11	point	1	3	2	1	0	
12	point	1	4	2	0	0	
13	point	1	4	2	1	0	
14	point	1	5	2	0	0	
15	point	1	5	2	1	0	
16	point	1	6	2	0	0	
17	point	1	6	2	1	0	
18							
19	unusual	-1	9	2	1		
20	unusual	-1	9	2	2		
21	unusual	-1	9	2	3		
22	unusual	-1	9	2	4		
23	unusual	-1	8	2	1		
24	unusual	-1	8	2	2		
25	unusual	-1	8	2	3		
26	unusual	-1	8	2	4		

Figure 4-2. Representative image of glycan point list.

4.3. LC/MS and LC/MS/MS data of glycopeptides (.mzML and .mgf)

The LC/MS data (.raw) should be deconvoluted and exported as an mzML format using Proteome Discoverer (PD). In the PD Ver.2.4, the settings of workflow tree and the advanced parameters of the Xtract node were set as indicated below (Figure 4-3). In this workflow tree, the deconvoluted .mzML and .mgf files were exported in the Spectrum Exporter (2) and (5) nodes, respectively.

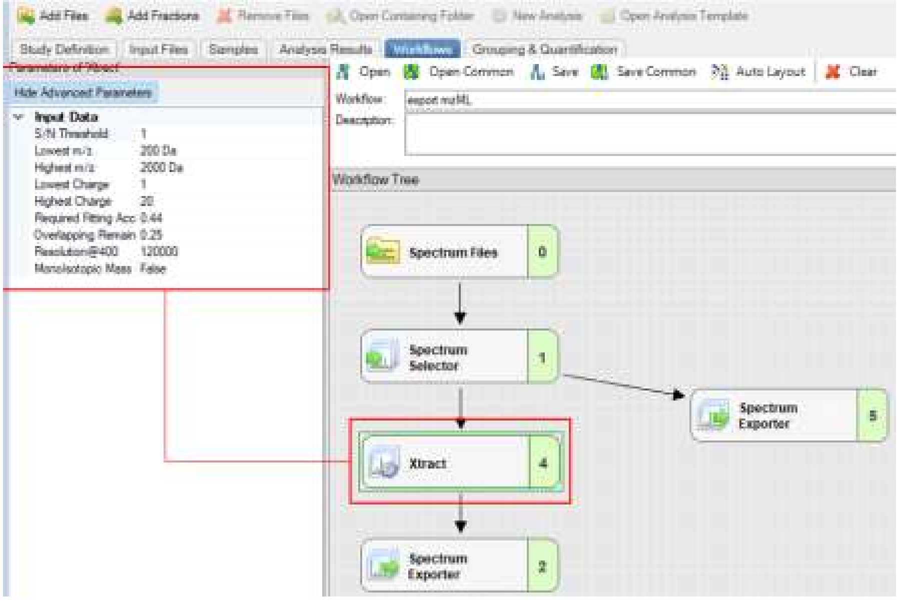

Figure 4-3. Workflow tree and Xtract parameters

The other parameters, except Max. Precursor Mass (set as 0 Da), are set as default (Figure 4-4).

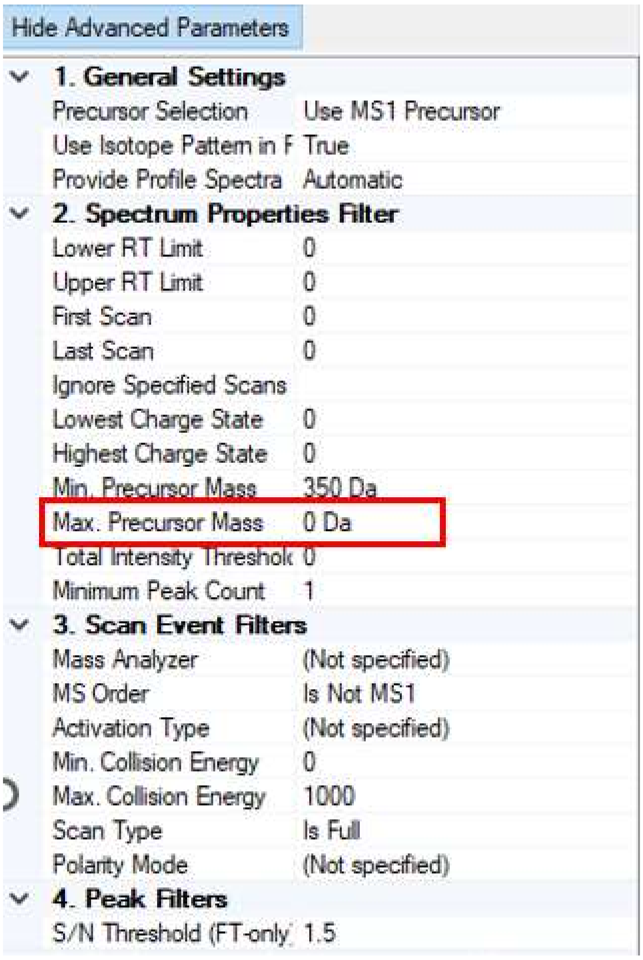

Figure 4-4. Spectrum selector parameters

Note that it is recommended to export both MS1 and MS2 data of Thermo Fisher's raw data within one mzML file. In the mzML file, the individual MS1 scans are kept and the MS2 information are also included. However, GRable utilizes the MS2 information in the .mgf file. The reason why the MS2 data should be separately uploaded in .mgf format is because we noticed that the deconvolution inadequately affects MS2 information written in a mzML file (e.g., the m/z for HexNAc(204) is changed to 205). In addition, when only MS2 information is exported in a .mzML format, scan No. will be re-numbered, leading to the disconnect to MS1 information.

5. Details of export files

Analysis results can be exported using the "Export" function in the data tree (Section 3.9). Peak list (Table 5-1) is separately exported as a .csv file. The other selected items (Table 5-2) are exported in one Excel file.

Table 5-1. Details of peak list.

Item	Description
SCAN	Scan number of MS1 spectra
RT	RT (min) of a local peak
MASS	Observed mass (m/z) of a local peak

Item	Description
INTENSITY	Signal intensity of a local peak
GID	Glycopeptide ID that is the same with the “monoiso no” in the “Monoiso Peak List” sheet
FLAG	This parameter is not worked.

Table 5-2. List of data exported in an Excel file.

Sheet name	Related step	Description	Details of items
Range setting	Range setting	Parameters specified for this step	
Monoiso Peak List	Monoisotopic peak picking	List of monoisotopic peaks detected in this step	Table 5-3
Monoiso Peak Picking log	Monoisotopic peak picking	Log information for this step	
Monoiso Peak Picking setting	Monoisotopic peak picking	Parameters specified for this step	
Clustering setting	Clustering	Parameters specified for this step	
Clusters	Clustering	List of clusters detected in this step	Table 5-4
Clusters(details)	Clustering	Detailed information of clusters detected in this step	Table 5-5
Clusters_stepX	Clustering	List of clusters detected only in the applied setting (stepX)	Table 5-4
Clusters(details)_stepX	Clustering	Detailed information of clusters detected only in the applied setting (stepX)	Table 5-5
Matching setting	Matching	Parameters specified for this step	
Glycan point list	Matching	Glycan point list identical to an input file	See Section 4.2
Peptide list	Matching	Core peptide list identical to an input file	See Section 4.1
Matching Results	Matching	List of all matches of glycopeptide cluster, core peptide, and glycan compositions	Table 5-6
relation between clusters	Matching	Inter-cluster analysis results (*Included only when inter-cluster analysis is applied)	Table 5-7
Selection setting	Selection	Parameters specified for this step	
Selection Results	Selection	List of matches selected based on the specified parameters	Table 5-6
MS2 info for clusters	Selection	MS2 information for each selected cluster	Table 5-8
All MS2 info	Selection	MS2 information for all scans in input MS2 data	Table 5-8
logging	Selection	Log information for this step	
peptide chart	Selection	2D-map (RT – Mass of predicted Y0 ion) for core peptides observed in the “All MS2 info” sheet	Table 5-8

Table 5-3. Details of monoisotopic peak list.

Item	Description
monoiso no	Monoisotopic peak No. that are numbered in a descending order of peak intensity
monoiso retention time	RT (min) of monoisotopic peak
monoiso mass	Mass (M) of monoisotopic peak
peak intensity	Signal intensity of a local peak
monoiso rtid	This parameter is not worked.
monoiso intensity	Signal intensity of a monoisotopic peak
charge	Charge state
center of distribution	RT of the center of chromatographic distribution
sigma of distribution	Width of chromatographic distribution (average approximately 0.1)
valid groups	Number of isotopes
multinomial pvalue	The P-value for the fitting of an observed spectrum to its theoretical spectrum.
composition	This parameter is a part of algorithm for the fitting of an observed spectrum to its theoretical spectrum.
suggest monoiso displacement	The degree of monoisotopic shift expressed as a difference from a multinomial distribution fitting curve in the spectrum.
suggested monoiso mass	Mass of a monoisotopic peak selected after check using the correction function
pvalue suggested monoiso	The P-value for a suggested monoisotopic peak
suggested monoiso signal found	This parameter is not worked. (Always “0”)
original Mz	Mass of a monoisotopic peak selected before the correction function
update flag	If there is an update using the correction function, “1” is indicated.

Table 5-4. Details of Clustering result sheets.

Item	Description
cluster_no	Cluster No. that is numbered in a descending order of the maximum of intensity within the cluster.
peak_no	Monoisotopic peak No. that is the same as “monoiso no” in the monoisotopic peak list.
charge	Charge state of the monoisotopic peak
m/z	m/z of the monoisotopic peak<br>(*When “Peaks with the same mass are counted as one” is “yes” in the setting, the masses having the same mass withing the cluster are highlighted in yellow)
rt	RT (min) of the monoisotopic peak

Item	Description
intensity	Signal intensity of the monoisotopic peak
no_member	No. of members for the cluster<br>(*When "Peaks with the same mass are counted as one" is "yes" in the setting, the masses having the same mass withing the cluster are counted as one)
step	Step No. that found the monoisotopic peak

Table 5-5. Details of Clustering result (details) sheet.

Item*	Description
cluster_no	Cluster No.
charge	Charge state of the monoisotopic peak
peak_no	Monoisotopic peak No. that is the same as "monoiso no" in the monoisotopic peak list.
rt	RT (min)
scan_no	Scan No.
m/z	Mass (MH^+)
mass	Mass (M)
intensity	Signal intensity
target_peak_no	Monoisotopic peak No. that is the same as "monoiso no" in the monoisotopic peak list.
rt	RT (min)
scan_no	Scan No.
m/z	Mass (MH^+)
mass	Mass (M)
intensity	Signal intensity
residue	A glycan unit equivalent to the difference between reference and target peaks
diff_mass	Difference in observed masses between reference and target peaks
diff_theor	Theoretical mass difference between reference and target peaks, which corresponds to the theoretical mass of the glycan unit
delta	Delta value (Da) between "diff_mass" and "diff_theor".
diff(ppm)	Delta value (ppm) converted from "delta" by the method selected for "Error Evaluation" of the setting
diff_rt	Difference in RTs between reference and target peaks
step	Step No. that found the monoisotopic peak

*Red: information on reference peaks. Blue: information on target peaks examined for relationship with reference peaks.

Table 5-6. Details of matching and selection result sheets.

Item *1	Description
cluster_no	Cluster No.
member	No. of the members within the cluster
peak_no	Monoisotopic peak no. that is the same as “monoiso no” in the monoisotopic peak list.
charge	Charge state
scan_no	Scan No. of the monoisotopic peak
m/z	Mass (MH^+)
rt	RT (min)
intensity	Signal intensity
M(gpep, obs)	Observed mass (M)
step	Step No. that found the monoisotopic peak
CP No.	Core peptide No.
prot_acc	Protein accession No.
prot_desc	Protein description
pep_start	Start position of peptide within a protein
pep_end	End position of peptide within a protein
pep_seq	Peptide sequence
pep_var_mod_pos	Position of the following variable modifications:<br>1: ammonia-loss (peptide N-term, carbamidomethyl C)<br>2: Delta:H(-1)N(-1)18O(1)(N)<br>3: Gln->pyro-Glu (peptide N-term, Q)<br>4: oxidation (M)
Siteseqpos	Position of IGOT-labeled Asn residue (+2.98822096000004) within a protein along with its consensus sequence
Nigot	Number of IGOT-labeled Asn residues
pep_calc_mr	Calculated mass of peptide
Mpep	Calculated mass of peptide without IGOT labeling
prot_seq	Protein sequence
intensity	Signal intensity of MS2 fragment ion
RT	RT (min) of MS2 fragment ion. Note that RT (sec) in mascot export files should be converted into RT (min)
rank	Ion score rank in the mascot search results
Hex	No. of Hex residues in the matched glycan composition
HexNAc	No. of HexNAc residues in the matched glycan composition
dHex	No. of dHex residues in the matched glycan composition
NeuAc	No. of NeuAc residues in the matched glycan composition
Hex[-core]	No. of Hex residues after subtracting by the trimannosyl core of N-glycan (i.e. -3) in the matched glycan composition
HexNAc[-core]	No. of HexNAc residues after subtracting by the trimannosyl core of N-glycan

Item *1	Description
	(i.e. -2) in the matched glycan composition
dHex	No. of dHex residues in the matched glycan composition
NeuAc	No. of NeuAc residues in the matched glycan composition
unusual comp	If the glycan composition is defined as “unusual” in the glycan point list, “1” will be indicated.
point	A point given for the glycan composition
factor	A coefficient value for weighting a glycan composition of interest. The factor is “1” unless other values are specified in the glycan point list. Note that this function is not guaranteed in this version.
score	A score for the glycan composition calculated by multiplying “point” and “factor”
total score	The sum of scores for all the members of the cluster
mass	Calculated mass (M) of theoretical glycopeptide in a combination of the core peptide and glycan composition
delta(Da)	Delta value (Da) between observed and calculated masses for the glycopeptide
delta(ppm)	Delta value (ppm) converted from delta(Da)
delta(RT)	Delta value (min) between RTs of the glycopeptide and core peptide
MS2? *2	If the MS1 spectra has any MS2 information, “1” will be indicated with a link to the corresponding data in the “MS2 info for clusters” sheet.
predict peptide *2	This is identical to the “peptide[seq]” information in the “MS2 info for clusters” sheet.
Y0 (z=1) *2	Y0 mass (MH^+; z=1) calculated using the mass of core peptide
Y0 (z=2) *2	Y0 mass (MH^+; z=2) calculated using the mass of core peptide
Y0 (z=3) *2	Y0 mass (MH^+; z=3) calculated using the mass of core peptide
Y1 (z=1) *2	Y1 mass (MH^+; z=1) calculated using the mass of core peptide
Y1 (z=2) *2	Y1 mass (MH^+; z=2) calculated using the mass of core peptide
Y1 (z=3) *2	Y1 mass (MH^+; z=3) calculated using the mass of core peptide

*1 Red: information on glycopeptide clusters. These items are the identical to those in the “Cluster(details)” sheet (Table 5-5). Blue: information on core peptides and glycan compositions. These items are the identical to those in the “Peptide list” sheet (Table 4-1).

*2 These items will be indicated only when the “show peaks having MS2 spectrum” is “yes” in the setting (Table 3-5).

Table 5-7. Details of inter-cluster analysis result sheet.

Item	Description
cluster_no*	Cluster No.
#member*	No. of members in the cluster
group_no*	Group No. for annotating each glycopeptide (C, cluster; G, group)<br>(*The peaks with the same mass are counted as one.)
peak_no*	Monoisotopic peak No. that is the same as “monoiso no” in the monoisotopic

Item	Description
	peak list.
charge*	Charge state
m/z*	m/z of the monoisotopic peak
rt*	RT (min) of the monoisotopic peak
intensity*	Signal intensity of the monoisotopic peak
#members	Non-redundant No. of the combination of reference and target peaks with a relationship specified by user. When the ratio of members is over the threshold specified in the setting (Figure 3-8), these peaks are considered as “related”.
Delta m/z	Difference in masses (m/z) between reference and target peaks
Relation	The relationship between reference and target peaks, which is identified by comparing the “Delta m/z” with the value entered in the setting.
Direction	Direction of relationship that indicates which peak is a naked or adduct)
Delta Delta m/z	Delta value (Da) between the “Delta m/z” and the value entered in the setting.<br>When this value is lower the threshold specified in the setting, these peaks are considered as “related”.
Delta rt	Delta value (min) of RTs between reference and target peaks. When this value is within the range specified in the setting, these peaks are considered as “related”.

*Column A-H indicate items for Reference cluster, whereas column I-P are for Target clusters examined whether it has a relationship specified by user.

Table 5-8.　Details of MS2 information sheets.

Item *1	Description
cluster_no	Cluster No.
peak_no	Monoisotopic peak No. that is the same as “monoiso no” in the monoisotopic peak list.
charge	Charge state
m/z	Observed mass (MH^+) of the monoisotopic peak
rt	RT (min) of the monoisotopic peak
intensity	Signal intensity of the monoisotopic peak
no_member	Ascending numbering for monoisotopic peaks in the cluster
precursors	Ascending numbering for precursor ions for one monoisotopic peak
MH+calc	Calculated mass (MH^+) of the glycopeptide based on the mass of precursor ion and its charge state
deltaMH+(Da)	Delta value (Da) between observed and calculated masses (MH^+) of the monoisotopic peak
delta rt	Delta value (min) between RTs of a monoisotopic peak (MS1) and its corresponding MS2 spectra
scan	Scan No. of MS2 spectra annotated in the uploaded mgf file. Note that the

Item *1	Description
	link is inactive in this version.
rt(min)	RT (min) of MS2 spectra
charge	Charge state of the precursor ion
precursor mass	Mass (MH^+) of the precursor ion in the indicated charge state
Intensity	Signal intensity of the precursor ion
z[Y0/Y1]	Charge state of Y0 and Y1 ions
matches[Y0/Y1]	No. of matches for glycopeptide-derived ions
Y0 mass predict	Predicted mass (M) of Y0 ion
peptide[seq]	CP No. and sequence of the core peptide that has the mass (M) corresponding to the "Y0 mass predict".<br>(*Present only if there is a corresponding core peptide in the list)
peptide[mass]	Observed mass (M) of the core peptide
peptide-Y0 mass	Delta value (Da) between the "peptide[mass]" and "Y0 mass predict"
Y0 mass obs *2	Observed mass of the Y0 ion
Y0 int *2	Signal intensity of the Y0 ion
Y1-Y0 dif *2	Delta value (Da) between masses of Y1 and Y0 ions
isotope count	Ascending numbering of isotopes
HexNAc(138) obs *3	Observed mass of a diagnostic ion (specified by user)
HexNAc(138) dif *3	Delta value (Da) between observed and calculated masses of a diagnostic ion (specified by user). When the delta value is lower than the value of "tolerance error for diag ion" value entered in the setting (Table 3-5), the observed ion will be considered as a diagnostic ion.
HexNAc(138) int *3	Signal intensity of a diagnostic ion (specified by user)
used in cluster *4	Cluster No. that includes a monoisotopic peak corresponding to the MS2 scan
precursor intensity *4	Signal intensity of the precursor ion

*1 Red: information on assigned clusters that are shown in the "Selection Results" sheet.

*2 Similar items are also indicated for other glycopeptide-related ions (Y0x, Y1, Y1F, and Y2).

*3 Similar items are also indicated for other diagnostic ions specified by user.

*4 Indicated only in the "MS2 info for clusters" sheet.
